# Fine Analysis of Genetic Diversity of the *tpr* Gene Family among Treponemal Species, Subspecies and Strains

**DOI:** 10.1371/journal.pntd.0002222

**Published:** 2013-05-16

**Authors:** Arturo Centurion-Lara, Lorenzo Giacani, Charmie Godornes, Barbara J. Molini, Tara Brinck Reid, Sheila A. Lukehart

**Affiliations:** 1 Department of Medicine, University of Washington, Seattle, Washington, United States of America; 2 Pathobiology Graduate Program, University of Washington, Seattle, Washington, United States of America; 3 Department of Global Health, University of Washington, Seattle, Washington, United States of America; Institut Pasteur, France

## Abstract

**Background:**

The pathogenic non-cultivable treponemes include three subspecies of *Treponema pallidum (pallidum, pertenue, endemicum)*, *T. carateum*, *T. paraluiscuniculi*, and the unclassified Fribourg-Blanc treponeme (Simian isolate). These treponemes are morphologically indistinguishable and antigenically and genetically highly similar, yet cross-immunity is variable or non-existent. Although all of these organisms cause chronic, multistage skin and systemic disease, they have historically been classified by mode of transmission, clinical presentations and host ranges. Whole genome studies underscore the high degree of sequence identity among species, subspecies and strains, pinpointing a limited number of genomic regions for variation. Many of these “hot spots” include members of the *tpr* gene family, composed of 12 paralogs encoding candidate virulence factors. We hypothesize that the distinct clinical presentations, host specificity, and variable cross-immunity might reside on virulence factors such as the *tpr* genes.

**Methodology/Principal Findings:**

Sequence analysis of 11 *tpr* loci (excluding *tprK*) from 12 strains demonstrated an impressive heterogeneity, including SNPs, indels, chimeric genes, truncated gene products and large deletions. Comparative analyses of sequences and 3D models of predicted proteins in Subfamily I highlight the striking co-localization of discrete variable regions with predicted surface-exposed loops. A hallmark of Subfamily II is the presence of chimeric genes in the *tprG* and *J* loci. Diversity in Subfamily III is limited to *tprA* and *tprL*.

**Conclusions/Significance:**

An impressive sequence variability was found in *tpr* sequences among the Treponema isolates examined in this study, with most of the variation being consistent within subspecies or species, or between syphilis vs. non-syphilis strains. Variability was seen in the *pallidum* subspecies, which can be divided into 5 genogroups. These findings support a genetic basis for the classification of these organisms into their respective subspecies and species. Future functional studies will determine whether the identified genetic differences relate to cross-immunity, clinical differences, or host ranges.

## Author Summary

Pathogenic treponemes include three subspecies of *Treponema pallidum* (*pallidum*, *pertenue*, *endemicum*), *T. carateum*, *T. paraluiscuniculi*, and the unclassified Fribourg-Blanc treponeme. Although they share morphology and have very similar antigenic profiles, they have traditionally been distinguished by mode of transmission, host specificity and the clinical manifestations that they cause. The molecular basis for these disease characteristics is not known. Comparative genomics has revealed that sequences differences among the species and subspecies are found in very localized regions of the chromosome. Many of these regions of sequence variation are found in the *tpr* genes, which encode a family of twelve candidate virulence factors, many of which are predicted to be outer membrane proteins. Most of the *tpr*-specific sequence changes are consistent within subspecies or species, supporting the historical classification of these organisms into separate subspecies and species. Functional studies are needed to determine whether any of the *tpr* gene differences are related to differences in host range, immunity, or clinical manifestations.

## Introduction

Non-cultivable pathogenic treponemes include three subspecies of *Treponema pallidum*: *T. pallidum* subsp. *pallidum* (*T. p. pallidum*), *T. pallidum* subsp. *pertenue* (*T. p. pertenue*) and *T. pallidum* subsp. *endemicum* (*T. p. endemicum*). These subspecies are human pathogens and cause venereal syphilis, yaws and bejel, respectively. Other very closely related species or isolates are *Treponema paraluiscuniculi* and the Fribourg-Blanc or Simian treponeme. *T. paraluiscuniculi* causes venereal syphilis in rabbits and is reportedly not infectious for humans [1], [2]. The unclassified Simian treponeme was isolated from a baboon, causes a yaws-like disease in non-human primates, and is able to cause active infections in humans [3]–[5]. All of these organisms can be propagated in rabbits and cause disease following experimental inoculation of rabbits. *Treponema carateum* causes the human disease, pinta, but no strains of this organism are available.

The infections caused by *T. pallidum* organisms are characterized by chronic infection with distinct early and late clinical manifestations. Syphilis, usually a sexually transmitted infection, is a highly invasive process and can involve virtually any organ or system including the central nervous system. In pregnant women, early syphilis infection often results in transmission to the fetus. Each year, approximately twelve million new cases of syphilis are estimated to occur globally [6], [7]. Yaws and bejel affect approximately 3 million people worldwide and are transmitted by non-sexual direct contact, usually during childhood and largely affecting people living in remote villages in developing countries. Yaws and bejel have predominantly skin or mucous membrane and osseous manifestations [8]–[10], with tissue destruction late in infection. Pinta causes significant skin discoloration in the late stages, but rarely causes tissue destruction. Unlike syphilis, these infections are said not to affect the central nervous or the fetus [9], although some scientists question this statement [11]. *T. paraluiscuniculi* infection in rabbits appears to be a chronic, but clinically mild, process characterized by long-lasting crusty lesions of the genitalia, nose, and mouth [12]. Treponemal infections in non-human primates have not been traditionally associated with genital disease; however, a recent study by Knauf et al. [13] reports asymptomatic, moderate or severely destructive genital lesions (and perhaps sexual transmission) resembling human syphilis, caused by organisms classified phylogenetically as more closely related to the Fribourg-Blanc and *T. pallidum* subsp. *pertenue* isolates.

The molecular basis for host specificity and the different clinical manifestations caused by these treponemes is not known. These organisms are morphologically identical [1], [3], [14]–[17] with very similar antigenic composition [18]–[23], stressed by the fact that, to date, infection-induced antibody or cellular immune responses cannot distinguish species, subspecies or strains. Protective immunity is induced only by long-term infection and is subspecies-specific [24]. In cross-immunity experiments [1] in which initial infections in the rabbit model lasted at least 3 months, three scenarios are observed: 1) inoculation with a particular strain results in complete protection against re-infection with the homologous strain, 2) protection against re-infection with another strain of the same subspecies is variable or non-existent, and 3) protection against challenge with other species or subspecies is absent. These cross-immunity observations are in concordance with inoculation studies in humans conducted by Magnuson et al. [25]. Subjects with treated late latent syphilis challenged with the Nichols strain had either of two outcomes: 1) those that did not develop either clinical signs or serological evidence of re-infection, indicating immunity; and 2) those that had increases in serological titers and/or development of darkfield positive lesions after inoculation, interpreted as active reinfection with the challenge strain. Although there was no evidence for waning immunity in the subjects who were susceptible to reinfection, this is a possible explanation. However, the lack of cross-immunity among highly similar species/subspecies may also reflect differences in a set of immunologically “inconspicuous” epitopes, underlying immunodominant, but not protective, antigens such as Tp47 (TP0574).These immunodominant antigens may act as decoy systems as described for other bacterial pathogens [26].

Recent comparative analyses of whole genome sequences [27]–[30] (Giacani et al., unpublished) reported <0.1% sequence differences among *T. p. pallidum* strains [29]; <0.2% between *T. p. pertenue* and *T. p. pallidum* subspecies [29]; and <1.2% between *T. paraluiscuniculi* and the human treponemes [31], [32]. Sequence diversity is primarily localized to six hot spots [29], which include regions encoding several members of the *tpr* gene family. The Tpr proteins represent candidate virulence factors, and have been the focus of intense research for the last decade. As a consequence, the distinct clinical presentations, host specificities, and variable cross-immunity studies suggest that foci of sequence diversity, including the *tpr* genes, may be the basis for explaining the differences described above for the treponemal infections.

Sequence homology divides the *tpr* family, a group of twelve paralogs, into three subfamilies: Subfamily I (*tpr C*, *D*, *F* and *I*), Subfamily II (*tprE*, *G* and *J*) and Subfamily III (*tprA*, *B*, *H*, *K* and *L*). As we progressively gain a better understanding of this gene family, an essential role for many of these genes is more apparent. Several studies show that the Tpr antigens are expressed during infection and are able to elicit marked antibody and cellular immune responses in the infected host [33]–[40]. Of the encoded Tpr antigens, TprA, B, C, D, E, F, I, J and K have been predicted to be outer membrane proteins (OMP) [33], [40], [41]. Opsonization and/or vaccine studies with these proteins support surface exposure [33], [37], [42]–[44] and both antigenic variation (TprK) [45] and phase variation (TprE, G, J) [46] mechanisms have been identified in *tpr* members. Yet, the high invasiveness and ability of *T. pallidum* to persist for decades in the host suggest that this spirochete may rely not only on antigenic and phase variation for survival. To influence infection outcomes, *T. pallidum* may also employ other strategies including genetic drift, genetic shift and or pathoadaptive point mutations, which can arise either during long term evolution or rapidly during a single infection. An important body of evidence has accumulated showing genetic variation in specific regions of the *T. pallidum* genome among subspecies and among strains [47]–[56]. The present study demonstrates significant sequence diversity in the *tpr* gene family, which can have important implications in understanding evolution of these organisms, as well as cross-immunity, strain typing and vaccine design.

## Materials and Methods

### Ethics statement

No investigations were undertaken using humans or human samples in this study. New Zealand white rabbits were used for strain propagation. Animal care was provided in accordance with the procedures outlined in the Guide for the Care and Use of Laboratory Animals, and all work was conducted under protocols approved by the University of Washington Institutional Animal Care and Use Committee.

### Bacterial strains and DNA extraction

*T. pallidum* subspecies, *T. paraluiscuniculi*, and the Fribourg-Blanc treponeme were propagated in New Zealand white rabbits by intratesticular inoculation as previously described [57]. DNA was extracted for PCR amplification from the following isolates: *T. pallidum* subsp. *pallidum* (Sea 81-4, Mexico A, Bal 3), *T. pallidum* subsp. *pertenue* (Gauthier, CDC2, Samoa D), *T. pallidum* subsp. *endemicum* (Iraq B, Bosnia A), the Fribourg-Blanc treponeme (Simian isolate) and *T. paraluiscuniculi* (Cuniculi A). These strains were selected to represent different species/subspecies, geographical regions of origin, years of isolation, and anatomical sources (Table 1). The sequences of the *tpr* genes for the *T. p. pallidum* Nichols and Street 14 strains were downloaded from their corresponding genome sequences, GenBank accession numbers NC_000919.1 and NC_010741.1, respectively [27], [28]. Although we determined *tpr* sequences for a number of other strains of *T. pallidum* subsp. *pallidum*, only strains defining the 5 identified genogroups of *T. p. pallidum* are included in this manuscript. To ensure that the correct strain was propagated and extracted, only one strain of treponeme was handled at any time during the propagation and freezing process, and rabbit ear tags as well as labels on tubes were double-checked. Bacteria were extracted from infected rabbit testes in sterile saline, collected in sterile 1.7-ml microcentrifuge tubes, taking precautions to prevent cross-contamination between samples, and spun immediately in a microcentrifuge at 1,000*g* for 10 minutes to remove rabbit debris, followed by centrifugation of the supernatant at 12,000*g* for 30 min at 4°C [57]. Pellets were resuspended in 200 µl of 1X lysis buffer (10 mM Tris [pH 8.0], 0.1 M EDTA, 0.5% sodium dodecyl sulfate), and DNA was extracted with the Qiagen (Chatsworth, Calif.) kit for genomic DNA extraction as described in the manufacturer's instructions, but adding 50 ul of proteinase K (100 mg/ml stock solution) and incubating the sample for 2 h at 65°C. After the final elution step in 200 µl of H_2_O, DNA was used for analysis by PCR and sequencing.

**Table 1:** Treponemal strains reported in this study.

Species, subspecies	Strain name	Source	Location	Year of isolation
*T. p. pallidum*	Nichols^a^	Cerebrospinal fluid	Washington DC	1912
	Sea 81-4^b^	Primary chancre	Seattle	1980
	Bal 3^c^	Blood, congenital	Baltimore	Unknown
	Mexico A^c^	Primary chancre	Mexico	1953
	Street 14^d^	Skin	Atlanta	1977
*T. p. pertenue*	Gauthier^e^	Skin	Brazzaville	Early 1960's
	Samoa D^c^	Skin	Western Samoa	1953
	CDC2^f^	Skin	Ghana	1980
*T. p. endemicum*	Bosnia A^c^	Penile ulcer	Bosnia	1950
	Iraq B^c^	Oral mucous patches	Iraq	1951
Simian species	Fribourg-Blanc^c^	Lymph node	Guinea	1966
*T. paraluiscuniculi*	Cuniculi A^c^	?	Baltimore	Unknown

a Originally provided by James N. Miller, University of California, Los Angeles, CA.

b Strain isolated in Seattle by Sheila A. Lukehart, University of Washington, Seattle, WA.

c Strains provided by Paul Hardy and Ellen Nell, Johns Hopkins University, Baltimore, MD.

d Provided by Sandra A. Larsen, Center for Disease Control and Prevention, Atlanta, GA.

e Provided by Peter Perine, Centers for Diseases Control and Prevention, Atlanta, GA.

f Provided by Robert George and Victoria Pope, Center for Disease Control and Prevention, Atlanta, GA.

### PCR amplification, cloning, sequencing, sequence analysis and 3D models

The Nichols *T. pallidum* genome sequence [27] was used to design primers in the 5′ and 3′ flanking regions of the *tpr* genes to amplify the corresponding DNA regions from genomic DNA of the 10 treponemal strains. Table S1 lists the primers used for amplification and sequencing. Using genomic DNA as a template, whole ORF amplifications were performed in a 50-µl final volume containing 200 µM deoxynucleoside triphosphates, 1.5 mM MgCl_2_, and 2.5 U of GoTaq DNA polymerase (Promega, USA). For larger amplicons such as the *tprG-F* or *tprJ-I* operons [38], the LongAmp Taq PCR Kit was used as instructed by the manufacturer (New England Biolabs, USA). The products were cloned into the pCRII-TOPO or TOPO-XL (long amplicons) cloning vectors (Invitrogen, USA) according to the manufacturer's instructions. Plasmid DNA was extracted by using the Qiagen Plasmid Minikit (Qiagen, USA), and two to ten clones for each strain were sequenced with the Applied Biosystems dye terminator sequencing kit (Perkin-Elmer, USA). Consensus sequences were obtained with the CAP sequence assembly program [58] and ORFs from each strain at each locus were aligned using the MAFFT alignment program [59]. GenBank accession numbers are listed in Table S2. Structural homologs were identified using the 3D jury approach [60]. Structural (3D) models for TprC, TprD and TprI were generated using the TMBpro algorithm [61]. The orientation of the predicted loops in the TMBpro models, surface exposed vs. periplasmic, was determined as previously described by Randall et al. [61]. Signal peptide predictions were performed using the Predisi algorithm [62].

## Results

**Figure 1:**
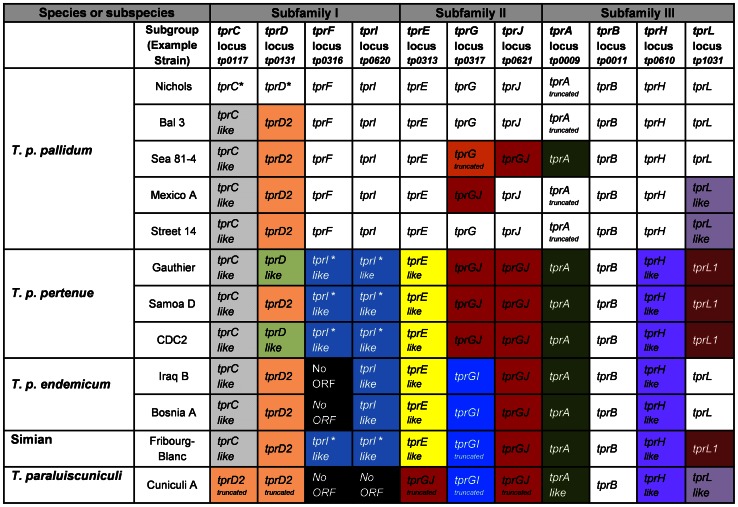
*tpr* alleles among treponemal species, subspecies and strains. *tprD2*: A *tprD* allele which contains a 330-bp unique central region and three smaller heterogeneous regions at the 3′ end. *tprC*-like *and tprD*-like: similar to *tprC* or *tprD*, respectively, with small sequence differences in discrete variable regions (DVRs). *tprGJ*: A chimera where the 3′ end contains *tprJ* signatures. *tprGI*: A chimera where the 5′ end is homologous to *tprG*, and the central and 3′ regions are homologous to the corresponding regions of *tprI*. truncated: Predicted truncated proteins due to a frameshift. *tprA*-like, *tprI*-like and *tprL*-like contain small sequence differences. *tprE*-like and *tprH*-like contains small sequence differences that segregate syphilis from non-syphilis treponemes. *tprL1*: A unique *tprL* allele in *T. p. pertenue* and Fribourg-Blanc strain. * indicates that *tprC* and *tprD* in the Nichols strain and that *tprI*-like sequences in the *tprF* and *tprI* loci are also identical in the *pertenue* subspecies and the Fribourg-Blanc treponeme.

The *tpr* loci defined in the original Nichols genome sequence [27] were used to determine corresponding genes in 11 additional treponemal strains, including four *T. p. pallidum*, three *T. p. pertenue*, two *T. p. endemicum*, one *T. paraluiscuniculi*, and the Fribourg-Blanc strains (Table 1). TprK is excluded from this analysis because of the already extensive work that has been done on this gene [33], [34], [42], [43], [45], [53], [63]–[67]. Sequence analyses of the *tpr* loci from these strains identified significant heterogeneity within and among *pallidum* subspecies, the Fribourg-Blanc isolate and *T. paraluiscuniculi*. Figure 1 summarizes our findings. While Subfamilies I and II display a wide range of changes, diversity in Subfamily III is limited largely to the *tprA* and *tprL* loci. The observed changes are quite diverse, including SNPs of synonymous and non-synonymous character, indels, deletions of entire ORFs, chimeras, and alleles with large unique regions. Readers interested in the entire spectrum of sequence modifications identified in this study are referred to DNA and amino acid sequence alignments for each locus appended as Figure S1. For practical and comparison purposes, the *tpr* loci annotated in the Nichols genome sequence [27] will be considered as the reference ORFs.

It is noteworthy that many of the sequence changes divide the *T. pallidum* strains cleanly by subspecies or species: examples include no *tprI* ORF in *endemicum* strains; and *tprG/J* chimeras in both *tprG* and *tprJ* loci in *pertenue* strains while these two loci contain *GI* and *GJ* chimeras, respectively, in *endemicum* strains. In some cases, the sequences clearly divide syphilis vs. non-syphilis *T pallidum* subspecies: as an example, the absolute conservation of *tprF* (with frameshift) and intact *tprI* sequences in the syphilis strains, while *tprI*-like sequences are found in these loci in non-syphilis strains. Within subspecies, the syphilis strains demonstrated the most heterogeneity, being divided into five genotypes.

### SUBFAMILY I: *tprC* and *tprD* loci and their encoded proteins

Subfamily I *tpr*s include the *tprC*, *D*, *F*, and *I* loci. Initial examination of deduced protein alignments from the Nichols strain showed a significantly high degree of sequence conservation within Subfamily I at the amino and carboxyl termini, with central unique regions [33]; however, discrete heterogeneity was later evident in the amino and carboxyl regions when additional strains were analyzed. The Nichols TprC/D proteins reportedly have porin activity and an OM localization [68]. Although not yet experimentally demonstrated, TprF and I are also predicted to have a cleavable signal peptide and to be surface exposed [33], [40], [41], [44].

**Figure 2:**
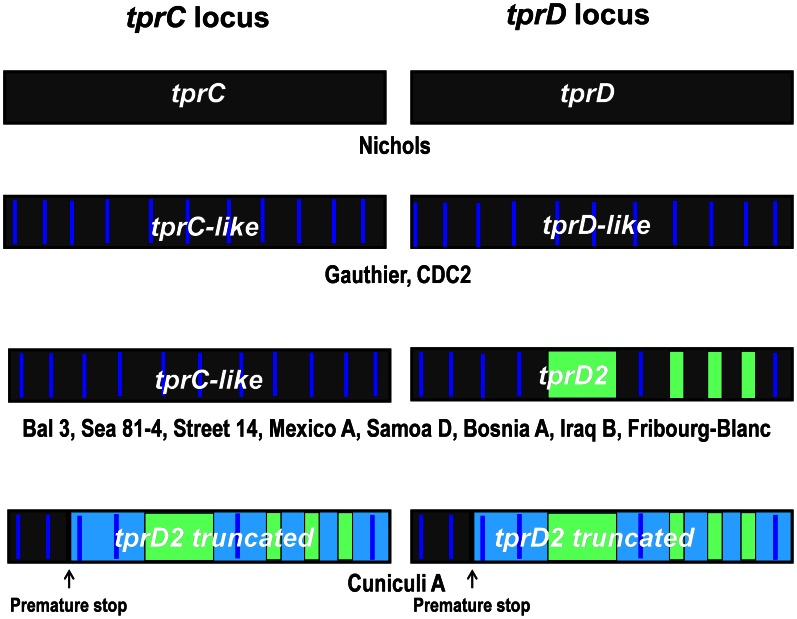
Allelic variants at the *tprC* and *tprD* loci. Four allelic combinations are found at these two loci: 1) identical *tprC* and *tprD*, 2) *tprC*-like and *tprD*-like, 3) *tprC*-like and *tprD2*, and 4) *tprD2* truncated and *tprD2* truncated. Same background color indicates sequence identity. Vertical blue lines indicate discrete variable regions (DVRs), which contain mutations in great majority of non-synonymous character. Green color indicates unique *tprD2* signatures. Light blue background indicates predicted untranslated regions of the ORFs in the *tprD2* alleles due to a single nucleotide insertion, frameshifting and a premature stop.

The *tprC* and *tprD* loci in the reference Nichols genome contain two identical coding sequences [27]. Earlier studies [37], [56] identified *tprC* and *tprD* variants among strains and among the three *pallidum* subspecies. The present study significantly expands our knowledge of the sequences in the *tprC* and *tprD* loci, and a schematic representation of all variants at the *C* and *D* loci identified to date is presented in Figure 2. Among the treponemal strains tested in this study, four alleles are found at the *tprD* locus: the reference *tprD* (Nichols), the *tprD2* allele (Bal 3, Mexico A, Sea 81-4, Street 14, Samoa D, Iraq B, Bosnia A, Fribourg-Blanc), a predicted truncated *tprD2* (Cuniculi A), and the *tprD*-like variants (Gauthier, CDC2). We previously referred to the sequence in the *tprD* locus of Gauthier as *tprD3* [56]. However, we have now found a very similar (but not identical) sequence in the CDC2 strain, and we have chosen to call these “*tprD*-like” sequences, which are further described below. As in Nichols, those *T. p. pallidum* strains that have the Nichols *tprD* allele in the *D* locus also contain an identical copy of *tprD* in the *C* locus [37], and none of the non-syphilis treponemes carries *tprC/D* ORFs identical to the Nichols strain. As previously reported by our group, *tprD2* has four unique regions that differentiate it from Nichols *tprD* and the *tprD*-like sequences: a 330-bp central region and three smaller regions toward the end of the open reading frame (Figure 2) [56]. The *tprC* locus of the *tprD2*-containing Bal 3, Sea 81-4, Street 14 and Mexico A *T. p. pallidum* strains contains *tprC*-like ORFs, with small sequence changes compared to the Nichols *tprC*. [37]. Overall, the sequence homology among *tprC* alleles is >95%. All *pertenue*, *endemicum* and the Fribourg-Blanc strains also have *tprC*-like sequences. As previously reported for *T. paraluiscuniculi*, Cuniculi A strain [31], [32], [36], the *tprC* and *D* loci are occupied by two truncated *tprD2* variants.

**Figure 3:**
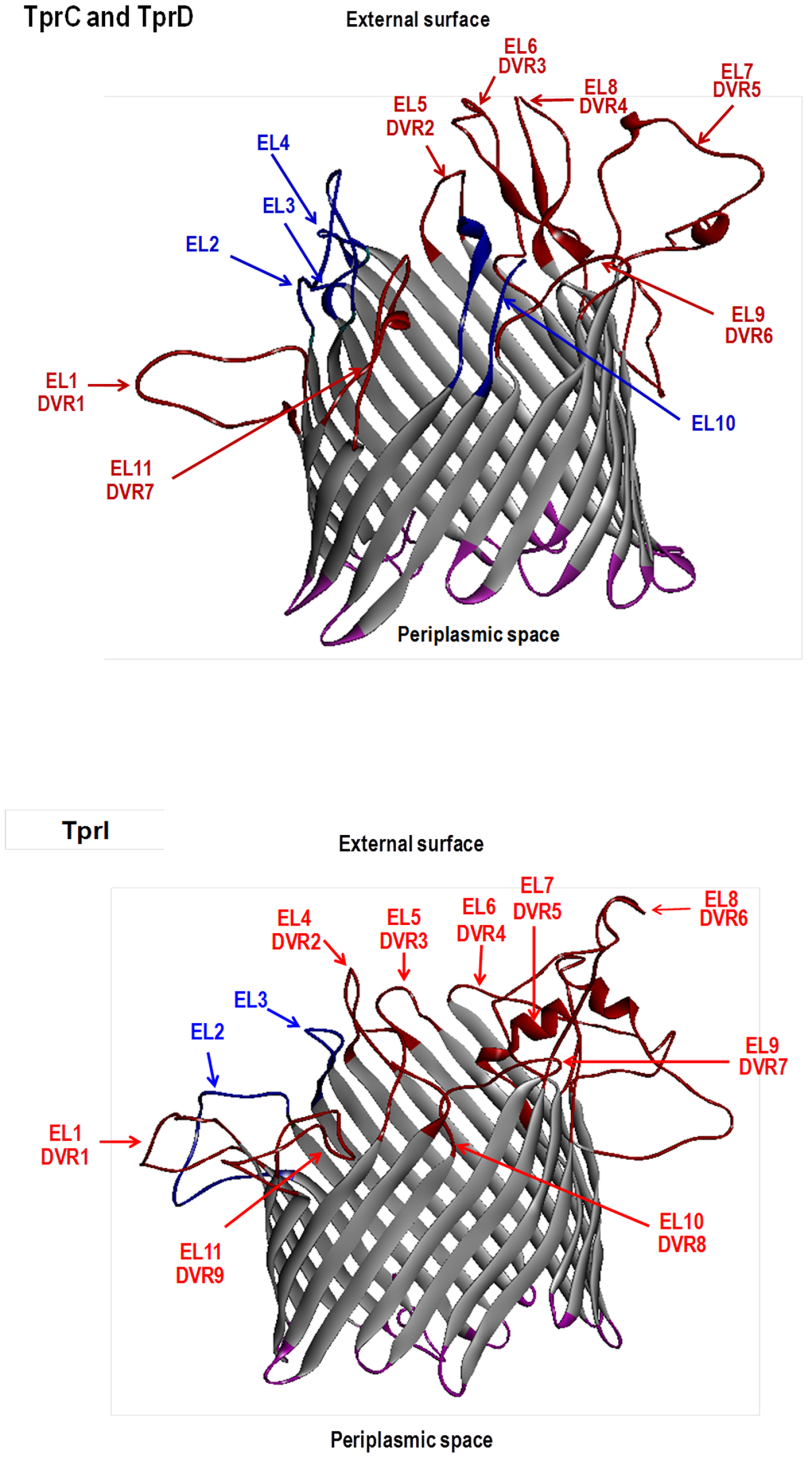
Structural models of TprC/D and TprI. Non-templated 3D models generated for the mature Nichols TprC/D and Nichols TprI peptides using the TMBpro algorithm [61] suggest a typical β-barrel structure. DVR,discrete variable regions. EL, external loops. Variable regions, DVR1–DVR7 for TprC/D and DVR1–DVR9 for TprI, as defined by protein sequence alignments (Figure S1 and S2) are indicated by red color (loops, font and arrows). Note that each DVR co-localizes with a predicted EL. Orientation of the structure was determined as specified by Randall et al [61]. Proposed conserved and variable surface exposed loops are highlighted in blue and red, respectively, and proposed periplasmic exposed regions of the proteins are in purple.

In both *tprC* and *tprD*, sequence variation does not occur randomly, but rather is found in discrete variable regions (DVRs; Supplemental Figures 2.1.and 2.2 in Figure S2). In the majority of cases, these base pair changes result in amino acid changes. Pore-forming activities for TprC/D have been recently reported by Anand et al. [68]. 3D predictions of peptides without signal peptides suggest typical β-barrel structures of 22 antiparallel transmembrane regions resulting in 11 loops at each end of the structure (Figure 3, top panel). Our analysis of 22 TprC/D sequences demonstrated seven DVRs, all of which co-localize with surface-exposed external loops predicted by the 3D models (Figure 3, and Supplemental Figure 2.1 and 2.2 in Figure S2). In addition, these 3D predictions suggest four external loops with conserved sequences, located primarily in the amino-half of the proteins. This sequence variation in predicted surface-exposed peptide loops could have significant implications for cross-immunity.

### SUBFAMILY I: *tpr F and I* loci and their encoded proteins

In the Nichols genome, *tprF* and *tprI* loci are 1107 and 1827 nucleotides long, respectively. Their sequences are identical except that *tprF* is a truncated version of *tprI* due to a 720 nucleotide deletion (spanning the central and most of the 3′ region) in *tprF*, resulting in a shorter ORF, frameshifting and a premature termination [27]. In *T. p. pallidum* strains, *tprF* genes are identical in all isolates sequenced to date (Figure 1 and Supplemental Figures 1 and 2.3 in Figure S1 and S2). In contrast to the syphilis strains, the *pertenue* and Fribourg-Blanc isolates have a full length (not frameshifted) duplicated *tprI*-like gene at the *tprF* locus. Interestingly, however, the *tprF* locus is deleted in the *endemicum* strains Iraq B and Bosnia A, and in *T. paraluiscuniculi*. *tprI* loci are virtually identical to each other in *T. p. pallidum* strains except for the presence of a few synonymous SNPs in the 5′ and central regions reported in Street 14 [28]. In contrast, however, *tprF* or *tprI* ORFs are absent in the rabbit pathogen *T. paraluiscuniculi*. [36].

For a more detailed analysis of the polymorphism observed in the *tprF* and *tprI* loci, a sequence alignment was generated including all genuine (not truncated or replaced) *tprF* and *tprI* loci from 11 strains (Supplemental Figure 2.3 in Figure S2). TprF and TprI in syphilis and non-syphilis organisms display DVR patterns resembling the heterogeneity observed in TprC and TprD above, though to a lesser extent (Supplemental Figure 2.3 in Figure S2). Changes are clustered in 9 DVRs spread throughout the protein sequences. Deduced TprF and TprI proteins are also predicted to be outer membrane proteins [33], [40], [41], [68]. Structural predictions also suggest that TprF and TprI are homologs of transport porins with OM localization, and 3D predictions of TprF/TprI peptides without signal peptides (Figure 3, bottom panel) yield typical β-barrel structures. Similar to TprC/D, all TprF/I DVRs show co-localization with predicted surface exposed loops (Figure 3 bottom panel, and Supplemental Figure 2.3 in Figure S2), again suggesting an important role for these variable regions during infection.

### SUBFAMILY II: *tprE*, *G* and *J* loci and their encoded proteins

The Subfamily II genes include *tpr E*, *G*, and *J*, which code for proteins nearly 800 amino acids in length with highly conserved amino termini, unique central regions and carboxyl ends with small unique gene-specific signatures [33]. *tprE* shows very limited sequence variation among strains and subspecies, however, the observed changes clearly segregate syphilis from non-syphilis treponemes and the Fribourg-Blanc strain (Supplemental Figure 1.4 in Figure S1). *T. paraluiscuniculi* has a *tprGJ* chimera (predicted truncation) in the *tprE* locus [36].

**Figure 4:**
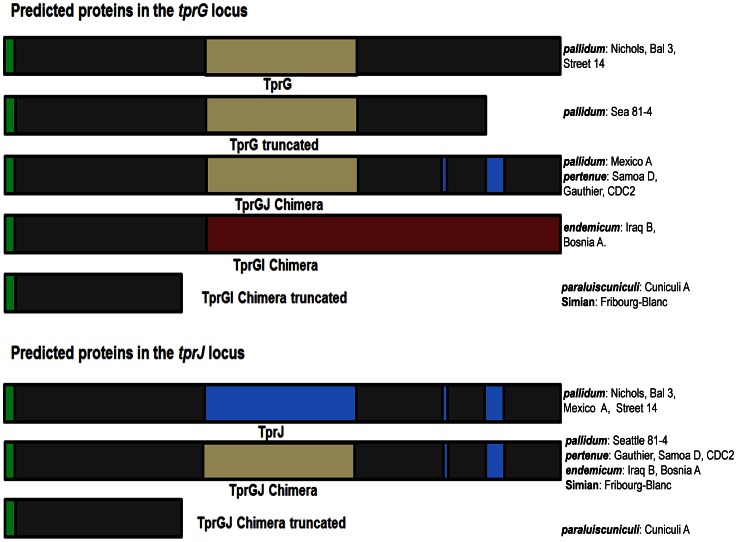
*tprG* and *tprJ* loci. Top: proteins encoded in the tprG locus. Bottom, proteins encoded in the tprJ locus. Regions of the same color indicate sequence identity among gene products. Five different variants can be identified among the 12 strains analyzed in the tprG locus. Nichols, Bal3 and Street 14 encode the Nichols reference TprG. Sea81-4encodes a truncated TprG. The TprGJ chimera is found in Mexico A, Samoa D, Gauthier and CDC2; the TprGI chimera is found in Iraq Band Bosnia A, and a truncated TprGI chimera is found in Cuniculi A and Fribourg-Blanc. At the tprJ locus, Nichols, Bal3, Mexico and Street 14 contain the Nichols TprJ. Sea81-4 and all non-syphilis strains carry theTprGJ hybrid. Green, signal peptide.

In contrast, the *tprG* locus is more diverse in its gene sequence, in that five different groups of ORFs can be found (Figure 1, Figure 4 and Supplemental Figure 1.6.1 and 1.6.2 in Figure S1): 1) *tprG* sequences as described in the Nichols genome (Bal 3, Street 14); 2) a truncated *tprG* due to two single and one 3-nucleotide insertions (position range 1885–1956), frameshifting, and a premature stop at its 3′ end (Sea81-4); 3) a *tprGJ* chimera, in which the 3′ end of *tprG* has been replaced by the corresponding region of *tprJ* as evidenced by the presence of a *tprJ*-specific signature (TAACGGGAACCCTCTCCCTTCCGGCGGTTCCTCAGGGCACATTGGCCT) near the 3′ end of *tprJ* (Mexico A and all *T. p. pertenue* strains); 4) a *tprGI* chimera in which the 5′ end of the ORF is homologous to the corresponding region of *tprG*, and its central and 3′ regions of the gene are homologous to the corresponding regions of *tprI* (all *T. p. endemicum* strains); and 5) a truncated *tprGI* chimera due to a single nucleotide insertion (*T. paraluiscuniculi* and the Fribourg-Blanc strain).

While four *T. p. pallidum* strains have the reference Nichols *tprJ* sequence, the *T. p. pallidum* Sea 81-4 strain and all non-*pallidum* treponemes studied to date contain a *tprGJ* chimera in the *tprJ* locus (Figure 1 and Figure 4). The rabbit pathogen, however, contains a *tprGJ* chimera that codes for a truncated protein due to an insertion in its 5′ end [36].

### SUBFAMILY III: *tprA*, *B*, *H*, and *L* loci

Subfamily III *tprs* show a reduced degree of homology among family members, compared to Subfamilies I and II, with only small regions of sequence identity scattered throughout the coding sequences [27], [33]. This is contrasted by a lower level of sequence heterogeneity at each locus among strains, subspecies, and species. *tprB* shows no variation among all strains (Supplemental Figure 1.2 in Figure S1). Among strains and subspecies, the *tprH* locus also contains highly homologous sequences, with only a few point mutations, of which 3 SNPs consistently distinguish syphilis vs. non-syphilis organisms (Supplemental Figure 1.7 in Figure S1).

**Figure 5:**
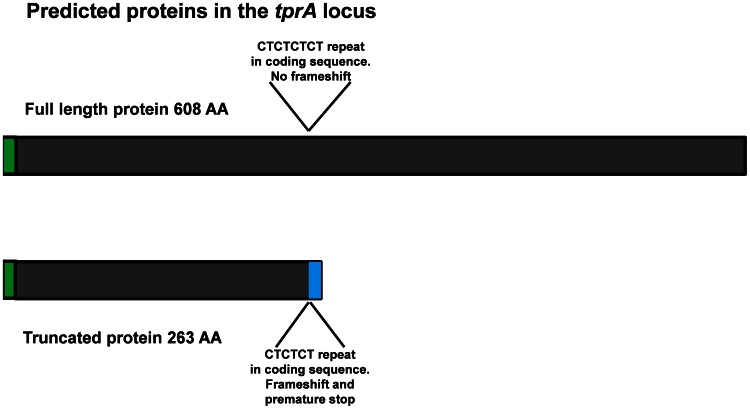
Encoded variants at the *tprA* locus. The Nichols, Bal3, Mexico A, and Street 14 isolates carry a gene encoding a truncated protein as result of the presence of only 3 CT dinucleotide repeats. A gene coding for a full length protein, which contain 4 CT dinucleotide repeats, is found in Sea81-4, Gauthier, Samoa D, CDC2, Iraq B, Bosnia A, Fribourg-Blanc and Cuniculi A. Blue color, unique sequence as result of frameshifting. Green, signal peptide.

In the *tprA* locus, at positions 706 to 711, there is a short region containing either three or four CT dinucleotide repeats. Strains containing only three CT repeats carry a gene that codes for a truncated protein due to a frameshift leading to a premature stop (Nichols, Mexico A, Street 14 and Bal 3). In contrast, strains carrying *tprA* genes with four CT repeats (the syphilis Sea 81-4 and all non-syphilis isolates) have no predicted frameshift and generate a sequence encoding a full length TprA product (Figure 5).

**Figure 6:**
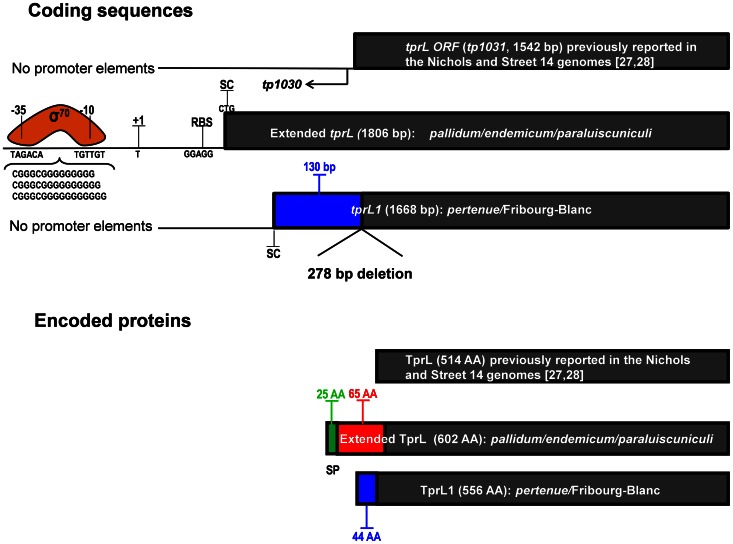
Encoded variants at the *tprL* (tp1031) locus. Coding sequences: Three different coding sequences have been identified for treponemal species and subspecies: the proposed *tprL* ORFs in the Nichols and Street 14 genome sequences; an extended *tprL* for *pallidum*, *endemicum*, and *paraluiscuniculi* strains; and a fused *tprL* (called *tprL1*) for *pertenue* and the Fribourg-Blanc strains. The Nichols ORF was predicted to be 1542 bp, although lacks identifiable promoter elements upstream. In this study, an extended *tprL* of 1806 bp has been identified in the Nichols and other *pallidum* strains, as well as in *endemicum* and *paraluiscuniculi* strains. The initially shorter Nichols tprL was the result of sequencing errors in the reported Nichols genome sequence [27]. Typical promoter elements are shown for the extended *tprL* ORF (SC, start codon. RBS, ribosomal binding site. +1, transcriptional start site (TSS). −10 and −35, σ^70^ signatures). A deletion of 278 bp (274 bp of the 5′ end of *tp1030*, whose coding sequence is located on the minus strand, and 4 bp of the 5′ end of the genome-derived *tprL*) creates an alternative start site in *tp1030* for *pertenue* and Fribourg-Blanc *tprL1*, resulting in a shorter ORF of 1668 base pairs. This ORF, however, lacks recognizable promoter elements. Encoded proteins: Differences in coding sequences result in two different proteins: 1) a shorter *pertenue*/Fribourg-Blanc variant with a 44 amino acid unique amino terminus and 2) a longer TprL in the remaining species/subspecies with a predicted signal peptide 25 amino acids long (green) in the longer product, but not identifiable in the *pertenue*/Fribourg-Blanc gene product. Blue color, region unique to *pertenue* and Fribourg-Blanc strains (132 nucleotides or 44 amino acids). Red color, region unique to the *pallidum*, *endemicum* and *paraluiscuniculi* species/subspecies (65 amino acids).

*tprL* (*tp1031*) shows major changes among strains and subspecies. Re-analysis of this region in the all of the *endemicum* and *pallidum* strains and 8 additional syphilis strains (Brinck Reid et al., unpublished) revealed a larger putative *tprL* ORF coding for a protein sequence of 602 amino acids, compared to 514 amino acids as previously reported for the Nichols and Street 14 strains [27], [28]. In this extended ORF (Figure 6), an alternative start codon (CTG) was identified with a typical ribosomal binding site (RBS, GGAGG). Furthermore, beginning at position −31, a 15 to 17 nucleotide poly-G tract flanked by −10 and −35 σ^70^ signatures (TAGACA and TGTTGT) is evident (Figure 6). Unlike the TprL product annotated in the Nichols genome sequence, the extended TprL is predicted to have a putative OM localization, with a predicted cleavable signal peptide (cleavage between positions 25 and 26, VFS-EQ). Compared to *T. p. pallidum and T. p. endemicum* sequences, our analysis revealed a gene fusion in the *T. p. pertenue* and Fribourg-Blanc strains caused by a deletion of 278 nucleotides (Figure 6), encompassing the 5′ end and central regions of the *tp1030* ORF and a small fragment of the 5′ end of *tprL* including its start codon. This deletion creates a hybrid sequence (*tp1030 and tprL*, here called *tprL1*) of 1668 bp with the start codon (ATG) in the plus strand of *tp1030* (the *tp1030* coding sequence is located on the minus strand of the chromosome) in frame with the rest of *tprL* (*tp1031*). As a consequence, the first 130 nucleotides of this new *pertenue tprL1* (Figure 1, Figure 6 and Supplemental Figure 1.9 in Figure S1) are unique, not found in *T. p. pallidum* or *endemicum tprL*. The new extended TprL (in *T. p. pallidum* and *T. p. endemicum* and *T. paraluiscuniculi*) and the newly predicted TprL1 proteins (*T. p. pertenue* and the Fribourg-Blanc treponeme) are 602 and 556 amino acids long, respectively. Because the first 44 amino acids of TprL1 are encoded by the plus strand, this region is unique to the yaws and simian strains, with no homologous peptide in the *pallidum* and *endemicum* proteins (Supplemental Fig. 1.9 Figure S1). This unique peptide sequence is also not found elsewhere in the chromosome. Unlike the newly predicted extended TprL, TprL1 does not have a predicted signal peptide (Figure 6). This raises the possibility that the *pallidum* and *endemicum* subspecies may have an OM-localized TprL, while this would be predicted to be absent in the *pertenue* subspecies.

## Discussion

The 12 treponemal isolates from the three *T. pallidum* subspecies (*pallidum*, *pertenue* and *endemicum*), the Fribourg-Blanc treponeme, and *T. paraluiscuniculi* show pleomorphic genetic changes in the *tpr* family characterized by SNPs, indels, chimeric sequences, and even absence of entire ORFs. Initial comparisons of the currently available full genome sequences of the Nichols, Chicago C, Sea81-4 and Street 14 syphilis strains revealed a high degree of sequence identity and a remarkable conservation of their genome organization [30] (and Giacani et al., unpublished). The study by Mikalova et al. [29] confirmed these observations, reporting clustering of sequence divergence in only a handful of distinct genomic regions among syphilis and non- syphilis strains, similar to those identified previously by Weinstock and colleagues [69]. Many of the hot spots of diversity are located in genes encoding members of the Tpr antigen family. The present study, however, provides a detailed description of sequence diversity within this paralog family and uncovers a rich number of sequence modifications among species, subspecies and strains. Importantly, our analyses also indicate some alternative genes or modified loci.

It is striking that much of the sequence diversity identified in the *tpr* genes segregates the strains into the same subspecies and species groups that were originally defined according to their modes of transmission, their natural hosts, and the diseases they cause. This is most effectively seen in the colored blocks in Figure 1. Given that the *tpr* loci represent the primary regions comprising the extremely low genomic diversity among the *T. pallidum* subspecies, it is likely that the proteins encoded by these variant genes play a major role in the differing pathogenesis of syphilis vs. yaws vs. endemic syphilis. Assigning a definitive role for individual proteins or combinations of proteins in determining clinical outcomes, however, awaits the determination of the functions of the Tpr proteins and the ability to genetically manipulate these genes within the organism. To inform studies of possible location and function, computational and immunological studies can provide clues for individual gene products.

Several arguments emphasize a key role for TprC and TprD during syphilis infection: 1) they are the targets of strong antibody and cellular immune responses [35], [37], [40], [56]; 2) immunization with recombinant TprC/D induces partial protection against infectious challenge [37]; 3) their surface exposure is supported by opsonophagocytosis assays [68] (Lukehart et al., unpublished); 4) TprC and D show sequence diversity among strains [37] (and this study); and 5) 3D models predict a typical β-barrel structure with surface-exposed loops that contain each of the regions where sequence diversity is localized (this study). It is highly unlikely that the co-location of sequence diversity and predicted surface-exposed loops is coincidental. A recent study by Anand et al. [68] proposes an alternative model for TprC and TprF, suggesting that the amino terminus of these two proteins is localized in the periplasmic space. However, experimental evidence argues against this model. Recombinant amino terminal TprF/I peptide induces partial protection against homologous challenge in immunization experiments in the rabbit model [37] and elicits opsonizing antibodies upon immunization (Lukehart et al., unpublished), observations supportive of surface exposure. However, the TprC and D sequence diversity (localized in the exposed DVR) identified among subspecies in the present study may contribute to the variable degree of cross-protection observed among *T. pallidum* strains and subspecies in infection-induced immunity. In this context, it is possible that sequence differences in the DVRs of TprC and D could lead to subspecies- or strain-specific surface-exposed epitopes that are critical to opsonic function or other mechanisms of protection. Studies are ongoing to test this hypothesis. A recognized example of functionally important strain-specific epitopes is loop 5 of the OMP P2 protein of non-typeable *Haemophilus influenzae*, which is associated with elicitation of bactericidal antibodies and protective immunity [70]. An alternative, or complementary, function of variable surface-exposed loops (e.g. DVR) could be that of providing steric hindrance to prevent the immune system from recognizing conserved external loops on the antigen, which are perhaps essential for correct protein structure or function. It is noteworthy that TprC and TprD are each predicted by 3D analysis to contain 4 conserved external loops.

During natural human infection and experimental infection of rabbits [37], [56], antibodies are made against TprC/D and TprD2. In addition to TprC and D, the TprD2 variant is also predicted to have surface exposure [37], [40], [56], and is found in both syphilis and non-syphilis treponemes (Figure 1). The regions unique to TprD2 also contain predicted external loops, thus adding another layer of complexity to the already existing set of predicted loops for TprC and D (not shown). Our structural predictions of TprC/D showing co-localization of external loops with DVRs is strong support for our hypothesis that antigenic differences in surface exposed loops of TprC and D have functional significance in immunity to the *T. pallidum* subspecies, and may be determinants of cross-immunity among subspecies and strains.

Of interest is the observation that the CDC2 strain maintained in Seattle (originally obtained in 2005 from Rob George and Victoria Pope from the Centers for Disease Control in Atlanta, GA) contains a *tprD*-like allele while the corresponding sequence reported by Mikalova et al. [29] contains a *tprD*2 sequence. Re-sequencing of the *tprD* locus of this strain using our original frozen stocks confirmed that the CDC2 strain indeed contains a *tprD*-like allele. Also, we have sequenced the *tprD* locus of the *pertenue* CDC1 strain, isolated in a neighboring village in Africa from where the CDC2 strain was obtained, and found that the CDC1 strain also contains a *tprD*-like gene. It may demand a significant effort to identify the source of discrepancy between our data and that of Mikalova et al., perhaps requiring the analysis of the two CDC2 lineages over the last several years.

In contrast to syphilis treponemes, the *tprF* and *I* loci in *T. p. pertenue* and the Fribourg-Blanc treponemes each contain identical full-length ORFs. Although their coding sequences are identical within each location, *tprF* and *tprI* are located in separate *tprG-F* and *tprJ-I* operons, respectively, and their expression may be differentially modulated. The number of G residues in a polyG string in their promoters controls phase variation of these operons [46], and the binding of TpCRP (Tp0262) to the promoters was shown to either increase (*tprJ*) or decrease (*tprG*) transcription of the operon [71]. The implications of a “double dose” of *tprI* in the non-*pallidum* strains might be reflected in the total amount of message made in tissue specific locations or in differential expression over time during infection. Preliminary studies of antibody reactivity in rabbits infected with *T. p. pertenue* Gauthier strain demonstrate high levels of antibody to TprI, consistent with high (or double) expression of the protein (Lukehart et al., unpublished). The strong resemblance of the TprI/F 3D predictions to the TprC/D structural models, and the co-localization of DVRs and external loops suggest analogous roles at the microbe-host interface.

*T. pallidum tprGI* chimeras were identified by Giacani et al. [36] in *T. paraluiscuniculi* and also present in the whole genome sequences later reported by Strouhal et al. [31] and Smajs et al. [32], whose unique sequence composition was also recognized by these authors. Our analysis shows that, in all strains of *T. p. pertenue*, *T. p. endemicum* and the Fribourg-Blanc treponeme, the *G* and *J* loci are occupied by either *tprGJ* or *tprGI* chimeric genes. In contrast, the Nichols reference *tprG* and *tprJ* genes are frequently found in syphilis isolates, but not in any *pertenue*, *endemicum* or the Fribourg-Blanc strains tested to date. Only the *T. p. pallidum* Mexico A and Seattle 81-4 strains carry the *GJ* chimeric gene in the *tprG* and *tprJ* loci, respectively. Of interest is the presence of three truncated chimeras encoded by the *tpr E*, *G*, and *J* loci in *T. paraluiscuniculi*. This, in addition to predicted truncations or absences of Subfamily I Tprs (Figure 1), is perhaps related to the inability of *T. paraluiscuniculi* to infect humans, although further study is needed to explore this issue more thoroughly.

One might wonder whether the *tpr* chimeras identified in this study are artifactual, due to “jumping” between highly similar sequences during PCR amplification [72]–[74]. In our study, *tpr* chimeras are unlikely to be artifacts for two reasons: 1) independent PCR amplifications of treponemal DNA obtained from different strain harvests rendered identical sequences, and 2) published sequences obtained by multiple sequencing approaches also show the same chimeras [31], [32], [36], [75].

With the exception of TprK, little is known about the other members of Subfamily III Tprs (*tprA*, *tprB*, *tprH*, and *tprL*). TprA, B and L are predicted to be OMPs [40], [41], and Tpr B induces antibodies that promote opsonophagocytosis (Lukehart et al., unpublished). Sequence conservation of *tprB* and *tprH* across species, subspecies, and strains suggests a required function for these proteins in the biology of *T. pallidum*. Nucleotide repeats, whether in regulatory or coding regions, are frequently associated with modulation of gene expression in an ON-OFF manner. The structure of the promoter region of the newly proposed extended *tprL* ORF is highly reminiscent of modulation of gene expression by single nucleotide repeats in the promoters of *porA* and *opc* loci of *Neisseria meningitidis* [76]–[78]. One could argue that predictions of an extended *tprL* ORF may lack accuracy because of the assumption of CTG as start codon, an underrepresented start codon in the annotated Nichols *T. pallidum* genome. However, our predictions are supported by the identification of a typical RBS, as well as −10 and −35 σ^70^ signatures with intervening homopolymeric G repeats of variable lengths resembling classic bacterial phase variation systems. In *tprA*, the variable number of CT dinucleotide repeats creates frameshifting and premature termination, dividing strains carrying *tprA* genes coding for full length product from those encoding predicted truncated products (Figure 5 and Supplemental Figure 1.1 in Figure S1). This is another mechanism for possible phase variation.

Our analysis of the *tpr* gene sequences is based on an approach of targeted PCR amplification, cloning, and sequencing a number of clones to obtain consensus sequences. The *tpr* ORF sequences appear to be unchanging within a given strain during infection. However, limited information at the population level invites speculation about the possible presence of genetically distinct subpopulations within isolates. Smajs et al. [28], [79] reported that at least two subpopulations are present within the Nichols strain as defined by a ∼1 Kb deletion in the flanking region of *tp0131*. Our approach could have overlooked underrepresented variant organisms within isolates and, if intrastrain variation indeed exists, our findings might then reflect amplification of the most predominant subpopulation. Small mutational changes, even SNPs, in coding or non-coding regions can affect transcription, translation, or folding of the protein themselves, of neighboring genes, or those at more distant sites [80]–[83]. This could explain, for example, some of the differences in transcription observed among treponemal strains [40]. On the other hand, the now standard use of template-based assembly of short stretches of sequence generated by newer sequencing technologies can overlook the existence of hybrid genes or missing ORFs, whereas our individual-ORF sequencing approach can clearly identify these variations. Renewed efforts to address all of the above questions may be effectively resolved using next generation approaches such as deep sequencing of targeted regions, single cell isolation, or whole transcriptome sequencing.

How might knowledge of *tpr* sequence diversity be translated into tools that are relevant to persons who are infected with one of the pathogenic treponemes? The geographical distribution of yaws and syphilis is not as distinct as decades ago, and travel or migration can serve to transport an infection between urban and rural settings, complicating diagnosis. Because of the re-emergence of yaws over the past 20 years [84], etiological differentiation of yaws vs. syphilis infections is desirable, and a practical approach for diagnosis is needed. The overall reported genetic variability between syphilis and yaws treponemes (0.2%) makes these organisms almost genetically indistinguishable, and existing serological tests fail to differentiate the infections. Several small signatures that differentiate the distinct species/subspecies have already been identified in several genes [47]–[49], [51], [52], [85]. The unique sequence composition of TprL described here in *pertenue* vs. *pallidum* strains reveals a possible 90 amino acid sequence unique to non-yaws treponemes, which includes a 25 amino acid predicted signal peptide, as well as a 44 amino acid peptide specific to *T. p. pertenue*. Given that Giacani et al. [40] showed that the *tprL* ORF is actively transcribed in both syphilis and yaws treponemes during experimental infection, our findings could facilitate the development of targeted serological screening for differentiating these two infections.

Treponemal infections are chronic, yet only a minority of infected persons develops the severe late manifestations of disease. Is it possible that small genetic markers in the infecting could predict clinical outcome? We previously showed that rabbits infected intravenously with the Sea 81-4 strain had higher levels of cerebrospinal fluid (CSF) inflammation, compared to other infecting strains, while animals infected with Bal 7 had more severe skin disease [86]. Our more recent work in humans supports the hypothesis that disease outcome may be related to genetically defined strain types [55]. Subfamily II *tpr*s and the *arp* genes were first utilized for strain typing purposes by Pillay et al. [50], although they were not able to correlate strain type with clinical outcome. Using an enhanced strain typing system developed by Marra et. al. [55], which includes the targets initially described by Pillay et. al. [50] and the *tp0548* gene, we demonstrated that patients infected by 14d/f type strains were significantly more likely to have neurosyphilis [55]. Four of the *pallidum* strains shown to represent different genotypes in this report (Nichols, Street 14, Mexico A and Sea 81-4) fall into four different molecular types using the enhanced typing system. The correlation supports the possibility that sequence changes in the *tpr* genes may be related to specific disease manifestations.

It is noteworthy that *T. paraluiscuniculi* causes a very mild infection in its natural host, compared to syphilis, and is unable to infect humans [2], [12], [87]. One possible explanation for mild natural infection and the failure to infect other hosts is the dearth of functional Tpr proteins in this organism: there are seven truncated Tpr proteins (TprC, D, F, I, E, G and J) in *T. paraluiscuniculi*. In contrast, all *T. pallidum* subspecies and the Fribourg-Blanc treponemes, which have fuller Tpr repertoires, can multiply in more than one vertebrate host and can cause infection in humans. The Fribourg-Blanc treponeme, isolated from non-human primates from a yaws-endemic region in Africa [3], [4], resembles very closely the *tpr* repertoire of yaws strains (10 out of 12 ORFs are of the same type), although it resembles *T. p. endemicum* at the *G* locus, implying shared evolutionary pathways, as previously proposed [29], [65], [88], as well as common strategies of interaction between microbes and their host.

Although the clinical outcome of infection is likely dependent upon several factors, including individual host immunity, inoculum size, and route of infection, sequence changes in the *tpr* genes could determine differences in antigenicity or function, resulting in different adaptive strategies and differences in pathogenicity. While the distribution of *tpr* gene variants among the 12 isolates studied here appears, in most cases, to be clustered by subspecies, some isolates in the *T. p. pallidum* group share *tpr* variants that are otherwise restricted to non-syphilis organisms. For example, Sea 81-4 contains four *tpr* ORFs present in the *endemicum* subgroups (Figure 1), and Mexico A contains the *tprGJ* chimera in the *tprG* locus. The recent demonstration of syphilis-like genital lesions and purported sexual transmission of a yaws-like treponeme in wild baboons [13] suggests that pathogenicity and mode of transmission may not, however, be completely hard-wired in the genome. The sharing of some *tpr* variants among individual *pallidum* strains and the non-*pallidum* strains confounds the concept of a purely genetic basis for the nature of the disease. These findings again raise the 1960's nature vs. nurture controversy between Hudson and Hackett with regard to the biological or environmental/epidemiological basis for the differing clinical manifestations seen among the treponematoses [89], [90]. Based upon *tpr* sequencing, there is genetic heterogeneity (five genogroups) within the *pallidum* subspecies, as well as some overlap among subspecies and species. Rather than having discrete organisms for each treponemal disease, there may in fact be a genetic continuum of the pathogenic Treponema, individual components of which affect pathogenesis in an individual host in concert with social or environmental factors that influence routes of transmission and disease manifestations. Finding the answer to this question will depend upon the ability to genetically manipulate *T. pallidum* so that the effects of individual genes can be definitively assessed.

## Supporting Information

Figure S1 **Predicted full length amino acid and DNA sequence alignments of the** ***tpr*** **gene family by locus.** For the ORFs containing indels resulting in frameshifts and truncated proteins, only the encoded amino acid sequences before the premature stop codon are shown. In *tprD* locus, *tprD2* alleles were excluded for clarity purposes only. In *tprG* locus, because of the significant dissimilarities among sequences, alignments are separated in two groups: *GI* and *GJ* chimeras. Red font, *T. p. pallidum* subspecies; blue *T. p. pertenue*; bright green, *T. p. endemicum*; yellow, the Simian treponeme; and pink, *T. paraluiscuniculi*.
(DOCX)

Figure S2 **Alignment of amino acid sequences of the predicted protein sequences encoded at the** ***tprD*** **(2.1),** ***tprC*** **(2.2), and** ***tprF*** **and** ***tprI*** **(2.3) loci.** The TprD2 truncated proteins encoded by the *tprC/D* loci in *T. paraluiscuniculi*, as well as by the *tprF* locus in *T. p. pallidum* strains are not included in the alignment for clarity purposes. Also, no *T. paraluiscuniculi tprF* and *tprI* ORFs are included because the *tprF* or *tprI* coding sequences are absent in the rabbit pathogen. DVR: Discrete variable regions. EL: External loops predicted by 3D models. SP: Predicted signal peptide. The last letter on the left column (strain name) indicates the locus where the predicted protein sequence is encoded. Red font, *T. p. pallidum* subspecies; blue *T. p. pertenue*; brilliant green, *T. p. endemicum*; and yellow, the Simian treponeme.
(DOCX)

Table S1 **Primers used for amplification (1) or sequencing (2).**
(DOCX)

Table S2 **GenBank accession numbers per locus and strain.**
(DOCX)

## References

[1] Turner TB, Hollander DH (1957) Biology of the Treponematoses. Geneva: World Health Organization. 13423342

[2] Graves S, Downes J (1981) Experimental infection of man with rabbit-virulent *Treponema paraluis- cuniculi* . Br J Vener Dis 57: 7–10. 7470837 10.1136/sti.57.1.7 PMC1045857

[3] Fribourg-Blanc A, Mollaret HH, Niel G (1966) [Serologic and microscopic confirmation of treponemosis in Guinea baboons]. Bull Soc Pathol Exot Filiales 59: 54–59. 5333741

[4] Fribourg-Blanc A, Mollaret HH (1969) Natural treponematosis of the African primate. Primates Med 3: 113–121. 5006024

[5] Smith JL, David NJ, Indgin S, Israel CW, Levine BM, et al (1971) Neuro-ophthalmological study of late yaws and pinta. II. The Caracas Project. Brit J Vener Dis 47: 226–251. 4936861 10.1136/sti.47.4.226 PMC1048203

[6] Gerbase AC, Rowley JT, Heymann DH, Berkely SF, Piot P (1998) Global prevalence and incidence estimates of selected curable STDs. Sex Transm Infect 74: S12–16. 10023347

[7] World Health Organization DoRHaR (2007) The global elimination of congenital syphilis: rationale and strategy for action. http://www.who.int/reproductivehealth/publications/rtis/9789241595858/en/

[8] Meheus A, Antal GM (1992) The endemic treponematoses: not yet eradicated. World Health Stat Q 45: 228–237. 1281363

[9] Antal GM, Lukehart SA, Meheus AZ (2002) The endemic treponematoses. Microbes Infect 4: 83–94. 11825779 10.1016/s1286-4579(01)01513-1

[10] Asiedu K, Amouzou B, Dhariwal A, Karam M, Lobo D, et al (2008) Yaws eradication: past efforts and future perspectives. Bull World Health Organ 86: 499–499A. 18670655 10.2471/BLT.08.055608 PMC2647478

[11] Roman GC, Roman LN (1986) Occurrence of congenital, cardiovascular, visceral, neurologic, and neuro-ophthalmologic complications in late yaws: a theme for future research. Rev Infect Dis 8: 760–770. 3538316

[12] DiGiacomo RF, Lukehart SA, Talburt CD, Baker-Zander SA, Condon J, et al (1984) Clinical course and treatment of venereal spirochaetosis in New Zealand white rabbits. Br J Vener Dis 60: 214–218. 6547627 10.1136/sti.60.4.214 PMC1046312

[13] Knauf S, Batamuzi EK, Mlengeya T, Kilewo M, Lejora IA, et al (2012) Treponema infection associated with genital ulceration in wild baboons. Vet Pathol 49: 292–303. 21411621 10.1177/0300985811402839

[14] Hovind-Hougen K (1983) Morpholgy. In: Schell RF, Musher DM, editors. Pathogenesis and Immunology of Treponemal Infection. New York, NY: Marcel Dekker, Inc.

[15] Hovind-Hougen K (1976) Determination by Means of Electron Microscopy of Morphological Criteria of Value for Classification of Some Spirochetes, in Particular Treponemes. Acta Path Microbiol Scan 255: 1–41. 58539

[16] Hovind-Hougen K, Birch-Andersen A, Jensen HJ (1976) Ultrastructure of cells of *Treponema pertenue* obtained from experimentally infected hamsters. Acta Pathol Microbiol Scand [B] 84: 101–108. 10.1111/j.1699-0463.1976.tb01909.x 775884

[17] Ovcinnikov NM, Delektorskij VV (1970) *Treponema pertenue* under the electron microscope. Br J Vener Dis 46: 349–379. 4920477 10.1136/sti.46.5.349 PMC1048099

[18] Sepetjian M, Guerraz FT, Salussola D, Thivolet J, Monier JC (1969) Contribution a l'etude du treponeme isole du singe par A. Bull World Health Organ 40: 141–151. 4978933 PMC2554448

[19] Thornburg RW, Baseman JB (1983) Comparison of major protein antigens and protein profiles of *Treponema pallidum* and *Treponema pertenue* . Infect Immun 42: 623–627. 6358027 10.1128/iai.42.2.623-627.1983 PMC264474

[20] Baker-Zander SA, Lukehart SA (1983) Molecular basis of immunological cross-reactivity between *Treponema pallidum* and *Treponema pertenue* . Infect Immun 42: 634–638. 6358028 10.1128/iai.42.2.634-638.1983 PMC264476

[21] Baker-Zander SA, Lukehart SA (1984) Antigenic cross-reactivity between *Treponema pallidum* and other pathogenic members of the family Spirochaetaceae. Infect Immun 46: 116–121. 6384040 10.1128/iai.46.1.116-121.1984 PMC261430

[22] Martin PM, Cockayne A, Georges AJ, Penn CW (1990) Immune response to *Treponema pertenue* and *Treponema pallidum* Nichols in patients with yaws. Res Microbiol 141: 181–186. 1693218 10.1016/0923-2508(90)90027-n

[23] Noordhoek GT, Cockayne A, Schouls LM, Meleon RH, Stolz E, et al (1990) A new attempt to distinguish serologically the subspecies of *Treponema pallidum* causing syphilis and yaws. J Clin Microbiol 28: 1600–1607. 2199521 10.1128/jcm.28.7.1600-1607.1990 PMC267996

[24] Magnuson HJ, Rosenau BJ (1948) The rate of development and degree of acquired immunity in experimental syphilis. Am J Syph Gonorrhea Vener Dis 32: 418–436. 18876783

[25] Magnuson HJ, Thomas EW, Olansky S, Kaplan BI, DeMello L, et al (1956) Inoculation syphilis in human volunteers. Medicine 35: 33–82. 13296652 10.1097/00005792-195602000-00002

[26] Wines BD, Ramsland PA, Trist HM, Gardam S, Brink R, et al (2011) Interaction of human, rat, and mouse immunoglobulin A (IgA) with Staphylococcal superantigen-like 7 (SSL7) decoy protein and leukocyte IgA receptor. J Biol Chem 286: 33118–33124. 21784854 10.1074/jbc.M111.272252 PMC3190891

[27] Fraser CM, Norris SJ, Weinstock GM, White O, Sutton GG, et al (1998) Complete genome sequence of *Treponema pallidum*, the syphilis spirochete. Science 281: 375–388. 9665876 10.1126/science.281.5375.375

[28] Matejkova P, Strouhal M, Smajs D, Norris SJ, Palzkill T, et al (2008) Complete genome sequence of *Treponema pallidum* ssp. *pallidum* strain SS14 determined with oligonucleotide arrays. BMC Microbiol 8: 76. 18482458 10.1186/1471-2180-8-76 PMC2408589

[29] Mikalova L, Strouhal M, Cejkova D, Zobanikova M, Pospisilova P, et al (2010) Genome analysis of *Treponema pallidum* subsp. *pallidum* and subsp. pertenue strains: most of the genetic differences are localized in six regions. PLoS One 5: e15713. 21209953 10.1371/journal.pone.0015713 PMC3012094

[30] Giacani L, Chattopadhyay S, Centurion-Lara A, Jeffrey BM, Le HT, et al (2012) Footprint of Positive Selection in *Treponema pallidum* subsp. *pallidum* Genome Sequences Suggests Adaptive Microevolution of the Syphilis Pathogen. PLoS Negl Trop Dis 6: e1698. 22720110 10.1371/journal.pntd.0001698 PMC3373638

[31] Strouhal M, Smajs D, Matejkova P, Sodergren E, Amin AG, et al (2007) Genome differences between *Treponema pallidum* subsp. *pallidum* strain Nichols and *T. paraluiscuniculi* strain Cuniculi A. Infect Immun 75: 5859–5866. 17893135 10.1128/IAI.00709-07 PMC2168363

[32] Smajs D, Zobanikova M, Strouhal M, Cejkova D, Dugan-Rocha S, et al (2011) Complete genome sequence of *Treponema paraluiscuniculi*, strain Cuniculi A: the loss of infectivity to humans is associated with genome decay. PLoS One 6: e20415. 21655244 10.1371/journal.pone.0020415 PMC3105029

[33] Centurion-Lara A, Castro C, Barrett L, Cameron C, Mostowfi M, et al (1999) *Treponema pallidum* major sheath protein homologue Tpr K is a target of opsonic antibody and the protective immune response. J Exp Med 189: 647–656. 9989979 10.1084/jem.189.4.647 PMC2192927

[34] Morgan CA, Molini BJ, Lukehart SA, Van Voorhis WC (2002) Segregation of B and T cell epitopes of *Treponema pallidum* repeat protein K to variable and conserved regions during experimental syphilis infection. J Immunol 169: 952–957. 12097401 10.4049/jimmunol.169.2.952

[35] Leader BT, Hevner K, Molini BJ, Barrett LK, Van Voorhis WC, et al (2003) Antibody responses elicited against the *Treponema pallidum* repeat proteins differ during infection with different isolates of *Treponema pallidum* subsp. *pallidum* . Infect Immun 71: 6054–6057. 14500529 10.1128/IAI.71.10.6054-6057.2003 PMC201100

[36] Giacani L, Sun ES, Hevner K, Molini BJ, Van Voorhis WC, et al (2004) Tpr homologs in *Treponema paraluiscuniculi* Cuniculi A strain. Infect Immun 72: 6561–6576. 15501788 10.1128/IAI.72.11.6561-6576.2004 PMC523035

[37] Sun ES, Molini BJ, Barrett LK, Centurion-Lara A, Lukehart SA, et al (2004) Subfamily I *Treponema pallidum* repeat protein family: sequence variation and immunity. Microbes Infect 6: 725–737. 15207819 10.1016/j.micinf.2004.04.001

[38] Giacani L, Hevner K, Centurion-Lara A (2005) Gene organization and transcriptional analysis of the *tprJ*, *tprI*, *tprG* and *tprF* loci in the Nichols and Sea 81-4 *Treponema pallidum* isolates. J Bacteriol 187: 6084–6093. 16109950 10.1128/JB.187.17.6084-6093.2005 PMC1196134

[39] Smajs D, McKevitt M, Howell JK, Norris SJ, Cai WW, et al (2005) Transcriptome of *Treponema pallidum*: Gene Expression Profile during Experimental Rabbit Infection. J Bacteriol 187: 1866–1874. 15716460 10.1128/JB.187.5.1866-1874.2005 PMC1063989

[40] Giacani L, Molini B, Godornes C, Barrett L, Van Voorhis WC, et al (2007) Quantitative analysis of *tpr* gene expression in *Treponema pallidum* isolates: differences among isolates and correlation with T-cell responsiveness in experimental syphilis. Infect Immun 75: 104–112. 17030565 10.1128/IAI.01124-06 PMC1828388

[41] Cox DL, Luthra A, Dunham-Ems S, Desrosiers DC, Salazar JC, et al (2010) Surface immunolabeling and consensus computational framework to identify candidate rare outer membrane proteins of *Treponema pallidum* . Infect Immun 78: 5178–5194. 20876295 10.1128/IAI.00834-10 PMC2981305

[42] Morgan CA, Lukehart SA, Van Voorhis WC (2002) Immunization with the N-terminal portion of *Treponema pallidum* repeat protein K attenuates syphilitic lesion development in the rabbit model. Infect Immun 70: 6811–6816. 12438357 10.1128/IAI.70.12.6811-6816.2002 PMC133068

[43] Morgan CA, Lukehart SA, Van Voorhis WC (2003) Protection against syphilis correlates with specificity of antibodies to the variable regions of *Treponema pallidum* repeat protein K. Infect Immun 71: 5605–5612. 14500480 10.1128/IAI.71.10.5605-5612.2003 PMC201104

[44] Giacani L, Sambri V, Marangoni A, Cavrini F, Storni E, et al (2005) Immunological evaluation and cellular location analysis of the TprI antigen of *Treponema pallidum* subsp. *pallidum* . Infect Immun 73: 3817–3822. 15908421 10.1128/IAI.73.6.3817-3822.2005 PMC1111852

[45] Giacani L, Molini BJ, Kim EY, Godornes BC, Leader BT, et al (2010) Antigenic variation in *Treponema pallidum*: TprK sequence diversity accumulates in response to immune pressure during experimental syphilis. J Immunol 184: 3822–3829. 20190145 10.4049/jimmunol.0902788 PMC3042355

[46] Giacani L, Lukehart S, Centurion-Lara A (2007) Length of guanosine homopolymeric repeats modulates promoter activity of Subfamily II *tpr* genes of *Treponema pallidum* ssp. *pallidum* . FEMS Immunol Med Microbiol 51: 289–301. 17683506 10.1111/j.1574-695X.2007.00303.x PMC3006228

[47] Noordhoek GT, Hermans PW, Paul AN, Schouls LM, van der Sluis JJ, et al (1989) *Treponema pallidum* subspecies *pallidum* (Nichols) and *Treponema pallidum* subspecies *pertenue* (CDC 2575) differ in at least one nucleotide: comparison of two homologous antigens. Microb Pathog 6: 29–42. 2471912 10.1016/0882-4010(89)90005-3

[48] Noordhoek GT, Wieles B, van der Sluis JJ, van Embden JD (1990) Polymerase chain reaction and synthetic DNA probes: a means of distinguishing the causative agents of syphilis and yaws? Infect Immun 58: 2011–2013. 2187816 10.1128/iai.58.6.2011-2013.1990 PMC258761

[49] Centurion-Lara A, Castro C, Castillo R, Shaffer JM, Van Voorhis WC, et al (1998) The flanking region sequences of the 15-kDa lipoprotein gene differentiate pathogenic treponemes. J Infect Dis 177: 1036–1040. 9534979 10.1086/515247

[50] Pillay A, Liu H, Chen CY, Holloway B, Sturm AW, et al (1998) Molecular subtyping of *Treponema pallidum* subspecies *pallidum* . Sex Transm Dis 25: 408–414. 9773432 10.1097/00007435-199809000-00004

[51] Cameron CE, Castro C, Lukehart SA, Van Voorhis WC (1999) Sequence conservation of glycerophosphodiester phosphodiesterase among *Treponema pallidum* strains. Infect Immun 67: 3168–3170. 10338539 10.1128/iai.67.6.3168-3170.1999 PMC96640

[52] Cameron CE, Lukehart SA, Castro C, Molini B, Godornes C, et al (2000) Opsonic potential, protective capacity, and sequence conservation of the *Treponema pallidum* subspecies *pallidum* Tp92. J Infect Dis 181: 1401–1413. 10762571 10.1086/315399

[53] Centurion-Lara A, Godornes C, Castro C, Van Voorhis WC, Lukehart SA (2000) The *tprK* gene is heterogeneous among *Treponema pallidum* strains and has multiple alleles. Infect Immun 68: 824–831. 10639452 10.1128/iai.68.2.824-831.2000 PMC97211

[54] Brinkman MB, McGill MA, Pettersson J, Rogers A, Matejkova P, et al (2008) A novel *Treponema pallidum* antigen, TP0136, is an outer membrane protein that binds human fibronectin. Infect Immun 76: 1848–1857. 18332212 10.1128/IAI.01424-07 PMC2346692

[55] Marra CM, Sahi SK, Tantalo LC, Godornes C, Reid T, et al (2010) Enhanced molecular typing of *Treponema pallidum*: geographical distribution of strain types and association with neurosyphilis. J Infect Dis 202: 1380–1388. 20868271 10.1086/656533 PMC3114648

[56] Centurion-Lara A, Sun ES, Barrett LK, Castro C, Lukehart SA, et al (2000) Multiple alleles of *Treponema pallidum* repeat gene D in *Treponema pallidum* isolates. J Bacteriol 182: 2332–2335. 10735882 10.1128/jb.182.8.2332-2335.2000 PMC111288

[57] Lukehart SA, Marra CM (2007) Isolation and laboratory maintenance of *Treponema pallidum* . Curr Protoc Microbiol Chapter 12: 7: 12A.11.11–12A.11.18. 10.1002/9780471729259.mc12a01s7 18770607

[58] Huang X, Madan A (1999) CAP3: A DNA sequence assembly program. Genome Res 9: 868–877. 10508846 10.1101/gr.9.9.868 PMC310812

[59] Katoh K, Kuma K, Toh H, Miyata T (2005) MAFFT version 5: improvement in accuracy of multiple sequence alignment. Nucleic Acids Res 33: 511–518. 15661851 10.1093/nar/gki198 PMC548345

[60] Ginalski K, Elofsson A, Fischer D, Rychlewski L (2003) 3D-Jury: a simple approach to improve protein structure predictions. Bioinformatics 19: 1015–1018. 12761065 10.1093/bioinformatics/btg124

[61] Randall A, Cheng J, Sweredoski M, Baldi P (2008) TMBpro: secondary structure, beta-contact and tertiary structure prediction of transmembrane beta-barrel proteins. Bioinformatics 24: 513–520. 18006547 10.1093/bioinformatics/btm548

[62] Hiller K, Grote A, Scheer M, Munch R, Jahn D (2004) PrediSi: prediction of signal peptides and their cleavage positions. Nucleic Acids Res 32: W375–379. 15215414 10.1093/nar/gkh378 PMC441516

[63] LaFond RE, Centurion-Lara A, Godornes C, Rompalo AM, Van Voorhis WC, et al (2003) Sequence diversity of *Treponema pallidum* subsp. *pallidum tprK* in human syphilis lesions and rabbit-propagated isolates. J Bacteriol 185: 6262–6268. 14563860 10.1128/JB.185.21.6262-6268.2003 PMC219401

[64] LaFond RE, Molini BJ, Van Voorhis WC, Lukehart SA (2006) Antigenic variation of TprK V regions abrogates specific antibody binding in syphilis. Infect Immun 74: 6244–6251. 16923793 10.1128/IAI.00827-06 PMC1695500

[65] Gray RR, Mulligan CJ, Molini BJ, Sun ES, Giacani L, et al (2006) Molecular evolution of the *tprC*, *D*, *I*, *K*, *G*, and *J* genes in the pathogenic genus Treponema. Mol Biol Evol 23: 2220–2233. 16926243 10.1093/molbev/msl092

[66] Giacani L, Brandt SL, Puray-Chavez M, Brinck Reid T, Godornes C, et al (2012) Comparative Investigation of the Genomic Regions Involved in Antigenic Variation of the TprK Antigen among Treponemal Species, Subspecies, and Strains. J Bacteriol 194: 4208–4225. 22661689 10.1128/JB.00863-12 PMC3416249

[67] Centurion-Lara A, LaFond RE, Hevner K, Godornes C, Molini BJ, et al (2004) Gene conversion: a mechanism for generation of heterogeneity in the *tprK* gene of *Treponema pallidum* during infection. Molecular Microbiology 52: 1579–1596. 15186410 10.1111/j.1365-2958.2004.04086.x

[68] Anand A, Luthra A, Dunham-Ems S, Caimano MJ, Karanian C, et al (2012) TprC/D (Tp0117/131), a trimeric, pore-forming rare outer membrane protein of *Treponema pallidum*, has a bipartite domain structure. J Bacteriol 194: 2321–2333. 22389487 10.1128/JB.00101-12 PMC3347077

[69] Weinstock GM, Hardham JM, McLeod MP, Sodergren EJ, Norris SJ (1998) The genome of *Treponema pallidum*: new light on the agent of syphilis. FEMS Microb Rev 22: 323–332. 10.1111/j.1574-6976.1998.tb00373.x 9862125

[70] Haase EM, Campagnari AA, Sarwar J, Shero M, Wirth M, et al (1991) Strain-specific and immunodominant surface epitopes of the P2 porin protein of nontypeable *Haemophilus influenzae* . Infect Immun 59: 1278–1284. 1706317 10.1128/iai.59.4.1278-1284.1991 PMC257839

[71] Giacani L, Godornes C, Puray-Chavez M, Guerra-Giraldez C, Tompa M, et al (2009) TP0262 is a modulator of promoter activity of tpr Subfamily II genes of *Treponema pallidum* ssp. *pallidum* . Mol Microbiol 72: 1087–1099. 19432808 10.1111/j.1365-2958.2009.06712.x PMC2698047

[72] Wang GC, Wang Y (1996) The frequency of chimeric molecules as a consequence of PCR co-amplification of 16S rRNA genes from different bacterial species. Microbiology 142 Pt 5: 1107–1114. 8704952 10.1099/13500872-142-5-1107

[73] Wang GC, Wang Y (1997) Frequency of formation of chimeric molecules as a consequence of PCR coamplification of 16S rRNA genes from mixed bacterial genomes. Appl Environ Microbiol 63: 4645–4650. 9406382 10.1128/aem.63.12.4645-4650.1997 PMC168786

[74] Haas BJ, Gevers D, Earl AM, Feldgarden M, Ward DV, et al (2011) Chimeric 16S rRNA sequence formation and detection in Sanger and 454-pyrosequenced PCR amplicons. Genome Res 21: 494–504. 21212162 10.1101/gr.112730.110 PMC3044863

[75] Stamm LV, Greene SR, Bergen HL, Hardham JM, Barnes NY (1998) Identification and sequence analysis of *Treponema pallidum tprJ*, a member of a polymorphic multigene family. FEMS Microbiol Lett 169: 155–163. 9851047 10.1111/j.1574-6968.1998.tb13312.x

[76] Sarkari J, Pandit N, Moxon ER, Achtman M (1994) Variable expression of the Opc outer membrane protein in *Neisseria meningitidis* is caused by size variation of a promoter containing poly-cytidine. Mol Microbiol 13: 207–217. 7984102 10.1111/j.1365-2958.1994.tb00416.x

[77] van der Ende A, Hopman CT, Zaat S, Essink BB, Berkhout B, et al (1995) Variable expression of class I outer membrane protein in *Neisseria meningitidis* is caused by variation in the spacing between the −10 and −35 regions of the promoter. J Bacteriol 177: 2475–2480. 7730280 10.1128/jb.177.9.2475-2480.1995 PMC176907

[78] Arhin FF, Moreau F, Coulton JW, Mills EL (1998) Sequencing of porA from clinical isolates of *Neisseria meningitidis* defines a subtyping scheme and its genetic regulation. Can J Microbiol 44: 56–63. 9522450

[79] Smajs D, McKevitt M, Wang L, Howell JK, Norris SJ, et al (2002) BAC library of *T. pallidum* DNA in E. coli. Genome Res 12: 515–522. 11875041 10.1101/gr.207302 PMC155280

[80] Nackley AG, Shabalina SA, Tchivileva IE, Satterfield K, Korchynskyi O, et al (2006) Human catechol-O-methyltransferase haplotypes modulate protein expression by altering mRNA secondary structure. Science 314: 1930–1933. 17185601 10.1126/science.1131262

[81] Komar AA (2007) Genetics. SNPs, silent but not invisible. Science 315: 466–467. 17185559 10.1126/science.1138239

[82] Komar AA (2007) Silent SNPs: impact on gene function and phenotype. Pharmacogenomics 8: 1075–1080. 17716239 10.2217/14622416.8.8.1075

[83] Kimchi-Sarfaty C, Oh JM, Kim IW, Sauna ZE, Calcagno AM, et al (2007) A “silent” polymorphism in the MDR1 gene changes substrate specificity. Science 315: 525–528. 17185560 10.1126/science.1135308

[84] World Health Organization (2012) Yaws. Fact sheet N°316 http://www.who.int/mediacentre/factsheets/fs316/en/

[85] Centurion-Lara A, Molini BJ, Godornes C, Sun E, Hevner K, et al (2006) Molecular differentiation of *Treponema pallidum* subspecies. J Clin Microbiol 44: 3377–3380. 16954278 10.1128/JCM.00784-06 PMC1594706

[86] Tantalo LC, Lukehart SA, Marra CM (2005) *Treponema pallidum* strain-specific differences in neuroinvasion and clinical phenotype in a rabbit model. J Infect Dis 191: 75–80. 15593006 10.1086/426510

[87] Small JD, Newman B (1972) Venereal spirochetosis of rabbits (rabbit syphilis) due to *Treponema cuniculi*: a clinical, serological, and histopathological study. Lab Anim Sci 22: 77–89. 4334440

[88] Smajs D, Norris SJ, Weinstock GM (2012) Genetic diversity in *Treponema pallidum*: Implications for pathogenesis, evolution and molecular diagnostics of syphilis and yaws. Infect Genet Evol 12: 191–202. 22198325 10.1016/j.meegid.2011.12.001 PMC3786143

[89] Hackett CJ (1963) On the Origin of the Human Treponematoses (Pinta, Yaws, Endemic Syphilis and Venereal Syphilis). Bull World Health Organ 29: 7–41. 14043755 PMC2554777

[90] Hudson EH (1965) Treponematosis in perspective. Bull World Health Org 32: 735–748. 5318224 PMC2555253

