# Supplementary material for: Fine Analysis of Genetic Diversity of the tpr Gene Family among Treponemal Species, Subspecies and Strains
Source: PLoS Negl Trop Dis. 2013 May 16;7(5):e2222. doi: 10.1371/journal.pntd.0002222 (PMC3656149; doi:10.1371/journal.pntd.0002222)
Supplement: Figure S1 — Predicted full length amino acid and DNA sequence alignments of the tpr gene family by locus. For the ORFs containing indels resulting in frameshifts and truncated proteins, only the encoded amino acid sequences before the premature stop codon are shown. In tprD locus, tprD2 alleles were excluded for clarity purposes only. In tprG locus, because of the significant dissimilarities among sequences, alignments are separated in two groups: GI and GJ chimeras. Red font, T. p. pallidum subspecies; blue T. p. pertenue; bright green, T. p. endemicum; yellow, the Simian treponeme; and pink, T. paraluiscuniculi. (DOCX) [file pntd.0002222.s001.docx]

**Supplemental Figure 1**

***1.1 tprA* Locus**

10 20 30 40 50 60 70 80

....|....|....|....|....|....|....|....|....|....|....|....|....|....|....|....|

**NicholsA**  **MVKTRYTTVCMRLVLGSCIFILLLRGRATVSRLHASPAVTISGSTRLTWGINLGAKANFVLPVAPLGATGTVRENPNHRF**

**Bal3A**  **MVKTRYTTVCMRLVLGSCIFILLLRGRATVSRLHASPAVTISGSTRLTWGINLGAKANFVLPVAPLGATGTVRENPNHRF**

**MexicoAA**  **MVKTRYTTVCMRLVLGSCIFILLLRGRATVSRLHASPAVTISGSTRLTWGINLGAKANFVPPVAPLGATGTVRENPNHRF**

**Street14A MVKTRYTTVCMRLVLGSCIFILLLRGRATVSRLHASPAVTISGSTRLTWGINLGAKANFVPPVAPLGATGTVRENPNHRF**

**Sea81-4A**  **MVKTRYTTVCMRLVLGSCIFILLLRGRATVSRLHASPAVTISGSTRLTWGINLGAKANFVLPVAPLGATGTVRENPNHRF**

**GauthierA**  **MVKTRYTTVCMRLVLGSCIFILLLRGRATVSRLHASPAVTISGSTRLTWGINLGAKANFVPPVAPLGATGTVRENPNHRF**

**SamoaDA**  **MVKTRYTTVCMRLVLGSCIFILLLRGRATVSRLHASPAVTISGSTRLTWGINLGAKANFVPPVAPLGATGTVRENPNHRF**

**CDC2A**  **MVKTRYTTVCMRLVLGSCIFILLLRGRATVSRLHASPAVTISGSTRLTWGINLGAKANFVPPVAPLGATGTVRENPNHRF**

**BosniaAA**  **MVKTRYTTVCMRLVLGSCIFILLLRGRATVSRLHASPAVTISGSTRLTWGINLGAKANFVPPVAPLGATGTVRENPNHRF**

**IraqBA**  **MVKTRYTTVCMRLVLGSCIFILLLRGRATVSRLHASPAVTISGSTRLTWGINLGAKANFVPPVAPLGATGTVRENPNHRF**

**Fribourg-BlancA** **MVKTRYTTVCMRLVLGSCIFILLLRGRATVSRLHASPAVTISGSTRLTWGINLGAKANFVPPVAPLGATGTVRENPNHRF**

**CuniculiAA**  **MMKIRYTTVCMRLVLGSCIFILLLRGRATVSRLHASPAVTISGSTRLTWGINLGAKANFVPPVAPSGATGNVRENPNHRF**

90 100 110 120 130 140 150 160

....|....|....|....|....|....|....|....|....|....|....|....|....|....|....|....|

**NicholsA**  **RHRRHGFRSSSTLFFSLTLCPPKTRSNLHKSSGVYAEILLRNLECALPLGSLSGEALGELTPTEKQSFSVEATLRFYGAY**

**Bal3A**  **RHRRHGFRSSSTLFFSLTLCPPKTRSNLHKSSGVYAEILLRNLECALPLGSLSGEALGELTPTEKQSFSVEATLRFYGAY**

**MexicoAA**  **RHRRHGFRSSSTLFFSLTLCPPKTRSNLHKSSGVYAEILLRNLECALPLGSLSGEALGELTPTEKQSFSVEATLRFYGAY**

**Street14A RHRRHGFRSSSTLFFSLTLCPPKTRSNLHKSSGVYAEILLRNLECALPLGSLSGEALGELTPTEKQSFSVEATLRFYGAY**

**Sea81-4A**  **RHRRHGFRSSSTLFFSLTLCPPKTRSNLHKSSGVYAEILLRNLECALPLGSLSGEALGELTPTEKQSFSVEATLRFYGAY**

**GauthierA**  **RHRRHGFRSSSTLFFSLTLCPPKTRSNLHKSSGVYAEILLRNLECALPLGSLSGEALGELTPTEKQSFSVEATLRFYGAY**

**SamoaDA**  **RHRRHGFRSSSTLFFSLTLCPPKTRSNLHKSSGVYAEILLRNLECALLLGSLSGEALGELTPTEKQSFSVEATLRFYGAY**

**CDC2A**  **RHRRHGFRSSSTLFFSLTLCPPKTRSNLHKSSGVYAEILLRNLECALPLGSLSGEALGELTPTEKQSFSVEATLRFYGAY**

**BosniaAA**  **RHRRHGFRSSSTLFFSLTLCPPKTRSNLHKSSGVYAEILLRNLECALPLGSLSGEALGELTPTEKQSFSVEATLRFYGAY**

**IraqBA**  **RHRRHGFRSSSTLFFSLTLCPPKTRSNLHKSSGVYAEILLRNLECALPLGSLSGEALGELTPTEKQSFSVEATLRFYGAY**

**Fribourg-BlancA** **RHRRHGFRSSSTLFFSLTLCPPKTRSNLHKSSGVYAEILLRNLECALPLGSLSGEALGELTPTEKQSFSVEATLRFYGAY**

**CuniculiAA**  **RHRRHGFRSSSTLFFSLTLCPPKTRSNLHKSSGVYAEILLRNLECALPLGSLSGETLGELTPTEKQSFSVEATLRFYGAY**

170 180 190 200 210 220 230 240

....|....|....|....|....|....|....|....|....|....|....|....|....|....|....|....|

**NicholsA**  **LTIGKNPTFSKNFAKLWPPFITTRYKEADTQYAPGFGGYGGKIGYRVEDVGNSGLGFDFGFLSFASNGDWSTSGTSHSKY**

**Bal3A**  **LTIGKNPTFSKNFAKLWPPFITTRYKEADTQYAPGFGGYGGKIGYRVEDVGNSGLGFDFGFLSFASNGDWSTSGTSHSKY**

**MexicoAA**  **LTIGKNPTFSKNFAKLWPPFITTRYKEADTQYAPGFGGYGGKIGYRVEDVGNSGLGFDFGFLSFASNGDWSTSGTSHSKY**

**Street14A LTIGKNPTFSKNFAKLWPPFITTRYKEADTQYAPGFGGYGGKIGYRVEDVGNSGLGFDFGFLSFASNGDWSTSGTSHSKY**

**Sea81-4A**  **LTIGKNPTFSKNFAKLWPPFITTRYKEADTQYAPGFGGYGGKIGYRVEDVGNSGLGFDFGFLSFASNGDWSTSGTSHSKY**

**GauthierA**  **LTIGKNPTFSKNFAKLWPPFITTRYKEADTQYAPGFGGYGGKIGYRVEDVGNSGLGFDFGFLSFASNGDWSTSGTSHSKY**

**SamoaDA**  **LTIGKNPTFSKNFAKLWPPFITTRYKEADTQYAPGFGGYGGKIGYRVEDVGNSGLGFDFGFLSFASNGDWSTSGTSHSKY**

**CDC2A**  **LTIGKNPTFSKNFAKLWPPFITTRYKEADTQYAPGFGGYGGKIGYRVEDVGNSGLGFDFGFLSFASNGDWSTSGTSHSKY**

**BosniaAA**  **LTIGKNPTFSKNFAKLWPPFITTRYKEADTQYAPGFGGYGGKIGYRVEDVGNSGLGFDFGFLSFASNGDWSTSGTSHSKY**

**IraqBA**  **LTIGKNPTFSKNFAKLWPPFITTRYKEADTQYAPGFGGYGGKIGYRVEDVGNSGLGFDFGFLSFASNGDWSTSGTSHSKY**

**Fribourg-BlancA** **LTIGKNPTFSKNFAKLWPPFITTRYKEADTQYAPGFGGYGGKIGYRVEDVGNSGLGFDFGFLSFASNGDWSTSGTSHSKY**

**CuniculiAA**  **LTIGKNPTFSKNFAKLWPLFITTRYKEADTQYAPGFGDYGGKIGYRVEDVGNSGLGFDFGFLSFASNGDWSTSGTSHSKY**

250 260 270 280 290 300 310 320

....|....|....|....|....|....|....|....|....|....|....|....|....|....|....|....|

**NicholsA**  **GFGSDLSMVQEKQEAVFNCGTRR**

**Bal3A**  **GFGSDLSMVQEKQEAVFNCGTRR**

**MexicoAA**  **GFGSDLSMVQEKQEAVFNCGTRR**

**Street14A GFGSDLSMVQEKQEAVFNCGTRR**

**Sea81-4A**  **GFGSDLSLWYKRNKKLFLTVELAGNATLQEGYATLAPTFSGAPNNKRASHALLWSVGGRLSIMPGAGFRFILATDAGNTY**

**GauthierA**  **GFGSDLSLWYKRNKKLFLTVELAGNATLQEGYATLAPTFSGAPNNKRASHALLWSVGGRLSIMPGAGFRFILATDAGNTY**

**SamoaDA**  **GFGSDLSLWYKRNKKLFLTVELAGNATLQEGYATLAPTFSGAPNNKRASHALLWSVGGRLSIMPGAGFRFILATDAGNTY**

**CDC2A**  **GFGSDLSLWYKRNKKLFLTVELAGNATLQEGYATLAPTFSGAPNNKRASHALLWSVGGRLSIMPGAGFRFILATDAGNTY**

**BosniaAA**  **GFGSDLSLWYKRNKKLFLTVELAGNATLQEGYATLAPTFSGAPNNKRASHALLWSVGGRLSIMPGAGFRFILATDAGNTY**

**IraqBA**  **GFGSDLSLWYKRNKKLFLTVELAGNATLQEGYATLAPTFSGAPNNKRASHALLWSVGGRLSIMPGAGFRFILATDAGNTY**

**Fribourg-BlancA** **GFGSDLSLWYKRNKKLFLTVELAGNATLQEGYATLAPTFSGAPNNKRASHALLWSVGGRLSIMPGAGFRFILATDAGNTY**

**CuniculiAA**  **GFGSDLSLWYKRNKKLFLTVELAGNATLQEGYATLDSRFLGAPNNKRAPHALLWSVGGRLSIMPGAGFRFILATDAGNTY**

330 340 350 360 370 380 390 400

....|....|....|....|....|....|....|....|....|....|....|....|....|....|....|....|

**NicholsA**

**Bal3A**

**MexicoAA**

**Street14A**

**Sea81-4A**  **RDTNSARARVVEQALELAEKTYPSLRTVRRIFSWMVQHVDSLGIDALVTAQWRWLSGGVYGATGAASVFGSGPFVKSTFQ**

**GauthierA**  **RDTNSARARVVEQALELAEKTYPSLRTVRRIFSWMVQHVDSLGIDALVTAQWRWLSGGVYGATGAASVFGSGPFVKSTFQ**

**SamoaDA**  **RDTNSARARVVEQALELAEKTYPSLRTVRRIFSWMVQHVDSLGIDALVTAQWRWLSGGVYGATGAASVFGSGPFVKSTFQ**

**CDC2A**  **RDTNSARARVVEQALELAEKTYPSLRTVRRIFSWMVQHVDSLGIDALVTAQWRWLSGGVYGATGAASVFGSGPFVKSTFQ**

**BosniaAA**  **RDTNSARARVVEQALELAEKTYPSLRTVRRIFSWMVQHVDSLGIDALVTAQWRWLSGGVYGATGAASVFGSGPFVKSTFQ**

**IraqBA**  **RDTNSARARVVEQALELAEKTYPSLRTVRRIFSWMVQHVDSLGIDALVTAQWRWLSGGVYGATGAASVFGSGPFVKSTFQ**

**Fribourg-BlancA** **RDTNSARARVVEQALELAEKTYPSLRTVRRIFSWMVQHVDSLGIDALVTAQWRWLSGGVYGATGAASVFGSGPFVKSTFQ**

**CuniculiAA**  **RDTNSARARVVEQALKFAEKTYPSLRTVRRIFSWMVQHVDSLGIDALVTAQWRWLSGGVYGATGAASVFGSSPFVNSTFQ**

410 420 430 440 450 460 470 480

....|....|....|....|....|....|....|....|....|....|....|....|....|....|....|....|

**NicholsA**

**Bal3A**

**MexicoAA**

**Street14A**

**Sea81-4A**  **YTDFAAFLRLETRSGDDYTHALHGLNAGIEARVYLPLGYKSYLDNEGLPPDVVPAQGVAQQVANAARVGILGLLNFLNSA**

**GauthierA**  **YTDFAAFLRLETRSGDDYTHALHGLNAGIEARVYLPLDYKSYLDNGGLPPDVVPAQGVAQQVANAARVGXLGLLNFLNSA**

**SamoaDA**  **YTDFAAFLRLETRSGDDYTHALHGLNAGIEARVYLPLDYKSYLDNGGLPPDVVPAQGVAQQVANAARVGILGLLNFLNSA**

**CDC2A**  **YTDFAAFLRLETRSGDDYTHALHGLNAGIEARVYLPLDYKSYLDNGGLPPDVVPAQGVAQQVANAARVGILGLLNFLNSA**

**BosniaAA**  **YTDFAAFLRLETRSGDDYTHALHGLNAGIEARVYLPLDYKSYLDNGGLPPDVVPAQGVAQQVANAARVGILGLLNFLNSA**

**IraqBA**  **YTDFAAFLRLETRSGDDYTHALHGLNAGIEARVYLPLDYKSYLDNGGLPPDVVPAQGVAQQVANAARVGILGLLNFLNSA**

**Fribourg-BlancA** **YTDFAAFLRLETRSGDDYTHALHGLNAGIEARVYLPLDYKSYLDNGGLPPDVVPAQGVAQQVANAARVGILGLLNFLNSA**

**CuniculiAA**  **YTDFAAFLRLETRSGDDYTHALHGLNAGIEARVYLPLTYKSYLDNGGLPPDVVPARGVAQQVANIFEAGLRNIFGFFNRA**

490 500 510 520 530 540 550 560

....|....|....|....|....|....|....|....|....|....|....|....|....|....|....|....|

**NicholsA**

**Bal3A**

**MexicoAA**

**Street14A**

**Sea81-4A**  **TNFVARAAGLPADAIATGAASIALPIMGNAWVGYRIPCYDSMWIEPRAHIYMATNRFNFNGFKGTRNLKGELCFQYAVEL**

**GauthierA**  **TNFVARAAGLPADAIATGAASIALPIMGNAWVGYRIPCYDSMWIEPRAHIYMATNRFNFNGFKGTRNLKGELCFQYAVEL**

**SamoaDA**  **TNFVARAAGLPADAIATGAASIALPIMGNAWVGYRIPCYDSMWIEPRAHIYMATNRFNFNGFKGTRNLKGELCFQYAVEL**

**CDC2A**  **TNFVARAAGLPADAIATGAASIALPIMGNAWVGYRIPCYDSMWIEPRAHIYMATNRFNFNGFKGTRNLKGELCFQYAVEL**

**BosniaAA**  **TNFVARAAGLPADAIATGAASIALPIMGNAWVGYRIPCYDSMWIEPRAHIYMATNRFNFNGFKGTRNLKGELCFQYAVEL**

**IraqBA**  **TNFVARAAGLPADAIATGAASIALPIMGNAWVGYRIPCYDSMWIEPRAHIYMATNRFNFNGFKGTRNLKGELCFQYAVEL**

**Fribourg-BlancA** **TNFVARAAGLPADAIATGAASIALPIMGNAWVGYRIPCYDSMWIEPRAHIYMATNRFNFNGFKGTRNLKGELCFQYAVEL**

**CuniculiAA**  **VNFVARAAGLPADAITTGAASIALPIMGNAWVGYRIPCYDSMWIEPRAHIYMATNRFNFNGFKGTHNLKGELCFQYAVEL**

570 580 590 600

....|....|....|....|....|....|....|....|....|...

**NicholsA**

**Bal3A**

**MexicoAA**

**Street14A**

**Sea81-4A**  **RASPIKPVEFSVRWEQGLLSQDPYMLIEENWSWPGYTGSLFLGCKITW**

**GauthierA**  **RASXIKPVEFSVRWEQGLLSQDPYMLIEENWSWPGYTGSLFLGCKIT**

**SamoaDA**  **RASPIKPVEFSVRWEQGLLSQDPYMLIEENWSWPGYTGSLFLGCKIT**

**CDC2A**  **RASPIKPVEFSVRWEQGLLSQDPYMLIEENWSWPGYTGSLFLGCKIT**

**BosniaAA**  **RASPIKPVEFSVRWEQGLLSQDPYMLIEENWSWPGYTGSLFLGCKIT**

**IraqBA**  **RASPIKPVEFSVRWEQGLLSQDPYMLIEENWSWPGYTGSLFLGCKIT**

**Fribourg-BlancA** **RASPIKPVEFSVRWEQGLLSQDPYMLIEENWSWPGYTGSLFLGCKIT**

**CuniculiAA**  **HASPIKHVEFSVRWEQGLLSQDPYMLIEENWSWPGYTGSLFLECKITW**

10 20 30 40 50 60 70 80

....|....|....|....|....|....|....|....|....|....|....|....|....|....|....|....|

**NicholsA**  **gtggtgaaaacacggtacactactgtatgcatgcgtctagtgttaggctcgtgcatttttatacttttactccgagggcg**

**Bal3A**  **gtggtgaaaacacggtacactactgtatgcatgcgtctagtgttaggctcgtgcatttttatacttttactccgagggcg**

**MexicoAA**  **gtggtgaaaacacggtacactactgtatgcatgcgtctagtgttaggctcgtgcatttttatacttttactccgagggcg**

**Street14A gtggtgaaaacacggtacactactgtatgcatgcgtctagtgttaggctcgtgcatttttatacttttactccgagggcg**

**Sea81-4A**  **gtggtgaaaacacggtacactactgtatgcatgcgtctagtgttaggctcgtgcatttttatacttttactccgagggcg**

**GauthierA**  **gtggtgaaaacacggtacactactgtatgcatgcgtctagtgttaggctcgtgcatttttatacttttactccgagggcg**

**SamoaDA**  **gtggtgaaaacacggtacactactgtatgcatgcgtctagtgttaggctcgtgcatttttatacttttactccgagggcg**

**CDC2A**  **gtggtgaaaacacggtacactactgtatgcatgcgtctagtgttaggctcgtgcatttttatacttttactccgagggcg**

**BosniaAA**  **gtggtgaaaacacggtacactactgtatgcatgcgtctagtgttaggctcgtgcatttttatacttttactccgagggcg**

**IraqBA**  **gtggtgaaaacacggtacactactgtatgcatgcgtctagtgttaggctcgtgcatttttatacttttactccgagggcg**

**Fribourg-BlancA** **gtggtgaaaacacggtacactactgtatgcatgcgtctagtgttaggctcgtgcatttttatacttttactccgagggcg**

**CuniculiAA**  **gtgatgaaaatacggtacactactgtatgcatgcgtctagtgttaggctcgtgcatttttatacttttactccgagggcg**

90 100 110 120 130 140 150 160

....|....|....|....|....|....|....|....|....|....|....|....|....|....|....|....|

**NicholsA**  **cgctaccgtatcgcgtctgcacgcgagcccggccgtcaccatttcggggagtactcgtcttacttggggcattaacttag**

**Bal3A**  **cgctaccgtatcgcgtctgcacgcgagcccggccgtcaccatttcggggagtactcgtcttacttggggcattaacttag**

**MexicoAA**  **cgctaccgtatcgcgtctgcacgcgagcccggccgtcaccatttcggggagtactcgtcttacttggggcattaacttag**

**Street14A cgctaccgtatcgcgtctgcacgcgagcccggccgtcaccatttcggggagtactcgtcttacttggggcattaacttag**

**Sea81-4A**  **cgctaccgtatcgcgtctgcacgcgagcccggccgtcaccatttcggggagtactcgtcttacttggggcattaacttag**

**GauthierA**  **cgctaccgtatcgcgtctgcacgcgagcccggccgtcaccatttcggggagtactcgtcttacttggggcattaacttag**

**SamoaDA**  **cgctaccgtatcgcgtctgcacgcgagcccggccgtcaccatttcggggagtactcgtcttacttggggcattaacttag**

**CDC2A**  **cgctaccgtatcgcgtctgcacgcgagcccggccgtcaccatttcggggagtactcgtcttacttggggcattaacttag**

**BosniaAA**  **cgctaccgtatcgcgtctgcacgcgagcccggccgtcaccatttcggggagtactcgtcttacttggggcattaacttag**

**IraqBA**  **cgctaccgtatcgcgtctgcacgcgagcccggccgtcaccatttcggggagtactcgtcttacttggggcattaacttag**

**Fribourg-BlancA** **cgctaccgtatcgcgtctgcacgcgagcccggccgtcaccatttcggggagtactcgtcttacttggggcattaacttag**

**CuniculiAA**  **cgctaccgtgtcgcgtctgcacgcgagcccggccgtcaccatttcggggagtactcgtcttacttggggtattaacttag**

170 180 190 200 210 220 230 240

....|....|....|....|....|....|....|....|....|....|....|....|....|....|....|....|

**NicholsA**  **gcgcgaaggcgaacttcgtgctacccgtagcaccgcttggggcaaccggcactgtgcgagagaaccccaatcatcgcttc**

**Bal3A**  **gcgcgaaggcgaacttcgtgctacccgtagcaccgcttggggcaaccggcactgtgcgagagaaccccaatcatcgcttc**

**MexicoAA**  **gcgcgaaggcgaacttcgtgccacccgtagcaccgcttggggcaaccggcactgtgcgagagaaccccaatcatcgcttc**

**Street14A gcgcgaaggcgaacttcgtgccacccgtagcaccgcttggggcaaccggcactgtgcgagagaaccccaatcatcgcttc**

**Sea81-4A**  **gcgcgaaggcgaacttcgtgctacccgtagcaccgcttggggcaaccggcactgtgcgagagaaccccaatcatcgcttc**

**GauthierA**  **gcgcgaaggcgaacttcgtgccacccgtagcaccgcttggggcaaccggcactgtgcgagagaaccccaatcatcgcttc**

**SamoaDA**  **gcgcgaaggcgaacttcgtgccacccgtagcaccgcttggggcaaccggcactgtgcgagagaaccccaatcatcgcttc**

**CDC2A**  **gcgcgaaggcgaacttcgtgccacccgtagcaccgcttggggcaaccggcactgtgcgagagaaccccaatcatcgcttc**

**BosniaAA**  **gcgcgaaggcgaacttcgtgccacccgtagcaccgcttggggcaaccggcactgtgcgagagaaccccaatcatcgcttc**

**IraqBA**  **gcgcgaaggcgaacttcgtgccacccgtagcaccgcttggggcaaccggcactgtgcgagagaaccccaatcatcgcttc**

**Fribourg-BlancA** **gcgcgaaggcgaacttcgtgccacccgtagcaccgcttggggcaaccggcactgtgcgagagaaccccaatcatcgcttc**

**CuniculiAA**  **gcgcgaaggcgaacttcgtgccacccgtagcaccgtctggggcaaccggcaatgtgcgagagaaccccaatcatcgcttc**

250 260 270 280 290 300 310 320

....|....|....|....|....|....|....|....|....|....|....|....|....|....|....|....|

**NicholsA**  **cgtcatcgcagacacggttttaggagttccagtactctctttttctcgctgacgctttgtccaccgaaaactcggtcgaa**

**Bal3A**  **cgtcatcgcagacacggttttaggagttccagtactctctttttctcgctgacgctttgtccaccgaaaactcggtcgaa**

**MexicoAA**  **cgtcatcgcagacacggttttaggagttccagtactctctttttctcgctgacgctttgtccaccgaaaactcggtcgaa**

**Street14A cgtcatcgcagacacggttttaggagttccagtactctctttttctcgctgacgctttgtccaccgaaaactcggtcgaa**

**Sea81-4A**  **cgtcatcgcagacacggttttaggagttccagtactctctttttctcgctgacgctttgtccaccgaaaactcggtcgaa**

**GauthierA**  **cgtcatcgcagacacggttttaggagttccagtactctctttttctcgctgacgctttgtccaccgaaaactcggtcgaa**

**SamoaDA**  **cgtcatcgcagacacggttttaggagttccagtactctctttttctcgctgacgctttgtccaccgaaaactcggtcgaa**

**CDC2A**  **cgtcatcgcagacacggttttaggagttccagtactctctttttctcgctgacgctttgtccaccgaaaactcggtcgaa**

**BosniaAA**  **cgtcatcgcagacacggttttaggagttccagtactctctttttctcgctgacgctttgtccaccgaaaactcggtcgaa**

**IraqBA**  **cgtcatcgcagacacggttttaggagttccagtactctctttttctcgctgacgctttgtccaccgaaaactcggtcgaa**

**Fribourg-BlancA** **cgtcatcgcagacacggttttaggagttccagtactctctttttctcgctgacgctttgtccaccgaaaactcggtcgaa**

**CuniculiAA**  **cgtcatcgcagacacggttttaggagttccagtactctctttttctcgctgacgctttgtccaccgaaaactcggtcgaa**

330 340 350 360 370 380 390 400

....|....|....|....|....|....|....|....|....|....|....|....|....|....|....|....|

**NicholsA**  **tctgcataaaagcagcggtgtgtatgcagaaatcctgttaaggaacctagagtgtgcgctccccctcggttccttatctg**

**Bal3A**  **tctgcataaaagcagcggtgtgtatgcagaaatcctgttaaggaacctagagtgtgcgctccccctcggttccttatctg**

**MexicoAA**  **tctgcataaaagcagcggtgtgtatgcagaaatcctgttaaggaacctagagtgtgcgctccccctcggttccttatctg**

**Street14A tctgcataaaagcagcggtgtgtatgcagaaatcctgttaaggaacctagagtgtgcgctccccctcggttccttatctg**

**Sea81-4A**  **tctgcataaaagcagcggtgtgtatgcagaaatcctgttaaggaacctagagtgtgcgctccccctcggttccttatctg**

**GauthierA**  **tctgcataaaagcagcggtgtgtatgcagaaatcctgttaaggaacctagagtgtgcgctccccctcggttccttatctg**

**SamoaDA**  **tctgcataaaagcagcggtgtgtatgcagaaatcctgttaaggaacctagagtgtgcgctcctcctcggttccttatctg**

**CDC2A**  **tctgcataaaagcagcggtgtgtatgcagaaatcctgttaaggaacctagagtgtgcgctccccctcggttccttatctg**

**BosniaAA**  **tctgcataaaagcagcggtgtgtatgcagaaatcctgttaaggaacctagagtgtgcgctccccctcggttccttatctg**

**IraqBA**  **tctgcataaaagcagcggtgtgtatgcagaaatcctgttaaggaacctagagtgtgcgctccccctcggttccttatctg**

**Fribourg-BlancA** **tctgcataaaagcagcggtgtgtatgcagaaatcctgttaaggaacctagagtgtgcgctccccctcggttccttatctg**

**CuniculiAA**  **tctgcataaaagcagcggtgtgtatgcagaaatcctgttaaggaacctagagtgtgcgctcccccttggttccttatctg**

410 420 430 440 450 460 470 480

....|....|....|....|....|....|....|....|....|....|....|....|....|....|....|....|

**NicholsA**  **gtgaggctttaggcgaactcacgcccacagaaaaacaaagcttctccgtagaagcgacccttcgcttctacggcgcatat**

**Bal3A**  **gtgaggctttaggcgaactcacgcccacagaaaaacaaagcttctccgtagaagcgacccttcgcttctacggcgcatat**

**MexicoAA**  **gtgaggctttaggcgaactcacgcccacagaaaaacaaagcttctccgtagaagcgacccttcgcttctacggcgcatat**

**Street14A gtgaggctttaggcgaactcacgcccacagaaaaacaaagcttctccgtagaagcgacccttcgcttctacggcgcatat**

**Sea81-4A**  **gtgaggctttaggcgaactcacgcccacagaaaaacaaagcttctccgtagaagcgacccttcgcttctacggcgcatat**

**GauthierA**  **gtgaggctttaggcgaactcacgcccacagaaaaacaaagcttctccgtagaagcgacccttcgcttctacggcgcatat**

**SamoaDA**  **gtgaggctttaggcgaactcacgcccacagaaaaacaaagcttctccgtagaagcgacccttcgcttctacggcgcatat**

**CDC2A**  **gtgaggctttaggcgaactcacgcccacagaaaaacaaagcttctccgtagaagcgacccttcgcttctacggcgcatat**

**BosniaAA**  **gtgaggctttaggcgaactcacgcccacagaaaaacaaagcttctccgtagaagcgacccttcgcttctacggcgcatat**

**IraqBA**  **gtgaggctttaggcgaactcacgcccacagaaaaacaaagcttctccgtagaagcgacccttcgcttctacggcgcatat**

**Fribourg-BlancA** **gtgaggctttaggcgaactcacgcccacagaaaaacaaagcttctccgtagaagcgacccttcgcttctacggcgcatat**

**CuniculiAA**  **gtgagactttaggcgaactcacgcccacagaaaaacaaagcttctccgtagaagcgacccttcgcttctacggcgcatat**

490 500 510 520 530 540 550 560

....|....|....|....|....|....|....|....|....|....|....|....|....|....|....|....|

**NicholsA**  **ctcactattggaaaaaatccgaccttttctaaaaattttgccaaattgtggcccccgttcatcaccacacgatacaagga**

**Bal3A**  **ctcactattggaaaaaatccgaccttttctaaaaattttgccaaattgtggcccccgttcatcaccacacgatacaagga**

**MexicoAA**  **ctcactattggaaaaaatccgaccttttctaaaaattttgccaaattgtggcccccgttcatcaccacacgatacaagga**

**Street14A ctcactattggaaaaaatccgaccttttctaaaaattttgccaaattgtggcccccgttcatcaccacacgatacaagga**

**Sea81-4A**  **ctcactattggaaaaaatccgaccttttctaaaaattttgccaaattgtggcccccgttcatcaccacacgatacaagga**

**GauthierA**  **ctcactattggaaaaaatccgaccttttctaaaaattttgccaaattgtggcccccgttcatcaccacacgatacaagga**

**SamoaDA**  **ctcactattggaaaaaatccgaccttttctaaaaattttgccaaattgtggcccccgttcatcaccacacgatacaagga**

**CDC2A**  **ctcactattggaaaaaatccgaccttttctaaaaattttgccaaattgtggcccccgttcatcaccacacgatacaagga**

**BosniaAA**  **ctcactattggaaaaaatccgaccttttctaaaaattttgccaaattgtggcccccgttcatcaccacacgatacaagga**

**IraqBA**  **ctcactattggaaaaaatccgaccttttctaaaaattttgccaaattgtggcccccgttcatcaccacacgatacaagga**

**Fribourg-BlancA** **ctcactattggaaaaaatccgaccttttctaaaaattttgccaaattgtggcccccgttcatcaccacacgatacaagga**

**CuniculiAA**  **ctcactattggaaaaaatccgaccttttctaaaaattttgccaaattgtggcccctgttcatcaccacacgatacaagga**

570 580 590 600 610 620 630 640

....|....|....|....|....|....|....|....|....|....|....|....|....|....|....|....|

**NicholsA**  **agcagacacccaatacgcccctggctttgggggttatggagggaagattggttaccgcgtagaagacgtcgggaattccg**

**Bal3A**  **agcagacacccaatacgcccctggctttgggggttatggagggaagattggttaccgcgtagaagacgtcgggaattccg**

**MexicoAA**  **agcagacacccaatacgcccctggctttgggggttatggagggaagattggttaccgcgtagaagacgtcgggaattccg**

**Street14A agcagacacccaatacgcccctggctttgggggttatggagggaagattggttaccgcgtagaagacgtcgggaattccg**

**Sea81-4A**  **agcagacacccaatacgcccctggctttgggggttatggagggaaaattggttaccgcgtagaagacgtcgggaattccg**

**GauthierA**  **agcagacacccaatacgcccctggctttgggggttatggagggaagattggttaccgcgtagaagacgtcgggaattccg**

**SamoaDA**  **agcagacacccaatacgcccctggctttgggggttatggagggaagattggttaccgcgtagaagacgtcgggaattccg**

**CDC2A**  **agcagacacccaatacgcccctggctttgggggttatggagggaagattggttaccgcgtagaagacgtcgggaattccg**

**BosniaAA**  **agcagacacccaatacgcccctggctttgggggttatggagggaagattggttaccgcgtagaagacgtcgggaattccg**

**IraqBA**  **agcagacacccaatacgcccctggctttgggggttatggagggaagattggttaccgcgtagaagacgtcgggaattccg**

**Fribourg-BlancA** **agcagacacccaatacgcccctggctttgggggttatggagggaagattggttaccgcgtagaagacgtcgggaattccg**

**CuniculiAA**  **agcagacacccaatacgcccctggctttggggattatggagggaagattggttaccgcgtagaagacgtcgggaattccg**

650 660 670 680 690 700 710 720

....|....|....|....|....|....|....|....|....|....|....|....|....|....|....|....|

**NicholsA**  **ggctaggttttgactttgggttcctttccttcgcttcaaacggcgactggagcacgagcgggactagccatagcaaatat**

**Bal3A**  **ggctaggttttgactttgggttcctttccttcgcttcaaacggcgactggagcacgagcgggactagccatagcaaatat**

**MexicoAA**  **ggctaggttttgactttgggttcctttccttcgcttcaaacggcgactggagcacgagcgggactagccatagcaaatat**

**Street14A ggctaggttttgactttgggttcctttccttcgcttcaaacggcgactggagcacgagcgggactagccatagcaaatat**

**Sea81-4A**  **ggctaggttttgactttgggttcctttccttcgcttcaaacggcgactggagcacgagcgggactagccatagcaaatat**

**GauthierA**  **ggctaggttttgactttgggttcctttccttcgcttcaaacggcgactggagcacgagcgggactagccatagcaaatat**

**SamoaDA**  **ggctaggttttgactttgggttcctttccttcgcttcaaacggcgactggagcacgagcgggactagccatagcaaatat**

**CDC2A**  **ggctaggttttgactttgggttcctttccttcgcttcaaacggcgactggagcacgagcgggactagccatagcaaatat**

**BosniaAA**  **ggctaggttttgactttgggttcctttccttcgcttcaaacggcgactggagcacgagcgggactagccatagcaaatat**

**IraqBA**  **ggctaggttttgactttgggttcctttccttcgcttcaaacggcgactggagcacgagcgggactagccatagcaaatat**

**Fribourg-BlancA** **ggctaggttttgactttgggttcctttccttcgcttcaaacggcgactggagcacgagcgggactagccatagcaaatat**

**CuniculiAA**  **ggctaggttttgactttgggttcctttccttcgcttcaaacggcgactggagcacgagcgggactagccatagcaaatat**

730 740 750 760 770 780 790 800

....|....|....|....|....|....|....|....|....|....|....|....|....|....|....|....|

**NicholsA**  **gggtttggtagtgac--ctctctatggtacaagagaaacaagaagctgtttttaactgtggaactcgccggtaatgctac**

**Bal3A**  **gggtttggtagtgac--ctctctatggtacaagagaaacaagaagctgtttttaactgtggaactcgccggtaatgctac**

**MexicoAA**  **gggtttggtagtgac--ctctctatggtacaagagaaacaagaagctgtttttaactgtggaactcgccggtaatgctac**

**Street14A gggtttggtagtgac--ctctctatggtacaagagaaacaagaagctgtttttaactgtggaactcgccggtaatgctac**

**Sea81-4A**  **gggtttggtagtgacctctctctatggtacaagagaaacaagaagctgtttttaactgtggaactcgccggtaatgctac**

**GauthierA**  **gggtttggtagtgacctctctctatggtacaagagaaacaagaagctgtttttaactgtggaactcgccggtaatgctac**

**SamoaDA**  **gggtttggtagtgacctctctctatggtacaagagaaacaagaagctgtttttaactgtggaactcgccggtaatgctac**

**CDC2A**  **gggtttggtagtgacctctctctatggtacaagagaaacaagaagctgtttttaactgtggaactcgccggtaatgctac**

**BosniaAA**  **gggtttggtagtgacctctctctatggtacaagagaaacaagaagctgtttttaactgtggaactcgccggtaatgctac**

**IraqBA**  **gggtttggtagtgacctctctctatggtacaagagaaacaagaagctgtttttaactgtggaactcgccggtaatgctac**

**Fribourg-BlancA** **gggtttggtagtgacctctctctatggtacaagagaaacaagaagctgtttttaactgtggaactcgccggtaatgctac**

**CuniculiAA**  **gggtttggtagtgacctctctctatggtacaagagaaacaagaagctgtttttaactgtggaactcgccggtaatgctac**

810 820 830 840 850 860 870 880

....|....|....|....|....|....|....|....|....|....|....|....|....|....|....|....|

**NicholsA**  **cctccaggagggttatgccacgttagctccaacattttcgggagcacccaacaacaaacgggcatcccacgcgctcttat**

**Bal3A**  **cctccaggagggttatgccacgttagctccaacattttcgggagcacccaacaacaaacgggcatcccacgcgctcttat**

**MexicoAA**  **cctccaggagggttatgccacgttagctccaacattttcgggagcacccaacaacaaacgggcatcccacgcgctcttat**

**Street14A cctccaggagggttatgccacgttagctccaacattttcgggagcacccaacaacaaacgggcatcccacgcgctcttat**

**Sea81-4A**  **cctccaggagggttatgccacgttagctccaacattttcgggagcaccaaacaacaaacgggcatcccacgcgctcttat**

**GauthierA**  **cctccaggagggttatgccacgttagctccaacattttcgggagcaccaaacaacaaacgggcatcccacgcgctcttat**

**SamoaDA**  **cctccaggagggttatgccacgttagctccaacattttcgggagcaccaaacaacaaacgggcatcccacgcgctcttat**

**CDC2A**  **cctccaggagggttatgccacgttagctccaacattttcgggagcaccaaacaacaaacgggcatcccacgcgctcttat**

**BosniaAA**  **cctccaggagggttatgccacgttagctccaacattttcgggagcaccaaacaacaaacgggcatcccacgcgctcttat**

**IraqBA**  **cctccaggagggttatgccacgttagctccaacattttcgggagcaccaaacaacaaacgggcatcccacgcgctcttat**

**Fribourg-BlancA** **cctccaggagggttatgccacgttagctccaacattttcgggagcaccaaacaacaaacgggcatcccacgcgctcttat**

**CuniculiAA**  **cctccaggagggttatgccacgttggattcaagatttttgggagcacccaacaacaaacgggcaccccacgcactcttat**

890 900 910 920 930 940 950 960

....|....|....|....|....|....|....|....|....|....|....|....|....|....|....|....|

**NicholsA**  **ggagtgtgggagggcgtctttcgatcatgcctggtgcaggattccgcttcattttagctacggatgccggaaatacctac**

**Bal3A**  **ggagtgtgggagggcgtctttcgatcatgcctggtgcaggattccgcttcattttagctacggatgccggaaatacctac**

**MexicoAA**  **ggagtgtgggagggcgtctttcgatcatgcctggtgcaggattccgcttcattttagctacggatgccggaaatacctac**

**Street14A ggagtgtgggagggcgtctttcgatcatgcctggtgcaggattccgcttcattttagctacggatgccggaaatacctac**

**Sea81-4A**  **ggagtgtgggagggcgtctttcgatcatgcctggtgcaggattccgcttcattttagctacggatgccggaaatacctac**

**GauthierA**  **ggagtgtgggagggcgtctttcgatcatgcctggtgcaggattccgcttcattttagctacggatgccggaaatacctac**

**SamoaDA**  **ggagtgtgggagggcgtctttcgatcatgcctggtgcaggattccgcttcattttagctacggatgccggaaatacctac**

**CDC2A**  **ggagtgtgggagggcgtctttcgatcatgcctggtgcaggattccgcttcattttagctacggatgccggaaatacctac**

**BosniaAA**  **ggagtgtgggagggcgtctttcgatcatgcctggtgcaggattccgcttcattttagctacggatgccggaaatacctac**

**IraqBA**  **ggagtgtgggagggcgtctttcgatcatgcctggtgcaggattccgcttcattttagctacggatgccggaaatacctac**

**Fribourg-BlancA** **ggagtgtgggagggcgtctttcgatcatgcctggtgcaggattccgcttcattttagctacggatgccggaaatacctac**

**CuniculiAA**  **ggagtgtgggagggcgtctttcgatcatgcctggtgcaggattccgcttcattttagctacggatgctggaaatacctac**

970 980 990 1000 1010 1020 1030 1040

....|....|....|....|....|....|....|....|....|....|....|....|....|....|....|....|

**NicholsA**  **cgggatacgaacagtgcgagagcacgtgtcgtcgaacaggcactagaactcgcggagaagacgtatccgtcattacggac**

**Bal3A**  **cgggatacgaacagtgcgagagcacgtgtcgtcgaacaggcactagaactcgcggagaagacgtatccgtcattacggac**

**MexicoAA**  **cgggatacgaacagtgcgagagcacgtgtcgtcgaacaggcactagaactcgcggagaagacgtatccgtcattacggac**

**Street14A cgggatacgaacagtgcgagagcacgtgtcgtcgaacaggcactagaactcgcggagaagacgtatccgtcattacggac**

**Sea81-4A**  **cgggatacgaacagtgcgagagcacgtgtcgtcgaacaggcactagaactcgcggagaagacgtatccgtcattacggac**

**GauthierA**  **cgggatacgaacagtgcgagagcacgtgtcgtcgaacaggcactagaactcgcggagaagacgtatccgtcattacggac**

**SamoaDA**  **cgggatacgaacagtgcgagagcacgtgtcgtcgaacaggcactagaactcgcggagaagacgtatccgtcattacggac**

**CDC2A**  **cgggatacgaacagtgcgagagcacgtgtcgtcgaacaggcactagaactcgcggagaagacgtatccgtcattacggac**

**BosniaAA**  **cgggatacgaacagtgcgagagcacgtgtcgtcgaacaggcactagaactcgcggagaagacgtatccgtcattacggac**

**IraqBA**  **cgggatacgaacagtgcgagagcacgtgtcgtcgaacaggcactagaactcgcggagaagacgtatccgtcattacggac**

**Fribourg-BlancA** **cgggatacgaacagtgcgagagcacgtgtcgtcgaacaggcactagaactcgcggagaagacgtatccgtcattacggac**

**CuniculiAA**  **cgggatacgaacagtgcgagagcacgtgtcgtcgaacaggcactaaaattcgcggagaagacgtatccgtcattacggac**

1050 1060 1070 1080 1090 1100 1110 1120

....|....|....|....|....|....|....|....|....|....|....|....|....|....|....|....|

**NicholsA**  **ggtgcgtcgtatattcagctggatggtacagcacgtggactcattaggcatagacgcgctggttacagcgcagtggcgtt**

**Bal3A**  **ggtgcgtcgtatattcagctggatggtacagcacgtggactcattaggcatagacgcgctggttacagcgcagtggcgtt**

**MexicoAA**  **ggtgcgtcgtatattcagctggatggtacagcacgtggactcattaggcatagacgcgctggttacagcgcagtggcgtt**

**Street14A ggtgcgtcgtatattcagctggatggtacagcacgtggactcattaggcatagacgcgctggttacagcgcagtggcgtt**

**Sea81-4A**  **ggtgcgtcgtatattcagctggatggtacagcacgtggactcattaggcatagacgcgctggttacagcgcagtggcgtt**

**GauthierA**  **ggtgcgtcgtatattcagctggatggtacagcacgtggactcattaggcatagacgcgctggttacagcgcagtggcgtt**

**SamoaDA**  **ggtgcgtcgtatattcagctggatggtacagcacgtggactcattaggcatagacgcgctggttacagcgcagtggcgtt**

**CDC2A**  **ggtgcgtcgtatattcagctggatggtacagcacgtggactcattaggcatagacgcgctggttacagcgcagtggcgtt**

**BosniaAA**  **ggtgcgtcgtatattcagctggatggtacagcacgtggactcattaggcatagacgcgctggttacagcgcagtggcgtt**

**IraqBA**  **ggtgcgtcgtatattcagctggatggtacagcacgtggactcattaggcatagacgcgctggttacagcgcagtggcgtt**

**Fribourg-BlancA** **ggtgcgtcgtatattcagctggatggtacagcacgtggactcattaggcatagacgcgctggttacagcgcagtggcgtt**

**CuniculiAA**  **ggtgcgtcgtatattcagctggatggtacagcacgtggactcattaggcatagacgcgctggttacagcgcagtggcgtt**

1130 1140 1150 1160 1170 1180 1190 1200

....|....|....|....|....|....|....|....|....|....|....|....|....|....|....|....|

**NicholsA**  **ggctttcaggaggtgtatacggcgcaacaggggcggcgtctgtttttgggagtggtccctttgtaaagtcaacttttcaa**

**Bal3A**  **ggctttcaggaggtgtatacggcgcaacaggggcggcgtctgtttttgggagtggtccctttgtaaagtcaacttttcaa**

**MexicoAA**  **ggctttcaggaggtgtatacggcgcaacaggggcggcgtctgtttttgggagtggtccctttgtaaagtcaacttttcaa**

**Street14A ggctttcaggaggtgtatacggcgcaacaggggcggcgtctgtttttgggagtggtccctttgtaaagtcaacttttcaa**

**Sea81-4A**  **ggctttcaggaggtgtatacggcgcaacaggggcggcgtctgtttttgggagtggtccctttgtaaagtcaacttttcaa**

**GauthierA**  **ggctttcaggaggtgtatacggcgcaacaggggcggcgtctgtttttgggagtggtccctttgtaaagtcaacttttcaa**

**SamoaDA**  **ggctttcaggaggtgtatacggcgcaacaggggcggcgtctgtttttgggagtggtccctttgtaaagtcaacttttcaa**

**CDC2A**  **ggctttcaggaggtgtatacggcgcaacaggggcggcgtctgtttttgggagtggtccctttgtaaagtcaacttttcaa**

**BosniaAA**  **ggctttcaggaggtgtatacggcgcaacaggggcggcgtctgtttttgggagtggtccctttgtaaagtcaacttttcaa**

**IraqBA**  **ggctttcaggaggtgtatacggcgcaacaggggcggcgtctgtttttgggagtggtccctttgtaaagtcaacttttcaa**

**Fribourg-BlancA** **ggctttcaggaggtgtatacggcgcaacaggggcggcgtctgtttttgggagtggtccctttgtaaagtcaacttttcaa**

**CuniculiAA**  **ggctttcaggaggtgtatacggcgcaacaggggcggcgtctgtttttgggagtagtccctttgtaaattcaacttttcaa**

1210 1220 1230 1240 1250 1260 1270 1280

....|....|....|....|....|....|....|....|....|....|....|....|....|....|....|....|

**NicholsA**  **tacacggactttgctgcgtttctcagactagaaactcgttcgggagatgattacacccatgccttgcacggcctaaacgc**

**Bal3A**  **tacacggactttgctgcgtttctcagactagaaactcgttcgggagatgattacacccatgccttgcacggcctaaacgc**

**MexicoAA**  **tacacggactttgctgcgtttctcagactagaaactcgttcgggagatgattacacccatgccttgcacggcctaaacgc**

**Street14A tacacggactttgctgcgtttctcagactagaaactcgttcgggagatgattacacccatgccttgcacggcctaaacgc**

**Sea81-4A**  **tacacggactttgctgcgtttctcagactagaaactcgttcgggagatgattacacccatgccttgcacggcctaaacgc**

**GauthierA**  **tacacggactttgctgcgtttctcagactagaaactcgttcgggagatgattacacccatgccttgcacggcctaaacgc**

**SamoaDA**  **tacacggactttgctgcgtttctcagactagaaactcgttcgggagatgattacacccatgccttgcacggcctaaacgc**

**CDC2A**  **tacacggactttgctgcgtttctcagactagaaactcgttcgggagatgattacacccatgccttgcacggcctaaacgc**

**BosniaAA**  **tacacggactttgctgcgtttctcagactagaaactcgttcgggagatgattacacccatgccttgcacggcctaaacgc**

**IraqBA**  **tacacggactttgctgcgtttctcagactagaaactcgttcgggagatgattacacccatgccttgcacggcctaaacgc**

**Fribourg-BlancA** **tacacggactttgctgcgtttctcagactagaaactcgttcgggagatgattacacccatgccttgcacggcctaaacgc**

**CuniculiAA**  **tacacggactttgctgcgtttctcagactagaaactcgttcgggagatgattacacccatgccttgcatggcctaaacgc**

1290 1300 1310 1320 1330 1340 1350 1360

....|....|....|....|....|....|....|....|....|....|....|....|....|....|....|....|

**NicholsA**  **aggcatagaagcacgtgtataccttcccctgggctacaaaagttatcttgacaacggaggtctccccccagacgtcgtac**

**Bal3A**  **aggcatagaagcacgtgtataccttcccctgggctacaaaagttatcttgacaacggaggtctccccccagacgtcgtac**

**MexicoAA**  **aggcatagaagcacgtgtataccttcccctgggctacaaaagttatcttgacaacggaggtctccccccagacgtcgtac**

**Street14A aggcatagaagcacgtgtataccttcccctgggctacaaaagttatcttgacaacggaggtctccccccagacgtcgtac**

**Sea81-4A**  **aggcatagaagcacgtgtataccttcccctgggctacaaaagttatcttgacaacgaaggtctccccccagacgtcgtac**

**GauthierA**  **aggcatagaagcacgtgtataccttcccctggactacaaaagttatcttgacaacggaggtctccccccagacgtcgtac**

**SamoaDA**  **aggcatagaagcacgtgtataccttcccctggactacaaaagttatcttgacaacggaggtctccccccagacgtcgtac**

**CDC2A**  **aggcatagaagcacgtgtataccttcccctggactacaaaagttatcttgacaacggaggtctccccccagacgtcgtac**

**BosniaAA**  **aggcatagaagcacgtgtataccttcccctggactacaaaagttatcttgacaacggaggtctccccccagacgtcgtac**

**IraqBA**  **aggcatagaagcacgtgtataccttcccctggactacaaaagttatcttgacaacggaggtctccccccagacgtcgtac**

**Fribourg-BlancA** **aggcatagaagcacgtgtataccttcccctggactacaaaagttatcttgacaacggaggtctccccccagacgtcgtac**

**CuniculiAA**  **aggcatagaagcacgtgtataccttcccctgacctacaaaagttaccttgacaacggaggtctccccccagacgtcgtac**

1370 1380 1390 1400 1410 1420 1430 1440

....|....|....|....|....|....|....|....|....|....|....|....|....|....|....|....|

**NicholsA**  **ctgcccaaggggtagcacagcaggtcgccaatgctgccagagtaggtattttaggtcttcttaatttcttgaatagtgcc**

**Bal3A**  **ctgcccaaggggtagcacagcaggtcgccaatgctgccagagtaggtattttaggtcttcttaatttcttgaatagtgcc**

**MexicoAA**  **ctgcccaaggggtagcacagcaggtcgccaatgctgccagagtaggtattttaggtcttcttaatttcttgaatagtgcc**

**Street14A ctgcccaaggggtagcacagcaggtcgccaatgctgccagagtaggtattttaggtcttcttaatttcttgaatagtgcc**

**Sea81-4A**  **ctgcccaaggggtagcacagcaggtcgccaatgctgccagagtaggtattttaggtcttcttaatttcttgaatagtgcc**

**GauthierA**  **ctgcccaaggggtagcacagcaggtcgccaatgctgccagagtaggtaytttaggtcttcttaatttcttgaatagtgcc**

**SamoaDA**  **ctgcccaaggggtagcacagcaggtcgccaatgctgccagagtaggtattttaggtcttcttaatttcttgaatagtgcc**

**CDC2A**  **ctgcccaaggggtagcacagcaggtcgccaatgctgccagagtaggtattttaggtcttcttaatttcttgaatagtgcc**

**BosniaAA**  **ctgcccaaggggtagcacagcaggtcgccaatgctgccagagtaggtattttaggtcttcttaatttcttgaatagtgcc**

**IraqBA**  **ctgcccaaggggtagcacagcaggtcgccaatgctgccagagtaggtattttaggtcttcttaatttcttgaatagtgcc**

**Fribourg-BlancA** **ctgcccaaggggtagcacagcaggtcgccaatgctgccagagtaggtattttaggtcttcttaatttcttgaatagtgcc**

**CuniculiAA**  **ctgcccgaggggtagcacagcaggtcgccaatattttcgaagcaggtcttcgcaatatttttggtttttttaacagagcc**

1450 1460 1470 1480 1490 1500 1510 1520

....|....|....|....|....|....|....|....|....|....|....|....|....|....|....|....|

**NicholsA**  **acaaactttgtagcacgcgcggcagggttaccggcagacgcgatagcaacaggcgcagcttcaatagcccttccgataat**

**Bal3A**  **acaaactttgtagcacgcgcggcagggttaccggcagacgcgatagcaacaggcgcagcttcaatagcccttccgataat**

**MexicoAA**  **acaaactttgtagcacgcgcggcagggttaccggcagacgcgatagcaacaggcgcagcttcaatagcccttccgataat**

**Street14A acaaactttgtagcacgcgcggcagggttaccggcagacgcgatagcaacaggcgcagcttcaatagcccttccgataat**

**Sea81-4A**  **acaaactttgtagcacgcgcggcagggttaccggcagacgcgatagcaacaggcgcagcttcaatagcccttccgataat**

**GauthierA**  **acaaactttgtagcacgcgcggcagggttaccggcagacgcgatagcaacaggcgcagcttcaatagcccttccgataat**

**SamoaDA**  **acaaactttgtagcacgcgcggcagggttaccggcagacgcgatagcaacaggcgcagcttcaatagcccttccgataat**

**CDC2A**  **acaaactttgtagcacgcgcggcagggttaccggcagacgcgatagcaacaggcgcagcttcaatagcccttccgataat**

**BosniaAA**  **acaaactttgtagcacgcgcggcagggttaccggcagacgcgatagcaacaggcgcagcttcaatagcccttccgataat**

**IraqBA**  **acaaactttgtagcacgcgcggcagggttaccggcagacgcgatagcaacaggcgcagcttcaatagcccttccgataat**

**Fribourg-BlancA** **acaaactttgtagcacgcgcggcagggttaccggcagacgcgatagcaacaggcgcagcttcaatagcccttccgataat**

**CuniculiAA**  **gtaaactttgtagcacgcgcggcagggttaccggcagacgcgataacaacaggcgcagcttcaatagcccttccgataat**

1530 1540 1550 1560 1570 1580 1590 1600

....|....|....|....|....|....|....|....|....|....|....|....|....|....|....|....|

**NicholsA**  **ggggaatgcatgggtaggctaccgcataccgtgctacgatagtatgtggatagaaccacgtgcgcacatatatatggcaa**

**Bal3A**  **ggggaatgcatgggtaggctaccgcataccgtgctacgatagtatgtggatagaaccacgtgcgcacatatatatggcaa**

**MexicoAA**  **ggggaatgcatgggtaggctaccgcataccgtgctacgatagtatgtggatagaaccacgtgcgcacatatatatggcaa**

**Street14A ggggaatgcatgggtaggctaccgcataccgtgctacgatagtatgtggatagaaccacgtgcgcacatatatatggcaa**

**Sea81-4A**  **ggggaatgcatgggtaggctaccgcataccgtgctacgatagtatgtggatagaaccacgtgcgcacatatatatggcaa**

**GauthierA**  **ggggaatgcatgggtaggctaccgcataccgtgctacgatagtatgtggatagaaccacgtgcgcacatatatatggcaa**

**SamoaDA**  **ggggaatgcatgggtaggctaccgcataccgtgctacgatagtatgtggatagaaccacgtgcgcacatatatatggcaa**

**CDC2A**  **ggggaatgcatgggtaggctaccgcataccgtgctacgatagtatgtggatagaaccacgtgcgcacatatatatggcaa**

**BosniaAA**  **ggggaatgcatgggtaggctaccgcataccgtgctacgatagtatgtggatagaaccacgtgcgcacatatatatggcaa**

**IraqBA**  **ggggaatgcatgggtaggctaccgcataccgtgctacgatagtatgtggatagaaccacgtgcgcacatatatatggcaa**

**Fribourg-BlancA** **ggggaatgcatgggtaggctaccgcataccgtgctacgatagtatgtggatagaaccacgtgcgcacatatatatggcaa**

**CuniculiAA**  **ggggaatgcatgggtaggctaccgcataccatgctacgacagtatgtggatagaaccacgtgcgcacatatatatggcaa**

1610 1620 1630 1640 1650 1660 1670 1680

....|....|....|....|....|....|....|....|....|....|....|....|....|....|....|....|

**NicholsA**  **ccaaccgattcaatttcaatgggttcaaaggaacgcgcaatttaaagggagaactttgtttccaatacgctgttgagctg**

**Bal3A**  **ccaaccgattcaatttcaatgggttcaaaggaacgcgcaatttaaagggagaactttgtttccaatacgctgttgagctg**

**MexicoAA**  **ccaaccgattcaatttcaatgggttcaaaggaacgcgcaatttaaagggagaactttgtttccaatacgctgttgagctg**

**Street14A ccaaccgattcaatttcaatgggttcaaaggaacgcgcaatttaaagggagaactttgtttccaatacgctgttgagctg**

**Sea81-4A**  **ccaaccgattcaatttcaatgggttcaaaggaacgcgcaatttaaagggagaactttgtttccaatacgctgttgagttg**

**GauthierA**  **ccaaccgattcaatttcaatgggttcaaaggaacgcgcaatttaaagggagaactttgtttccaatacgctgttgagctg**

**SamoaDA**  **ccaaccgattcaatttcaatgggttcaaaggaacgcgcaatttaaagggagaactttgtttccaatacgctgttgagctg**

**CDC2A**  **ccaaccgattcaatttcaatgggttcaaaggaacgcgcaatttaaagggagaactttgtttccaatacgctgttgagctg**

**BosniaAA**  **ccaaccgattcaatttcaatgggttcaaaggaacgcgcaatttaaagggagaactttgtttccaatacgctgttgagctg**

**IraqBA**  **ccaaccgattcaatttcaatgggttcaaaggaacgcgcaatttaaagggagaactttgtttccaatacgctgttgagctg**

**Fribourg-BlancA** **ccaaccgattcaatttcaatgggttcaaaggaacgcgcaatttaaagggagaactttgtttccaatacgctgttgagctg**

**CuniculiAA**  **ccaaccgattcaatttcaatgggttcaaaggaacgcacaatttaaagggagaactttgtttccaatacgctgttgagctg**

1690 1700 1710 1720 1730 1740 1750 1760

....|....|....|....|....|....|....|....|....|....|....|....|....|....|....|....|

**NicholsA**  **cgtgcaagtcctatcaaacctgtggagttttccgtgcggtgggagcaagggttgctttcacaggatccctacatgttgat**

**Bal3A**  **cgtgcaagtcctatcaaacctgtggagttttccgtgcggtgggagcaagggttgctttcacaggatccctacatgttgat**

**MexicoAA**  **cgtgcaagtcctatcaaacctgtggagttttccgtgcggtgggagcaagggttgctttcacaggatccctacatgttgat**

**Street14A cgtgcaagtcctatcaaacctgtggagttttccgtgcggtgggagcaagggttgctttcacaggatccctacatgttgat**

**Sea81-4A**  **cgtgcaagtcctatcaaacctgtggagttttccgtgcggtgggagcaagggttgctttcacaggatccctacatgttgat**

**GauthierA**  **cgtgcaagtcytatcaaacctgtggagttttccgtgcggtgggagcaagggttgctttcacaggatccctacatgttgat**

**SamoaDA**  **cgtgcaagtcctatcaaacctgtggagttttccgtgcggtgggagcaagggttgctttcacaggatccctacatgttgat**

**CDC2A**  **cgtgcaagtcctatcaaacctgtggagttttccgtgcggtgggagcaagggttgctttcacaggatccctacatgttgat**

**BosniaAA**  **cgtgcaagtcctatcaaacctgtggagttttccgtgcggtgggagcaagggttgctttcacaggatccctacatgttgat**

**IraqBA**  **cgtgcaagtcctatcaaacctgtggagttttccgtgcggtgggagcaagggttgctttcacaggatccctacatgttgat**

**Fribourg-BlancA** **cgtgcaagtcctatcaaacctgtggagttttccgtgcggtgggagcaagggttgctttcacaggatccctacatgttgat**

**CuniculiAA**  **catgctagtcctatcaaacatgtggagttttccgtgcggtgggagcaagggttgctttcacaggatccctacatgttgat**

1770 1780 1790 1800 1810 1820

....|....|....|....|....|....|....|....|....|....|....|....|....|..

**NicholsA**  **agaagagaactggagctggcctggatacaccggttcactctttctagggtgcaagatcacttggtga**

**Bal3A**  **agaagagaactggagctggcctggatacaccggttcactctttctagggtgcaagatcacttggtga**

**MexicoAA**  **agaagagaactggagctggcctggatacaccggttcactctttctagggtgcaagatcacttggtga**

**Street14A agaagagaactggagctggcctggatacaccggttcactctttctagggtgcaagatcacttggtga**

**Sea81-4A**  **agaagagaactggagctggcctggatacaccggttcactctttctagggtgcaagatcacttggtga**

**GauthierA**  **agaagagaactggagctggcctggatacaccggttcactctttctagggtgcaagatcacttgatga**

**SamoaDA**  **agaagagaactggagctggcctggatacaccggttcactctttctagggtgcaagatcacttgatga**

**CDC2A**  **agaagagaactggagctggcctggatacaccggttcactctttctagggtgcaagatcacttgatga**

**BosniaAA**  **agaagagaactggagctggcctggatacaccggttcactctttctagggtgcaagatcacttgatga**

**IraqBA**  **agaagagaactggagctggcctggatacaccggttcactctttctagggtgcaagatcacttgatga**

**Fribourg-BlancA** **agaagagaactggagctggcctggatacaccggttcactctttctagggtgcaagatcacttgatga**

**CuniculiAA**  **agaagagaactggagctggcctggatacaccggttcactctttctagagtgcaagatcacttggtga**

***1.2 tprB* Locus**

10 20 30 40 50 60 70 80

....|....|....|....|....|....|....|....|....|....|....|....|....|....|....|....|

**NicholsB**  **MGACISVYARFALGCGVFFLHGAVLDGVSRAFSSSAAFSGSAELSWGVVFDAEGASPVTAGKSIRHGFRTKSSWKLAFPL**

**Bal3B**  **MGACISVYARFALGCGVFFLHGAVLDGVSRAFSSSAAFSGSAELSWGVVFDAEGASPVTAGKSIRHGFRTKSSWKLAFPL**

**Sea81-4B**  **MGACISVYARFALGCGVFFLHGAVLDGVSRAFSSSAAFSGSAELSWGVVFDAEGASPVTAGKSIRHGFRTKSSWKLAFPL**

**MexicoAB**  **MGACISVYARFALGCGVFFLHGAVLDGVSRAFSSSAAFSGSAELSWGVVFDAEGASPVTAGKSIRHGFRTKSSWKLAFPL**

**Street14B MGACISVYARFALGCGVFFLHGAVLDGVSRAFSSSAAFSGSAELSWGVVFDAEGASPVTAGKSIRHGFRTKSSWKLAFPL**

**GauthierB**  **MGACISVYARFALGCGVFFLHGAVLDGVSRAFSSSAAFSGSAELSWGVVFDAEGASPVTAGKSIRHGFRTKSSWKLAFPL**

**SamoaDB**  **MGACISVYARFALGCGVFFLHGAVLDGVSRAFSSSAAFSGSAELSWGVVFDAEGASPVTAGKSIRHGFRTKSSWKLAFPL**

**CDC2B**  **MGACISVYARFALGCGVFFLHGAVLDGVSRAFSSSAAFSGSAELSWGVVFDAEGASPVTAGKSIRHGFRTKSSWKLAFPL**

**BosniaAB**  **MGACISVYARFALGCGVFFLHGAVLDGVSRAFSSSAAFSGSAELSWGVVFDAEGASPVTAGKSIRHGFRTKSSWKLAFPL**

**IraqBB**  **MGACISVYARFALGCGVFFLHGAVLDGVSRAFSSSAAFSGSAELSWGVVFDAEGASPVTAGKSIRHGFRTKSSWKLAFPL**

**Fribourg-BlancB** **MGACISVYARFALGCGVFFLHGAVLDGVSRAFSSSAAFSGSAELSWGVVFDAEGASPVTAGKSIRHGFRTKSSWKLAFPL**

**CuniculiAB**  **MGACISVYARFALGCGVFFLHGAVLDGVSRAFSSSAAFSGSAELSWGVVFDAEGASPVTAGKSIRHGFRTKSSWKLAFPL**

90 100 110 120 130 140 150 160

....|....|....|....|....|....|....|....|....|....|....|....|....|....|....|....|

**NicholsB**  **LPKKGATYTSFSGEDPIWVELSLKGLKVDFESALGSGTADPSMTTRSPFLKSGRSDFSLEATLHLYDVSFSVGKDPVFPS**

**Bal3B**  **LPKKGATYTSFSGEDPIWVELSLKGLKVDFESALGSGTADPSMTTRSPFLKSGRSDFSLEATLHLYDVSFSVGKDPVFPS**

**Sea81-4B**  **LPKKGATYTSFSGEDPIWVELSLKGLKVDFESALGSGTADPSMTTRSPFLKSGRSDFSLEATLHLYDVSFSVGKDPVFPS**

**MexicoAB**  **LPKKGATYTSFSGEDPIWVELSLKGLKVDFESALGSGTADPSMTTRSPFLKSGRSDFSLEATLHLYDVSFSVGKDPVFPS**

**Street14B LPKKGATYTSFSGEDPIWVELSLKGLKVDFESALGSGTADPSMTTRSPFLKSGRSDFSLEATLHLYDVSFSVGKDPVFPS**

**GauthierB**  **LPKKGATYTSFSGEDPIWVELSLKGLKVDFESALGSGTADPSMTTRSPFLKSGRSDFSLEATLHLYDVSFSVGKDPVFPS**

**SamoaDB**  **LPKKGATYTSFSGEDPIWVELSLKGLKVDFESALGSGTADPSMTTRSPFLKSGRSDFSLEATLHLYDVSFSVGKDPVFPS**

**CDC2B**  **LPKKGATYTSFSGEDPIWVELSLKGLKVDFESALGSGTADPSMTTRSPFLKSGRSDFSLEATLHLYDVSFSVGKDPVFPS**

**BosniaAB**  **LPKKGATYTSFSGEDPIWVELSLKGLKVDFESALGSGTADPSMTTRSPFLKSGRSDFSLEATLHLYDVSFSVGKDPVFPS**

**IraqBB**  **LPKKGATYTSFSGEDPIWVELSLKGLKVDFESALGSGTADPSMTTRSPFLKSGRSDFSLEATLHLYDVSFSVGKDPVFPS**

**Fribourg-BlancB** **LPKKGATYTSFSGEDPIWVELSLKGLKVDFESALGSGTADPSMTTRSPFLKSGRSDFSLEATLHLYDVSFSVGKDPVFPS**

**CuniculiAB**  **LPKKGATYTSFSGEDPIWVELSLKGLKVDFESALGSGTADPSMTTRSPFLKSRRSDFSLEATLHLYDVSFSVGKDPVFPS**

170 180 190 200 210 220 230 240

....|....|....|....|....|....|....|....|....|....|....|....|....|....|....|....|

**NicholsB**  **NFAQLWTPFITTSYESRSVKYAPGFGGVGGKIAYQARNISNSGITFNCALSFSSNGIWKSAPSVTSKVKGKGTNSRRMPA**

**Bal3B**  **NFAQLWTPFITTSYESRSVKYAPGFGGVGGKIAYQARNISNSGITFNCALSFSSNGIWKSAPSVTSKVKGKGTNSRRMPA**

**Sea81-4B**  **NFAQLWTPFITTSYESRSVKYAPGFGGVGGKIAYQARNISNSGITFNCALSFSSNGIWKSAPSVTSKVKGKGTNSRRMPA**

**MexicoAB**  **NFAQLWTPFITTSYESRSVKYAPGFGGVGGKIAYQARNISNSGITFNCALSFSSNGIWKSAPSVTSKVKGKGTNSRRMPA**

**Street14B NFAQLWTPFITTSYESRSVKYAPGFGGVGGKIAYQARNISNSGITFNCALSFSSNGIWKSAPSVTSKVKGKGTNSRRMPA**

**GauthierB**  **NFAQLWTPFITTSYESRSVKYAPGFGGVGGKIAYQARNISNSGITFNCALSFSSNGIWKSAPSVTSKVKGKGTNSRRMPA**

**SamoaDB**  **NFAQLWTPFITTSYESRSVKYAPGFGGVGGKIAYQARNISNSGITFNCALSFSSNGIWKSAPSVTSKVKGKGTNSRRMPA**

**CDC2B**  **NFAQLWTPFITTSYESRSVKYAPGFGGVGGKIAYQARNISNSGITFNCALSFSSNGIWKSAPSVTSKVKGKGTNSRRMPA**

**BosniaAB**  **NFAQLWTPFITTSYESRSVKYAPGFGGVGGKIAYQARNISNSGITFNCALSFSSNGIWKSAPSVTSKVKGKGTNSRRMPA**

**IraqBB**  **NFAQLWTPFITTSYESRSVKYAPGFGGVGGKIAYQARNISNSGITFNCALSFSSNGIWKSAPSVTSKVKGKGTNSRRMPA**

**Fribourg-BlancB** **NFAQLWTPFITTSYESRSVKYAPGFGGVGGKIAYQARNISNSGITFNCALSFSSNGIWKSAPSVTSKVKGKGTNSRRMPA**

**CuniculiAB**  **NFAQLWTPFITTSYESRSVKYAPGFGGVGGKIAYQARNISNSGITFNCALSFSSNGIWKSAPSVTSKVKGKGTNSRRMPA**

250 260 270 280 290 300 310 320

....|....|....|....|....|....|....|....|....|....|....|....|....|....|....|....|

**NicholsB**  **DPHSKYGLGTEFTLVYARKGREQVRLEAASCATLSAGYRTGPDQTHHQNKDTVLWNVGARLTLSPGAGFKIVCAFDAGTP**

**Bal3B**  **DPHSKYGLGTEFTLVYARKGREQVRLEAASCATLSAGYRTGPDQTHHQNKDTVLWNVGARLTLSPGAGFKIVCAFDAGTP**

**Sea81-4B**  **DPHSKYGLGTEFTLVYARKGREQVRLEAASCATLSAGYRTGPDQTHHQNKDTVLWNVGARLTLSPGAGFKIVCAFDAGTP**

**MexicoAB**  **DPHSKYGLGTEFTLVYARKGREQVRLEAASCATLSAGYRTGPDQTHHQNKDTVLWNVGARLTLSPGAGFKIVCAFDAGTP**

**Street14B DPHSKYGLGTEFTLVYARKGREQVRLEAASCATLSAGYRTGPDQTHHQNKDTVLWNVGARLTLSPGAGFKIVCAFDAGTP**

**GauthierB**  **DPHSKYGLGTEFTLVYARKGREQVRLEAASCATLSAGYRTGPDQTHHQNKDTVLWNVGARLTLSPGAGFKIVCAFDAGTP**

**SamoaDB**  **DPHSKYGLGTEFTLVYARKGREQVRLEAASCATLSAGYRTGPDQTHHQNKDTVLWNVGARLTLSPGAGFKIVCAFDAGTP**

**CDC2B**  **DPHSKYGLGTEFTLVYARKGREQVRLEAASCATLSAGYRTGPDQTHHQNKDTVLWNVGARLTLSPGAGFKIVCAFDAGTP**

**BosniaAB**  **DPHSKYGLGTEFTLVYARKGREQVRLEAASCATLSAGYRTGPDQTHHQNKDTVLWNVGARLTLSPGAGFKIVCAFDAGTP**

**IraqBB**  **DPHSKYGLGTEFTLVYARKGREQVRLEAASCATLSAGYRTGPDQTHHQNKDTVLWNVGARLTLSPGAGFKIVCAFDAGTP**

**Fribourg-BlancB** **DPHSKYGLGTEFTLVYARKGREQVRLEAASCATLSAGYRTGPDQTHHQNKDTVLWNVGARLTLSPGAGFKIVCAFDAGTP**

**CuniculiAB**  **DPHSKYGLGTEFTLVYARKGREQVRLEAASCATLSAGYRTGPDQTHHQNKDTVLWNVGARLTLSPGAGFKIVCAFDAGTP**

330 340 350 360 370 380 390 400

....|....|....|....|....|....|....|....|....|....|....|....|....|....|....|....|

**NicholsB**  **YKKGAARESLAETLAAQRGCNRFDTALMHALGLLVAAAKTRNELAAQMRSQSPPGVWEKFEQAVQSLPPITQGKPGVVGA**

**Bal3B**  **YKKGAARESLAETLAAQRGCNRFDTALMHALGLLVAAAKTRNELAAQMRSQSPPGVWEKFEQAVQSLPPITQGKPGVVGA**

**Sea81-4B**  **YKKGAARESLAETLAAQRGCNRFDTALMHALGLLVAAAKTRNELAAQMRSQSPPGVWEKFEQAVQSLPPITQGKPGVVGA**

**MexicoAB**  **YKKGAARESLAETLAAQRGCNRFDTALMHALGLLVAAAKTRNELAAQMRSQSPPGVWEKFEQAVQSLPPITQGKPGVVGA**

**Street14B YKKGAARESLAETLAAQRGCNRFDTALMHALGLLVAAAKTRNELAAQMRSQSPPGVWEKFEQAVQSLPPITQGKPGVVGA**

**GauthierB**  **YKKGAARESLAETLAAQRGCNRFDTALMHALGLLVAAAKTRNELAAQMRSQSPPGVWEKFEQAVQSLPPITQGKPGVVGA**

**SamoaDB**  **YKKGAARESLAETLAAQRGCNRFDTALMHALGLLVAAAKTRNELAAQMRSQSPPGVWEKFEQAVQSLPPITQGKPGVVGA**

**CDC2B**  **YKKGAARESLAETLAAQRGCNRFDTALMHALGLLVAAAKTRNELAAQMRSQSPPGVWEKFEQAVQSLPPITQGKPGVVGA**

**BosniaAB**  **YKKGAARESLAETLAAQRGCNRFDTALMHALGLLVAAAKTRNELAAQMRSQSPPGVWEKFEQAVQSLPPITQGKPGVVGA**

**IraqBB**  **YKKGAARESLAETLAAQRGCNRFDTALMHALGLLVAAAKTRNELAAQMRSQSPPGVWEKFEQAVQSLPPITQGKPGVVGA**

**Fribourg-BlancB** **YKKGAARESLAETLAAQRGCNRFDTALMHALGLLVAAAKTRNELAAQMRSQSPPGVWEKFEQAVQSLPPITQGKPGVVGA**

**CuniculiAB**  **YKKGAARESLAETLAAQRGCNRFDTALMHALGLLVAAAKTRNELAAQMRSQSPPGVWEKFEQAVQSLPPITQGKPGVVGA**

410 420 430 440 450 460 470 480

....|....|....|....|....|....|....|....|....|....|....|....|....|....|....|....|

**NicholsB**  **EVRPGTMWMELSPVRKALVDVLSVLEQGGFDRVAFDALLIVQWRWISLGAYVASAPTNVFGSMLFPRGSSDHFDCAAFVR**

**Bal3B**  **EVRPGTMWMELSPVRKALVDVLSVLEQGGFDRVAFDALLIVQWRWISLGAYVASAPTNVFGSMLFPRGSSDHFDCAAFVR**

**Sea81-4B**  **EVRPGTMWMELSPVRKALVDVLSVLEQGGFDRVAFDALLIVQWRWISLGAYVASAPTNVFGSMLFPRGSSDHFDCAAFVR**

**MexicoAB**  **EVRPGTMWMELSPVRKALVDVLSVLEQGGFDRVAFDALLIVQWRWISLGAYVASAPTNVFGSMLFPRGSSDHFDCAAFVR**

**Street14B EVRPGTMWMELSPVRKALVDVLSVLEQGGFDRVAFDALLIVQWRWISLGAYVASAPTNVFGSMLFPRGSSDHFDCAAFVR**

**GauthierB**  **EVRPGTMWMELSPVRKALVDVLSVLEQGGFDRVAFDALLIVQWRWISLGAYVASAPTNVFGSMLFPRGSSDHFDCAAFVR**

**SamoaDB**  **EVRPGTMWMELSPVRKALVDVLSVLEQGGFDRVAFDALLIVQWRWISLGAYVASAPTNVFGSMLFPRGSSDHFDCAAFVR**

**CDC2B**  **EVRPGTMWMELSPVRKALVDVLSVLEQGGFDRVAFDALLIVQWRWISLGAYVASAPTNVFGSMLFPRGSSDHFDCAAFVR**

**BosniaAB**  **EVRPGTMWMELSPVRKALVDVLSVLEQGGFDRVAFDALLIVQWRWISLGAYVASAPTNVFGSMLFPRGSSDHFDCAAFVR**

**IraqBB**  **EVRPGTMWMELSPVRKALVDVLSVLEQGGFDRVAFDALLIVQWRWISLGAYVASAPTNVFGSMLFPRGSSDHFDCAAFVR**

**Fribourg-BlancB** **EVRPGTMWMELSPVRKALVDVLSVLEQGGFDRVAFDALLIVQWRWISLGAYVASAPTNVFGSMLFPRGSSDHFDCAAFVR**

**CuniculiAB**  **EVRPGTMWMELSPVRKALVDVLSVLEQGGFDRVAFDALLIVQWRWISLGAYVASASTNVFGSMLFPRGSSDHFDCAAFVR**

490 500 510 520 530 540 550 560

....|....|....|....|....|....|....|....|....|....|....|....|....|....|....|....|

**NicholsB**  **VESKWYDSLSKLVSGLSGGVEARLYIPFTHGLYLEPGSCTPNSTRGKKPQAFVLPPPGVVHPGAHSALPVVGKLWLNYRI**

**Bal3B**  **VESKWYDSLSKLVSGLSGGVEARLYIPFTHGLYLEPGSCTPNSTRGKKPQAFVLPPPGVVHPGAHSALPVVGKLWLNYRI**

**Sea81-4B**  **VESKWYDSLSKLVSGLSGGVEARLYIPFTHGLYLEPGSCTPNSTRGKKPQAFVLPPPGVVHPGAHSALPVVGKLWLNYRI**

**MexicoAB**  **VESKWYDSLSKLVSGLSGGVEARLYIPFTHGLYLEPGSCTPNSTRGKKPQAFVLPPPGVVHPGAHSALPVVGKLWLNYRI**

**Street14B VESKWYDSLSKLVSGLSGGVEARLYIPFTHGLYLEPGSCTPNSTRGKKPQAFVLPPPGVVHPGAHSALPVVGKLWLNYRI**

**GauthierB**  **VESKWYDSLSKLVSGLSGGVEARLYIPFTHGLYLEPGSCTPNSTRGKKPQAFVLPPPGVVHPGAHSALPVVGKLWLNYRI**

**SamoaDB**  **VESKWYDSLSKLVSGLSGGVEARLYIPFTHGLYLEPGSCTPNSTRGKKPQAFVLPPPGVVHPGAHSALPVVGKLWLNYRI**

**CDC2B**  **VESKWYDSLSKLVSGLSGGVEARLYIPFTHGLYLEPGSCTPNSTRGKKPQAFVLPPPGVVHPGAHSALPVVGKLWLNYRI**

**BosniaAB**  **VESKWYDSLSKLVSGLSGGVEARLYIPFTHGLYLEPGSCTPNSTRGKKPQAFVLPPPGVVHPGAHSALPVVGKLWLNYRI**

**IraqBB**  **VESKWYDSLSKLVSGLSGGVEARLYIPFTHGLYLEPGSCTPNSTRGKKPQAFVLPPPGVVHPGAHSALPVVGKLWLNYRI**

**Fribourg-BlancB** **VESKWYDSLSKLVSGLSGGVEARLYIPFTHGLYLEPGSCTPNSTRGKKPQAFVLPPPGVVHPGAHSALPVVGKLWLNYRI**

**CuniculiAB**  **VESKWYDSLSKLVSGLSGGVEARLYIPFTHGLYLEPGSCTPNSTRGKKPQAFVLPPPGVVHPGAHSALPVVGKLWLNYRI**

570 580 590 600 610 620 630 640

....|....|....|....|....|....|....|....|....|....|....|....|....|....|....|....|

**NicholsB**  **TLAAHAWIRPMVSLYGATYGAQGFSYGPGGAAGTVKRNRTFRAGKNLYYQVGVAVSPFERCELLIEWSQGMLARRPYISL**

**Bal3B**  **TLAAHAWIRPMVSLYGATYGAQGFSYGPGGAAGTVKRNRTFRAGKNLYYQVGVAVSPFERCELLIEWSQGMLARRPYISL**

**Sea81-4B**  **TLAAHAWIRPMVSLYGATYGAQGFSYGPGGAAGTVKRNRTFRAGKNLYYQVGVAVSPFERCELLIEWSQGMLARRPYISL**

**MexicoAB**  **TLAAHAWIRPMVSLYGATYGAQGFSYGPGGAAGTVKRNRTFRAGKNLYYQVGVAVSPFERCELLIEWSQGMLARRPYISL**

**Street14B TLAAHAWIRPMVSLYGATYGAQGFSYGPGGAAGTVKRNRTFRAGKNLYYQVGVAVSPFERCELLIEWSQGMLARRPYISL**

**GauthierB**  **TLAAHAWIRPMVSLYGATYGAQGFSYGPGGAAGTVKRNRTFRAGKNLYYQVGVAVSPFERCELLIEWSQGMLARRPYISL**

**SamoaDB**  **TLAAHAWIRPMVSLYGATYGAQGFSYGPGGAAGTVKRNRTFRAGKNLYYQVGVAVSPFERCELLIEWSQGMLARRPYISL**

**CDC2B**  **TLAAHAWIRPMVSLYGATYGAQGFSYGPGGAAGTVKRNRTFRAGKNLYYQVGVAVSPFERCELLIEWSQGMLARRPYISL**

**BosniaAB**  **TLAAHAWIRPMVSLYGATYGAQGFSYGPGGAAGTVKRNRTFRAGKNLYYQVGVAVSPFERCELLIEWSQGMLARRPYISL**

**IraqBB**  **TLAAHAWIRPMVSLYGATYGAQGFSYGPGGAAGTVKRNRTFRAGKNLYYQVGVAVSPFERCELLIEWSQGMLARRPYISL**

**Fribourg-BlancB** **TLAAHAWIRPMVSLYGATYGAQGFSYGPGGAAGTVKRNRTFRAGKNLYYQVGVAVSPFERCELLIEWSQGMLARRPYISL**

**CuniculiAB**  **TLAAHAWIRPMVSLYGATYGAQGFSYGPGGAAGTVKRSRTFRAGKNLYYQVGVAVSPFERCELLIEWSQGMLARRPYISL**

650 660

....|....|....|....|.

**NicholsB**  **EHGSWPDKRSELVCSCKVLW**

**Bal3B**  **EHGSWPDKRSELVCSCKVLW**

**Sea81-4B**  **EHGSWPDKRSELVCSCKVLW**

**MexicoAB**  **EHGSWPDKRSELVCSCKVLW**

**Street14B EHGSWPDKRSELVCSCKVLW**

**GauthierB**  **EHGSWPDKRSELVCSCKVLW**

**SamoaDB**  **EHGSWPDKRSELVCSCKVLW**

**CDC2B**  **EHGSWPDKRSELVCSCKVLW**

**BosniaAB**  **EHGSWPDKRSELVCSCKVLW**

**IraqBB**  **EHGSWPDKRSELVCSCKVLW**

**Fribourg-BlancB** **EHGSWPDKRSELVCSCKVLW**

**CuniculiAB**  **EHGSWPNKRSELVCSCKVLW**

10 20 30 40 50 60 70 80

....|....|....|....|....|....|....|....|....|....|....|....|....|....|....|....|

**NicholsB**  **atgggcgcctgtatatccgtatatgcgcgttttgcgttagggtgtggggtgtttttccttcatggtgcggttttggacgg**

**Bal3B**  **atgggcgcctgtatatccgtatatgcgcgttttgcgttagggtgtggggtgtttttccttcatggtgcggttttggacgg**

**Sea81-4B**  **atgggcgcctgtatatccgtatatgcgcgttttgcgttagggtgtggggtgtttttccttcatggtgcggttttggacgg**

**MexicoAB**  **atgggcgcctgtatatccgtatatgcgcgttttgcgttagggtgtggggtgtttttccttcatggtgcggttttggacgg**

**Street14B atgggcgcctgtatatccgtatatgcgcgttttgcgttagggtgtggggtgtttttccttcatggtgcggttttggacgg**

**GauthierB**  **atgggcgcctgtatatccgtatatgcgcgttttgcgttagggtgtggggtgtttttccttcatggtgcggttttggacgg**

**SamoaDB**  **atgggcgcctgtatatccgtatatgcgcgttttgcgttagggtgtggggtgtttttccttcatggtgcggttttggacgg**

**CDC2B**  **atgggcgcctgtatatccgtatatgcgcgttttgcgttagggtgtggggtgtttttccttcatggtgcggttttggacgg**

**BosniaAB**  **atgggcgcctgtatatccgtatatgcgcgttttgcgttagggtgtggggtgtttttccttcatggtgcggttttggacgg**

**IraqBB**  **atgggcgcctgtatatccgtatatgcgcgttttgcgttagggtgtggggtgtttttccttcatggtgcggttttggacgg**

**Fribourg-BlancB** **atgggcgcctgtatatccgtatatgcgcgttttgcgttagggtgtggggtgtttttccttcatggtgcggttttggacgg**

**CuniculiAB**  **atgggcgcctgtatatccgtatatgcgcgttttgcgttagggtgtggggtgtttttccttcatggtgcggttttggacgg**

90 100 110 120 130 140 150 160

....|....|....|....|....|....|....|....|....|....|....|....|....|....|....|....|

**NicholsB**  **ggtttcacgcgccttttcgtcctccgccgcgttcagcggttctgctgaacttagctggggtgtcgtctttgatgcagaag**

**Bal3B**  **ggtttcacgcgccttttcgtcctccgccgcgttcagcggttctgctgaacttagctggggtgtcgtctttgatgcagaag**

**Sea81-4B**  **ggtttcacgcgccttttcgtcctccgccgcgttcagcggttctgctgaacttagctggggtgtcgtctttgatgcagaag**

**MexicoAB**  **ggtttcacgcgccttttcgtcctccgccgcgttcagcggttctgctgaacttagctggggtgtcgtctttgatgcagaag**

**Street14B ggtttcacgcgccttttcgtcctccgccgcgttcagcggttctgctgaacttagctggggtgtcgtctttgatgcagaag**

**GauthierB**  **ggtttcacgcgccttttcgtcctccgccgcgttcagcggttctgctgaacttagctggggtgtcgtctttgatgcagaag**

**SamoaDB**  **ggtttcacgcgccttttcgtcctccgccgcgttcagcggttctgctgaacttagctggggtgtcgtctttgatgcagaag**

**CDC2B**  **ggtttcacgcgccttttcgtcctccgccgcgttcagcggttctgctgaacttagctggggtgtcgtctttgatgcagaag**

**BosniaAB**  **ggtttcacgcgccttttcgtcctccgccgcgttcagcggttctgctgaacttagctggggtgtcgtctttgatgcagaag**

**IraqBB**  **ggtttcacgcgccttttcgtcctccgccgcgttcagcggttctgctgaacttagctggggtgtcgtctttgatgcagaag**

**Fribourg-BlancB** **ggtttcacgcgccttttcgtcctccgccgcgttcagcggttctgctgaacttagctggggtgtcgtctttgatgcagaag**

**CuniculiAB**  **ggtttcacgcgccttttcgtcctccgccgcgttcagcggttctgctgaacttagctggggtgtcgtctttgatgcagaag**

170 180 190 200 210 220 230 240

....|....|....|....|....|....|....|....|....|....|....|....|....|....|....|....|

**NicholsB**  **gtgcctctccagttacagcgggtaaaagcatacgacatgggtttcgcacgaagagcagctggaagcttgcttttcccttg**

**Bal3B**  **gtgcctctccagttacagcgggtaaaagcatacgacatgggtttcgcacgaagagcagctggaagcttgcttttcccttg**

**Sea81-4B**  **gtgcctctccagttacagcgggtaaaagcatacgacatgggtttcgcacgaagagcagctggaagcttgcttttcccttg**

**MexicoAB**  **gtgcctctccagttacagcgggtaaaagcatacgacatgggtttcgcacgaagagcagctggaagcttgcttttcccttg**

**Street14B gtgcctctccagttacagcgggtaaaagcatacgacatgggtttcgcacgaagagcagctggaagcttgcttttcccttg**

**GauthierB**  **gtgcctctccagttacagcgggtaaaagcatacgacatgggtttcgcacgaagagcagctggaagcttgcttttcccttg**

**SamoaDB**  **gtgcctctccagttacagcgggtaaaagcatacgacatgggtttcgcacgaagagcagctggaagcttgcttttcccttg**

**CDC2B**  **gtgcctctccagttacagcgggtaaaagcatacgacatgggtttcgcacgaagagcagctggaagcttgcttttcccttg**

**BosniaAB**  **gtgcctctccagttacagcgggtaaaagcatacgacatgggtttcgcacgaagagcagctggaagcttgcttttcccttg**

**IraqBB**  **gtgcctctccagttacagcgggtaaaagcatacgacatgggtttcgcacgaagagcagctggaagcttgcttttcccttg**

**Fribourg-BlancB** **gtgcctctccagttacagcgggtaaaagcatacgacatgggtttcgcacgaagagcagctggaagcttgcttttcccttg**

**CuniculiAB**  **gtgcctctccagttacagcgggtaaaagcatacgacatgggtttcgcacgaagagtagctggaagcttgcttttcccttg**

250 260 270 280 290 300 310 320

....|....|....|....|....|....|....|....|....|....|....|....|....|....|....|....|

**NicholsB**  **ttgcccaagaaaggcgccacgtatacgagcttttcaggtgaggatcccatatgggttgagctttctctcaagggattgaa**

**Bal3B**  **ttgcccaagaaaggcgccacgtatacgagcttttcaggtgaggatcccatatgggttgagctttctctcaagggattgaa**

**Sea81-4B**  **ttgcccaagaaaggcgccacgtatacgagcttttcaggtgaggatcccatatgggttgagctttctctcaagggattgaa**

**MexicoAB**  **ttgcccaagaaaggcgccacgtatacgagcttttcaggtgaggatcccatatgggttgagctttctctcaagggattgaa**

**Street14B ttgcccaagaaaggcgccacgtatacgagcttttcaggtgaggatcccatatgggttgagctttctctcaagggattgaa**

**GauthierB**  **ttgcccaagaaaggcgccacgtatacgagcttttcaggtgaggatcccatatgggttgagctttctctcaagggattgaa**

**SamoaDB**  **ttgcccaagaaaggcgccacgtatacgagcttttcaggtgaggatcccatatgggttgagctttctctcaagggattgaa**

**CDC2B**  **ttgcccaagaaaggcgccacgtatacgagcttttcaggtgaggatcccatatgggttgagctttctctcaagggattgaa**

**BosniaAB**  **ttgcccaagaaaggcgccacgtatacgagcttttcaggtgaggatcccatatgggttgagctttctctcaagggattgaa**

**IraqBB**  **ttgcccaagaaaggcgccacgtatacgagcttttcaggtgaggatcccatatgggttgagctttctctcaagggattgaa**

**Fribourg-BlancB** **ttgcccaagaaaggcgccacgtatacgagcttttcaggtgaggatcccatatgggttgagctttctctcaagggattgaa**

**CuniculiAB**  **ttgcccaagaaaggcgccacgtatacgagcttttcaggtgaggatcccatatgggttgagctttctctcaagggattgaa**

330 340 350 360 370 380 390 400

....|....|....|....|....|....|....|....|....|....|....|....|....|....|....|....|

**NicholsB**  **ggtggattttgaaagtgctttagggtcgggaactgcggatccaagtatgacgacgcgttctcctttcttaaagtcaggaa**

**Bal3B**  **ggtggattttgaaagtgctttagggtcgggaactgcggatccaagtatgacgacgcgttctcctttcttaaagtcaggaa**

**Sea81-4B**  **ggtggattttgaaagtgctttagggtcgggaactgcggatccaagtatgacgacgcgttctcctttcttaaagtcaggaa**

**MexicoAB**  **ggtggattttgaaagtgctttagggtcgggaactgcggatccaagtatgacgacgcgttctcctttcttaaagtcaggaa**

**Street14B ggtggattttgaaagtgctttagggtcgggaactgcggatccaagtatgacgacgcgttctcctttcttaaagtcaggaa**

**GauthierB**  **ggtggattttgaaagtgctttagggtcgggaactgcggatccaagtatgacgacgcgttctcctttcttaaagtcaggaa**

**SamoaDB**  **ggtggattttgaaagtgctttagggtcgggaactgcggatccaagtatgacgacgcgttctcctttcttaaagtcaggaa**

**CDC2B**  **ggtggattttgaaagtgctttagggtcgggaactgcggatccaagtatgacgacgcgttctcctttcttaaagtcaggaa**

**BosniaAB**  **ggtggattttgaaagtgctttagggtcgggaactgcggatccaagtatgacgacgcgttctcctttcttaaagtcaggaa**

**IraqBB**  **ggtggattttgaaagtgctttagggtcgggaactgcggatccaagtatgacgacgcgttctcctttcttaaagtcaggaa**

**Fribourg-BlancB** **ggtggattttgaaagtgctttagggtcgggaactgcggatccaagtatgacgacgcgttctcctttcttaaagtcaggaa**

**CuniculiAB**  **ggtggattttgaaagtgctttagggtcgggaactgcggatccaagtatgacgacgcgttctcctttcttaaagtcaagaa**

410 420 430 440 450 460 470 480

....|....|....|....|....|....|....|....|....|....|....|....|....|....|....|....|

**NicholsB**  **gaagcgatttttcccttgaggccacactccacctctacgatgtctctttttctgtaggaaaagatcccgtttttccctct**

**Bal3B**  **gaagcgatttttcccttgaggccacactccacctctacgatgtctctttttctgtaggaaaagatcccgtttttccctct**

**Sea81-4B**  **gaagcgatttttcccttgaggccacactccacctctacgatgtctctttttctgtaggaaaagatcccgtttttccctct**

**MexicoAB**  **gaagcgatttttcccttgaggccacactccacctctacgatgtctctttttctgtaggaaaagatcccgtttttccctct**

**Street14B gaagcgatttttcccttgaggccacactccacctctacgatgtctctttttctgtaggaaaagatcccgtttttccctct**

**GauthierB**  **gaagcgatttttcccttgaggccacactccacctctacgatgtctctttttctgtaggaaaagatcccgtttttccctct**

**SamoaDB**  **gaagcgatttttcccttgaggccacactccacctctacgatgtctctttttctgtaggaaaagatcccgtttttccctct**

**CDC2B**  **gaagcgatttttcccttgaggccacactccacctctacgatgtctctttttctgtaggaaaagatcccgtttttccctct**

**BosniaAB**  **gaagcgatttttcccttgaggccacactccacctctacgatgtctctttttctgtaggaaaagatcccgtttttccctct**

**IraqBB**  **gaagcgatttttcccttgaggccacactccacctctacgatgtctctttttctgtaggaaaagatcccgtttttccctct**

**Fribourg-BlancB** **gaagcgatttttcccttgaggccacactccacctctacgatgtctctttttctgtaggaaaagatcccgtttttccctct**

**CuniculiAB**  **gaagcgatttttcccttgaggccacactccacctctacgatgtctctttttctgtaggaaaagatcccgtttttccctct**

490 500 510 520 530 540 550 560

....|....|....|....|....|....|....|....|....|....|....|....|....|....|....|....|

**NicholsB**  **aattttgcgcagttgtggaccccctttattactactagttatgagtcaaggagcgtcaaatacgctccagggtttggtgg**

**Bal3B**  **aattttgcgcagttgtggaccccctttattactactagttatgagtcaaggagcgtcaaatacgctccagggtttggtgg**

**Sea81-4B**  **aattttgcgcagttgtggaccccctttattactactagttatgagtcaaggagcgtcaaatacgctccagggtttggtgg**

**MexicoAB**  **aattttgcgcagttgtggaccccctttattactactagttatgagtcaaggagcgtcaaatacgctccagggtttggtgg**

**Street14B aattttgcgcagttgtggaccccctttattactactagttatgagtcaaggagcgtcaaatacgctccagggtttggtgg**

**GauthierB**  **aattttgcgcagttgtggaccccctttattactactagttatgagtcaaggagcgtcaaatacgctccagggtttggtgg**

**SamoaDB**  **aattttgcgcagttgtggaccccctttattactactagttatgagtcaaggagcgtcaaatacgctccagggtttggtgg**

**CDC2B**  **aattttgcgcagttgtggaccccctttattactactagttatgagtcaaggagcgtcaaatacgctccagggtttggtgg**

**BosniaAB**  **aattttgcgcagttgtggaccccctttattactactagttatgagtcaaggagcgtcaaatacgctccagggtttggtgg**

**IraqBB**  **aattttgcgcagttgtggaccccctttattactactagttatgagtcaaggagcgtcaaatacgctccagggtttggtgg**

**Fribourg-BlancB** **aattttgcgcagttgtggaccccctttattactactagttatgagtcaaggagcgtcaaatacgctccagggtttggtgg**

**CuniculiAB**  **aattttgcgcagttgtggaccccctttattactactagttatgagtcaaggagcgtcaaatacgctccagggtttggtgg**

570 580 590 600 610 620 630 640

....|....|....|....|....|....|....|....|....|....|....|....|....|....|....|....|

**NicholsB**  **ggttggcggaaaaatcgcatatcaggcacggaatatttcgaacagtggcattacattcaactgtgccctttccttttcgt**

**Bal3B**  **ggttggcggaaaaatcgcatatcaggcacggaatatttcgaacagtggcattacattcaactgtgccctttccttttcgt**

**Sea81-4B**  **ggttggcggaaaaatcgcatatcaggcacggaatatttcgaacagtggcattacattcaactgtgccctttccttttcgt**

**MexicoAB**  **ggttggcggaaaaatcgcatatcaggcacggaatatttcgaacagtggcattacattcaactgtgccctttccttttcgt**

**Street14B ggttggcggaaaaatcgcatatcaggcacggaatatttcgaacagtggcattacattcaactgtgccctttccttttcgt**

**GauthierB**  **ggttggcggaaaaatcgcatatcaggcacggaatatttcgaacagtggcattacattcaactgtgccctttccttttcgt**

**SamoaDB**  **ggttggcggaaaaatcgcatatcaggcacggaatatttcgaacagtggcattacattcaactgtgccctttccttttcgt**

**CDC2B**  **ggttggcggaaaaatcgcatatcaggcacggaatatttcgaacagtggcattacattcaactgtgccctttccttttcgt**

**BosniaAB**  **ggttgggggaaaaatcgcatatcaggcacggaatatttcgaacagtggcattacattcaactgtgccctttccttttcgt**

**IraqBB**  **ggttgggggaaaaatcgcatatcaggcacggaatatttcgaacagtggcattacattcaactgtgccctttccttttcgt**

**Fribourg-BlancB** **ggttggcggaaaaatcgcatatcaggcacggaatatttcgaacagtggcattacattcaactgtgccctttccttttcgt**

**CuniculiAB**  **ggttggcggaaaaatcgcatatcaggcacggaatatttcgaacagtggcattacattcaactgtgccctttccttttcgt**

650 660 670 680 690 700 710 720

....|....|....|....|....|....|....|....|....|....|....|....|....|....|....|....|

**NicholsB**  **cgaacggtatatggaaaagtgctccttctgtcacctctaaggtgaaaggaaagggcaccaatagtcggcgcatgccagcg**

**Bal3B**  **cgaacggtatatggaaaagtgctccttctgtcacctctaaggtgaaaggaaagggcaccaatagtcggcgcatgccagcg**

**Sea81-4B**  **cgaacggtatatggaaaagtgctccttctgtcacctctaaggtgaaaggaaagggcaccaatagtcggcgcatgccagcg**

**MexicoAB**  **cgaacggtatatggaaaagtgctccttctgtcacctctaaggtgaaaggaaagggcaccaatagtcggcgcatgccagcg**

**Street14B cgaacggtatatggaaaagtgctccttctgtcacctctaaggtgaaaggaaagggcaccaatagtcggcgcatgccagcg**

**GauthierB**  **cgaacggtatatggaaaagtgctccttctgtcacctctaaggtgaaaggaaagggcaccaatagtcggcgcatgccagcg**

**SamoaDB**  **cgaacggtatatggaaaagtgctccttctgtcacctctaaggtgaaaggaaagggcaccaatagtcggcgcatgccagcg**

**CDC2B**  **cgaacggtatatggaaaagtgctccttctgtcacctctaaggtgaaaggaaagggcaccaatagtcggcgcatgccagcg**

**BosniaAB**  **cgaacggtatatggaaaagtgctccttctgtcacctctaaggtgaaaggaaagggcaccaatagtcggcgcatgccagcg**

**IraqBB**  **cgaacggtatatggaaaagtgctccttctgtcacctctaaggtgaaaggaaagggcaccaatagtcggcgcatgccagcg**

**Fribourg-BlancB** **cgaacggtatatggaaaagtgctccttctgtcacctctaaggtgaaaggaaagggcaccaatagtcggcgcatgccagcg**

**CuniculiAB**  **cgaacggcatatggaagagtgctccttctgtcacctctaaggtgaaaggaaagggcaccaatagtcggcgcatgccagcg**

730 740 750 760 770 780 790 800

....|....|....|....|....|....|....|....|....|....|....|....|....|....|....|....|

**NicholsB**  **gacccgcacagtaaatatggccttggtactgagttcacgctcgtatacgcccgaaaggggcgggaacaggtcaggcttga**

**Bal3B**  **gacccgcacagtaaatatggccttggtactgagttcacgctcgtatacgcccgaaaggggcgggaacaggtcaggcttga**

**Sea81-4B**  **gacccgcacagtaaatatggccttggtactgagttcacgctcgtatacgcccgaaaggggcgggaacaggtcaggcttga**

**MexicoAB**  **gacccgcacagtaaatatggccttggtactgagttcacgctcgtatacgcccgaaaggggcgggaacaggtcaggcttga**

**Street14B gacccgcacagtaaatatggccttggtactgagttcacgctcgtatacgcccgaaaggggcgggaacaggtcaggcttga**

**GauthierB**  **gacccgcacagtaaatatggccttggtactgagttcacgctcgtatacgcccgaaaggggcgggaacaggtcaggcttga**

**SamoaDB**  **gacccgcacagtaaatatggccttggtactgagttcacgctcgtatacgcccgaaaggggcgggaacaggtcaggcttga**

**CDC2B**  **gacccgcacagtaaatatggccttggtactgagttcacgctcgtatacgcccgaaaggggcgggaacaggtcaggcttga**

**BosniaAB**  **gacccgcacagtaaatatggccttggtactgagttcacgctcgtatacgcccgaaaggggcgggaacaggtcaggcttga**

**IraqBB**  **gacccgcacagtaaatatggccttggtactgagttcacgctcgtatacgcccgaaaggggcgggaacaggtcaggcttga**

**Fribourg-BlancB** **gacccgcacagtaaatatggccttggtactgagttcacgctcgtatacgcccgaaaggggcgggaacaggtcaggcttga**

**CuniculiAB**  **gacccgcacagtaaatatggccttggtactgagttcacgctcgtatacgcccgaaaggggcgggaacaggtcaggcttga**

810 820 830 840 850 860 870 880

....|....|....|....|....|....|....|....|....|....|....|....|....|....|....|....|

**NicholsB**  **ggcagcaagttgtgctactctttctgcagggtacagaactggacctgatcagacgcatcatcaaaacaaagatactgtgc**

**Bal3B**  **ggcagcaagttgtgctactctttctgcagggtacagaactggacctgatcagacgcatcatcaaaacaaagatactgtgc**

**Sea81-4B**  **ggcagcaagttgtgctactctttctgcagggtacagaactggacctgatcagacgcatcatcaaaacaaagatactgtgc**

**MexicoAB**  **ggcagcaagttgtgctactctttctgcagggtacagaactggacctgatcagacgcatcatcaaaacaaagatactgtgc**

**Street14B ggcagcaagttgtgctactctttctgcagggtacagaactggacctgatcagacgcatcatcaaaacaaagatactgtgc**

**GauthierB**  **ggcagcaagttgtgctactctttctgcagggtacagaactggacctgatcagacgcatcatcaaaacaaagatactgtgc**

**SamoaDB**  **ggcagcaagttgtgctactctttctgcagggtacagaactggacctgatcagacgcatcatcaaaacaaagatactgtgc**

**CDC2B**  **ggcagcaagttgtgctactctttctgcagggtacagaactggacctgatcagacgcatcatcaaaacaaagatactgtgc**

**BosniaAB**  **ggcagcaagttgtgctactctttctgcagggtacagaactggacctgatcagacgcatcatcaaaacaaagatactgtgc**

**IraqBB**  **ggcagcaagttgtgctactctttctgcagggtacagaactggacctgatcagacgcatcatcaaaacaaagatactgtgc**

**Fribourg-BlancB** **ggcagcaagttgtgctactctttctgcagggtacagaactggacctgatcagacgcatcatcaaaacaaagatactgtgc**

**CuniculiAB**  **ggcagcaagttgtgctactctttctgcagggtacagaactggacctgatcagacgcatcatcaaaacaaagatactgtgc**

890 900 910 920 930 940 950 960

....|....|....|....|....|....|....|....|....|....|....|....|....|....|....|....|

**NicholsB**  **tgtggaatgtaggcgcgcgtttgacgctttccccgggagcaggattcaagatcgtgtgtgccttcgatgctgggacaccg**

**Bal3B**  **tgtggaatgtaggcgcgcgtttgacgctttccccgggagcaggattcaagatcgtgtgtgccttcgatgctgggacaccg**

**Sea81-4B**  **tgtggaatgtaggcgcgcgtttgacgctttccccgggagcaggattcaagatcgtgtgtgccttcgatgctgggacaccg**

**MexicoAB**  **tgtggaatgtaggcgcgcgtttgacgctttccccgggagcaggattcaagatcgtgtgtgccttcgatgctgggacaccg**

**Street14B tgtggaatgtaggcgcgcgtttgacgctttccccgggagcaggattcaagatcgtgtgtgccttcgatgctgggacaccg**

**GauthierB**  **tgtggaatgtaggcgcgcgtttgacgctttccccgggagcaggattcaagatcgtgtgtgccttcgatgctgggacaccg**

**SamoaDB**  **tgtggaatgtaggcgcgcgtttgacgctttccccgggagcaggattcaagatcgtgtgtgccttcgatgctgggacaccg**

**CDC2B**  **tgtggaatgtaggcgcgcgtttgacgctttccccgggagcaggattcaagatcgtgtgtgccttcgatgctgggacaccg**

**BosniaAB**  **tgtggaatgtaggcgcgcgtttgacgctttccccgggagcaggattcaagatcgtgtgtgccttcgatgctgggacaccg**

**IraqBB**  **tgtggaatgtaggcgcgcgtttgacgctttccccgggagcaggattcaagatcgtgtgtgccttcgatgctgggacaccg**

**Fribourg-BlancB** **tgtggaatgtaggcgcgcgtttgacgctttccccgggagcaggattcaagatcgtgtgtgccttcgatgctgggacaccg**

**CuniculiAB**  **tgtggaatgtaggcgcgcgtttgacgctttccccgggagcaggattcaagatcgtgtgtgccttcgatgctgggacaccg**

970 980 990 1000 1010 1020 1030 1040

....|....|....|....|....|....|....|....|....|....|....|....|....|....|....|....|

**NicholsB**  **tacaagaagggtgccgcgagggagtccctcgctgaaacgcttgcggcacagcgtggttgtaatcgttttgacaccgcgct**

**Bal3B**  **tacaagaagggtgccgcgagggagtccctcgctgaaacgcttgcggcacagcgtggttgtaatcgttttgacaccgcgct**

**Sea81-4B**  **tacaagaagggtgccgcgagggagtccctcgctgaaacgcttgcggcacagcgtggttgtaatcgttttgacaccgcgct**

**MexicoAB**  **tacaagaagggtgccgcgagggagtccctcgctgaaacgcttgcggcacagcgtggttgtaatcgttttgacaccgcgct**

**Street14B tacaagaagggtgccgcgagggagtccctcgctgaaacgcttgcggcacagcgtggttgtaatcgttttgacaccgcgct**

**GauthierB**  **tacaagaagggtgccgcgagggagtccctcgctgaaacgcttgcggcacagcgtggttgtaatcgttttgacaccgcgct**

**SamoaDB**  **tacaagaagggtgccgcgagggagtccctcgctgaaacgcttgcggcacagcgtggttgtaatcgttttgacaccgcgct**

**CDC2B**  **tacaagaagggtgccgcgagggagtccctcgctgaaacgcttgcggcacagcgtggttgtaatcgttttgacaccgcgct**

**BosniaAB**  **tacaagaagggtgccgcgagggagtccctcgctgaaacgcttgcggcacagcgtggttgtaatcgttttgacaccgcgct**

**IraqBB**  **tacaagaagggtgccgcgagggagtccctcgctgaaacgcttgcggcacagcgtggttgtaatcgttttgacaccgcgct**

**Fribourg-BlancB** **tacaagaagggtgccgcgagggagtccctcgctgaaacgcttgcggcacagcgtggttgtaatcgttttgacaccgcgct**

**CuniculiAB**  **tacaagaagggtgccgcgagggagtccctcgctgaaacgcttgcggcacagcgtggttgtaatcgttttgacaccgcgct**

1050 1060 1070 1080 1090 1100 1110 1120

....|....|....|....|....|....|....|....|....|....|....|....|....|....|....|....|

**NicholsB**  **catgcacgcgcttgggttacttgttgctgctgcgaagacacgcaatgaactcgccgcacagatgcgatcgcagtcaccac**

**Bal3B**  **catgcacgcgcttgggttacttgttgctgctgcgaagacacgcaatgaactcgccgcacagatgcgatcgcagtcaccac**

**Sea81-4B**  **catgcacgcgcttgggttacttgttgctgctgcgaagacacgcaatgaactcgccgcacagatgcgatcgcagtcaccac**

**MexicoAB**  **catgcacgcgcttgggttacttgttgctgctgcgaagacacgcaatgaactcgccgcacagatgcgatcgcagtcaccac**

**Street14B catgcacgcgcttgggttacttgttgctgctgcgaagacacgcaatgaactcgccgcacagatgcgatcgcagtcaccac**

**GauthierB**  **catgcacgcgcttgggttacttgttgctgctgcgaagacacgcaatgaactcgccgcacagatgcgatcgcagtcaccac**

**SamoaDB**  **catgcacgcgcttgggttacttgttgctgctgcgaagacacgcaatgaactcgccgcacagatgcgatcgcagtcaccac**

**CDC2B**  **catgcacgcgcttgggttacttgttgctgctgcgaagacacgcaatgaactcgccgcacagatgcgatcgcagtcaccac**

**BosniaAB**  **catgcacgcgcttgggttacttgttgctgctgcgaagacacgcaatgaactcgccgcacagatgcgatcgcagtcaccac**

**IraqBB**  **catgcacgcgcttgggttacttgttgctgctgcgaagacacgcaatgaactcgccgcacagatgcgatcgcagtcaccac**

**Fribourg-BlancB** **catgcacgcgcttgggttacttgttgctgctgcgaagacacgcaatgaactcgccgcacagatgcgatcgcagtcaccac**

**CuniculiAB**  **catgcacgcgcttgggttacttgttgctgctgcgaagacacgcaatgaactcgccgcacagatgcgatcgcagtcaccac**

1130 1140 1150 1160 1170 1180 1190 1200

....|....|....|....|....|....|....|....|....|....|....|....|....|....|....|....|

**NicholsB**  **caggtgtgtgggaaaaatttgaacaggcggtgcaatcgttacctcctataacgcagggaaagcctggcgtcgttggggcg**

**Bal3B**  **caggtgtgtgggaaaaatttgaacaggcggtgcaatcgttacctcctataacgcagggaaagcctggcgtcgttggggcg**

**Sea81-4B**  **caggtgtgtgggaaaaatttgaacaggcggtgcaatcgttacctcctataacgcagggaaagcctggcgtcgttggggcg**

**MexicoAB**  **caggtgtgtgggaaaaatttgaacaggcggtgcaatcgttacctcctataacgcagggaaagcctggcgtcgttggggcg**

**Street14B caggtgtgtgggaaaaatttgaacaggcggtgcaatcgttacctcctataacgcagggaaagcctggcgtcgttggggcg**

**GauthierB**  **caggtgtgtgggaaaaatttgaacaggcggtgcaatcgttacctcctataacgcagggaaagcctggcgtcgttggggcg**

**SamoaDB**  **caggtgtgtgggaaaaatttgaacaggcggtgcaatcgttacctcctataacgcagggaaagcctggcgtcgttggggcg**

**CDC2B**  **caggtgtgtgggaaaaatttgaacaggcggtgcaatcgttacctcctataacgcagggaaagcctggcgtcgttggggcg**

**BosniaAB**  **caggtgtgtgggaaaaatttgaacaggcggtgcaatcgttacctcctataacgcagggaaagcctggcgtcgttggggcg**

**IraqBB**  **caggtgtgtgggaaaaatttgaacaggcggtgcaatcgttacctcctataacgcagggaaagcctggcgtcgttggggcg**

**Fribourg-BlancB** **caggtgtgtgggaaaaatttgaacaggcggtgcaatcgttacctcctataacgcagggaaagcctggcgtcgttggggcg**

**CuniculiAB**  **caggtgtgtgggaaaaatttgaacaggcggtgcaatcgttacctcctataacgcagggaaagcctggcgtcgttggggcg**

1210 1220 1230 1240 1250 1260 1270 1280

....|....|....|....|....|....|....|....|....|....|....|....|....|....|....|....|

**NicholsB**  **gaggtccgcccgggtacgatgtggatggaactttccccggtaaggaaagcacttgtcgatgtactttctgtacttgagca**

**Bal3B**  **gaggtccgcccgggtacgatgtggatggaactttccccggtaaggaaagcacttgtcgatgtactttctgtacttgagca**

**Sea81-4B**  **gaggtccgcccgggtacgatgtggatggaactttccccggtaaggaaagcacttgtcgatgtactttctgtacttgagca**

**MexicoAB**  **gaggtccgcccgggtacgatgtggatggaactttccccggtaaggaaagcacttgtcgatgtactttctgtacttgagca**

**Street14B gaggtccgcccgggtacgatgtggatggaactttccccggtaaggaaagcacttgtcgatgtactttctgtacttgagca**

**GauthierB**  **gaggtccgcccgggtacgatgtggatggaactttccccggtaaggaaagcacttgtcgatgtactttctgtacttgagca**

**SamoaDB**  **gaggtccgcccgggtacgatgtggatggaactttccccggtaaggaaagcacttgtcgatgtactttctgtacttgagca**

**CDC2B**  **gaggtccgcccgggtacgatgtggatggaactttccccggtaaggaaagcacttgtcgatgtactttctgtacttgagca**

**BosniaAB**  **gaggtccgcccgggtacgatgtggatggaactttccccggtaaggaaagcacttgtcgatgtactttctgtacttgagca**

**IraqBB**  **gaggtccgcccgggtacgatgtggatggaactttccccggtaaggaaagcacttgtcgatgtactttctgtacttgagca**

**Fribourg-BlancB** **gaggtccgcccgggtacgatgtggatggaactttccccggtaaggaaagcacttgtcgatgtactttctgtacttgagca**

**CuniculiAB**  **gaggtccgcccgggtacgatgtggatggaactttccccggtaaggaaagcacttgtcgatgtactttctgtacttgagca**

1290 1300 1310 1320 1330 1340 1350 1360

....|....|....|....|....|....|....|....|....|....|....|....|....|....|....|....|

**NicholsB**  **gggtggttttgatcgtgtcgcctttgacgcattgctgattgtgcaatggcgctggatttcgctgggagcatacgtagcaa**

**Bal3B**  **gggtggttttgatcgtgtcgcctttgacgcattgctgattgtgcaatggcgctggatttcgctgggagcatacgtagcaa**

**Sea81-4B**  **gggtggttttgatcgtgtcgcctttgacgcattgctgattgtgcaatggcgctggatttcgctgggagcatacgtagcaa**

**MexicoAB**  **gggtggttttgatcgtgtcgcctttgacgcattgctgattgtgcaatggcgctggatttcgctgggagcatacgtagcaa**

**Street14B gggtggttttgatcgtgtcgcctttgacgcattgctgattgtgcaatggcgctggatttcgctgggagcatacgtagcaa**

**GauthierB**  **gggtggttttgatcgtgtcgcctttgacgcattgctgattgtgcaatggcgctggatttcgctgggagcatacgtagcaa**

**SamoaDB**  **gggtggttttgatcgtgtcgcctttgacgcattgctgattgtgcaatggcgctggatttcgctgggagcatacgtagcaa**

**CDC2B**  **gggtggttttgatcgtgtcgcctttgacgcattgctgattgtgcaatggcgctggatttcgctgggagcatacgtagcaa**

**BosniaAB**  **gggtggttttgatcgtgtcgcctttgacgcattgctgattgtgcaatggcgctggatttcgctgggagcatacgtagcaa**

**IraqBB**  **gggtggttttgatcgtgtcgcctttgacgcattgctgattgtgcaatggcgctggatttcgctgggagcatacgtagcaa**

**Fribourg-BlancB** **gggtggttttgatcgtgtcgcctttgacgcattgctgattgtgcaatggcgctggatttcgctgggagcatacgtagcaa**

**CuniculiAB**  **gggtggttttgatcgtgtcgcctttgacgcattgctgattgtgcaatggcgctggatttcgctgggagcatacgtagcaa**

1370 1380 1390 1400 1410 1420 1430 1440

....|....|....|....|....|....|....|....|....|....|....|....|....|....|....|....|

**NicholsB**  **gtgctcctaccaatgtgtttggctcaatgctttttccgcgtgggagtagtgaccattttgactgtgccgcattcgtgcgg**

**Bal3B**  **gtgctcctaccaatgtgtttggctcaatgctttttccgcgtgggagtagtgaccattttgactgtgccgcattcgtgcgg**

**Sea81-4B**  **gtgctcctaccaatgtgtttggctcaatgctttttccgcgtgggagtagtgaccattttgactgtgccgcattcgtgcgg**

**MexicoAB**  **gtgctcctaccaatgtgtttggctcaatgctttttccgcgtgggagtagtgaccattttgactgtgccgcattcgtgcgg**

**Street14B gtgctcctaccaatgtgtttggctcaatgctttttccgcgtgggagtagtgaccattttgactgtgccgcattcgtgcgg**

**GauthierB**  **gtgctcctaccaatgtgtttggctcaatgctttttccgcgtgggagtagtgaccattttgactgtgccgcattcgtgcgg**

**SamoaDB**  **gtgctcctaccaatgtgtttggctcaatgctttttccgcgtgggagtagtgaccattttgactgtgccgcattcgtgcgg**

**CDC2B**  **gtgctcctaccaatgtgtttggctcaatgctttttccgcgtgggagtagtgaccattttgactgtgccgcattcgtgcgg**

**BosniaAB**  **gtgctcctaccaatgtgtttggctcaatgctttttccgcgtgggagtagtgaccattttgactgtgccgcattcgtgcgg**

**IraqBB**  **gtgctcctaccaatgtgtttggctcaatgctttttccgcgtgggagtagtgaccattttgactgtgccgcattcgtgcgg**

**Fribourg-BlancB** **gtgctcctaccaatgtgtttggctcaatgctttttccgcgtgggagtagtgaccattttgactgtgccgcattcgtgcgg**

**CuniculiAB**  **gtgcttctaccaatgtgtttggctcaatgctttttccgcgcgggagtagtgaccattttgactgtgccgcattcgtgcgg**

1450 1460 1470 1480 1490 1500 1510 1520

....|....|....|....|....|....|....|....|....|....|....|....|....|....|....|....|

**NicholsB**  **gtggaaagtaagtggtacgattctctttctaagcttgtgtccggtctgagcggaggtgtcgaggcgcgtttatatattcc**

**Bal3B**  **gtggaaagtaagtggtacgattctctttctaagcttgtgtccggtctgagcggaggtgtcgaggcgcgtttatatattcc**

**Sea81-4B**  **gtggaaagtaagtggtacgattctctttctaagcttgtgtccggtctgagcggaggtgtcgaggcgcgtttatatattcc**

**MexicoAB**  **gtggaaagtaagtggtacgattctctttctaagcttgtgtccggtctgagcggaggtgtcgaggcgcgtttatatattcc**

**Street14B gtggaaagtaagtggtacgattctctttctaagcttgtgtccggtctgagcggaggtgtcgaggcgcgtttatatattcc**

**GauthierB**  **gtggaaagtaagtggtacgattctctttctaagcttgtgtccggtctgagcggaggtgtcgaggcgcgtttatatattcc**

**SamoaDB**  **gtggaaagtaagtggtacgattctctttctaagcttgtgtccggtctgagcggaggtgtcgaggcgcgtttatatattcc**

**CDC2B**  **gtggaaagtaagtggtacgattctctttctaagcttgtgtccggtctgagcggaggtgtcgaggcgcgtttatatattcc**

**BosniaAB**  **gtggaaagtaagtggtacgattctctttctaagcttgtgtccggtctgagcggaggtgtcgaggcgcgtttatatattcc**

**IraqBB**  **gtggaaagtaagtggtacgattctctttctaagcttgtgtccggtctgagcggaggtgtcgaggcgcgtttatatattcc**

**Fribourg-BlancB** **gtggaaagtaagtggtacgattctctttctaagcttgtgtccggtctgagcggaggtgtcgaggcgcgtttatatattcc**

**CuniculiAB**  **gtggaaagtaagtggtacgattctctttctaagcttgtgtccggtctgagcggaggtgtcgaggcgcgtttatatattcc**

1530 1540 1550 1560 1570 1580 1590 1600

....|....|....|....|....|....|....|....|....|....|....|....|....|....|....|....|

**NicholsB**  **tttcactcatggcttgtacctggaacctggctcctgtacgccgaattccacacggggaaagaagcctcaggcgtttgtgc**

**Bal3B**  **tttcactcatggcttgtacctggaacctggctcctgtacgccgaattccacacggggaaagaagcctcaggcgtttgtgc**

**Sea81-4B**  **tttcactcatggcttgtacctggaacctggctcctgtacgccgaattccacacggggaaagaagcctcaggcgtttgtgc**

**MexicoAB**  **tttcactcatggcttgtacctggaacctggctcctgtacgccgaattccacacggggaaagaagcctcaggcgtttgtgc**

**Street14B tttcactcatggcttgtacctggaacctggctcctgtacgccgaattccacacggggaaagaagcctcaggcgtttgtgc**

**GauthierB**  **tttcactcatggcttgtacctggaacctggctcctgtacgccgaattccacacggggaaagaagcctcaggcgtttgtgc**

**SamoaDB**  **tttcactcatggcttgtacctggaacctggctcctgtacgccgaattccacacggggaaagaagcctcaggcgtttgtgc**

**CDC2B**  **tttcactcatggcttgtacctggaacctggctcctgtacgccgaattccacacggggaaagaagcctcaggcgtttgtgc**

**BosniaAB**  **tttcactcatggcttgtacctggaacctggctcctgtacgccgaattccacacggggaaagaagcctcaggcgtttgtgc**

**IraqBB**  **tttcactcatggcttgtacctggaacctggctcctgtacgccgaattccacacggggaaagaagcctcaggcgtttgtgc**

**Fribourg-BlancB** **tttcactcatggcttgtacctggaacctggctcctgtacgccgaattccacacggggaaagaagcctcaggcgtttgtgc**

**CuniculiAB**  **tttcactcatggcttgtacctggaacctggctcctgtacgccgaattccacacggggaaagaagcctcaggcgtttgtgc**

1610 1620 1630 1640 1650 1660 1670 1680

....|....|....|....|....|....|....|....|....|....|....|....|....|....|....|....|

**NicholsB**  **ttcctccacccggtgttgtccatccgggtgcacacagcgcgttgcctgttgtggggaaactgtggcttaactaccggatt**

**Bal3B**  **ttcctccacccggtgttgtccatccgggtgcacacagcgcgttgcctgttgtggggaaactgtggcttaactaccggatt**

**Sea81-4B**  **ttcctccacccggtgttgtccatccgggtgcacacagcgcgttgcctgttgtggggaaactgtggcttaactaccggatt**

**MexicoAB**  **ttcctccacccggtgttgtccatccgggtgcacacagcgcgttgcctgttgtggggaaactgtggcttaactaccggatt**

**Street14B ttcctccacccggtgttgtccatccgggtgcacacagcgcgttgcctgttgtggggaaactgtggcttaactaccggatt**

**GauthierB**  **ttcctccacccggtgttgtccatccgggtgcacacagcgcgttgcctgttgtggggaaactgtggcttaactaccggatt**

**SamoaDB**  **ttcctccacccggtgttgtccatccgggtgcacacagcgcgttgcctgttgtggggaaactgtggcttaactaccggatt**

**CDC2B**  **ttcctccacccggtgttgtccatccgggtgcacacagcgcgttgcctgttgtggggaaactgtggcttaactaccggatt**

**BosniaAB**  **ttcctccacccggtgttgtccatccgggtgcacacagcgcgttgcctgttgtggggaaactgtggcttaactaccggatt**

**IraqBB**  **ttcctccacccggtgttgtccatccgggtgcacacagcgcgttgcctgttgtggggaaactgtggcttaactaccggatt**

**Fribourg-BlancB** **ttcctccacccggtgttgtccatccgggtgcacacagcgcgttgcctgttgtggggaaactgtggcttaactaccggatt**

**CuniculiAB**  **ttcctccacccggtgttgtccatccgggtgcacacagcgcgttgcctgttgtggggaaactatggcttaactaccggatt**

1690 1700 1710 1720 1730 1740 1750 1760

....|....|....|....|....|....|....|....|....|....|....|....|....|....|....|....|

**NicholsB**  **actcttgctgctcacgcatggataagaccgatggtttcgctgtatggcgctacatatggtgcgcagggtttttcttacgg**

**Bal3B**  **actcttgctgctcacgcatggataagaccgatggtttcgctgtatggcgctacatatggtgcgcagggtttttcttacgg**

**Sea81-4B**  **actcttgctgctcacgcatggataagaccgatggtttcgctgtatggcgctacatatggtgcgcagggtttttcttacgg**

**MexicoAB**  **actcttgctgctcacgcatggataagaccgatggtttcgctgtatggcgctacatatggtgcgcagggtttttcttacgg**

**Street14B actcttgctgctcacgcatggataagaccgatggtttcgctgtatggcgctacatatggtgcgcagggtttttcttacgg**

**GauthierB**  **actcttgctgctcacgcatggataagaccgatggtttcgctgtatggcgctacatatggtgcgcagggtttttcttacgg**

**SamoaDB**  **actcttgctgctcacgcatggataagaccgatggtttcgctgtatggcgctacatatggtgcgcagggtttttcttacgg**

**CDC2B**  **actcttgctgctcacgcatggataagaccgatggtttcgctgtatggcgctacatatggtgcgcagggtttttcttacgg**

**BosniaAB**  **actcttgctgctcacgcatggataagaccgatggtttcgctgtatggcgctacatatggtgcgcagggtttttcttacgg**

**IraqBB**  **actcttgctgctcacgcatggataagaccgatggtttcgctgtatggcgctacatatggtgcgcagggtttttcttacgg**

**Fribourg-BlancB** **actcttgctgctcacgcatggataagaccgatggtttcgctgtatggcgctacatatggtgcgcagggtttttcttacgg**

**CuniculiAB**  **actcttgctgctcacgcatggataagaccgatggtttcgctgtatggcgctacatatggtgcgcagggtttttcttacgg**

1770 1780 1790 1800 1810 1820 1830 1840

....|....|....|....|....|....|....|....|....|....|....|....|....|....|....|....|

**NicholsB**  **accaggtggtgctgccggaacggtaaagaggaaccgcacgttccgcgcaggtaaaaatctgtattatcaggtaggggtgg**

**Bal3B**  **accaggtggtgctgccggaacggtaaagaggaaccgcacgttccgcgcaggtaaaaatctgtattatcaggtaggggtgg**

**Sea81-4B**  **accaggtggtgctgccggaacggtaaagaggaaccgcacgttccgcgcaggtaaaaatctgtattatcaggtaggggtgg**

**MexicoAB**  **accaggtggtgctgccggaacggtaaagaggaaccgcacgttccgcgcaggtaaaaatctgtattatcaggtaggggtgg**

**Street14B accaggtggtgctgccggaacggtaaagaggaaccgcacgttccgcgcaggtaaaaatctgtattatcaggtaggggtgg**

**GauthierB**  **accaggtggtgctgccggaacggtaaagaggaaccgcacgttccgcgcaggtaaaaatctgtattatcaggtaggggtgg**

**SamoaDB**  **accaggtggtgctgccggaacggtaaagaggaaccgcacgttccgcgcaggtaaaaatctgtattatcaggtaggggtgg**

**CDC2B**  **accaggtggtgctgccggaacggtaaagaggaaccgcacgttccgcgcaggtaaaaatctgtattatcaggtaggggtgg**

**BosniaAB**  **accaggtggtgctgccggaacggtaaagaggaaccgcacgttccgcgcaggtaaaaatctgtattatcaggtaggggtgg**

**IraqBB**  **accaggtggtgctgccggaacggtaaagaggaaccgcacgttccgcgcaggtaaaaatctgtattatcaggtaggggtgg**

**Fribourg-BlancB** **accaggtggtgctgccggaacggtaaagaggaaccgcacgttccgcgcaggtaaaaatctgtattatcaggtaggggtgg**

**CuniculiAB**  **accaggtggtgctgccggaacggtaaagaggagtcgcacgttccgcgcaggtaaaaatctgtactatcaggtaggggtgg**

1850 1860 1870 1880 1890 1900 1910 1920

....|....|....|....|....|....|....|....|....|....|....|....|....|....|....|....|

**NicholsB**  **cggtgagtccgtttgaaagatgtgagttgctcattgagtggtcgcagggaatgcttgcgcgtcgtccctacataagtttg**

**Bal3B**  **cggtgagtccgtttgaaagatgtgagttgctcattgagtggtcgcagggaatgcttgcgcgtcgtccctacataagtttg**

**Sea81-4B**  **cggtgagtccgtttgaaagatgtgagttgctcattgagtggtcgcagggaatgcttgcgcgtcgtccctacataagtttg**

**MexicoAB**  **cggtgagtccgtttgaaagatgtgagttgctcattgagtggtcgcagggaatgcttgcgcgtcgtccctacataagtttg**

**Street14B cggtgagtccgtttgaaagatgtgagttgctcattgagtggtcgcagggaatgcttgcgcgtcgtccctacataagtttg**

**GauthierB**  **cggtgagtccgtttgaaagatgtgagttgctcattgagtggtcgcagggaatgcttgcgcgtcgtccctacataagtttg**

**SamoaDB**  **cggtgagtccgtttgaaagatgtgagttgctcattgagtggtcgcagggaatgcttgcgcgtcgtccctacataagtttg**

**CDC2B**  **cggtgagtccgtttgaaagatgtgagttgctcattgagtggtcgcagggaatgcttgcgcgtcgtccctacataagtttg**

**BosniaAB**  **cggtgagtccgtttgaaagatgtgagttgctcattgagtggtcgcagggaatgcttgcgcgtcgtccctacataagtttg**

**IraqBB**  **cggtgagtccgtttgaaagatgtgagttgctcattgagtggtcgcagggaatgcttgcgcgtcgtccctacataagtttg**

**Fribourg-BlancB** **cggtgagtccgtttgaaagatgtgagttgctcattgagtggtcgcagggaatgcttgcgcgtcgtccctacataagtttg**

**CuniculiAB**  **cggtgagtccgtttgaaagatgtgagttactcattgagtggtcgcagggaatgcttgcgcgtcgtccctatataagtttg**

1930 1940 1950 1960 1970 1980

....|....|....|....|....|....|....|....|....|....|....|....|...

**NicholsB**  **gagcatggttcttggccggataagcggagcgagctcgtgtgttcgtgtaaggttctgtggtga**

**Bal3B**  **gagcatggttcttggccggataagcggagcgagctcgtgtgttcgtgtaaggttctgtggtga**

**Sea81-4B**  **gagcatggttcttggccggataagcggagcgagctcgtgtgttcgtgtaaggttctgtggtga**

**MexicoAB**  **gagcatggttcttggccggataagcggagcgagctcgtgtgttcgtgtaaggttctgtggtga**

**Street14B gagcatggttcttggccggataagcggagcgagctcgtgtgttcgtgtaaggttctgtggtga**

**GauthierB**  **gagcatggttcttggccggataagcggagcgagctcgtgtgttcgtgtaaggttctgtggtga**

**SamoaDB**  **gagcatggttcttggccggataagcggagcgagctcgtgtgttcgtgtaaggttctgtggtga**

**CDC2B**  **gagcatggttcttggccggataagcggagcgagctcgtgtgttcgtgtaaggttctgtggtga**

**BosniaAB**  **gagcatggttcttggccggataagcggagcgagctcgtgtgttcgtgtaaggttctgtggtga**

**IraqBB**  **gagcatggttcttggccggataagcggagcgagctcgtgtgttcgtgtaaggttctgtggtga**

**Fribourg-BlancB** **gagcatggttcttggccggataagcggagcgagctcgtgtgttcgtgtaaggttctgtggtga**

**CuniculiAB**  **gagcatggttcctggccgaataagcggagcgagctcgtgtgttcgtgtaaggttctgtggtga**

***1.3 tprC* and *tprD* loci**

10 20 30 40 50 60 70 80

....|....|....|....|....|....|....|....|....|....|....|....|....|....|....|....|

**NicholsC**  **MGRQVMQAGVLAGMVCAASGYAGVLTPQVSGTAQLQWGIAFQKNPRTGPGKHTHGFRTTNSLTISLPLVSKHTHTRRGEA**

**NicholsD**  **MGRQVMQAGVLAGMVCAASGYAGVLTPQVSGTAQLQWGIAFQKNPRTGPGKHTHGFRTTNSLTISLPLVSKHTHTRRGEA**

**Bal3C**  **MGRQVMQAGVLAGMVCAASGYAGVLTPQVSGTAQLQWGIAFQKNPRTGPGKHTHGFRTTNSLTISLPLVSKHTHTRRGEA**

**Sea81-4C**  **MGRQVMQAGVLAGMVCAASGYAGVLTPQVSGTAQLQWGIAFQKNPRTGPGKHTHGFRTTNSLTISLPLVSKHTHTRRGEA**

**Street14C**  **MGRQVMQAGVLAGMVCAASGYAGVLTPQVSGTAQLQWGIAFQKNPRTGPGKHTHGFRTTNSLTISLPLVSKHTHTRRGEA**

**MexicoAC**  **MGRQVMQAGVLAGMVCAASGYAGVLTPQVSGTAQLQWGIAFQKNPRTGPGKHTHGFRTTNSLTISLPLVSKHTHTRRGEA**

**GauthierC**  **MGKQVMQAGVLAGMVCAASGYAGVLTPQVSGTAQLQWGIAFQKNPHTVPGEHTHGFRTTNSLTISLPLVSKHTHTRRGEA**

**GauthierD**  **MGKQVMQAGVLAGMVCAASGYAGVLTPQVSGTAQLQWGIAFQKNPHTVPGKHTHGFRTTNSLTISLPLVSKHTHTRRGEA**

**CDC2C**  **MGKQVMQAGVLAGMVCAASGYAGVLTPQVSGTAQLQWGIAFQKNPHTVPGEHTHGFRTTNSLTISLPLVSKHTHTRRGEA**

**CDC2D**  **MGKQVMQAGVLAGMVCAASGYAGVLTPQVSGTAQLQWGIAFQKNPHTVPGEHTHGFRTTNSLTISLPLVSKHTHTRRGEA**

**SamoaDC**  **MGKQVMQAGVLAGMVCAASGYAGVLTPQVSGTAQLQWGIAFQKNPHTVPGKHTHGFRTTNSLTISLPLVSKHTHTRRGEA**

**IraqBC**  **MGKQVMQAGVLAGMVCAASGYAGVLTPQVSGTAQLQWGIAFQKNPHTVPGKHTHGFRTTNSLTISLPLVSKHTHTRRGEA**

**BosniaAC**  **MGKQVMQAGVLAGMVCAASGYAGVLTPQVSGAAQLQWGIAFQKNPHTVPGKHTHGFRTTNSLTISLPLVSKHTHTRRGEA**

**Fribourg-BlancC** **MGKQVMQAGVLAGMVCAASGYAGVLTPQVSGTAQLQWGIAFQKNPHTVPGKHTHGFRTTNSLTISLPLVSKHTHTRRGEA**

90 100 110 120 130 140 150 160

....|....|....|....|....|....|....|....|....|....|....|....|....|....|....|....|

**NicholsC**  **RSGVWAQLQLKDLAVELASSKSSTALSFTKPTASFQATLHCYGAYLTVGTSPSCVVNFAQLWKPFVTRAYSEKDTRYAPG**

**NicholsD**  **RSGVWAQLQLKDLAVELASSKSSTALSFTKPTASFQATLHCYGAYLTVGTSPSCVVNFAQLWKPFVTRAYSEKDTRYAPG**

**Bal3C**  **RSGVWAQLQLKDLAVELASSKSSTALSFTKPTASFQATLHCYGAYLTVGTSPSCVVNFAQLWKPFVTRAYSEKDTRYAPG**

**Sea81-4C**  **RSGVWAQLQLKDLAVELASSKSSTALSFTKPTASFQATLHCYGAYLTVGTSPSCVVNFAQLWKPFVTRAYSEKDTRYAPG**

**Street14C RSGVWAQLQLKDLAVELASSKSSTALSFTKPTASFQATLHCYGAYLTVGTSPSCVVNFAQLWKPFVTRAYSEKDTRYAPG**

**MexicoAC**  **RSGVWAQLQLKDLAVELASSKSSTALSFTKPTASFQATLHCYGAYLTVGTSPSCVVNFAQLWKPFVTRAYSEKDTRYAPG**

**GauthierC**  **RSGVWAQLQLKDLAVELASSKSSTALSFTKPTASFQATLHCYGAYLTVGTSPSCVVNFAQLWKPFVTRAYSEKDTRYAPG**

**GauthierD**  **RSGVWAQLQLKDLAVELASSKSSTALSFTKPTASFQATLHCYGAYLTVGTSPSCVVNFAQLWKPFVTRAYSEKDTRYAPG**

**CDC2C**  **RSGVWAQLQLKDLAVELASSKSSTALSFTKPTASFQATLHCYGAYLTVGTSPSCVVNFAQLWKPFVTRAYSEKDTRYAPG**

**CDC2D**  **RSGVWAQLQLKDLAVELASSKSSTALSFTKPTASFQATLHCYGAYLTVGTSPSCVVNFAQLWKPFVTRAYSEKDTRYAPG**

**SamoaDC**  **RSGVWAQLQLKDLAVELASSKSSTALSFTKPTASFQATLHCYGAYLTVGTSPSCVVNFAQLWKPFVTRAYSEKDTRYAPG**

**IraqBC**  **RSGVWAQLQLKDLAVELASSKSSTALSFTKPTASFQATLHCYGAYLTVGTSPSCVVNFAQLWKPFVTRAYSEKDTRYAPG**

**BosniaAC**  **RSGVWAQLQLKDLAVELASSKSSTALSFTKPTASFQATLHCYGAYLTVGTSPSCVVNFAQLWKPFVTRAYSEKDTRYAPG**

**Fribourg-BlancC** **RSGVWAQLQLKDLAVELASSKSSTALSFTKPTASFQATLHCYGAYLTVGTSPSCVVNFAQLWKPFVTRAYSEKDTRYAPG**

170 180 190 200 210 220 230 240

....|....|....|....|....|....|....|....|....|....|....|....|....|....|....|....|

**NicholsC**  **FSGSGAKLGYQAHNVGNSGVDVDIGFLSFLSNGAWDSTDTTHSKYGFGADATLSYGVDRQRLLTLELAGNATLDQNYVKG**

**NicholsD**  **FSGSGAKLGYQAHNVGNSGVDVDIGFLSFLSNGAWDSTDTTHSKYGFGADATLSYGVDRQRLLTLELAGNATLDQNYVKG**

**Bal3C**  **FSGSGAKLGYQAHNVGNSGVDVDIGFLSFLSNGAWDSTDTTHSKYGFGADATLSYGVDRQRLLTLELAGNATLDQNYVKG**

**Sea81-4C**  **FSGSGAKLGYQAHNVGNSGVDVDIGFLSFLSNGAWDSTDTTHSKYGFGADATLSYGVDRQRLLTLELAGNATLDQNYVKG**

**Street14C FSGSGAKLGYQAHNVGNSGVDVDIGFLSFLSNGAWDSTDTTHSKYGFGADATLSYGVDRQRLLTLELAGNATLDQNYVKG**

**MexicoAC**  **FSGSGAKLGYQAHNVGNSGVDVDIGFLSFLSNGAWDSTDTTHSKYGFGADATLSYGVDRQRLLTLELAGNATLEQHYRKG**

**GauthierC**  **FSGSGAKLGYQAHNVGNSGVDVDIGFLSFLSNGAWDSTDTTHSKYGFGADATLSYGVDRQRLLTLELAGNATLEQHYRKG**

**GauthierD**  **FSGSGAKLGYQAHNVGNSGVDVDIGFLSFLSNGAWDSTDTTHSKYGFGADATLSYGVDRQRLLTLELAGNATLEQHYRKG**

**CDC2C**  **FSGSGAKLGYQAHNVGNSGVDVDIGFLSFLSNGAWDSTDTTHSKYGFGADATLSYGVDRQRLLTLELAGNATLEQHYRKG**

**CDC2D**  **FSGSGAKLGYQAHNVGNSGVDVDIGFLSFLSNGAWDSTDTTHSKYGFGADATLSYGVDRQRLLTLELAGNATLEQHYRKG**

**SamoaDC**  **FSGSGAKLGYQAHNVGNSGVDVDIGFLSFLSNGAWDSTDTTHSKYGFGADATLSYGVDRQRLLTLELAGNATLEQHYRKG**

**IraqBC**  **FSGSGAKLGYQAHNVGNSGVDVDIGFLSFLSNGAWDSTDTTHSKYGFGADATLSYGVDRQRLLTLELAGNATLEQNYLKG**

**BosniaAC**  **FSGSGAKLGYQAHNVGNSGVDVDIGFLSFLSNGAWDSTDTTHSKYGFGADATLSYGVDRQRLLTLELAGNATLEQNYLKG**

**Fribourg-BlancC** **FSGSGAKLGYQAHNVGNSGVDVDIGFLSFLSNGAWDSTDATHSKYGFGADATLSYGVDRQRLLTLELAGNATLEQHYRKG**

250 260 270 280 290 300 310 320

....|....|....|....|....|....|....|....|....|....|....|....|....|....|....|....|

**NicholsC**  **TEDSKNENKTALLWGVGGRLTLEPGAGFRFSFALDAGNQHQSNAHAQTQERAILKAREVFRRVEGKLVQNLPNIMMPPGI**

**NicholsD**  **TEDSKNENKTALLWGVGGRLTLEPGAGFRFSFALDAGNQHQSNAHAQTQERAILKAREVFRRVEGKLVQNLPNIMMPPGI**

**Bal3C**  **TEDSKNENKTALLWGVGGRLTLEPGAGFRFSFALDAGNQHQSNAHAQTQERAILKAREVFRRVEGKLVQNLPNIMMPPGI**

**Sea81-4C**  **TEDSKNENKTALLWGVGGRLTLEPGAGFRFSFALDAGNQHQSNAHAQTQERAILKAREVFRRVEGKLVQNLPNIMMPPGI**

**Street14C TEDSKNENKTALLWGVGSRLTLEPGAGFRFSFALDAGNQHQSNAHAQTQERAILKAREVFRRVEGKLVQNLPNIMMPPGI**

**MexicoAC**  **TEDSTNENKTALLWGVGGRLTLEPGAGFRFSFALDAGNQHQSNAHAQTQERAILKAREVFRRVEGKLVQNLPNIMMPPGI**

**GauthierC**  **TEDSTNENKTALLWGVGGRLTLEPGAGFRFSFALDAGNQHQSNADAQTQKERVSLAGEVFGQVVGKLVQNLPNIMMPLGI**

**GauthierD**  **TEDSTNENKTALLWGVGGRLTLEPGAGFRFSFALDAGNQHQSNADAQTQKERVSLAGEVFGQVVGKLVQNLPNIMMPLGI**

**CDC2C**  **TEDSTNENKTALLWGVGGRLTLEPGAGFRFSFALDAGNQHQSNADAQTQKERVSLAGEVFGQVVGKLVQNLPNIMMPLGI**

**CDC2D**  **TEDSTNENKTALLWGVGGRLTLEPGAGFRFSFALDAGNQHQSNADAQTQKERVSLAGEVFGQVVGKLVQNLPNIMMPLGI**

**SamoaDC**  **TEDSTNENKTALLWGVGGRLTLEPGAGFRFSFALDAGNQHQSNAHAQTQKERVSLAGEVFGQVVGKLVQNLPNIMMPLGI**

**IraqBC**  **TEDPKNENKTALLWGVGGRLTLEPGAGFRFSFALDAGNQHQSNADAQTQEERVSLAGEVFGQVVGKLVQNLPNIMMPLGI**

**BosniaAC**  **TEDPKNENKTALLWGVGGRLTLEPGAGFRFSFALDAGNQHQSNADAQTQKERVSLAGEVFGQVVGKLVQNLPNIMMPLGI**

**Fribourg-BlancC** **TEDSTNENKTALLWGVGGRLTLEPGAGFRFSFALDAGNQHQSDTDAQTQKERVSLAGEVFGRVVEKLVQNLLNIMMPLGI**

330 340 350 360 370 380 390 400

....|....|....|....|....|....|....|....|....|....|....|....|....|....|....|....|

**NicholsC**  **TEQTTLIEMVGLAALIAEGTLGSAIQTVLAAGALAALVSQLVPNIEQGVRDVFRSSDPRVVTAKLLAFLERAPMNALNID**

**NicholsD**  **TEQTTLIEMVGLAALIAEGTLGSAIQTVLAAGALAALVSQLVPNIEQGVRDVFRSSDPRVVTAKLLAFLERAPMNALNID**

**Bal3C**  **TEQTTLIEMVGLAALIAEGTLGSAIQTVLAAGALAALVSQLVPNIEQGVRDVFRSSDPRVVTAKLLAFLERAPMNALNID**

**Sea81-4C**  **TEQTTLIEMVGLAALIAEGTLGSAIQTVLAAGALAALVSQLVPNIEQGVRDVFRSSDPRVVTAKLLAFLERAPMNALNID**

**Street14C TEQTTLIEMVGLAALIAEGTLGSAIQTVLAAGALAALVSQLVPNIEQGVRDVFRSSDPRVVTAKLLAFLERAPMNALNID**

**MexicoAC**  **TEQTTLIEMVGLAALIAEGTLGSAIQTVLAAGALAALVSQLVPNIEQGVRDVFRSSDPRVVTAKLLAFLERAPMNALNID**

**GauthierC**  **TEQTTLIEMVGLAALIAEGTLGSAIQTVLAAGALAALVSQLVPHIEQGVRDVFRSSDPRVVTAKLLAFLERAPMNALNID**

**GauthierD**  **TEQTTLIEMVGLAALIAEGTLGSAIQTVLAAGALAALVSQLVPHIEQGVRDVFRSSDPRVVTAKLLAFLERAPMNALNID**

**CDC2C**  **TEQTTLIEMVGLAALIAEGTLGSAIQTVLAAGALAALVSQLVPHIEQGVRDVFRSSDPRVVTAKLLAFLERAPMNALNID**

**CDC2D**  **TEQTTLIEMVGLAALIAEGTLGSAIQTVLAAGALAALVSQLVPHIEQGVRDVFRSSDPRVVTAKLLAFLERAPMNALNID**

**SamoaDC**  **TEQTTLIEMVGLAALIAEGTLGSAIQTVLAAGALAALVSQLVPHIEQGVRDVFRSSDPRVVTAKLLAFLERAPMNALNID**

**IraqBC**  **TEQTTLIEMVGLAALIAEGTLGSAIQTVLAAGALAALVSQLVPHIEQGVRDVFRSSDPRVVTAKLLAFLERAPMNALNID**

**BosniaAC**  **TEQTTLIEMVGLAALIAEGTLGSAIQTVLAAGALAALVSQLVPHIEQGVRDVFRSSDPRVVTAKLLAFLERAPMNALNID**

**Fribourg-BlancC** **TEQTTLIEMVGLAALIAEGTLGSAIQTVPAAGVPAALVSQLVPHIEQGVRDVFRSSDPRVVTAKLLAFLERAPMNALNID**

410 420 430 440 450 460 470 480

....|....|....|....|....|....|....|....|....|....|....|....|....|....|....|....|

**NicholsC**  **ALLRMQWKWLSSGIYFATAGTNIFGKRVFATTRAHYFDFAGFLKLETKSGDPYTHLLTGLNAGVEARVYIPLTYIRYRNN**

**NicholsD**  **ALLRMQWKWLSSGIYFATAGTNIFGKRVFATTRAHYFDFAGFLKLETKSGDPYTHLLTGLNAGVEARVYIPLTYIRYRNN**

**Bal3C**  **ALLRMQWKWLSSGIYFATAGTNIFGKRVFATTRAHYFDFAGFLKLETKSGDPYTHLLTGLNAGVEARVYIPLTYVFYRNN**

**Sea81-4C**  **ALLRMQWKWLSSGIYFATAGTNIFGKRVFATTRAHYFDFAGFLKLETKSGDPYTHLLTGLNAGVEARVYIPLTYVFYRNN**

**Street14C ALLRMQWKWLSSGIYFATAGTNIFGKRVFATTRAHYFDFAGFLKLETKSGDPYTHLLTGLNAGVEARVYIPLTYVFYRNN**

**MexicoAC**  **ALLRMQWKWLSSGIYFATAGTNIFGKRVFATTRAHYFDFAGFLKLETKSGDPYTHLLTGLNAGVEARVYIPLTYVFYRNN**

**GauthierC**  **ALLRMQWKWLSSGIYFATAGTNIFGKRVFATTRAHYFDFAGFLKLETKSGDPYTHLLTGLNAGVEARVYIPLTYVFYKNN**

**GauthierD**  **ALLRMQWKWLSSGIYFATAGTNIFGKRVFATTRAHYFDFAGFLKLETKSGDPYTHLLTGLNAGVEARVYIPLTYVFYKNN**

**CDC2C**  **ALLRMQWKWLSSGIYFATAGTNIFGKRVFATTRAHYFDFAGFLKLETKSGDPYTHLLTGLNAGVEARVYIPLTYVFYKNN**

**CDC2D**  **ALLRMQWKWLSSGIYFATAGTNIFGKRVFATTRAHYFDFAGFLKLETKSGDPYTHLLTGLNAGVEARVYIPLTYVFYKNN**

**SamoaDC**  **ALLRMQWKWLSSGIYFATAGTNIFGKRVFATTRAHYFDFAGFLKLETKSGDPYTHLLTGLNAGVEARVYIPLTYVFYKNN**

**IraqBC**  **ALLRMQWKWLSSGIYFATAGTNIFGKRVFATTRAHYFDFAGFLKLETKSGDPYTHLLTGLNAGVEARVYIPLTYVFYKNN**

**BosniaAC**  **ALLRMQWKWLSSGIYFATAGTNIFGKRVFATTRAHYFDFAGFLKLETKSGDPYTHLLTGLNAGVEARVYIPLTYVFYKNN**

**Fribourg-BlancC** **ALLRMQWKWLSSGIYFATAGTNIFGKRVFATTRAHYFDFAGFLKLETKSGDPYTHLLTGLNAGVEARVYIPLTYVFYKNN**

490 500 510 520 530 540 550 560

....|....|....|....|....|....|....|....|....|....|....|....|....|....|....|....|

**NicholsC**  **GGYELNGAVPPGTINMPILGKAWCSYRIPLGSHAWLAPHTSVLGTTNRFNIINPAGNLLNERALQYQVGLTFSPFEKVEL**

**NicholsD**  **GGYELNGAVPPGTINMPILGKAWCSYRIPLGSHAWLAPHTSVLGTTNRFNIINPAGNLLNERALQYQVGLTFSPFEKVEL**

**Bal3C**  **GGYELNRVVPSGIINMPILGKAWCSYRIPLGSHAWLAPHTSVLGTTNRFNIINAAGNLLNERALQYQVGLTFSPFEKVEL**

**Sea81-4C**  **GGYELNRVVPSGIINMPILGKAWCSYRIPLGSHAWLAPHTSVLGTTNRFNIINAAGNLLNERALQYQVGLTFSPFEKVEL**

**Street14C GGYELNGAVPPGTINMPILGKAWCSYRIPLGSHAWLTPHTSVLGTTNRFNVINPAGNLLNERALQYQVGLTFSPFEKVEL**

**MexicoAC**  **GGYELNRVVPSGIINMPILGKAWCSYRIPLGSHAWLAPHTSVLGTTNRFNIINAAGNLLNERALQYQVGLTFSPFEKVEL**

**GauthierC**  **GGYPLNGVVPSGTINMPILGKAWCSYRIPLGSHAWLAPHTSVLGTTNRFNIINAAGNLVNERALQYQVGLTFSPFEKVEL**

**GauthierD**  **GGYPLNGVVPSGTINMPILGKAWCSYRIPLGSHAWLAPHTSVLGTTNRFNIINAAGNLVNERALQYQVGLTFSPFEKVEL**

**CDC2C**  **GGHPLNGVVPSGTINMPILGKAWCSYRIPLGSHAWLAPHTSVLGTTNRFNIINAAGNLVNERALQYQVGLTFSPFEKVEL**

**CDC2D**  **GGHPLNGVVPSGTINMPILGKAWCSYRIPLGSHAWLAPHTSVLGTTNRFNIINAAGNLVNERALQYQVGLTFSPFEKVEL**

**SamoaDC**  **GGYPLNGVVPSGTINMPILGKAWCSYRIPLGSHAWLAPHTSVLGTTNRFNIINAAGNLVNERALQYQVGLTFSPFEKVEL**

**IraqBC**  **GGYELNGVVPPGIINMPILGKAWCSYRIPLGSHAWLAPHTSVLGTTNRFNIINAAGNLVNERALQYQVGLTFSPFEKVEL**

**BosniaAC**  **GGYELNGVVPPGIINMPILGKAWCSYRIPLGSHAWLAPHTSVLGTTNRFNIINAAGNLVNERALQYQVGLTFSPFEKVEL**

**Fribourg-BlancC** **GGHLLDGVVPLGTINMPILGKAWCSYRIPLGSHAWLAPHTSVLGTTNRFNIINAAGNLVNERALQYQVGLTFSPFEKVEL**

570 580 590

....|....|....|....|....|....|....|...

**NicholsC**  **SAQWEQGVLADAPYMGIAESIWSERHFGTLVCGMKVTW**

**NicholsD**  **SAQWEQGVLADAPYMGIAESIWSERHFGTLVCGMKVTW**

**Bal3C**  **SAQWEQGVLSDVPYMGIAESIWSERHFGTLVCGMKVTW**

**Sea81-4C**  **SAQWEQGVLSDVPYMGIAESIWSERHFGTLVCGMKVTW**

**Street14C SAQWEQGVLADAPYMGITQSIGSDRHFGTLVCGMKVTW**

**MexicoAC**  **SAQWEQGVLADAPYMGITQSIGSDRHFGTLVCGMKVTW**

**GauthierC**  **SAQWEQGVLSDVPYMGITQSIWSERHFGTFVCGMKVTW**

**GauthierD**  **SAQWEQGVLSDVPYMGITQSIWSERHFGTFVCGMKVTW**

**CDC2C**  **SAQWEQGVLSDVPYMGITQSIWSERHFGTFVCGMKVTW**

**CDC2D**  **SAQWEQGVLSDVPYMGITQSIWSERHFGTLVCGMKVTW**

**SamoaDC**  **SAQWEQGVLSDVPYMGITQSIWSERHFGTFVCGMKVTW**

**IraqBC**  **SAQWEQGVLSDVPYMGIAESIWSERHFGTLVCGMKVTW**

**BosniaAC**  **SAQWEQGVLSDVPYMGIAESIWSERHFGTLVCGMKVTW**

**Fribourg-BlancC** **SAQWEQGVLSDVPYMGIAESIWSERHFGTFVCGMKVTW**

10 20 30 40 50 60 70 80

....|....|....|....|....|....|....|....|....|....|....|....|....|....|....|....|

**NicholsC**  **gtgggcaggcaggtgatgcaagcgggggtacttgcgggcatggtatgtgctgcttctggttatgcaggcgtactcactcc**

**NicholsD**  **gtgggcaggcaggtgatgcaagcgggggtacttgcgggcatggtatgtgctgcttctggttatgcaggcgtactcactcc**

**Bal3C**  **gtgggcaggcaggtgatgcaagcgggggtacttgcgggcatggtatgtgctgcttctggttatgcaggcgtactcactcc**

**Sea81-4C**  **gtgggcaggcaggtgatgcaagcgggggtacttgcgggcatggtatgtgctgcttctggttatgcaggcgtactcactcc**

**Street14C gtgggcaggcaggtgatgcaagcgggggtacttgcgggcatggtatgtgctgcttctggttatgcaggcgtactcactcc**

**MexicoAC**  **gtgggcaggcaggtgatgcaagcgggggtacttgcgggcatggtatgtgctgcttctggttatgcaggcgtactcactcc**

**GauthierC**  **gtgggcaagcaggtgatgcaagcgggggtacttgcgggcatggtatgtgctgcttctggttatgcaggcgtactcactcc**

**CDC2C**  **gtgggcaagcaggtgatgcaagcgggggtacttgcgggcatggtatgtgctgcttctggttatgcaggcgtactcactcc**

**GauthierD**  **gtgggcaagcaggtgatgcaagcgggggtacttgcgggcatggtatgtgctgcttctggttatgcaggcgtactcactcc**

**CDC2D**  **gtgggcaagcaggtgatgcaagcgggggtacttgcgggcatggtatgtgctgcttctggttatgcaggcgtactcactcc**

**SamoaDC**  **gtgggcaagcaggtgatgcaagcgggggtacttgcgggcatggtatgtgctgcttctggttatgcaggcgtactcactcc**

**Fribourg-BlancC** **gtgggcaagcaggtgatgcaagcgggggtacttgcgggcatggtatgtgctgcttctggttatgcaggcgtactcactcc**

**IraqBC**  **gtgggcaagcaggtgatgcaagcgggggtacttgcgggcatggtatgtgctgcttctggttatgcaggcgtactcactcc**

**BosniaAC**  **gtgggcaagcaggtgatgcaagcgggggtacttgcgggcatggtatgtgctgcttctggttatgcaggcgtactcactcc**

90 100 110 120 130 140 150 160

....|....|....|....|....|....|....|....|....|....|....|....|....|....|....|....|

**NicholsC**  **gcaggtcagtggcacagcccagctccagtggggcattgcgttccagaagaatccacgcactggcccgggcaagcacaccc**

**NicholsD**  **gcaggtcagtggcacagcccagctccagtggggcattgcgttccagaagaatccacgcactggcccgggcaagcacaccc**

**Bal3C**  **gcaggtcagtggcacagcccagctccagtggggcattgcgttccagaagaatccacgcactggcccgggcaagcacaccc**

**Sea81-4C**  **gcaggtcagtggcacagcccagctccagtggggcattgcgttccagaagaatccacgcactggcccgggcaagcacaccc**

**Street14C gcaggtcagtggcacagcccagctccagtggggcattgcgttccagaagaatccacgcactggcccgggcaagcacaccc**

**MexicoAC**  **gcaggtcagtggcacagcccagctccagtggggcattgcgttccagaagaatccacgcactggcccgggcaagcacaccc**

**GauthierC**  **gcaggtcagtggcacagcccagctccagtggggcattgcgttccagaagaatccacacactgtcccgggcgagcacaccc**

**CDC2C**  **gcaggtcagtggcacagcccagctccagtggggcattgcgttccagaagaatccacacactgtcccgggcgagcacaccc**

**GauthierD**  **gcaggtcagtggcacagcccagctccagtggggcattgcgttccagaagaatccacacactgtcccgggcaagcacaccc**

**CDC2D**  **gcaggtcagtggcacagcccagctccagtggggcattgcgttccagaagaatccacacactgtcccgggcgagcacaccc**

**SamoaDC**  **gcaggtcagtggcacagcccagctccagtggggcattgcgttccagaagaatccacacactgtcccgggcaagcacaccc**

**Fribourg-BlancC** **gcaggtcagtggcacagcccagctccagtggggcattgcgttccagaagaatccacacactgtcccgggcaagcacaccc**

**IraqBC**  **gcaggtcagtggcacagcccagctccagtggggcattgcgttccagaagaatccacacactgtcccgggcaagcacaccc**

**BosniaAC**  **gcaggtcagtggcgcagcccagctccagtggggcattgcgttccagaagaatccacacactgtcccgggcaagcacaccc**

170 180 190 200 210 220 230 240

....|....|....|....|....|....|....|....|....|....|....|....|....|....|....|....|

**NicholsC**  **atgggtttcgcactaccaatagtctgactatttccctgccgttggtgtcaaagcacacccacacccgccgaggggaggca**

**NicholsD**  **atgggtttcgcactaccaatagtctgactatttccctgccgttggtgtcaaagcacacccacacccgccgaggggaggca**

**Bal3C**  **atgggtttcgcactaccaatagtctgactatttccctgccgttggtgtcaaagcacacccacacccgccgaggggaggca**

**Sea81-4C**  **atgggtttcgcactaccaatagtctgactatttccctgccgttggtgtcaaagcacacccacacccgccgaggggaggca**

**Street14C atgggtttcgcactaccaatagtctgactatttccctgccgttggtgtcaaagcacacccacacccgccgaggggaggca**

**MexicoAC**  **atgggtttcgcactaccaatagtctgactatttccctgccgttggtgtcaaagcacacccacacccgccgaggggaggca**

**GauthierC**  **atgggtttcgcactaccaatagtctgactatttccctgccgttggtgtcaaagcacacccacacccgccgaggggaggca**

**CDC2C**  **atgggtttcgcactaccaatagtctgactatttccctgccgttggtgtcaaagcacacccacacccgccgaggggaggca**

**GauthierD**  **atgggtttcgcactaccaatagtctgactatttccctgccgttggtgtcaaagcacacccacacccgccgaggggaggca**

**CDC2D**  **atgggtttcgcactaccaatagtctgactatttccctgccgttggtgtcaaagcacacccacacccgccgaggggaggca**

**SamoaDC**  **atgggtttcgcactaccaatagtctgactatttccctgccgttggtgtcaaagcacacccacacccgccgaggggaggca**

**Fribourg-BlancC** **atgggtttcgcactaccaatagtctgactatttccctgccgttggtgtcaaagcacacccacacccgccgaggggaggca**

**IraqBC**  **atgggtttcgcactaccaatagtctgactatttccctgccgttggtgtcaaagcacacccacacccgccgaggggaggca**

**BosniaAC**  **atgggtttcgcactaccaatagtctgactatttccctgccgttggtgtcaaagcacacccacacccgccgaggggaggca**

250 260 270 280 290 300 310 320

....|....|....|....|....|....|....|....|....|....|....|....|....|....|....|....|

**NicholsC**  **cgctcaggggtgtgggcacagctgcagctgaaggacctggcagtagagcttgcgtcttctaaaagctcaacggccctgtc**

**NicholsD**  **cgctcaggggtgtgggcacagctgcagctgaaggacctggcagtagagcttgcgtcttctaaaagctcaacggccctgtc**

**Bal3C**  **cgctcaggggtgtgggcacagctgcagctgaaggacctggcagtagagcttgcgtcttctaaaagctcaacggccctgtc**

**Sea81-4C**  **cgctcaggggtgtgggcacagctgcagctgaaggacctggcagtagagcttgcgtcttctaaaagctcaacggccctgtc**

**Street14C cgctcaggggtgtgggcacagctgcagctgaaggacctggcagtagagcttgcgtcttctaaaagctcaacggccctgtc**

**MexicoAC**  **cgctcaggggtgtgggcacagctgcagctgaaggacctggcagtagagcttgcgtcttctaaaagctcaacggccctgtc**

**GauthierC**  **cgctcaggggtgtgggcacagctgcagctgaaggacctggcagtagagcttgcgtcttctaaaagctcaacggccctgtc**

**CDC2C**  **cgctcaggggtgtgggcacagctgcagctgaaggacctggcagtagagcttgcgtcttctaaaagctcaacggccctgtc**

**GauthierD**  **cgctcaggggtgtgggcacagctgcagctgaaggacctggcagtagagcttgcgtcttctaaaagctcaacggccctgtc**

**CDC2D**  **cgctcaggggtgtgggcacagctgcagctgaaggacctggcagtagagcttgcgtcttctaaaagctcaacggccctgtc**

**SamoaDC**  **cgctcaggggtgtgggcacagctgcagctgaaggacctggcagtagagcttgcgtcttctaaaagctcaacggccctgtc**

**Fribourg-BlancC** **cgctcaggggtgtgggcacagctgcagctgaaggacctggcagtagagcttgcgtcttctaaaagctcaacggccctgtc**

**IraqBC**  **cgctcaggggtgtgggcacagctgcagctgaaggacctggcagtagagcttgcgtcttctaaaagctcaacggccctgtc**

**BosniaAC**  **cgctcaggggtgtgggcacagctgcagctgaaggacctggcagtagagcttgcgtcttctaaaagctcaacggccctgtc**

330 340 350 360 370 380 390 400

....|....|....|....|....|....|....|....|....|....|....|....|....|....|....|....|

**NicholsC**  **ctttaccaaacctaccgcttccttccaggcaaccctgcactgttatggggcctacctgacagtgggtaccagtccttcct**

**NicholsD**  **ctttaccaaacctaccgcttccttccaggcaaccctgcactgttatggggcctacctgacagtgggtaccagtccttcct**

**Bal3C**  **ctttaccaaacctaccgcttccttccaggcaaccctgcactgttatggggcctacctgacagtgggtaccagtccttcct**

**Sea81-4C**  **ctttaccaaacctaccgcttccttccaggcaaccctgcactgttatggggcctacctgacagtgggtaccagtccttcct**

**Street14C ctttaccaaacctaccgcttccttccaggcaaccctgcactgttatggggcctacctgacagtgggtaccagtccttcct**

**MexicoAC**  **ctttaccaaacctaccgcttccttccaggcaaccctgcactgttatggggcctacctgacagtgggtaccagtccttcct**

**GauthierC**  **ctttaccaaacctaccgcttccttccaggcaaccctgcactgttatggggcctacctgacagtgggtaccagtccttcct**

**CDC2C**  **ctttaccaaacctaccgcttccttccaggcaaccctgcactgttatggggcctacctgacagtgggtaccagtccttcct**

**GauthierD**  **ctttaccaaacctaccgcttccttccaggcaaccctgcactgttatggggcctacctgacagtgggtaccagtccttcct**

**CDC2D**  **ctttaccaaacctaccgcttccttccaggcaaccctgcactgttatggggcctacctgacagtgggtaccagtccttcct**

**SamoaDC**  **ctttaccaaacctaccgcttccttccaggcaaccctgcactgttatggggcctacctgacagtgggtaccagtccttcct**

**Fribourg-BlancC** **ctttaccaaacctaccgcttccttccaggcaaccctgcactgttatggggcctacctgacagtgggtaccagtccttcct**

**IraqBC**  **ctttaccaaacctaccgcttccttccaggcaaccctgcactgttatggggcctacctgacagtgggtaccagtccttcct**

**BosniaAC**  **ctttaccaaacctaccgcttccttccaggcaaccctgcactgttatggggcctacctgacagtgggtaccagtccttcct**

410 420 430 440 450 460 470 480

....|....|....|....|....|....|....|....|....|....|....|....|....|....|....|....|

**NicholsC**  **gtgtggttaactttgcccagctgtggaaaccctttgtcacccgtgcctattcagaaaaggacactcgctatgcccctggt**

**NicholsD**  **gtgtggttaactttgcccagctgtggaaaccctttgtcacccgtgcctattcagaaaaggacactcgctatgcccctggt**

**Bal3C**  **gtgtggttaactttgcccagctgtggaaaccctttgtcacccgtgcctattcagaaaaggacactcgctatgcccctggt**

**Sea81-4C**  **gtgtggttaactttgcccagctgtggaaaccctttgtcacccgtgcctattcagaaaaggacactcgctatgcccctggt**

**Street14C gtgtggttaactttgcccagctgtggaaaccctttgtcacccgtgcctattcagaaaaggacactcgctatgcccctggt**

**MexicoAC**  **gtgtggttaactttgcccagctgtggaaaccctttgtcacccgtgcctattcagaaaaggacactcgctatgcccctggt**

**GauthierC**  **gtgtggttaactttgcccagctgtggaaaccctttgtcacccgtgcctattcagaaaaggacactcgctatgcccctggt**

**CDC2C**  **gtgtggttaactttgcccagctgtggaaaccctttgtcacccgtgcctattcagaaaaggacactcgctatgcccctggt**

**GauthierD**  **gtgtggttaactttgcccagctgtggaaaccctttgtcacccgtgcctattcagaaaaggacactcgctatgcccctggt**

**CDC2D**  **gtgtggttaactttgcccagctgtggaaaccctttgtcacccgtgcctattcagaaaaggacactcgctatgcccctggt**

**SamoaDC**  **gtgtggttaactttgcccagctgtggaaaccctttgtcacccgtgcctattcagaaaaggacactcgctatgcccctggt**

**Fribourg-BlancC** **gtgtggttaactttgcccagctgtggaaaccctttgtcacccgtgcctattcagaaaaggacactcgctatgcccctggt**

**IraqBC**  **gtgtggttaactttgcccagctgtggaaaccctttgtcacccgtgcctattcagaaaaggacactcgctatgcccctggt**

**BosniaAC**  **gtgtggttaactttgcccagctgtggaaaccctttgtcacccgtgcctattcagaaaaggacactcgctatgcccctggt**

490 500 510 520 530 540 550 560

....|....|....|....|....|....|....|....|....|....|....|....|....|....|....|....|

**NicholsC**  **ttctccggctccggggcaaaactcggctaccaggcccacaatgtgggaaacagcggagtagatgtggacatcggtttcct**

**NicholsD**  **ttctccggctccggggcaaaactcggctaccaggcccacaatgtgggaaacagcggagtagatgtggacatcggtttcct**

**Bal3C**  **ttctccggctccggggcaaaactcggctaccaggcccacaatgtgggaaacagcggagtagatgtggacatcggtttcct**

**Sea81-4C**  **ttctccggctccggggcaaaactcggctaccaggcccacaatgtgggaaacagcggagtagatgtggacatcggtttcct**

**Street14C ttctccggctccggggcaaaactcggctaccaggcccacaatgtgggaaacagcggagtagatgtggacatcggtttcct**

**MexicoAC**  **ttctccggctccggggcaaaactcggctaccaggcccacaatgtgggaaacagcggagtagatgtggacatcggtttcct**

**GauthierC**  **ttctccggctccggggcaaaactcggctaccaggcccacaatgtgggaaacagcggagtagatgtggacatcggtttcct**

**CDC2C**  **ttctccggctccggggcaaaactcggctaccaggcccacaatgtgggaaacagcggagtagatgtggacatcggtttcct**

**GauthierD**  **ttctccggctccggggcaaaactcggctaccaggcccacaatgtgggaaacagcggagtagatgtggacatcggtttcct**

**CDC2D**  **ttctccggctccggggcaaaactcggctaccaggcccacaatgtgggaaacagcggagtagatgtggacatcggtttcct**

**SamoaDC**  **ttctccggctccggggcaaaactcggctaccaggcccacaatgtgggaaacagcggagtagatgtggacatcggtttcct**

**Fribourg-BlancC** **ttctccggctccggggcaaaactcggctaccaggcccacaatgtgggaaacagcggagtagatgtggacatcggtttcct**

**IraqBC**  **ttctccggctccggggcaaaactcggctaccaggcccacaatgtgggaaacagcggagtagatgtggacatcggtttcct**

**BosniaAC**  **ttctccggctccggggcaaaactcggctaccaggcccacaatgtgggaaacagcggagtagatgtggacatcggtttcct**

570 580 590 600 610 620 630 640

....|....|....|....|....|....|....|....|....|....|....|....|....|....|....|....|

**NicholsC**  **ctccttcctttccaatggtgcctgggatagtactgacaccacgcacagcaagtatggcttcggggccgatgcaacgcttt**

**NicholsD**  **ctccttcctttccaatggtgcctgggatagtactgacaccacgcacagcaagtatggcttcggggccgatgcaacgcttt**

**Bal3C**  **ctccttcctttccaatggtgcctgggatagtactgacaccacgcacagcaagtatggcttcggggccgatgcaacgcttt**

**Sea81-4C**  **ctccttcctttccaatggtgcctgggatagtactgacaccacgcacagcaagtatggcttcggggccgatgcaacgcttt**

**Street14C ctccttcctttccaatggtgcctgggatagtactgacaccacgcacagcaagtatggcttcggggccgatgcaacgcttt**

**MexicoAC**  **ctccttcctttccaatggtgcctgggatagtactgacaccacgcacagcaagtatggcttcggggccgatgcaacgcttt**

**GauthierC**  **ctccttcctttccaatggtgcctgggatagtactgacaccacgcacagcaagtatggcttcggggccgatgcaacgcttt**

**CDC2C**  **ctccttcctttccaatggtgcctgggatagtactgacaccacgcacagcaagtatggcttcggggccgatgcaacgcttt**

**GauthierD**  **ctccttcctttccaatggtgcctgggatagtactgacaccacgcacagcaagtatggcttcggggccgatgcaacgcttt**

**CDC2D**  **ctccttcctttccaatggtgcctgggatagtactgacaccacgcacagcaagtatggcttcggggccgatgcaacgcttt**

**SamoaDC**  **ctccttcctttccaatggtgcctgggatagtactgacaccacgcacagcaagtatggcttcggggccgatgcaacgcttt**

**Fribourg-BlancC** **ctccttcctttccaatggtgcctgggatagtactgacgccacgcacagcaagtatggcttcggggccgatgcaacgcttt**

**IraqBC**  **ctccttcctttccaatggtgcctgggatagtactgacaccacgcacagcaagtatggcttcggggccgatgcaacgcttt**

**BosniaAC**  **ctccttcctttccaatggtgcctgggatagtactgacaccacgcacagcaagtatggcttcggggccgatgcaacgcttt**

650 660 670 680 690 700 710 720

....|....|....|....|....|....|....|....|....|....|....|....|....|....|....|....|

**NicholsC**  **cctatggcgtcgaccgtcagcggctgcttacgttggagctggcagggaatgccacactggaccagaactacgttaagggt**

**NicholsD**  **cctatggcgtcgaccgtcagcggctgcttacgttggagctggcagggaatgccacactggaccagaactacgttaagggt**

**Bal3C**  **cctatggcgtcgaccgtcagcggctgcttacgttggagctggcagggaatgccacactggaccagaactacgttaagggt**

**Sea81-4C**  **cctatggcgtcgaccgtcagcggctgcttacgttggagctggcagggaatgccacactggaccagaactacgttaagggt**

**Street14C cctatggcgtcgaccgtcagcggctgcttacgttggagctggcagggaatgccacactggaccagaactacgttaagggt**

**MexicoAC**  **cctatggcgtcgaccgtcagcggctgcttacgttggagctggcagggaatgccacactggagcagcactaccgtaagggt**

**GauthierC**  **cctatggcgtcgaccgtcagcggctgcttacgttggagctggcagggaatgccacactggagcagcactaccgtaagggt**

**CDC2C**  **cctatggcgtcgaccgtcagcggctgcttacgttggagctggcagggaatgccacactggagcagcactaccgtaagggt**

**GauthierD**  **cctatggcgtcgaccgtcagcggctgcttacgttggagctggcagggaatgccacactggagcagcactaccgtaagggt**

**CDC2D**  **cctatggcgtcgaccgtcagcggctgcttacgttggagctggcagggaatgccacactggagcagcactaccgtaagggt**

**SamoaDC**  **cctatggcgtcgaccgtcagcggctgcttacgttggagctggcagggaatgccacactggagcagcactaccgtaagggt**

**Fribourg-BlancC** **cctatggcgtcgaccgtcagcggctgcttacgttggagctggcagggaatgccacactggagcagcactaccgtaagggt**

**IraqBC**  **cctatggcgtcgaccgtcagcggctgcttacgttggagctggcagggaatgccacactggagcagaactacctcaagggt**

**BosniaAC**  **cctatggcgtcgaccgtcagcggctgcttacgttggagctggcagggaatgccacactggagcagaactacctcaagggt**

730 740 750 760 770 780 790 800

....|....|....|....|....|....|....|....|....|....|....|....|....|....|....|....|

**NicholsC**  **accgaagactccaagaacgaaaacaaaacagcactcctgtggggagtaggaggccgactcaccctcgaaccaggcgccgg**

**NicholsD**  **accgaagactccaagaacgaaaacaaaacagcactcctgtggggagtaggaggccgactcaccctcgaaccaggcgccgg**

**Bal3C**  **accgaagactccaagaacgaaaacaaaacagcactcctgtggggagtaggaggccgactcaccctcgaaccaggcgccgg**

**Sea81-4C**  **accgaagactccaagaacgaaaacaaaacagcactcctgtggggagtaggaggccgactcaccctcgaaccaggcgccgg**

**Street14C accgaagactccaagaacgaaaacaaaacagcactcctgtggggagtaggaagccgactcaccctcgaaccaggcgccgg**

**MexicoAC**  **accgaagactccacgaacgaaaacaaaacagcactcctgtggggagtaggaggccgactcaccctcgaaccaggcgccgg**

**GauthierC**  **accgaagactccacgaacgaaaacaaaacagcactcctgtggggagtaggaggccgactcaccctcgaaccaggcgccgg**

**CDC2C**  **accgaagactccacgaacgaaaacaaaacagcactcctgtggggagtaggaggccgactcaccctcgaaccaggcgccgg**

**GauthierD**  **accgaagactccacgaacgaaaacaaaacagcactcctgtggggagtaggaggccgactcaccctcgaaccaggcgccgg**

**CDC2D**  **accgaagactccacgaacgaaaacaaaacagcactcctgtggggagtaggaggccgactcaccctcgaaccaggcgccgg**

**SamoaDC**  **accgaagactccacgaacgaaaacaaaacagcactcctgtggggagtaggaggccgactcaccctcgaaccaggcgccgg**

**Fribourg-BlancC** **accgaagactccacgaacgaaaacaaaacagcactcctgtggggagtaggaggccgactcaccctcgaaccaggcgccgg**

**IraqBC**  **accgaagaccccaagaacgaaaacaaaacagcactcctgtggggagtaggaggccgactcaccctcgaaccaggcgccgg**

**BosniaAC**  **accgaagaccccaagaacgaaaacaaaacagcactcctgtggggagtaggaggccgactcaccctcgaaccaggcgccgg**

810 820 830 840 850 860 870 880

....|....|....|....|....|....|....|....|....|....|....|....|....|....|....|....|

**NicholsC**  **cttccgcttctccttcgccctcgacgccggtaaccaacaccagagtaacgcacatgctcagacccaagagagagctatcc**

**NicholsD**  **cttccgcttctccttcgccctcgacgccggtaaccaacaccagagtaacgcacatgctcagacccaagagagagctatcc**

**Bal3C**  **cttccgcttctccttcgccctcgacgccggtaaccaacaccagagtaacgcacatgctcagacccaagagagagctatcc**

**Sea81-4C**  **cttccgcttctccttcgccctcgacgccggtaaccaacaccagagtaacgcacatgctcagacccaagagagagctatcc**

**Street14C cttccgcttctccttcgccctcgacgccggtaaccaacaccagagtaacgcacatgctcagacccaagagagagctatcc**

**MexicoAC**  **cttccgcttctccttcgccctcgacgccggtaaccaacaccagagtaacgcacatgctcagacccaagagagagctatcc**

**GauthierC**  **cttccgcttctccttcgccctcgacgccggtaaccaacaccagagtaacgcagatgctcagacccaaaaagagagagtta**

**CDC2C**  **cttccgcttctccttcgccctcgacgccggtaaccaacaccagagtaacgcagatgctcagacccaaaaagagagagtta**

**GauthierD**  **cttccgcttctccttcgccctcgacgccggtaaccaacaccagagtaacgcagatgctcagacccaaaaagagagagtta**

**CDC2D**  **cttccgcttctccttcgccctcgacgccggtaaccaacaccagagtaacgcagatgctcagacccaaaaagagagagtta**

**SamoaDC**  **cttccgcttctccttcgccctcgacgccggtaaccaacaccagagtaacgcacatgctcagacccaaaaagagagagtta**

**Fribourg-BlancC** **cttccgcttctccttcgccctcgacgccggtaaccaacaccagagtgacacagatgctcagacccaaaaagagagagtta**

**IraqBC**  **cttccgcttctccttcgccctcgacgccggtaaccaacaccagagtaacgcagatgctcagacccaagaagagagagtta**

**BosniaAC**  **cttccgcttctccttcgccctcgacgccggtaaccaacaccagagtaacgcagatgctcagacccaaaaagagagagtta**

890 900 910 920 930 940 950 960

....|....|....|....|....|....|....|....|....|....|....|....|....|....|....|....|

**NicholsC**  **tcaaagcaagggaagtgtttagacgggtggaggggaaactcgtgcagaaccttcccaatatcatgatgccaccaggaatc**

**NicholsD**  **tcaaagcaagggaagtgtttagacgggtggaggggaaactcgtgcagaaccttcccaatatcatgatgccaccaggaatc**

**Bal3C**  **tcaaagcaagggaagtgtttagacgggtggaggggaaactcgtgcagaaccttcccaatatcatgatgccaccaggaatc**

**Sea81-4C**  **tcaaagcaagggaagtgtttagacgggtggaggggaaactcgtgcagaaccttcccaatatcatgatgccaccaggaatc**

**Street14C tcaaagcaagggaagtgtttagacgggtggaggggaaactcgtgcagaaccttcccaatatcatgatgccaccaggaatc**

**MexicoAC**  **tcaaagcaagggaagtgtttagacgggtggaggggaaactcgtgcagaaccttcccaatatcatgatgccaccaggaatc**

**GauthierC**  **gcctcgcaggggaagtgtttggacaggtggtggggaaactcgtgcagaaccttcccaatatcatgatgccactaggaatc**

**CDC2C**  **gcctcgcaggggaagtgtttggacaggtggtggggaaactcgtgcagaaccttcccaatatcatgatgccactaggaatc**

**GauthierD**  **gcctcgcaggggaagtgtttggacaggtggtggggaaactcgtgcagaaccttcccaatatcatgatgccactaggaatc**

**CDC2D**  **gcctcgcaggggaagtgtttggacaggtggtggggaaactcgtgcagaaccttcccaatatcatgatgccactaggaatc**

**SamoaDC**  **gcctcgcaggggaagtgtttggacaggtggtggggaaactcgtgcagaaccttcccaatatcatgatgccactaggaatc**

**Fribourg-BlancC** **gcctcgcaggggaagtgtttggacgggtggtggagaaactcgtgcagaaccttctcaatatcatgatgccactaggaatc**

**IraqBC**  **gcctcgcaggggaagtgtttggacaggtggtggggaaactcgtgcagaaccttcccaatatcatgatgccactaggaatc**

**BosniaAC**  **gcctcgcaggggaagtgtttggacaggtggtggggaaactcgtgcagaaccttcccaatatcatgatgccactaggaatc**

970 980 990 1000 1010 1020 1030 1040

....|....|....|....|....|....|....|....|....|....|....|....|....|....|....|....|

**NicholsC**  **accgaacaaaccactctcatagagatggtaggacttgctgctttgattgcagaaggaacgctcggcagcgccattcaaac**

**NicholsD**  **accgaacaaaccactctcatagagatggtaggacttgctgctttgattgcagaaggaacgctcggcagcgccattcaaac**

**Bal3C**  **accgaacaaaccactctcatagagatggtaggacttgctgctttgattgcagaaggaacgctcggcagcgccattcaaac**

**Sea81-4C**  **accgaacaaaccactctcatagagatggtaggacttgctgctttgattgcagaaggaacgctcggcagcgccattcaaac**

**Street14C accgaacaaaccactctcatagagatggtaggacttgctgctttgattgcagaaggaacgctcggcagcgccattcaaac**

**MexicoAC**  **accgaacaaaccactctcatagagatggtaggacttgctgctttgattgcagaaggaacgctcggcagcgccattcaaac**

**GauthierC**  **accgaacaaaccactctcatagagatggtaggacttgctgctttgattgcagaaggaacgctcggcagcgccattcaaac**

**CDC2C**  **accgaacaaaccactctcatagagatggtaggacttgctgctttgattgcagaaggaacgctcggcagcgccattcaaac**

**GauthierD**  **accgaacaaaccactctcatagagatggtaggacttgctgctttgattgcagaaggaacgctcggcagcgccattcaaac**

**CDC2D**  **accgaacaaaccactctcatagagatggtaggacttgctgctttgattgcagaaggaacgctcggcagcgccattcaaac**

**SamoaDC**  **accgaacaaaccactctcatagagatggtaggacttgctgctttgattgcagaaggaacgctcggcagcgccattcaaac**

**Fribourg-BlancC** **accgaacaaaccactctcatagagatggtaggacttgctgctttgattgcagaaggaacgctcggcagcgccattcaaac**

**IraqBC**  **accgaacaaaccactctcatagagatggtaggacttgctgctttgattgcagaaggaacgctcggcagcgccattcaaac**

**BosniaAC**  **accgaacaaaccactctcatagagatggtaggacttgctgctttgattgcagaaggaacgctcggcagcgccattcaaac**

1050 1060 1070 1080 1090 1100 1110 1120

....|....|....|....|....|....|....|....|....|....|....|....|....|....|....|....|

**NicholsC**  **cgtgctagccgctggcgcgctcgcggcgcttgtatcgcaacttgtaccgaacatagagcaaggagtacgtgatgtcttcc**

**NicholsD**  **cgtgctagccgctggcgcgctcgcggcgcttgtatcgcaacttgtaccgaacatagagcaaggagtacgtgatgtcttcc**

**Bal3C**  **cgtgctagccgctggcgcgctcgcggcgcttgtatcgcaacttgtaccgaacatagagcaaggagtacgtgatgtcttcc**

**Sea81-4C**  **cgtgctagccgctggcgcgctcgcggcgcttgtatcgcaacttgtaccgaacatagagcaaggagtacgtgatgtcttcc**

**Street14C cgtgctagccgctggcgcgctcgcggcgcttgtatcgcaacttgtaccgaacatagagcaaggagtacgtgatgtcttcc**

**MexicoAC**  **cgtgctagccgctggcgcgctcgcggcgcttgtatcgcaacttgtaccgaacatagagcaaggagtacgtgatgtcttcc**

**GauthierC**  **cgtgctagccgctggcgcgctcgcggcgcttgtatcgcaacttgtaccgcacatagagcaaggagtacgtgatgtcttcc**

**CDC2C**  **cgtgctagccgctggcgcgctcgcggcgcttgtatcgcaacttgtaccgcacatagagcaaggagtacgtgatgtcttcc**

**GauthierD**  **cgtgctagccgctggcgcgctcgcggcgcttgtatcgcaacttgtaccgcacatagagcaaggagtacgtgatgtcttcc**

**CDC2D**  **cgtgctagccgctggcgcgctcgcggcgcttgtatcgcaacttgtaccgcacatagagcaaggagtacgtgatgtcttcc**

**SamoaDC**  **cgtgctagccgctggcgcgctcgcggcgcttgtatcgcaacttgtaccgcacatagagcaaggagtacgtgatgtcttcc**

**Fribourg-BlancC** **cgtgccagccgctggcgtgcccgcggcgcttgtatcgcaacttgtaccgcacatagagcaaggagtacgtgatgtcttcc**

**IraqBC**  **cgtgctagccgctggcgcgctcgcggcgcttgtatcgcaacttgtaccgcacatagagcaaggagtacgtgatgtcttcc**

**BosniaAC**  **cgtgctagccgctggcgcgctcgcggcgcttgtatcgcaacttgtaccgcacatagagcaaggagtacgtgatgtcttcc**

1130 1140 1150 1160 1170 1180 1190 1200

....|....|....|....|....|....|....|....|....|....|....|....|....|....|....|....|

**NicholsC**  **gctcttccgatccaagagttgtcactgctaaacttctcgctttccttgagcgcgcacctatgaacgcgctcaacatagac**

**NicholsD**  **gctcttccgatccaagagttgtcactgctaaacttctcgctttccttgagcgcgcacctatgaacgcgctcaacatagac**

**Bal3C**  **gctcttccgatccaagagttgtcactgctaaacttctcgctttccttgagcgcgcacctatgaacgcgctcaacatagac**

**Sea81-4C**  **gctcttccgatccaagagttgtcactgctaaacttctcgctttccttgagcgcgcacctatgaacgcgctcaacatagac**

**Street14C gctcttccgatccaagagttgtcactgctaaacttctcgctttccttgagcgcgcacctatgaacgcgctcaacatagac**

**MexicoAC**  **gctcttccgatccaagagttgtcactgctaaacttctcgctttccttgagcgcgcacctatgaacgcgctcaacatagac**

**GauthierC**  **gctcttccgatccaagagttgtcactgctaaacttctcgctttccttgagcgcgcacctatgaacgcgctcaacatagac**

**CDC2C**  **gctcttccgatccaagagttgtcactgctaaacttctcgctttccttgagcgcgcacctatgaacgcgctcaacatagac**

**GauthierD**  **gctcttccgatccaagagttgtcactgctaaacttctcgctttccttgagcgcgcacctatgaacgcgctcaacatagac**

**CDC2D**  **gctcttccgatccaagagttgtcactgctaaacttctcgctttccttgagcgcgcacctatgaacgcgctcaacatagac**

**SamoaDC**  **gctcttccgatccaagagttgtcactgctaaacttctcgctttccttgagcgcgcacctatgaacgcgctcaacatagac**

**Fribourg-BlancC** **gctcttccgatccaagagttgtcactgctaaacttctcgctttccttgagcgcgcacctatgaacgcgctcaacatagac**

**IraqBC**  **gctcttccgatccaagagttgtcactgctaaacttctcgctttccttgagcgcgcacctatgaacgcgctcaacatagac**

**BosniaAC**  **gctcttccgatccaagagttgtcactgctaaacttctcgctttccttgagcgcgcacctatgaacgcgctcaacatagac**

1210 1220 1230 1240 1250 1260 1270 1280

....|....|....|....|....|....|....|....|....|....|....|....|....|....|....|....|

**NicholsC**  **gcgctcctgcgtatgcagtggaagtggctctcttctggcatatactttgccaccgcaggcactaatatctttggcaaacg**

**NicholsD**  **gcgctcctgcgtatgcagtggaagtggctctcttctggcatatactttgccaccgcaggcactaatatctttggcaaacg**

**Bal3C**  **gcgctcctgcgtatgcagtggaagtggctctcttctggcatatactttgccaccgcaggcactaatatctttggcaaacg**

**Sea81-4C**  **gcgctcctgcgtatgcagtggaagtggctctcttctggcatatactttgccaccgcaggcactaatatctttggcaaacg**

**Street14C gcgctcctgcgtatgcagtggaagtggctctcttctggcatatactttgccaccgcaggcactaatatctttggcaaacg**

**MexicoAC**  **gcgctcctgcgtatgcagtggaagtggctctcttctggcatatactttgccaccgcaggcactaatatctttggcaaacg**

**GauthierC**  **gcgctcctgcgtatgcagtggaagtggctctcttccggcatatactttgccaccgcaggcactaatatctttggcaaacg**

**CDC2C**  **gcgctcctgcgtatgcagtggaagtggctctcttccggcatatactttgccaccgcaggcactaatatctttggcaaacg**

**GauthierD**  **gcgctcctgcgtatgcagtggaagtggctctcttccggcatatactttgccaccgcaggcactaatatctttggcaaacg**

**CDC2D**  **gcgctcctgcgtatgcagtggaagtggctctcttccggcatatactttgccaccgcaggcactaatatctttggcaaacg**

**SamoaDC**  **gcgctcctgcgtatgcagtggaagtggctctcttccggcatatactttgccaccgcaggcactaatatctttggcaaacg**

**Fribourg-BlancC** **gcgctcctgcgtatgcagtggaagtggctctcttccggcatatactttgccaccgcaggcactaatatctttggcaaacg**

**IraqBC**  **gcgctcctgcgtatgcagtggaagtggctctcttccggcatatactttgccaccgcaggcactaatatctttggcaaacg**

**BosniaAC**  **gcgctcctgcgtatgcagtggaagtggctctcttccggcatatactttgccaccgcaggcactaatatctttggcaaacg**

1290 1300 1310 1320 1330 1340 1350 1360

....|....|....|....|....|....|....|....|....|....|....|....|....|....|....|....|

**NicholsC**  **cgtctttgctaccactcgtgcgcactactttgattttgccggattccttaagctcgaaaccaaaagcggtgacccctaca**

**NicholsD**  **cgtctttgctaccactcgtgcgcactactttgattttgccggattccttaagctcgaaaccaaaagcggtgacccctaca**

**Bal3C**  **cgtctttgctaccactcgtgcgcactactttgattttgccggattccttaagctcgaaaccaaaagcggtgacccctaca**

**Sea81-4C**  **cgtctttgctaccactcgtgcgcactactttgattttgccggattccttaagctcgaaaccaaaagcggtgacccctaca**

**Street14C cgtctttgctaccactcgtgcgcactactttgattttgccggattccttaagctcgaaaccaaaagcggtgacccctaca**

**MexicoAC**  **cgtctttgctaccactcgtgcgcactactttgattttgccggattccttaagctcgaaaccaaaagcggtgacccctaca**

**GauthierC**  **cgtctttgctaccactcgtgcgcactactttgattttgccggattccttaagctcgaaaccaaaagcggtgacccctaca**

**CDC2C**  **cgtctttgctaccactcgtgcgcactactttgattttgccggattccttaagctcgaaaccaaaagcggtgacccctaca**

**GauthierD**  **cgtctttgctaccactcgtgcgcactactttgattttgccggattccttaagctcgaaaccaaaagcggtgacccctaca**

**CDC2D**  **cgtctttgctaccactcgtgcgcactactttgattttgccggattccttaagctcgaaaccaaaagcggtgacccctaca**

**SamoaDC**  **cgtctttgctaccactcgtgcgcactactttgattttgccggattccttaagctcgaaaccaaaagcggtgacccctaca**

**Fribourg-BlancC** **cgtctttgctaccactcgtgcgcactactttgattttgccggattccttaagctcgaaaccaaaagcggtgacccctaca**

**IraqBC**  **cgtctttgctaccactcgtgcgcactactttgattttgccggattccttaagctcgaaactaagagcggtgacccctaca**

**BosniaAC**  **cgtctttgctaccactcgtgcgcactactttgattttgccggattccttaagctcgaaactaagagcggtgacccctaca**

1370 1380 1390 1400 1410 1420 1430 1440

....|....|....|....|....|....|....|....|....|....|....|....|....|....|....|....|

**NicholsC**  **cccacctgctcaccggcctgaacgccggcgtcgaagcacgcgtgtacatccccctcacctacatccgttacagaaataac**

**NicholsD**  **cccacctgctcaccggcctgaacgccggcgtcgaagcacgcgtgtacatccccctcacctacatccgttacagaaataac**

**Bal3C**  **cccacctgctcaccggcctgaacgccggcgtcgaagcacgcgtgtacatccccctcacctacgtcttttacagaaataac**

**Sea81-4C**  **cccacctgctcaccggcctgaacgccggcgtcgaagcacgcgtgtacatccccctcacctacgtcttttacagaaataac**

**Street14C cccacctgctcaccggcctgaacgccggcgtcgaagcacgcgtgtacatccccctcacctacgtcttttacagaaataac**

**MexicoAC**  **cccacctgctcaccggcctgaacgccggcgtcgaagcacgcgtgtacatccccctcacctacgtcttttacagaaataac**

**GauthierC**  **cccacctgctcaccggcctgaacgccggcgtcgaagcacgcgtgtacatccccctcacctacgtcttttacaaaaataac**

**CDC2C**  **cccacctgctcaccggcctgaacgccggcgtcgaagcacgcgtgtacatccccctcacctacgtcttttacaaaaataac**

**GauthierD**  **cccacctgctcaccggcctgaacgccggcgtcgaagcacgcgtgtacatccccctcacctacgtcttttacaaaaataac**

**CDC2D**  **cccacctgctcaccggcctgaacgccggcgtcgaagcacgcgtgtacatccccctcacctacgtcttttacaaaaataac**

**SamoaDC**  **cccacctgctcaccggcctgaacgccggcgtcgaagcacgcgtgtacatccccctcacctacgtcttttacaaaaataac**

**Fribourg-BlancC** **cccacctgctcaccggcctgaacgccggcgtcgaagcacgcgtgtacatccccctcacctacgtcttttacaaaaataac**

**IraqBC**  **cccacctgctcaccggcctgaacgccggcgtcgaagcacgcgtgtacatccccctcacctacgtcttttacaaaaataac**

**BosniaAC**  **cccacctgctcaccggcctgaacgccggcgtcgaagcacgcgtgtacatccccctcacctacgtcttttacaaaaataac**

1450 1460 1470 1480 1490 1500 1510 1520

....|....|....|....|....|....|....|....|....|....|....|....|....|....|....|....|

**NicholsC**  **ggagggtacgaactgaatggagctgtgccccctgggactatcaatatgccaattttggggaaggcgtggtgcagctatcg**

**NicholsD**  **ggagggtacgaactgaatggagctgtgccccctgggactatcaatatgccaattttggggaaggcgtggtgcagctatcg**

**Bal3C**  **ggagggtacgaactgaatagagttgtgccctctgggattatcaatatgccaattttggggaaggcgtggtgcagctatcg**

**Sea81-4C**  **ggagggtacgaactgaatagagttgtgccctctgggattatcaatatgccaattttggggaaggcgtggtgcagctatcg**

**Street14C ggagggtacgaactgaatggagctgtgccccctgggactatcaatatgccaattttggggaaggcgtggtgcagctatcg**

**MexicoAC**  **ggagggtacgaactgaatagagttgtgccctctgggattatcaatatgccaattttggggaaggcgtggtgcagctatcg**

**GauthierC**  **ggagggtacccactgaatggagttgtgccctctgggactatcaatatgccgattttggggaaggcgtggtgcagctatcg**

**CDC2C**  **ggagggcacccactgaatggagttgtgccctctgggactatcaatatgccgattttggggaaggcgtggtgcagctatcg**

**GauthierD**  **ggagggtacccactgaatggagttgtgccctctgggactatcaatatgccgattttggggaaggcgtggtgcagctatcg**

**CDC2D**  **ggagggcacccactgaatggagttgtgccctctgggactatcaatatgccgattttggggaaggcgtggtgcagctatcg**

**SamoaDC**  **ggagggtacccactgaatggagttgtgccctctgggactatcaatatgccgattttggggaaggcgtggtgcagctatcg**

**Fribourg-BlancC** **ggagggcacctactggatggagttgtgccccttgggactatcaatatgccgattttggggaaggcgtggtgcagctatcg**

**IraqBC**  **ggagggtacgaactgaatggagttgtgccccctgggattatcaatatgccgattttgggaaaggcgtggtgcagctatcg**

**BosniaAC**  **ggagggtacgaactgaatggagttgtgccccctgggattatcaatatgccgattttggggaaggcgtggtgcagctatcg**

1530 1540 1550 1560 1570 1580 1590 1600

....|....|....|....|....|....|....|....|....|....|....|....|....|....|....|....|

**NicholsC**  **catccccctcggttcccacgcctggcttgcaccacacacatccgtgctcggcacaaccaatcgctttaacattattaacc**

**NicholsD**  **catccccctcggttcccacgcctggcttgcaccacacacatccgtgctcggcacaaccaatcgctttaacattattaacc**

**Bal3C**  **catccccctcggttcccacgcctggcttgcaccacacacatccgtgctcggcacaaccaatcgctttaacattattaacg**

**Sea81-4C**  **catccccctcggttcccacgcctggcttgcaccacacacatccgtgctcggcacaaccaatcgctttaacattattaacg**

**Street14C catccccctcggttcccacgcctggcttacaccgcatacatccgtgctcggcacaaccaatcgctttaacgttattaacc**

**MexicoAC**  **catccccctcggttcccacgcctggcttgcaccacacacatccgtgctcggcacaaccaatcgctttaacattattaacg**

**GauthierC**  **catccccctcggttcccacgcctggcttgcaccgcacacatccgtgctcggcacaaccaatcgctttaacattattaacg**

**CDC2C**  **catccccctcggttcccacgcctggcttgcaccgcacacatccgtgctcggcacaaccaatcgctttaacattattaacg**

**GauthierD**  **catccccctcggttcccacgcctggcttgcaccgcacacatccgtgctcggcacaaccaatcgctttaacattattaacg**

**CDC2D**  **catccccctcggttcccacgcctggcttgcaccgcacacatccgtgctcggcacaaccaatcgctttaacattattaacg**

**SamoaDC**  **catccccctcggttcccacgcctggcttgcaccgcacacatccgtgctcggcacaaccaatcgctttaacattattaacg**

**Fribourg-BlancC** **catccccctcggttcccacgcctggcttgcaccgcacacatccgtgctcggcacaaccaatcgctttaacattattaacg**

**IraqBC**  **catccccctcggttcccacgcctggcttgcaccgcacacatccgtgctcggcacaaccaatcgctttaacattattaacg**

**BosniaAC**  **catccccctcggttcccacgcctggcttgcaccgcacacatccgtgctcggcacaaccaatcgctttaacattattaacg**

1610 1620 1630 1640 1650 1660 1670 1680

....|....|....|....|....|....|....|....|....|....|....|....|....|....|....|....|

**NicholsC**  **ccgcgggcaacctgttgaatgaacgagcgctccagtaccaggtgggactgacgttcagtcccttcgagaaggtggagctc**

**NicholsD**  **ccgcgggcaacctgttgaatgaacgagcgctccagtaccaggtgggactgacgttcagtcccttcgagaaggtggagctc**

**Bal3C**  **ccgcgggcaacctgttgaatgaacgagcgctccagtaccaggtgggactgacgttcagtcccttcgagaaggtggagctc**

**Sea81-4C**  **ccgcgggcaacctgttgaatgaacgagcgctccagtaccaggtgggactgacgttcagtcccttcgagaaggtggagctc**

**Street14C ccgcgggcaacctgttgaatgaacgagcgctccagtaccaggtgggactgacgttcagtcccttcgagaaggtggagctc**

**MexicoAC**  **ccgcgggcaacctgttgaatgaacgagcgctccagtaccaggtgggactgacgttcagtcccttcgagaaggtggagctc**

**GauthierC**  **ccgcgggcaacctggtgaatgaacgagcgctccagtaccaggtgggactgacgttcagtcccttcgagaaggtggagctc**

**CDC2C**  **ccgcgggcaacctggtgaatgaacgagcgctccagtaccaggtgggactgacgttcagtcccttcgagaaggtggagctc**

**GauthierD**  **ccgcgggcaacctggtgaatgaacgagcgctccagtaccaggtgggactgacgttcagtcccttcgagaaggtggagctc**

**CDC2D**  **ccgcgggcaacctggtgaatgaacgagcgctccagtaccaggtgggactgacgttcagtcccttcgagaaggtggagctc**

**SamoaDC**  **ccgcgggcaacctggtgaatgaacgagcgctccagtaccaggtgggactgacgttcagtcccttcgagaaggtggagctc**

**Fribourg-BlancC** **ccgcgggcaacctggtgaatgaacgagcgctccagtaccaggtgggactgacgttcagtcccttcgagaaggtggagctc**

**IraqBC**  **ccgcgggcaacctggtgaatgaacgagcgctccagtaccaggtgggactgacgttcagtcccttcgagaaggtggagctc**

**BosniaAC**  **ccgcgggcaacctggtgaatgaacgagcgctccagtaccaggtgggactgacgttcagtcccttcgagaaggtggagctc**

1690 1700 1710 1720 1730 1740 1750 1760

....|....|....|....|....|....|....|....|....|....|....|....|....|....|....|....|

**NicholsC**  **agcgcccagtgggaacagggcgtgcttgctgacgctccttacatgggcattgccgagagcatctggtccgaacgccactt**

**NicholsD**  **agcgcccagtgggaacagggcgtgcttgctgacgctccttacatgggcattgccgagagcatctggtccgaacgccactt**

**Bal3C**  **agcgcccagtgggaacagggggtgctctccgatgttccctacatgggcattgccgagagcatctggtccgaacgccactt**

**Sea81-4C**  **agcgcccagtgggaacagggggtgctctccgatgttccctacatgggcattgccgagagcatctggtccgaacgccactt**

**Street14C agcgcccagtgggaacagggcgtgcttgctgacgctccttacatgggtattactcagagcatcgggtccgaccgccactt**

**MexicoAC**  **agcgcccagtgggaacagggcgtgcttgctgacgctccttacatgggtattactcagagcatcgggtccgaccgccactt**

**GauthierC**  **agcgcccagtgggaacagggggtgctctccgatgttccctacatgggtattactcagagcatctggtccgaacgccactt**

**CDC2C**  **agcgcccagtgggaacagggggtgctctccgatgttccctacatgggtattactcagagcatctggtccgaacgccactt**

**GauthierD**  **agcgcccagtgggaacagggggtgctctccgatgttccctacatgggtattactcagagcatctggtccgaacgccactt**

**CDC2D**  **agcgcccagtgggaacagggggtgctctccgatgttccctacatgggtattactcagagcatctggtccgaacgccactt**

**SamoaDC**  **agcgcccagtgggaacagggggtgctctccgatgttccctacatgggtattactcagagcatctggtccgaacgccactt**

**Fribourg-BlancC** **agcgcccagtgggaacagggggtgctctccgatgttccctacatgggcattgccgagagcatctggtccgaacgccactt**

**IraqBC**  **agcgcccagtgggaacagggggtgctctccgatgttccctacatgggcattgccgagagcatctggtccgaacgccactt**

**BosniaAC**  **agcgcccagtgggaacagggggtgctctccgatgttccctacatgggcattgccgagagcatctggtccgaacgccactt**

1770 1780 1790

....|....|....|....|....|....|....|..

**NicholsC**  **cggcacccttgtctgcggaatgaaagtgacatggtaa**

**NicholsD**  **cggcacccttgtctgcggaatgaaagtgacatggtaa**

**Bal3C**  **cggcacccttgtctgcggaatgaaagtgacatggtaa**

**Sea81-4C**  **cggcacccttgtctgcggaatgaaagtgacatggtaa**

**Street14C cggcacccttgtctgtggaatgaaagtgacatggtaa**

**MexicoAC**  **cggcacccttgtctgtggaatgaaagtgacatggtaa**

**GauthierC**  **cggcacctttgtctgcggaatgaaagtgacatggtaa**

**CDC2C**  **cggcacctttgtctgcggaatgaaagtgacatggtaa**

**GauthierD**  **cggcacctttgtctgcggaatgaaagtgacatggtaa**

**CDC2D**  **cggcacccttgtctgtggaatgaaagtgacatggtaa**

**SamoaDC**  **cggcacctttgtctgcggaatgaaagtgacatggtaa**

**Fribourg-BlancC** **cggcacctttgtctgcggaatgaaagtgacatggtaa**

**IraqBC**  **cggcacccttgtctgcggaatgaaagtgacatggtaa**

**BosniaAC**  **cggcacccttgtctgcggaatgaaagtgacatggtaa**

***1.4 tprE* Locus**

**10 20 30 40 50 60 70 80**

**....|....|....|....|....|....|....|....|....|....|....|....|....|....|....|....|**

**NicholsE**  **MGCMRWGSVLCVVVGVGASGGVLGQEFSPKLTGSATLEWGISYGKGVGSHGQAPGAVMGTGPYNLKHGFRTTNTVGVSFP**

**Bal3E**  **MGCMRWGSVLCVVVGVGASGGVLGQEFSPKLTGSATLEWGISYGKGVGSHGQAPGAVMGTGPYNLKHGFRTTNTVGVSFP**

**Sea81-4E**  **MGCMRWGSVLCVVVGVGASGGVLGQEFSPKLTGSATLEWGISYGKGVGSHGQAPGAVMGTGPYNLKHGFRTTNTVGVSFP**

**MexicoAE**  **MGCMRWGSVLCVVVGVGASGGVLGQEFSPKLTGSATLEWGISYGKGVGSHGQAPGAVMGTGPYNLKHGFRTTNTVGVSFP**

**Street14E**  **MGCMRWGSVLCVVVGVGASGGVLGQEFSPKLTGSATLEWGISYGKGVGSHGQAPGAVMGTGPYNLKHGFRTTNTVGVSFP**

**GauthierE**  **MGCMRWGSVLCVVVGVGASGGVLGQEFSPKLTGSATLEWGISYGKGVGSHGQAPGAVMGTGPYNLKHGFRTTNTVGVSFP**

**SamoaDE**  **MGCMRWGSVLCVVVGVGASGGVLGQEFSPKLTGSATLEWGISYGKGVGSHGQAPGAVMGTGPYNLKHGFRTTNTVGVSFP**

**CDC2E**  **MGCMRWGSVLCVVVGVGASGGVLGQEFSPKLTGSATLEWGISYGKGVGSHGQAPGAVMGTGPYNLKHGFRTTNTVGVSFP**

**BosniaAE**  **MGCMRWGSVLCVVVGVGASGGVLGQEFSPKLTGSATLEWGISYGKGVGSHGQAPGAVMGTGPYNLKHGFRTTNTVGVSFP**

**IraqBE**  **MGCMRWGSVLCVVVGVGASGGVLGQEFSPKLTGSATLEWGISYGKGVGSHGQAPGAVMGTGPYNLKHGFRTTNTVGVSFP**

**Fribourg-BlancE** **MGCMRWGSVLCVVVGVGTSGGVLGQEFSPKLTGSATLEWGISYGKGVGSHGQAPGAVMGTGPYNLKHGFRTTNTVGVSFP**

**CuniculiAE**  **MGCMRWGSVLCVVVGVGASGGVLGQEFSPKLTGSATLEWGISYGKGVGSHDQAPGAVMGTGPYNLKHGFRTTNTVGVSFP**

**90 100 110 120 130 140 150 160**

**....|....|....|....|....|....|....|....|....|....|....|....|....|....|....|....|**

**NicholsE**  **LVMRTTHTRRGQHPALYAELKVADLQADLSQGKAGFAVKRKGKVEATLHCYGAYLTIGKNPTFLTNFARLWKPWVTAQYQ**

**Bal3E**  **LVMRTTHTRRGQHPALYAELKVADLQADLSQGKAGFAVKRKGKVEATLHCYGAYLTIGKNPTFLTNFARLWKPWVTAQYQ**

**Sea81-4E**  **LVMRTTHTRRGQHPALYAELKVADLQADLSQGKAGFAVKRKGKVEATLHCYGAYLTIGKNPTFLTNFARLWKPWVTAQYQ**

**MexicoAE**  **LVMRTTHTRRGQHPALYAELKVADLQADLSQGKAGFAVKRKGKVEATLHCYGAYLTIGKNPTFLTNFARLWKPWVTAQYQ**

**Street14E LVMRTTHTRRGQHPALYAELKVADLQADLSQGKAGFAVKRKGKVEATLHCYGAYLTIGKNPTFLTNFARLWKPWVTAQYQ**

**GauthierE**  **LVMRTTHTRRGQHPALYAELKVADLQADLSQGKAGFAVKRKGKVEATLHCYGAYLTIGKNPTFLTNFARLWKPWVTAQYQ**

**SamoaDE**  **LVMRTTHTRRGQHPALYAELKVADLQADLSQGKVGFAVKRKGKVEATLHCYGAYLTIGKNPTFLTNFARLWKPWVTAQYQ**

**CDC2E**  **LVMRTTHTRRGQHPALYAELKVADLQADLSQGKAGFAVKRKGKVEATLHCYGAYLTIGKNPTFLTNFARLWKPWVTAQYQ**

**BosniaAE**  **LVMRTTHTRRGQHPALYAELKVADLQADLSQGKVGFAVKRKGKVEATLHCYGAYLTIGKNPTFLTNFARLWKPWVTAQYQ**

**IraqBE**  **LVMRTTHTRRGQHPALYAELKVADLQADLSQGKVGFAVKRKGKVEATLHCYGAYLTIGKNPTFLTNFARLWKPWVTAQYQ**

**Fribourg-BlancE** **LVMRTTHTRRGQHPALYAELKVADLQADLSQGKAGFAVKRKGKVEATLHCYGAYLTIGKNPTFLTNFARLWKPWVTAQYQ**

**CuniculiAE**  **LVMRTTHTRRGQHPALYAELKVADLQADLSQGKAGFDFKRKGKVEATLHCYGAYLTIGKNPTFLTNFARLWKPWVTAQYQ**

**170 180 190 200 210 220 230 240**

**....|....|....|....|....|....|....|....|....|....|....|....|....|....|....|....|**

**NicholsE**  **EDAVQYAPGFGGLGGKVGYRAQDIGGSGVSLDVGFLSFASNGAWDSTDPTHSKYGFGADLKLMYARAGHPLCTVELASNV**

**Bal3E**  **EDAVQYAPGFGGLGGKVGYRAQDIGGSGVSLDVGFLSFASNGAWDSTDPTHSKYGFGADLKLMYARAGHPLCTVELASNV**

**Sea81-4E**  **EDAVQYAPGFGGLGGKVGYRAQDIGGSGVSLDVGFLSFASNGAWDSTDPTHSKYGFGADLKLMYARAGHPLCTVELASNV**

**MexicoAE**  **EDAVQYAPGFGGLGGKVGYRAQDIGGSGVSLDVGFLSFASNGAWDSTDPTHSKYGFGADLKLMYARAGHPLCTVELASNV**

**Street14E EDAVQYAPGFGGLGGKVGYRAQDIGGSGVSLDVGFLSFASNGAWDSTDPTHSKYGFGADLKLMYARAGHPLCTVELASNV**

**GauthierE**  **EDAVQYAPGFGGLGGKVGYRAQDIGGSGVSLDVGFLSFASNGAWDSTDPTHSKYGFGADLKLMYARAGHPLCTVELASNV**

**SamoaDE**  **EDAVQYAPGFGGLGGKVGYRAQDIGGSGVSLDVGFLSFASNGAWDSTDPTHSKYGFGADLKLMYARAGHPLCTVELASNV**

**CDC2E**  **EDAVQYAPGFGGLGGKVGYRAQDIGGSGVSLDVGFLSFASNGAWDSTDPTHSKYGFGADLKLMYARAGHPLCTVELASNV**

**BosniaAE**  **EDAVQYAPGFGGLGGKVGYRAQDIGGSGVSLDVGFLSFASNGAWDSTDPTHSKYGFGADLKLMYARAGHPLCTVELASNV**

**IraqBE**  **EDAVQYAPGFGGLGGKVGYRAQDIGGSGVSLDVGFLSFATNGAWDSTDPTHSKYGFGADLKLMYARAGHPLCTVELASNV**

**Fribourg-BlancE** **EDAVQYAPGFGGLGGKVGYRAQDIGGSGVSLDVGFLSFATNGAWDSTDPTHSKYGFGADLKLMYARAGHPLCTVELASNV**

**CuniculiAE**  **EDEVQYAPGFGGLGGKVGYRAQDIGGSGVSLDVGFLSFASNGAWDSTDTTHSKYGFGGRLEANVCACRTPSVHGRACQQC**

**250 260 270 280 290 300 310 320**

**....|....|....|....|....|....|....|....|....|....|....|....|....|....|....|....|**

**NicholsE**  **TLEDGYLIGAQKDANNQNKDKLLWNVGGRLTLEPGAGFRFSFALDAGNQHQSEATAAMRTERTRERAQEVALAIFTHAAQ**

**Bal3E**  **TLEDGYLIGAQKDANNQNKDKLLWNVGGRLTLEPGAGFRFSFALDAGNQHQSEATAAMRTERTRERAQEVALAIFTHAAQ**

**Sea81-4E**  **TLEDGYLIGAQKDANNQNKDKLLWNVGGRLTLEPGAGFRFSFALDAGNQHQSEATAAMRTERTRERAQEVALAIFTHAAQ**

**MexicoAE**  **TLEDGYLIGAQKDANNQNKDKLLWNVGGRLTLEPGAGFRFSFALDAGNQHQSEATAAMRTERTRERAQEVALAIFTHAAQ**

**Street14E TLEDGYLIGAQKDANNQNKDKLLWNVGGRLTLEPGAGFRFSFALDAGNQHQSEATAAMRTERTRERAQEVALAIFTHAAQ**

**GauthierE**  **TLEDGYLIGAQKDANNQNKDKLLWNVGGRLTLEPGAGFRFSFALDAGNQHQSEATAAMRTERTRERAQEVALAIFTHAAQ**

**SamoaDE**  **TLEDGYLIGAQKDANNQNKDKLLWNVGGRLTLEPGAGFRFSFALDAGNQHQSEATAAMRTERTRERAQEVALAIFTHAAQ**

**CDC2E**  **TLEDGYLIGAQKDANNQNKDKLLWNVGGRLTLEPGAGFRFSFALDAGNQHQSEATAAMRTERTRERAQEVALAIFTHAAQ**

**BosniaAE**  **TLEDGYLIGAQKDANNQNKDKLLWNVGGRLTLEPGAGFRFSFALDAGNQHQSEATAAMRTERTRERAQEVALAIFTHAAQ**

**IraqBE**  **TLEDGYLIGAQKDANNQNKDKLLWNVGGRLTLEPGAGFRFSFALDAGNQHQSEATAAMRTERTRERAQEVALAIFTHAAQ**

**Fribourg-BlancE** **TLEDGYLIGAQKDANNQNKDKLLWNVGGRLTLEPGAGFRFSFALDAGNQHQSEATAAMRTERTRERAQEVALAIFTHAAQ**

**CuniculiAE**  **YAGRRIPHRCAEGRKQSEQG**

**330 340 350 360 370 380 390 400**

**....|....|....|....|....|....|....|....|....|....|....|....|....|....|....|....|**

**NicholsE**  **EQAKQAADTVGSTIDNSVQVARSVITQIAEGAVKQAHDQIKRTNGTQVVNIDVTVPVNVRQSPVRQPDLPSLTAIAAQLP**

**Bal3E**  **EQAKQAADTVGSTIDNSVQVARSVITQIAEGAVKQAHDQIKRTNGTQVVNIDVTVPVNVRQSPVRQPDLPSLTAIAAQLP**

**Sea81-4E**  **EQAKQAADTVGSTIDNSVQVARSVITQIAEGAVKQAHDQIKRTNGTQVVNIDVTVPVNVRQSPVRQPDLPSLTAIAAQLP**

**MexicoAE**  **EQAKQAADTVGSTIDNSVQVARSVITQIAEGAVKQAHDQIKRTNGTQVVNIDVTVPVNVRQSPVRQPDLPSLTAIAAQLP**

**Street14E EQAKQAADTVGSTIDNSVQVARSVITQIAEGAVKQAHDQIKRTNGTQVVNIDVTVPVNVRQSPVRQPDLPSLTAIAAQLP**

**GauthierE**  **EQAKQAADTVGSTIDNSVQVARSVITQIAEGAVKQAHDQIKRTNGTQVVNIDVTVPVNVRQSPVRQPDLPSLTAIAAQLP**

**SamoaDE**  **EQAKQAADTVGSTIDNSVQVARSVITQIAEGAVKQAHDQIKRTNGTQVVNIDVTVPVNVRQSPVRQPDLPSLTAIAAQLP**

**CDC2E**  **EQAKQAADTVGSTIDNSVQVARSVITQIAEGAVKQAHDQIKRTNGTQVVNIDVTVPVNVRQSPVRQPDLPSLTAIAAQLP**

**BosniaAE**  **EQAKQAADTVGSTIDNSVQVARSVITQIAEGAVKQAHDQIKRTNGTQVVNIDVTVPVNVRQSPVRQPDLPSLTAIAAQLP**

**IraqBE**  **EQAKQAADTVGSTIDNSVQVARSVITQVAEGAVKQAHDQIKRTNGTQVVNIDVTVPVNVRQSPVRQPDLPSLTAIAAQLP**

**Fribourg-BlancE** **EQAKQAADTVGSTIDNSVQVARSVITQIAEGAVKQAHDQIKRTNGTQVVNIDVTVPVNVRQSPVRQPDLPSLTAIAAQLP**

**CuniculiAE**

**410 420 430 440 450 460 470 480**

**....|....|....|....|....|....|....|....|....|....|....|....|....|....|....|....|**

**NicholsE**  **NVTKLFFLSAGAAAARPIIGQITGVVQNVITQQVQARVAQSTAVAIQQVLVFNQQTVAAEKANTQKHTINGKSYAAHIGS**

**Bal3E**  **NVTKLFFLSAGAAAARPIIGQITGVVQNVITQQVQARVAQSTAVAIQQVLVFNQQTVAAEKANTQKHTINGKSYAAHIGS**

**Sea81-4E**  **NVTKLFFLSAGAAAARPIIGQITGVVQNVITQQVQARVAQSTAVAIQQVLVFNQQTVAAEKANTQKHTINGKSYAAHIGS**

**MexicoAE**  **NVTKLFFLSAGAAAARPIIGQITGVVQNVITQQVQARVAQSTAVAIQQVLVFNQQTVAAEKANTQKHTINGKSYAAHIGS**

**Street14E NVTKLFFLSAGAAAARPIIGQITGVVQNVITQQVQARVAQSTAVAIQQVLVFNQQTVAAEKANTQKHTINGKSYAAHIGS**

**GauthierE**  **NVTKLFFLSAGAAAARPIIGQITGVVQNVITQQVQARVAQSTAVAIQQVSVFNQQTVAAEKANTQKHTINGKSYAAHIGS**

**SamoaDE**  **NVTKLFFLSAGAAAARPIIGQITGVVQNVITQQVQARVAQSTAVAIQQVSVFNQQTVAAEKANTQKHTINGKSYAAHIGS**

**CDC2E**  **NVTKLFFLSAGAAAARPIIGQITGVVQNVITQQVQARVAQSTAVAIQQVSVFNQQTVAAEKANTQKHTINGKSYAAHIGS**

**BosniaAE**  **NVTKLFFLSAGAAAARPIIGQITGVVQNVITQQVQARVAQSTAVAIQQVSVFNQQTVAAEKANTQKHTINGKSYAAHIGS**

**IraqBE**  **NVTKLFFLSAGAAAARPIIGQITGVVQNVITQQVQARVAQSTAVAIQQVSVFNQQTVAAEKANTQKHTINGKSYAAHIGS**

**Fribourg-BlancE** **NVTKLFFLSAGAAAARPIIGQITGVVQNVITQQVQARVAQSTAVAIQQVSVFNQQTVAAEKANTQKHTINGKSYAAHIGS**

**CuniculiAE**

**490 500 510 520 530 540 550 560**

**....|....|....|....|....|....|....|....|....|....|....|....|....|....|....|....|**

**NicholsE**  **LVSLATNRALPTIRQRVEQAVQENIRRINAVVQQKAQTLTSSQELEKAVYSLFVPTFENLVLGAGALLALLDMHQIAVDA**

**Bal3E**  **LVSLATNRALPTIRQRVEQAVQENIRRINAVVQQKAQTLTSSQELEKAVYSLFVPTFENLVLGAGALLALLDMHQIAVDA**

**Sea81-4E**  **LVSLATNRALPTIRQRVEQAVQENIRRINAVVQQKAQTLTSSQELEKAVYSLFVPTFENLVLGAGALLALLDMHQIAVDA**

**MexicoAE**  **LVSLATNRALPTIRQRVEQAVQENIRRINAVVQQKAQTLTSSQELEKAVYSLFVPTFENLVLGAGALLALLDMHQIAVDA**

**Street14E LVSLATNRALPTIRQRVEQAVQENIRRINAVVQQKAQTLTSSQELEKAVYSLFVPTFENLVLGAGALLALLDMHQIAVDA**

**GauthierE**  **LVSLATNRALPTIQQRVKQAVQENIRRINAVVQQKAQTLTSSQELEKAVYSLFVPTFENLVLGAGALLALLDMHQIAVDA**

**SamoaDE**  **LVSLATNRALPTIQQRVKQAVQENIRRINAVVQQKAQTLTSSQELEKAVYSLFVPTFENLVLGAGALLALLDMHQIAVDA**

**CDC2E**  **LVSLATNRALPTIQQRVKQAVQENIRRINAVVQQKAQTLTSSQELEKAVYSLFVPTFENLVLGAGALLALLDMHQIAVDA**

**BosniaAE**  **LVSLATNRALPTIQQRVKQAVQENIRRINAVVQQKAQTLTSSQELEKAVYSLFVPTFENLVLGAGALLALLDMHQIAVDA**

**IraqBE**  **LVSLATNRALPTIQQRVKQAVQENIRRINAVVQQKAQTLTSSQELEKAVYSLFVPTFENLVLGAGALLALLDMHQIAVDA**

**Fribourg-BlancE** **LVSLATNRALPTIQQRVKQAVQENIRRINAVVQQKAQTLTSSQELEKAVYSLFVPTFENLVLGAGALLALLDMHQIAVDA**

**CuniculiAE**

**570 580 590 600 610 620 630 640**

**....|....|....|....|....|....|....|....|....|....|....|....|....|....|....|....|**

**NicholsE**  **LFTAQWKWLSSGIYFATAPANVFGTRVLDNTIASCGDFAGFLKLETKSGDPYTHLLTGLDAGVETRVYIPLTYALYKNNG**

**Bal3E**  **LFTAQWKWLSSGIYFATAPANVFGTRVLDNTIASCGDFAGFLKLETKSGDPYTHLLTGLDAGVETRVYIPLTYALYKNNG**

**Sea81-4E**  **LFTAQWKWLSSGIYFATAPANVFGTRVLDNTIASCGDFAGFLKLETKSGDPYTHLLTGLDAGVETRVYIPLTYALYKNNG**

**MexicoAE**  **LFTAQWKWLSSGIYVATAPANVFGTRVLDNTIASCGDFAGFLKLETKSGDPYTHLLTGLDAGVETRVYIPLTYALYKNNG**

**Street14E LFTAQWKWLSSGIYFATAPANVFGTRVLDNTIASCGDFAGFLKLETKSGDPYTHLLTGLDAGVETRVYIPLTYALYKNNG**

**GauthierE**  **LFTAQWKWLSSGIYFATAPANVFGTRVLDNTIASCGDFAGFLKLETKSGDPYTHLLTGLDAGVETRMYIPLTYALYKNNG**

**SamoaDE**  **LFTAQWKWLSSGIYFATAPANVFGTRVLDNTIASCGDFAGFLKLETKSGDPYTHLLTGLDAGVETRMYIPLTYALYKNNG**

**CDC2E**  **LFTAQWKWLSSGIYFATAPANVFGTRVLDNTIASCGDFAGFLKLETKSGDPYTHLLTGLDAGVETRMYIPLTYALYKNNG**

**BosniaAE**  **LFTAQWKWLSSGIYFATAPANVFGTRVLDNTIASCGDFAGFLKLETKSGDPYTHLLTGLDAGVETRMYIPLTYALYKNNG**

**IraqBE**  **LFTAQWKWLSSGIYFATAPANVFGTRVLDNTIASCGDFAGFLKLETKSGDPYTHLLTGLDAGVETRMYIPLTYALYKNNG**

**Fribourg-BlancE** **LFTAQWKWLSSGIYFATAPANVFGTRVLDNTIASCGDFAGFLKLETKSGDPYTHLLTGLDAGVETRMYIPLTYALYKNNG**

**CuniculiAE**

**650 660 670 680 690 700 710 720**

**....|....|....|....|....|....|....|....|....|....|....|....|....|....|....|....|**

**NicholsE**  **GTAVRGIQEKEYIRPPVVGKAWCSYRIPVQDYGWVKPSVTVHASTNRAHLNAPAAGGAVGATYLTKEYCAQLRAGISASL**

**Bal3E**  **GTAVRGIQEKEYIRPPVVGKAWCSYRIPVQDYGWVKPSVTVHASTNRAHLNAPAAGGAVGATYLTKEYCAQLRAGISASL**

**Sea81-4E**  **GTAVRGIQEKEYIRPPVVGKAWCSYRIPVQDYGWVKPSVTVHASTNRAHLNAPAAGGAVGATYLTKEYCAQLRAGISASL**

**MexicoAE**  **GTAVRGIQEKEYIRPPVVGKAWCSYRIPVQDYGWVKPSVTVHASTNRAHLNAPAAGGAVGATYLTKEYCAQLRAGISASL**

**Street14E GTAVRGIQEKEYIRPPVVGKAWCSYRIPVQDYGWVKPSVTVHASTNRAHLNAPAAGGAVGATYLTKEYCAQLRAGISASL**

**GauthierE**  **GTAVRGIQEKEYIRPPVVGKAWCSYRIPVQDYGWVKPSVTVHASTNRAHLNAPAAGGAVGATYLTKEYCAQLRAGISASL**

**SamoaDE**  **GTAVRGIQEKEYIRPPVVGKAWCSYRIPVQDYGWVKPSVTVHASTNRAHLNAPAAGGAVGATYLTKEYCAQLRAGISASL**

**CDC2E**  **GTAVRGIQEKEYIRPPVVGKAWCSYRIPVQDYGWVKPSVTVHASTNRAHLNAPAAGGAVGATYLTKEYCAQLRAGISASL**

**BosniaAE**  **GTAVRGIQEKEYIRPPVVGKAWCSYRIPVQDYGWVKPSVTVHASTNRAHLNAPAAGGAVGATYLTKEYCAQLRAGISASL**

**IraqBE**  **GTAVRGIQEKEYIRPPVVGKAWCSYRIPVQDYGWVKPSVTVHASTNRAHLNAPAAGGAVGATYLTKEYCAQLRAGISASL**

**Fribourg-BlancE** **GTAVRGIQEKEYIRPPVVGKAWCSYRIPVQDYGWVKPSVTVHASTNRAHLNAPAAGGAVGATYLTKEYCAQLRAGISASL**

**CuniculiAE**

**730 740 750 760**

**....|....|....|....|....|....|....|....|..**

**NicholsE**  **IEKTVFSLDWEQGMLSDVPYLLVSECLTQGIGRIVCGVTLSW**

**Bal3E**  **IEKTVFSLDWEQGMLSDVPYLLVSECLTQGIGRIVCGVTLSW**

**Sea81-4E**  **IEKTVFSLDWEQGMLSDVPYLLVSECLTQGIGRIVCGVTLSW**

**MexicoAE**  **IEKTVFSLDWEQGMLSDVPYLLVSECLTQGIGRIVCGVTLSW**

**Street14E IEKTVFSLDWEQGMLSDVPYLLVSECLTQGIGRIVCGVTLSW**

**GauthierE**  **IEKTVFSLDWEQGMLSDVPYLLVSECLTQGIGRIVCGVTLSW**

**SamoaDE**  **IEKTVFSLDWEQGMLSDVPYLLVSECLTQGIGRIVCGVTLSW**

**CDC2E**  **IEKTVFSLDWEQGMLSDVPYLLVSECLTQGIGRIVCGVTLSW**

**BosniaAE**  **IEKTVFSLDWEQGMLSDVPYLLVSECLTQGIGRIVCGVTLSW**

**IraqBE**  **IEKTVFSLDWEQGMLSDVPYLLVSECLTQGIGRIVCGVTLSW**

**Fribourg-BlancE** **IEKTVFSLDWEQGMLSDVPYLLVSECLTQGIGRIVCGVTLSW**

**CuniculiAE**

**....|....|....|....|....|....|....|....|....|....|....|....|....|....|....|....|**

**NicholsE**  **atggggtgcatgcggtgggggagtgtgctgtgtgtggtggtgggggtaggagcgagcgggggagtgctcggacaggagtt**

**Bal3E**  **atggggtgcatgcggtgggggagtgtgctgtgtgtggtggtgggggtaggagcgagcgggggagtgctcggacaggagtt**

**Sea81-4E**  **atggggtgcatgcggtgggggagtgtgctgtgtgtggtggtgggggtaggagcgagcgggggagtgctcggacaggagtt**

**MexicoAE**  **atggggtgcatgcggtgggggagtgtgctgtgtgtggtggtgggggtaggagcgagcgggggagtgctcggacaggagtt**

**Street14E**  **atggggtgcatgcggtgggggagtgtgctgtgtgtggtggtgggggtaggagcgagcgggggagtgctcggacaggagtt**

**GauthierE**  **atggggtgcatgcggtgggggagtgtgctgtgtgtggtggtgggggtaggagcgagcgggggagtgctcggacaggagtt**

**SamoaDE**  **atggggtgcatgcggtgggggagtgtgctgtgtgtggtggtgggggtaggagcgagcgggggagtgctcggacaggagtt**

**CDC2E**  **atggggtgcatgcggtgggggagtgtgctgtgtgtggtggtgggggtaggagcgagcgggggagtgctcggacaggagtt**

**BosniaAE**  **atggggtgcatgcggtgggggagtgtgctgtgtgtggtggtgggggtaggagcgagcgggggagtgctcggacaggagtt**

**IraqBE**  **atggggtgcatgcggtgggggagtgtgctgtgtgtggtggtgggggtaggagcgagcgggggagtgctcggacaggagtt**

**Fribourg-BlanCE** **atggggtgcatgcggtgggggagtgtgctgtgtgtggtggtgggggtaggaacgagcgggggagtgctcggacaggagtt**

**CuniculiAE**  **atggggtgcatgcggtgggggagtgtgctgtgtgtggtggtgggggtaggagcgagcgggggagtgcttggacaggagtt**

**90 100 110 120 130 140 150 160**

**....|....|....|....|....|....|....|....|....|....|....|....|....|....|....|....|**

**NicholsE**  **ttccccgaagctaactggctctgccacacttgagtggggcatcagctatggcaagggggtaggcagtcatggccaggccc**

**Bal3E**  **ttccccgaagctaactggctctgccacacttgagtggggcatcagctatggcaagggggtaggcagtcatggccaggccc**

**Sea81-4E**  **ttccccgaagctaactggctctgccacacttgagtggggcatcagctatggcaagggggtaggcagtcatggccaggccc**

**MexicoAE**  **ttccccgaagctaactggctctgccacacttgagtggggcatcagctatggcaagggggtaggcagtcatggccaggccc**

**Street14E**  **ttccccgaagctaactggctctgccacacttgagtggggcatcagctatggcaagggggtaggcagtcatggccaggccc**

**GauthierE**  **ttccccgaagctaactggctctgccacacttgagtggggcatcagctatggcaagggggtaggcagtcatggccaggccc**

**SamoaDE**  **ttccccgaagctaactggctctgccacacttgagtggggcatcagctatggcaagggggtaggcagtcatggccaggccc**

**CDC2E**  **ttccccgaagctaactggctctgccacacttgagtggggcatcagctatggcaagggggtaggcagtcatggccaggccc**

**BosniaAE**  **ttccccgaagctaactggctctgccacacttgagtggggcatcagctatggcaagggggtaggcagtcatggccaggccc**

**IraqBE**  **ttccccgaagctaactggctctgccacacttgagtggggcatcagctatggcaagggggtaggcagtcatggccaggccc**

**Fribourg-BlanCE** **ttccccgaagctaactggctctgccacacttgagtggggcatcagctatggcaagggggtaggcagtcatggccaggccc**

**CuniculiAE**  **ttccccgaagctaactggctccgccacacttgagtggggcatcagctatggcaagggggtaggcagtcacgaccaggccc**

**170 180 190 200 210 220 230 240**

**....|....|....|....|....|....|....|....|....|....|....|....|....|....|....|....|**

**NicholsE**  **ctggtgcagttatgggcaccggtccctacaatctgaagcacgggtttcgtactaccaacacggtgggagtatcctttccc**

**Bal3E**  **ctggtgcagttatgggcaccggtccctacaatctgaagcacgggtttcgtactaccaacacggtgggagtatcctttccc**

**Sea81-4E**  **ctggtgcagttatgggcaccggtccctacaatctgaagcacgggtttcgtactaccaacacggtgggagtatcctttccc**

**MexicoAE**  **ctggtgcagttatgggcaccggtccctacaatctgaagcacgggtttcgtactaccaacacggtgggagtatcctttccc**

**Street14E**  **ctggtgcagttatgggcaccggtccctacaatctgaagcacgggtttcgtactaccaacacggtgggagtatcctttccc**

**GauthierE**  **ctggtgcagttatgggcaccggtccctacaatctgaagcacgggtttcgtactaccaacacggtgggagtatcctttccc**

**SamoaDE**  **ctggtgcagttatgggcaccggtccctacaatctgaagcacgggtttcgtactaccaacacggtgggagtatcctttccc**

**CDC2E**  **ctggtgcagttatgggcaccggtccctacaatctgaagcacgggtttcgtactaccaacacggtgggagtatcctttccc**

**BosniaAE**  **ctggtgcagttatgggcaccggtccctacaatctgaagcacgggtttcgtactaccaacacggtgggagtatcctttccc**

**IraqBE**  **ctggtgcagttatgggcaccggtccctacaatctgaagcacgggtttcgtactaccaacacggtgggagtatcctttccc**

**Fribourg-BlanCE** **ctggtgcagttatgggcaccggtccctacaatctgaagcacgggtttcgtactaccaacacggtgggagtatcctttccc**

**CuniculiAE**  **ctggtgcagttatgggcaccggtccctacaatctgaagcatgggtttcgtactaccaacacggtgggagtatcctttccc**

**250 260 270 280 290 300 310 320**

**....|....|....|....|....|....|....|....|....|....|....|....|....|....|....|....|**

**NicholsE**  **ctggttatgcgcaccacccacacgcgccgtgggcagcacccggcactgtatgcggagctgaaggtggcggacctgcaggc**

**Bal3E**  **ctggttatgcgcaccacccacacgcgccgtgggcagcacccggcactgtatgcggagctgaaggtggcggacctgcaggc**

**Sea81-4E**  **ctggttatgcgcaccacccacacgcgccgtgggcagcacccggcactgtatgcggagctgaaggtggcggacctgcaggc**

**MexicoAE**  **ctggttatgcgcaccacccacacgcgccgtgggcagcacccggcactgtatgcggagctgaaggtggcggacctgcaggc**

**Street14E**  **ctggttatgcgcaccacccacacgcgccgtgggcagcacccggcactgtatgcggagctgaaggtggcggacctgcaggc**

**GauthierE**  **ctggttatgcgcaccacccacacgcgccgtgggcagcacccggcactgtatgcggagctgaaggtggcggacctgcaggc**

**SamoaDE**  **ctggttatgcgcaccacccacacgcgccgtgggcagcacccggcactgtatgcggagctgaaggtggcggacctgcaggc**

**CDC2E**  **ctggttatgcgcaccacccacacgcgccgtgggcagcacccggcactgtatgcggagctgaaggtggcggacctgcaggc**

**BosniaAE**  **ctggttatgcgcaccacccacacgcgccgtgggcagcacccggcactgtatgcggagctgaaggtggcggacctgcaggc**

**IraqBE**  **ctggttatgcgcaccacccacacgcgccgtgggcagcacccggcactgtatgcggagctgaaggtggcggacctgcaggc**

**Fribourg-BlanCE** **ctggttatgcgcaccacccacacgcgccgtgggcagcacccggcactgtatgcggagctgaaggtggcggacctgcaggc**

**CuniculiAE**  **ctggttatgcgcaccacccacacgcgccgtgggcagcacccggcactgtatgcggagctgaaggtagcggacctgcaggc**

**330 340 350 360 370 380 390 400**

**....|....|....|....|....|....|....|....|....|....|....|....|....|....|....|....|**

**NicholsE**  **ggacctgagtcaggggaaggcaggttttgccgttaagcgcaaggggaaggtagaggcgacactacactgttatggggcct**

**Bal3E**  **ggacctgagtcaggggaaggcaggttttgccgttaagcgcaaggggaaggtagaggcgacactacactgttatggggcct**

**Sea81-4E**  **ggacctgagtcaggggaaggcaggttttgccgttaagcgcaaggggaaggtagaggcgacactacactgttatggggcct**

**MexicoAE**  **ggacctgagtcaggggaaggcaggttttgccgttaagcgcaaggggaaggtagaggcgacactacactgttatggggcct**

**Street14E**  **ggacctgagtcaggggaaggcaggttttgccgttaagcgcaaggggaaggtagaggcgacactacactgttatggggcct**

**GauthierE**  **ggacctgagtcaggggaaggcaggttttgccgttaagcgcaaggggaaggtagaggcgacactacactgttatggggcct**

**SamoaDE**  **ggacctgagtcaggggaaggtaggttttgccgttaagcgcaaggggaaggtagaggcgacactacactgttatggggcct**

**CDC2E**  **ggacctgagtcaggggaaggcaggttttgccgttaagcgcaaggggaaggtagaggcgacactacactgttatggggcct**

**BosniaAE**  **ggacctgagtcaggggaaggtaggttttgccgttaagcgcaaggggaaggtagaggcgacactacactgttatggggcct**

**IraqBE**  **ggacctgagtcaggggaaggtaggttttgccgttaagcgcaaggggaaggtagaggcgacactacactgttatggggcct**

**Fribourg-BlanCE** **ggacctgagtcaggggaaggcaggttttgccgttaagcgcaaggggaaggtagaggcgacactacactgttatggggcct**

**CuniculiAE**  **ggacctgagtcaggggaaggcaggttttgactttaagcgcaaggggaaggtggaggcgacgctgcactgttatggggcct**

**410 420 430 440 450 460 470 480**

**....|....|....|....|....|....|....|....|....|....|....|....|....|....|....|....|**

**NicholsE**  **acctgacgattgggaagaaccccacgtttctgacgaactttgcccggctgtggaagccgtgggtgacagcgcagtaccag**

**Bal3E**  **acctgacgattgggaagaaccccacgtttctgacgaactttgcccggctgtggaagccgtgggtgacagcgcagtaccag**

**Sea81-4E**  **acctgacgattgggaagaaccccacgtttctgacgaactttgcccggctgtggaagccgtgggtgacagcgcagtaccag**

**MexicoAE**  **acctgacgattgggaagaaccccacgtttctgacgaactttgcccggctgtggaagccgtgggtgacagcgcagtaccag**

**Street14E**  **acctgacgattgggaagaaccccacgtttctgacgaactttgcccggctgtggaagccgtgggtgacagcgcagtaccag**

**GauthierE**  **acctgacgattgggaagaaccccacgtttctgacgaactttgcccggctgtggaagccgtgggtgacagcgcagtaccag**

**SamoaDE**  **acctgacgattgggaagaaccccacgtttctgacgaactttgcccggctgtggaagccgtgggtgacagcgcagtaccag**

**CDC2E**  **acctgacgattgggaagaaccccacgtttctgacgaactttgcccggctgtggaagccgtgggtgacagcgcagtaccag**

**BosniaAE**  **acctgacgattgggaagaaccccacgtttctgacgaactttgcccggctgtggaagccgtgggtgacagcgcagtaccag**

**IraqBE**  **acctgacgattgggaagaaccccacgtttctgacgaactttgcccggctgtggaagccgtgggtgacagcgcagtaccag**

**Fribourg-BlanCE** **acctgacgattgggaagaaccccacgtttctgacgaacttcgcccggctgtggaagccgtgggtgacagcgcagtaccag**

**CuniculiAE**  **acctgacgattgggaagaaccccacgtttctgacgaactttgcccggctgtggaagccgtgggtgacagcgcagtaccag**

**490 500 510 520 530 540 550 560**

**....|....|....|....|....|....|....|....|....|....|....|....|....|....|....|....|**

**NicholsE**  **gaggatgcggtacagtatgcgccggggtttgggggtttaggcggcaaggttgggtatcgggcacaggacattgggggcag**

**Bal3E**  **gaggatgcggtacagtatgcgccggggtttgggggtttaggcggcaaggttgggtatcgggcacaggacattgggggcag**

**Sea81-4E**  **gaggatgcggtacagtatgcgccggggtttgggggtttaggcggcaaggttgggtatcgggcacaggacattgggggcag**

**MexicoAE**  **gaggatgcggtacagtatgcgccggggtttgggggtttaggcggcaaggttgggtatcgggcacaggacattgggggcag**

**Street14E**  **gaggatgcggtacagtatgcgccggggtttgggggtttaggcggcaaggttgggtatcgggcacaggacattgggggcag**

**GauthierE**  **gaggatgcggtacagtatgcgccggggtttgggggtttaggcggcaaggttgggtatcgggcacaggacattgggggcag**

**SamoaDE**  **gaggatgcggtacagtatgcgccggggtttgggggtttaggcggcaaggttgggtatcgggcacaggacattgggggcag**

**CDC2E**  **gaggatgcggtacagtatgcgccggggtttgggggtttaggcggcaaggttgggtatcgggcacaggacattgggggcag**

**BosniaAE**  **gaggatgcggtacagtatgcgccggggtttgggggtttaggcggcaaggttgggtatcgggcacaggacattgggggcag**

**IraqBE**  **gaggatgcggtacagtatgcgccggggtttgggggtttaggcggcaaggttgggtatcgggcacaggacattgggggcag**

**Fribourg-BlanCE** **gaggatgcggtacagtatgcgccggggtttgggggtttaggcggcaaggttgggtatcgggcacaggacattgggggcag**

**CuniculiAE**  **gaggatgaggtacagtatgcgccggggtttgggggtttaggcggcaaggttgggtatcgagcacaggacattgggggcag**

**570 580 590 600 610 620 630 640**

**....|....|....|....|....|....|....|....|....|....|....|....|....|....|....|....|**

**NicholsE**  **tggggtcagccttgatgtggggtttctctcctttgcctctaacggtgcctgggatagtactgaccccacgcacagtaagt**

**Bal3E**  **tggggtcagccttgatgtggggtttctctcctttgcctctaacggtgcctgggatagtactgaccccacgcacagtaagt**

**Sea81-4E**  **tggggtcagccttgatgtggggtttctctcctttgcctctaacggtgcctgggatagtactgaccccacgcacagtaagt**

**MexicoAE**  **tggggtcagccttgatgtggggtttctctcctttgcctctaacggtgcctgggatagtactgaccccacgcacagtaagt**

**Street14E**  **tggggtcagccttgatgtggggtttctctcctttgcctctaacggtgcctgggatagtactgaccccacgcacagtaagt**

**GauthierE**  **tggggtcagccttgatgtggggtttctctcctttgcctctaacggtgcctgggatagtactgaccccacgcacagtaagt**

**SamoaDE**  **tggggtcagccttgatgtggggtttctctcctttgcctctaacggtgcctgggatagtactgaccccacgcacagtaagt**

**CDC2E**  **tggggtcagccttgatgtggggtttctctcctttgcctctaacggtgcctgggatagtactgaccccacgcacagtaagt**

**BosniaAE**  **tggggtcagccttgatgtggggtttctctcctttgcctctaacggtgcctgggatagtactgaccccacgcacagtaagt**

**IraqBE**  **tggggtcagccttgatgtggggtttctctcctttgccactaacggtgcctgggatagtactgaccccacgcacagtaagt**

**Fribourg-BlanCE** **tggggtcagccttgatgtggggtttctctcctttgccactaacggtgcctgggatagtactgaccccacgcacagtaagt**

**CuniculiAE**  **tggggtcagccttgatgtggggtttctctcctttgcctctaacggtgcctgggatagtactgacaccacgcacagcaagt**

**650 660 670 680 690 700 710 720**

**....|....|....|....|....|....|....|....|....|....|....|....|....|....|....|....|**

**NicholsE**  **atggctttgggg-cagacttgaagctaatgtatgcgcgtgcaggacaccctctgtgcacggtagagcttgccagcaatgt**

**Bal3E**  **atggctttgggg-cagacttgaagctaatgtatgcgcgtgcaggacaccctctgtgcacggtagagcttgccagcaatgt**

**Sea81-4E**  **atggctttgggg-cagacttgaagctaatgtatgcgcgtgcaggacaccctctgtgcacggtagagcttgccagcaatgt**

**MexicoAE**  **atggctttgggg-cagacttgaagctaatgtatgcgcgtgcaggacaccctctgtgcacggtagagcttgccagcaatgt**

**Street14E**  **atggctttgggg-cagacttgaagctaatgtatgcgcgtgcaggacaccctctgtgcacggtagagcttgccagcaatgt**

**GauthierE**  **atggctttgggg-cagacttgaagctaatgtatgcgcgtgcaggacaccctctgtgcacggtagagcttgccagcaatgt**

**SamoaDE**  **atggctttgggg-cagacttgaagctaatgtatgcgcgtgcaggacaccctctgtgcacggtagagcttgccagcaatgt**

**CDC2E**  **atggctttgggg-cagacttgaagctaatgtatgcgcgtgcaggacaccctctgtgcacggtagagcttgccagcaatgt**

**BosniaAE**  **atggctttgggg-cagacttgaagctaatgtatgcgcgtgcaggacaccctctgtgcacggtagagcttgccagcaatgt**

**IraqBE**  **atggctttgggg-cagacttgaagctaatgtatgcgcgtgcaggacaccctctgtgcacggtagagcttgccagcaatgt**

**Fribourg-BlanCE** **atggctttgggg-cagacttgaagctaatgtatgcgcgtgcaggacaccctctgtgcacggtagagcttgccagcaatgt**

**CuniculiAE**  **atggctttgggggcagacttgaagctaatgtatgcgcgtgcaggacaccctctgtgcacggtagagcttgccagcaatgt**

**730 740 750 760 770 780 790 800**

**....|....|....|....|....|....|....|....|....|....|....|....|....|....|....|....|**

**NicholsE**  **tacgctagaagacggatacctcatcggtgcacagaaggacgcaaacaatcagaacaaggataaactgctgtggaatgtag**

**Bal3E**  **tacgctagaagacggatacctcatcggtgcacagaaggacgcaaacaatcagaacaaggataaactgctgtggaatgtag**

**Sea81-4E**  **tacgctagaagacggatacctcatcggtgcacagaaggacgcaaacaatcagaacaaggataaactgctgtggaatgtag**

**MexicoAE**  **tacgctagaagacggatacctcatcggtgcacagaaggacgcaaacaatcagaacaaggataaactgctgtggaatgtag**

**Street14E**  **tacgctagaagacggatacctcatcggtgcacagaaggacgcaaacaatcagaacaaggataaactgctgtggaatgtag**

**GauthierE**  **tacgctagaagacggatacctcatcggtgcacagaaggacgcaaacaatcagaacaaggataaactgctgtggaatgtag**

**SamoaDE**  **tacgctagaagacggatacctcatcggtgcacagaaggacgcaaacaatcagaacaaggataaactgctgtggaatgtag**

**CDC2E**  **tacgctagaagacggatacctcatcggtgcacagaaggacgcaaacaatcagaacaaggataaactgctgtggaatgtag**

**BosniaAE**  **tacgctagaagacggatacctcatcggtgcacagaaggacgcaaacaatcagaacaaggataaactgctgtggaatgtag**

**IraqBE**  **tacgctagaagacggatacctcatcggtgcacagaaggacgcaaacaatcagaacaaggataaactgctgtggaatgtag**

**Fribourg-BlanCE** **tacgctagaagacggatacctcatcggtgcacagaaggacgcaaacaatcagaacaaggataaactgctgtggaatgtag**

**CuniculiAE**  **tacgctggaagacggatacctcatcggtgcgcagaaggacgcaaacaatcagaacaaggataaactgctgtggaatgtag**

**810 820 830 840 850 860 870 880**

**....|....|....|....|....|....|....|....|....|....|....|....|....|....|....|....|**

**NicholsE**  **ggggccgactcaccctcgaaccaggcgccggcttccgcttctccttcgccctcgacgccggtaaccaacaccagagtgag**

**Bal3E**  **ggggccgactcaccctcgaaccaggcgccggcttccgcttctccttcgccctcgacgccggtaaccaacaccagagtgag**

**Sea81-4E**  **ggggccgactcaccctcgaaccaggcgccggcttccgcttctccttcgccctcgacgccggtaaccaacaccagagtgag**

**MexicoAE**  **ggggccgactcaccctcgaaccaggcgccggcttccgcttctccttcgccctcgacgccggtaaccaacaccagagtgag**

**Street14E**  **ggggccgactcaccctcgaaccaggcgccggcttccgcttctccttcgccctcgacgccggtaaccaacaccagagtgag**

**GauthierE**  **ggggccgactcaccctcgaaccaggcgccggcttccgcttctccttcgccctcgacgccggtaaccaacaccagagtgag**

**SamoaDE**  **ggggccgactcaccctcgaaccaggcgccggcttccgcttctccttcgccctcgacgccggtaaccaacaccagagtgag**

**CDC2E**  **ggggccgactcaccctcgaaccaggcgccggcttccgcttctccttcgccctcgacgccggtaaccaacaccagagtgag**

**BosniaAE**  **ggggccgactcaccctcgaaccaggcgccggcttccgcttctccttcgccctcgacgccggtaaccaacaccagagtgag**

**IraqBE**  **ggggccgactcaccctcgaaccaggcgccggcttccgcttctccttcgccctcgacgccggtaaccaacaccagagtgag**

**Fribourg-BlanCE** **ggggccgactcaccctcgaaccaggcgccggcttccgcttctccttcgccctcgacgccggtaaccaacaccagagtgag**

**CuniculiAE**  **gaggccgactcaccctcgaaccaggcgccggcttccgcttctccttcgccctcgacgccggtaaccaacgccagagtgca**

**890 900 910 920 930 940 950 960**

**....|....|....|....|....|....|....|....|....|....|....|....|....|....|....|....|**

**NicholsE**  **gctaccgcggcgatgaggaccgaaaggacacgcgagcgtgcacaggaggttgcactggcaatttttacgcacgctgcgca**

**Bal3E**  **gctaccgcggcgatgaggaccgaaaggacacgcgagcgtgcacaggaggttgcactggcaatttttacgcacgctgcgca**

**Sea81-4E**  **gctaccgcggcgatgaggaccgaaaggacacgcgagcgtgcacaggaggttgcactggcaatttttacgcacgctgcgca**

**MexicoAE**  **gctaccgcggcgatgaggaccgaaaggacacgcgagcgtgcacaggaggttgcactggcaatttttacgcacgctgcgca**

**Street14E**  **gctaccgcggcgatgaggaccgaaaggacacgcgagcgtgcacaggaggttgcactggcaatttttacgcacgctgcgca**

**GauthierE**  **gctaccgcggcgatgaggaccgaaaggacacgcgagcgtgcacaggaggttgcactggcaatttttacgcacgctgcgca**

**SamoaDE**  **gctaccgcggcgatgaggaccgaaaggacacgcgagcgtgcacaggaggttgcactggcaatttttacgcacgctgcgca**

**CDC2E**  **gctaccgcggcgatgaggaccgaaaggacacgcgagcgtgcacaggaggttgcactggcaatttttacgcacgctgcgca**

**BosniaAE**  **gctaccgcggcgatgaggaccgaaaggacacgcgagcgtgcacaggaggttgcactggcaatttttacgcacgctgcgca**

**IraqBE**  **gctaccgcggcgatgaggaccgaaaggacacgcgagcgtgcacaggaggttgcactggcaatttttacgcacgctgcgca**

**Fribourg-BlanCE** **gctaccgcggcgatgaggaccgaaaggacacgcgagcgtgcacaggaggttgcactggcaatttttacgcacgctgcgca**

**CuniculiAE**  **-----caagactttcaaaatcgc----acaca-gagggcgcagaatgaact-caccgccctctcaaataacctcttccag**

**970 980 990 1000 1010 1020 1030 1040**

**....|....|....|....|....|....|....|....|....|....|....|....|....|....|....|....|**

**NicholsE**  **ggaacaggctaaacaggcggctgatacggttggtagcac-catagataactcggtgcaggtggcaagatcagttattact**

**Bal3E**  **ggaacaggctaaacaggcggctgatacggttggtagcac-catagataactcggtgcaggtggcaagatcagttattact**

**Sea81-4E**  **ggaacaggctaaacaggcggctgatacggttggtagcac-catagataactcggtgcaggtggcaagatcagttattact**

**MexicoAE**  **ggaacaggctaaacaggcggctgatacggttggtagcac-catagataactcggtgcaggtggcaagatcagttattact**

**Street14E**  **ggaacaggctaaacaggcggctgatacggttggtagcac-catagataactcggtgcaggtggcaagatcagttattact**

**GauthierE**  **ggaacaggctaaacaggcggctgatacggttggtagcac-catagataactcggtgcaggtggcaagatcagttattact**

**SamoaDE**  **ggaacaggctaaacaggcggctgatacggttggtagcac-catagataactcggtgcaggtggcaagatcagttattact**

**CDC2E**  **ggaacaggctaaacaggcggctgatacggttggtagcac-catagataactcggtgcaggtggcaagatcagttattact**

**BosniaAE**  **ggaacaggctaaacaggcggctgatacggttggtagcac-catagataactcggtgcaggtggcaagatcagttattact**

**IraqBE**  **ggaacaggctaaacaggcggctgatacggttggtagcac-catagataactcggtgcaggtggcaagatcagttattact**

**Fribourg-BlanCE** **ggaacaggctaaacaggcggctgatacggttggtagcac-catagataactcggtgcaggtggcaagatcagttattact**

**CuniculiAE**  **ggagaaagtcaaaaa--caggaagcctggctggacgaatacgcaaagaa---ggtgcttgatgccgtaacggcagccacc**

**1050 1060 1070 1080 1090 1100 1110 1120**

**....|....|....|....|....|....|....|....|....|....|....|....|....|....|....|....|**

**NicholsE**  **cagatcgctgaaggagcggtgaagcaggcacacgatcagattaaacgcaccaatggaacacaagtagtgaatattgacgt**

**Bal3E**  **cagatcgctgaaggagcggtgaagcaggcacacgatcagattaaacgcaccaatggaacacaagtagtgaatattgacgt**

**Sea81-4E**  **cagatcgctgaaggagcggtgaagcaggcacacgatcagattaaacgcaccaatggaacacaagtagtgaatattgacgt**

**MexicoAE**  **cagatcgctgaaggagcggtgaagcaggcacacgatcagattaaacgcaccaatggaacacaagtagtgaatattgacgt**

**Street14E**  **cagatcgctgaaggagcggtgaagcaggcacacgatcagattaaacgcaccaatggaacacaagtagtgaatattgacgt**

**GauthierE**  **cagatcgctgaaggagcggtgaagcaggcacacgatcagattaaacgcaccaatggaacacaagtagtgaatattgacgt**

**SamoaDE**  **cagatcgctgaaggagcggtgaagcaggcacacgatcagattaaacgcaccaatggaacacaagtagtgaatattgacgt**

**CDC2E**  **cagatcgctgaaggagcggtgaagcaggcacacgatcagattaaacgcaccaatggaacacaagtagtgaatattgacgt**

**BosniaAE**  **cagatcgctgaaggagcggtgaagcaggcacacgatcagattaaacgcaccaatggaacacaagtagtgaatattgacgt**

**IraqBE**  **caggtcgctgaaggagcggtgaagcaggcacacgatcagattaaacgcaccaatggaacacaagtagtgaatattgacgt**

**Fribourg-BlanCE** **cagatcgctgaaggagcggtgaagcaggcacacgatcagattaaacgcaccaatggaacacaagtagtgaatattgacgt**

**CuniculiAE**  **gaaaccgccattcagtcgaggggaaacgcgtac-ataacggcagtgtcaaacgtaaaagtcacccctccggta--gctgc**

**1130 1140 1150 1160 1170 1180 1190 1200**

**....|....|....|....|....|....|....|....|....|....|....|....|....|....|....|....|**

**NicholsE**  **gaccgttccggtgaacgtccggcaaagtcctgttcggcaacctgacttgccttcacttaccgcaatcgcagcgcaattgc**

**Bal3E**  **gaccgttccggtgaacgtccggcaaagtcctgttcggcaacctgacttgccttcacttaccgcaatcgcagcgcaattgc**

**Sea81-4E**  **gaccgttccggtgaacgtccggcaaagtcctgttcggcaacctgacttgccttcacttaccgcaatcgcagcgcaattgc**

**MexicoAE**  **gaccgttccggtgaacgtccggcaaagtcctgttcggcaacctgacttgccttcacttaccgcaatcgcagcgcaattgc**

**Street14E**  **gaccgttccggtgaacgtccggcaaagtcctgttcggcaacctgacttgccttcacttaccgcaatcgcagcgcaattgc**

**GauthierE**  **gaccgttccggtgaacgtccggcaaagtcctgttcggcaacctgacttgccttcacttaccgcaatcgcagcgcaattgc**

**SamoaDE**  **gaccgttccggtgaacgtccggcaaagtcctgttcggcaacctgacttgccttcacttaccgcaatcgcagcgcaattgc**

**CDC2E**  **gaccgttccggtgaacgtccggcaaagtcctgttcggcaacctgacttgccttcacttaccgcaatcgcagcgcaattgc**

**BosniaAE**  **gaccgttccggtgaacgtccggcaaagtcctgttcggcaacctgacttgccttcacttaccgcaatcgcagcgcaattgc**

**IraqBE**  **gaccgttccggtgaacgtccggcaaagtcctgttcggcaacctgacttgccttcacttaccgcaatcgcagcgcaattgc**

**Fribourg-BlanCE** **gaccgttccggtgaacgtccggcaaagtcctgttcggcaacctgacttgccttcacttaccgcaatcgcagcgcaattgc**

**CuniculiAE**  **cacgcttttggcgaacctgaaggtgttcattaccgaccctcctacaccgtcaccgcttcccgcgcttcctgcatt-ttcc**

**1210 1220 1230 1240 1250 1260 1270 1280**

**....|....|....|....|....|....|....|....|....|....|....|....|....|....|....|....|**

**NicholsE**  **caaatgtaaccaagctcttcttccttagtgc-cggggcggccgccgcgaggcccattatcgggcagattactggcgtggt**

**Bal3E**  **caaatgtaaccaagctcttcttccttagtgc-cggggcggccgccgcgaggcccattatcgggcagattactggcgtggt**

**Sea81-4E**  **caaatgtaaccaagctcttcttccttagtgc-cggggcggccgccgcgaggcccattatcgggcagattactggcgtggt**

**MexicoAE**  **caaatgtaaccaagctcttcttccttagtgc-cggggcggccgccgcgaggcccattatcgggcagattactggcgtggt**

**Street14E**  **caaatgtaaccaagctcttcttccttagtgc-cggggcggccgccgcgaggcccattatcgggcagattactggcgtggt**

**GauthierE**  **caaatgtaaccaagctcttcttccttagtgc-cggggcggccgccgcgaggcccattatcgggcagattactggcgtggt**

**SamoaDE**  **caaatgtaaccaagctcttcttccttagtgc-cggggcggccgccgcgaggcccattatcgggcagattactggcgtggt**

**CDC2E**  **caaatgtaaccaagctcttcttccttagtgc-cggggcggccgccgcgaggcccattatcgggcagattactggcgtggt**

**BosniaAE**  **caaatgtaaccaagctcttcttccttagtgc-cggggcggccgccgcgaggcccattatcgggcagattactggcgtggt**

**IraqBE**  **caaatgtaaccaagctcttcttccttagtgc-cggggcggccgccgcgaggcccattatcgggcagattactggcgtggt**

**Fribourg-BlanCE** **caaatgtaaccaagctcttcttccttagtgc-cggggcggccgccgcgaggcccattatcgggcagattactggcgtggt**

**CuniculiAE**  **ctgatg-gggcaggttttgctgcagtatggcgtagagcaggtggtgaaggggtt-tgagcaggtacagacgcaaatcgtc**

**1290 1300 1310 1320 1330 1340 1350 1360**

**....|....|....|....|....|....|....|....|....|....|....|....|....|....|....|....|**

**NicholsE**  **gcagaacgttatcacccagcaggtacaggcccgggttgcgcagtcgaccgcggttgcaatc--cagcaagttcttgtgtt**

**Bal3E**  **gcagaacgttatcacccagcaggtacaggcccgggttgcgcagtcgaccgcggttgcaatc--cagcaagttcttgtgtt**

**Sea81-4E**  **gcagaacgttatcacccagcaggtacaggcccgggttgcgcagtcgaccgcggttgcaatc--cagcaagttcttgtgtt**

**MexicoAE**  **gcagaacgttatcacccagcaggtacaggcccgggttgcgcagtcgaccgcggttgcaatc--cagcaagttcttgtgtt**

**Street14E**  **gcagaacgttatcacccagcaggtacaggcccgggttgcgcagtcgaccgcggttgcaatc--cagcaagttcttgtgtt**

**GauthierE**  **gcagaacgttatcacccagcaggtacaggcccgggttgcgcagtcgaccgcggttgcaatc--cagcaagtttctgtgtt**

**SamoaDE**  **gcagaacgttatcacccagcaggtacaggcccgggttgcgcagtcgaccgcggttgcaatc--cagcaagtttctgtgtt**

**CDC2E**  **gcagaacgttatcacccagcaggtacaggcccgggttgcgcagtcgaccgcggttgcaatc--cagcaagtttctgtgtt**

**BosniaAE**  **gcagaacgttatcacccagcaggtacaggcccgggttgcgcagtcgaccgcggttgcaatc--cagcaagtttctgtgtt**

**IraqBE**  **gcagaacgttatcacccagcaggtacaggcccgggttgcgcagtcgaccgcggttgcaatc--cagcaagtttctgtgtt**

**Fribourg-BlanCE** **gcagaacgttatcacccagcaggtacaggcccgggttgcgcagtcgaccgcggttgcaatc--cagcaagtttctgtgtt**

**CuniculiAE**  **gctgaaattaatcagaaagtgcaaatagccgtgggt--cggagtagaactgcagcgcagacattctcaaatggcctcgcc**

**1370 1380 1390 1400 1410 1420 1430 1440**

**....|....|....|....|....|....|....|....|....|....|....|....|....|....|....|....|**

**NicholsE**  **caaccagcaaaccgtcgctgcagaaaaagcgaatacgcaaaagcatacgataaatggcaagtcatac--gcggctcatat**

**Bal3E**  **caaccagcaaaccgtcgctgcagaaaaagcgaatacgcaaaagcatacgataaatggcaagtcatac--gcggctcatat**

**Sea81-4E**  **caaccagcaaaccgtcgctgcagaaaaagcgaatacgcaaaagcatacgataaatggcaagtcatac--gcggctcatat**

**MexicoAE**  **caaccagcaaaccgtcgctgcagaaaaagcgaatacgcaaaagcatacgataaatggcaagtcatac--gcggctcatat**

**Street14E**  **caaccagcaaaccgtcgctgcagaaaaagcgaatacgcaaaagcatacgataaatggcaagtcatac--gcggctcatat**

**GauthierE**  **caaccagcaaaccgtcgctgcagaaaaagcgaatacgcaaaagcatacgataaatggcaagtcatac--gcggctcatat**

**SamoaDE**  **caaccagcaaaccgtcgctgcagaaaaagcgaatacgcaaaagcatacgataaatggcaagtcatac--gcggctcatat**

**CDC2E**  **caaccagcaaaccgtcgctgcagaaaaagcgaatacgcaaaagcatacgataaatggcaagtcatac--gcggctcatat**

**BosniaAE**  **caaccagcaaaccgtcgctgcagaaaaagcgaatacgcaaaagcatacgataaatggcaagtcatac--gcggctcatat**

**IraqBE**  **caaccagcaaaccgtcgctgcagaaaaagcgaatacgcaaaagcatacgataaatggcaagtcatac--gcggctcatat**

**Fribourg-BlanCE** **caaccagcaaaccgtcgctgcagaaaaagcgaatacgcaaaagcatacgataaatggcaagtcatac--gcggctcatat**

**CuniculiAE**  **caaacggtagcacg-catagcagacaagttgcttatgcctca-cataggaaatctacgcagccttttcaatgatccaaat**

**1450 1460 1470 1480 1490 1500 1510 1520**

**....|....|....|....|....|....|....|....|....|....|....|....|....|....|....|....|**

**NicholsE**  **cggctcgttggtaagtctcgctaccaacagggcgctgcctactatacgacagc-gtgttgagcaagctgttca--ggaaa**

**Bal3E**  **cggctcgttggtaagtctcgctaccaacagggcgctgcctactatacgacagc-gtgttgagcaagctgttca--ggaaa**

**Sea81-4E**  **cggctcgttggtaagtctcgctaccaacagggcgctgcctactatacgacagc-gtgttgagcaagctgttca--ggaaa**

**MexicoAE**  **cggctcgttggtaagtctcgctaccaacagggcgctgcctactatacgacagc-gtgttgagcaagctgttca--ggaaa**

**Street14E**  **cggctcgttggtaagtctcgctaccaacagggcgctgcctactatacgacagc-gtgttgagcaagctgttca--ggaaa**

**GauthierE**  **cggctcgttggtaagtctcgctaccaacagggcgctgcctactatacaacagc-gtgttaagcaagctgttca--ggaaa**

**SamoaDE**  **cggctcgttggtaagtctcgctaccaacagggcgctgcctactatacaacagc-gtgttaagcaagctgttca--ggaaa**

**CDC2E**  **cggctcgttggtaagtctcgctaccaacagggcgctgcctactatacaacagc-gtgttaagcaagctgttca--ggaaa**

**BosniaAE**  **cggctcgttggtaagtctcgctaccaacagggcgctgcctactatacaacagc-gtgttaagcaagctgttca--ggaaa**

**IraqBE**  **cggctcgttggtaagtctcgctaccaacagggcgctgcctactatacaacagc-gtgttaagcaagctgttca--ggaaa**

**Fribourg-BlanCE** **cggctcgttggtaagtctcgctaccaacagggcgctgcctactatacaacagc-gtgttaagcaagctgttca--ggaaa**

**CuniculiAE**  **caacaggcaacgttgcggcaagatcttacggatcttattccaaagcttacggctgaggctacaaagtttttcaccgaagg**

**1530 1540 1550 1560 1570 1580 1590 1600**

**....|....|....|....|....|....|....|....|....|....|....|....|....|....|....|....|**

**NicholsE**  **atatacggaggatcaacgctgtggtgcagcaaaaagcgcaaacgctcacctcttcccaggaactggaaaaggcagtgtat**

**Bal3E**  **atatacggaggatcaacgctgtggtgcagcaaaaagcgcaaacgctcacctcttcccaggaactggaaaaggcagtgtat**

**Sea81-4E**  **atatacggaggatcaacgctgtggtgcagcaaaaagcgcaaacgctcacctcttcccaggaactggaaaaggcagtgtat**

**MexicoAE**  **atatacggaggatcaacgctgtggtgcagcaaaaagcgcaaacgctcacctcttcccaggaactggaaaaggcagtgtat**

**Street14E**  **atatacggaggatcaacgctgtggtgcagcaaaaagcgcaaacgctcacctcttcccaggaactggaaaaggcagtgtat**

**GauthierE**  **atatacggaggatcaacgctgtggtgcagcaaaaagcgcaaacgctcacctcttcccaggaactggaaaaggcagtgtat**

**SamoaDE**  **atatacggaggatcaacgctgtggtgcagcaaaaagcgcaaacgctcacctcttcccaggaactggaaaaggcagtgtat**

**CDC2E**  **atatacggaggatcaacgctgtggtgcagcaaaaagcgcaaacgctcacctcttcccaggaactggaaaaggcagtgtat**

**BosniaAE**  **atatacggaggatcaacgctgtggtgcagcaaaaagcgcaaacgctcacctcttcccaggaactggaaaaggcagtgtat**

**IraqBE**  **atatacggaggatcaacgctgtggtgcagcaaaaagcgcaaacgctcacctcttcccaggaactggaaaaggcagtgtat**

**Fribourg-BlanCE** **atatacggaggatcaacgctgtggtgcagcaaaaagcgcaaacgctcacctcttcccaggaactggaaaaggcagtgtat**

**CuniculiAE**  **gcagacgtttg-taaccgaagaagtgaagaagaagacggatgcgttggacgcggggaagcagatacgtcaggctatacag**

**1610 1620 1630 1640 1650 1660 1670 1680**

**....|....|....|....|....|....|....|....|....|....|....|....|....|....|....|....|**

**NicholsE**  **tcgttgttcgttcccacgtttgaaaacctggtgttgggtgcaggcgcgctgctggctcttttggatatgcatcagattgc**

**Bal3E**  **tcgttgttcgttcccacgtttgaaaacctggtgttgggtgcaggcgcgctgctggctcttttggatatgcatcagattgc**

**Sea81-4E**  **tcgttgttcgttcccacgtttgaaaacctggtgttgggtgcaggcgcgctgctggctcttttggatatgcatcagattgc**

**MexicoAE**  **tcgttgttcgttcccacgtttgaaaacctggtgttgggtgcaggcgcgctgctggctcttttggatatgcatcagattgc**

**Street14E**  **tcgttgttcgttcccacgtttgaaaacctggtgttgggtgcaggcgcgctgctggctcttttggatatgcatcagattgc**

**GauthierE**  **tcgttgttcgttcccacgtttgaaaacctggtgttgggtgcaggcgcgctgctggctcttttggatatgcatcagattgc**

**SamoaDE**  **tcgttgttcgttcccacgtttgaaaacctggtgttgggtgcaggcgcgctgctggctcttttggatatgcatcagattgc**

**CDC2E**  **tcgttgttcgttcccacgtttgaaaacctggtgttgggtgcaggcgcgctgctggctcttttggatatgcatcagattgc**

**BosniaAE**  **tcgttgttcgttcccacgtttgaaaacctggtgttgggtgcaggcgcgctgctggctcttttggatatgcatcagattgc**

**IraqBE**  **tcgttgttcgttcccacgtttgaaaacctggtgttgggtgcaggcgcgctgctggctcttttggatatgcatcagattgc**

**Fribourg-BlanCE** **tcgttgttcgttcccacgtttgaaaacctggtgttgggtgcaggcgcgctgctggctcttttggatatgcatcagattgc**

**CuniculiAE**  **aacctgcgtgcgtctgcatggcgtgcctttctaatgggagtcagtgccgtgtgtctgtatcttgacacctacaatgtcgc**

**1690 1700 1710 1720 1730 1740 1750 1760**

**....|....|....|....|....|....|....|....|....|....|....|....|....|....|....|....|**

**NicholsE**  **ggtggacgcgctgtttacggcgcagtggaagtggctgtcttctggcatatactttgccacagcaccggcaaacgtttttg**

**Bal3E**  **ggtggacgcgctgtttacggcgcagtggaagtggctgtcttctggcatatactttgccacagcaccggcaaacgtttttg**

**Sea81-4E**  **ggtggacgcgctgtttacggcgcagtggaagtggctgtcttctggcatatactttgccacagcaccggcaaacgtttttg**

**MexicoAE**  **ggtggacgcgctgtttacggcgcagtggaagtggctgtcttctggcatatacgttgccacagcaccggcaaacgtttttg**

**Street14E**  **ggtggacgcgctgtttacggcgcagtggaagtggctgtcttctggcatatactttgccacagcaccggcaaacgtttttg**

**GauthierE**  **ggtggacgcgctgtttacagcgcagtggaagtggctgtcttctggcatatactttgccacagcaccggcaaacgtttttg**

**SamoaDE**  **ggtggacgcgctgtttacagcgcagtggaagtggctgtcttctggcatatactttgccacagcaccggcaaacgtttttg**

**CDC2E**  **ggtggacgcgctgtttacagcgcagtggaagtggctgtcttctggcatatactttgccacagcaccggcaaacgtttttg**

**BosniaAE**  **ggtggacgcgctgtttacagcgcagtggaagtggctgtcttctggcatatactttgccacagcaccggcaaacgtttttg**

**IraqBE**  **ggtggacgcgctgtttacagcgcagtggaagtggctgtcttctggcatatactttgccacagcaccggcaaacgtttttg**

**Fribourg-BlanCE** **ggtggacgcgctgtttacagcgcagtggaagtggctgtcttctggcatatactttgccacagcaccggcaaacgtttttg**

**CuniculiAE**  **cttcgatgcactgcttacagcgcagtggaagtggctgtcttctggcatatactttgccacagcaccggcaaacgtttttg**

**1770 1780 1790 1800 1810 1820 1830 1840**

**....|....|....|....|....|....|....|....|....|....|....|....|....|....|....|....|**

**NicholsE**  **gcaccagggtgttagataacaccatcgcaagctgtggcgactttgccggattccttaagctcgaaactaagagcggtgac**

**Bal3E**  **gcaccagggtgttagataacaccatcgcaagctgtggcgactttgccggattccttaagctcgaaactaagagcggtgac**

**Sea81-4E**  **gcaccagggtgttagataacaccatcgcaagctgtggcgactttgccggattccttaagctcgaaactaagagcggtgac**

**MexicoAE**  **gcaccagggtgttagataacaccatcgcaagctgtggcgactttgccggattccttaagctcgaaactaagagcggtgac**

**Street14E**  **gcaccagggtgttagataacaccatcgcaagctgtggcgactttgccggattccttaagctcgaaactaagagcggtgac**

**GauthierE**  **gcaccagggtgttagataacaccatcgcaagctgtggcgactttgccggattccttaagctcgaaactaagagcggtgac**

**SamoaDE**  **gcaccagggtgttagataacaccattgcaagctgtggcgactttgccggattccttaagctcgaaactaagagcggtgac**

**CDC2E**  **gcaccagggtgttagataacaccattgcaagctgtggcgactttgccggattccttaagctcgaaactaagagcggtgac**

**BosniaAE**  **gcaccagggtgttagataacaccattgcaagctgtggcgactttgccggattccttaagctcgaaactaagagcggtgac**

**IraqBE**  **gcaccagggtgttagataacaccattgcaagctgtggcgactttgccggattccttaagctcgaaactaagagcggtgac**

**Fribourg-BlanCE** **gcaccagggtgttagataacaccattgcaagctgtggcgactttgccggattccttaagctcgaaactaagagcggtgac**

**CuniculiAE**  **gcaccagggtgttagacaacaccattgcaagctgtggcgactttgccggattccttaaactcgaaactaaaagcggtgac**

**1850 1860 1870 1880 1890 1900 1910 1920**

**....|....|....|....|....|....|....|....|....|....|....|....|....|....|....|....|**

**NicholsE**  **ccctacacccacctgctcaccggcctggacgccggcgttgaaacacgcgtgtacatccccctcacctatgcgctatacaa**

**Bal3E**  **ccctacacccacctgctcaccggcctggacgccggcgttgaaacacgcgtgtacatccccctcacctatgcgctatacaa**

**Sea81-4E**  **ccctacacccacctgctcaccggcctggacgccggcgttgaaacacgcgtgtacatccccctcacctatgcgctatacaa**

**MexicoAE**  **ccctacacccacctgctcaccggcctggacgccggcgttgaaacacgcgtgtacatccccctcacctatgcgctatacaa**

**Street14E**  **ccctacacccacctgctcaccggcctggacgccggcgttgaaacacgcgtgtacatccccctcacctatgcgctatacaa**

**GauthierE**  **ccctacacccacctgctcaccggcctggacgccggcgttgaaacacgcatgtacatccccctcacctatgcgctatacaa**

**SamoaDE**  **ccctacacccacctgctcaccggcctggacgccggcgttgaaacacgcatgtacatccccctcacctatgcgctatacaa**

**CDC2E**  **ccctacacccacctgctcaccggcctggacgccggcgttgaaacacgcatgtacatccccctcacctatgcgctatacaa**

**BosniaAE**  **ccctacacccacctgctcaccggcctggacgccggcgttgaaacacgcatgtacatccccctcacctatgcgctatacaa**

**IraqBE**  **ccctacacccacctgctcaccggcctggacgccggcgttgaaacacgcatgtacatccccctcacctatgcgctatacaa**

**Fribourg-BlanCE** **ccctacacccacctgctcaccggcctggacgccggcgttgaaacacgcatgtacatccccctcacctatgcgctatacaa**

**CuniculiAE**  **ccctacacccacctgctcaccggcctggacgccggcgttgaaacacgcgtgtacatccccctcacccatgacccgtacaa**

**1930 1940 1950 1960 1970 1980 1990 2000**

**....|....|....|....|....|....|....|....|....|....|....|....|....|....|....|....|**

**NicholsE**  **aaataacggggggacggctgtgcgtggcattcaggaaaaggagtatatccgtccaccggtggtggggaaggcgtggtgta**

**Bal3E**  **aaataacggggggacggctgtgcgtggcattcaggaaaaggagtatatccgtccaccggtggtggggaaggcgtggtgta**

**Sea81-4E**  **aaataacggggggacggctgtgcgtggcattcaggaaaaggagtatatccgtccaccggtggtggggaaggcgtggtgta**

**MexicoAE**  **aaataacggggggacggctgtgcgtggcattcaggaaaaggagtatatccgtccaccggtggtggggaaggcgtggtgta**

**Street14E**  **aaataacggggggacggctgtgcgtggcattcaggaaaaggagtatatccgtccaccggtggtggggaaggcgtggtgta**

**GauthierE**  **aaataacggggggacggctgtgcgtggcattcaggaaaaggagtatatccgtccaccggtggtggggaaggcgtggtgta**

**SamoaDE**  **aaataacggggggacggctgtgcgtggcattcaggaaaaggagtatatccgtccaccggtggtggggaaggcgtggtgta**

**CDC2E**  **aaataacggggggacggctgtgcgtggcattcaggaaaaggagtatatccgtccaccggtggtggggaaggcgtggtgta**

**BosniaAE**  **aaataacggggggacggctgtgcgtggcattcaggaaaaggagtatatccgtccaccggtggtggggaaggcgtggtgta**

**IraqBE**  **aaataacggggggacggctgtgcgtggcattcaggaaaaggagtatatccgtccaccggtggtggggaaggcgtggtgta**

**Fribourg-BlanCE** **aaataacggggggacggctgtgcgtggcattcaggaaaaggagtatatccgtccaccggtggtggggaaggcgtggtgta**

**CuniculiAE**  **aaataataacgggaaccctctcccttccggcggctcctcagggcacattagcctgccgatagtgagcagggcgtggtgta**

**2010 2020 2030 2040 2050 2060 2070 2080**

**....|....|....|....|....|....|....|....|....|....|....|....|....|....|....|....|**

**NicholsE**  **gctatcgcatcccggtgcaggattacggctgggtgaagccaagcgttacggtccatgcctctaccaaccgtgcacacctg**

**Bal3E**  **gctatcgcatcccggtgcaggattacggctgggtgaagccaagcgttacggtccatgcctctaccaaccgtgcacacctg**

**Sea81-4E**  **gctatcgcatcccggtgcaggattacggctgggtgaagccaagcgttacggtccatgcctctaccaaccgtgcacacctg**

**MexicoAE**  **gctatcgcatcccggtgcaggattacggctgggtgaagccaagcgttacggtccatgcctctaccaaccgtgcacacctg**

**Street14E**  **gctatcgcatcccggtgcaggattacggctgggtgaagccaagcgttacggtccatgcctctaccaaccgtgcacacctg**

**GauthierE**  **gctatcgcatcccggtgcaggattacggctgggtgaagccaagcgttacggtccatgcctctaccaaccgtgcacacctg**

**SamoaDE**  **gctatcgcatcccggtgcaggattacggctgggtgaagccaagcgttacggtccatgcctctaccaaccgtgcacacctg**

**CDC2E**  **gctatcgcatcccggtgcaggattacggctgggtgaagccaagcgttacggtccatgcctctaccaaccgtgcacacctg**

**BosniaAE**  **gctatcgcatcccggtgcaggattacggctgggtgaagccaagcgttacggtccatgcctctaccaaccgtgcacacctg**

**IraqBE**  **gctatcgcatcccggtgcaggattacggctgggtgaagccaagcgttacggtccatgcctctaccaaccgtgcacacctg**

**Fribourg-BlanCE** **gctatcgcatcccggtgcaggattacggctgggtgaagccaagcgttacggtccatgcctctaccaaccgtgcacacctg**

**CuniculiAE**  **gctatcgcatcccggtgcaggattacggctgggtgaagccaagcgttacggtccacgcctctaccaaccgtgcacacctg**

**2090 2100 2110 2120 2130 2140 2150 2160**

**....|....|....|....|....|....|....|....|....|....|....|....|....|....|....|....|**

**NicholsE**  **aatgcccctgctgcaggtggagcagtaggagctacctatctaaccaaggagtactgtgcacagctgcgtgctggtatttc**

**Bal3E**  **aatgcccctgctgcaggtggagcagtaggagctacctatctaaccaaggagtactgtgcacagctgcgtgctggtatttc**

**Sea81-4E**  **aatgcccctgctgcaggtggagcagtaggagctacctatctaaccaaggagtactgtgcacagctgcgtgctggtatttc**

**MexicoAE**  **aatgcccctgctgcaggtggagcagtaggagctacctatctaaccaaggagtactgtgcacagctgcgtgctggtatttc**

**Street14E**  **aatgcccctgctgcaggtggagcagtaggagctacctatctaaccaaggagtactgtgcacagctgcgtgctggtatttc**

**GauthierE**  **aatgcccctgctgcaggcggagcagtaggagctacctatctaaccaaggagtactgtgcacagctgcgtgctggtatttc**

**SamoaDE**  **aatgcccctgctgcaggcggagcagtaggagctacctatctaaccaaggagtactgtgcacagctgcgtgctggtatttc**

**CDC2E**  **aatgcccctgctgcaggcggagcagtaggagctacctatctaaccaaggagtactgtgcacagctgcgtgctggtatttc**

**BosniaAE**  **aatgcccctgctgcaggcggagcagtaggagctacctatctaaccaaggagtactgtgcacagctgcgtgctggtatttc**

**IraqBE**  **aatgcccctgctgcaggcggagcagtaggagctacctatctaaccaaggagtactgtgcacagctgcgtgctggtatttc**

**Fribourg-BlanCE** **aatgcccctgctgcaggcggagcagtaggagctacctatctaaccaaggagtactgtgcacagctgcgtgctggtatttc**

**CuniculiAE**  **aatacccctgctgcaggcggagcagtaggagctacctatctaactaaggagtactgtgcacagctgcgtgctggtatttc**

**2170 2180 2190 2200 2210 2220 2230 2240**

**....|....|....|....|....|....|....|....|....|....|....|....|....|....|....|....|**

**NicholsE**  **agccagtctcatagagaagacggtattctcccttgattgggaacagggtatgctctctgatgtcccgtacctgctggtgt**

**Bal3E**  **agccagtctcatagagaagacggtattctcccttgattgggaacagggtatgctctctgatgtcccgtacctgctggtgt**

**Sea81-4E**  **agccagtctcatagagaagacggtattctcccttgattgggaacagggtatgctctctgatgtcccgtacctgctggtgt**

**MexicoAE**  **agccagtctcatagagaagacggtattctcccttgattgggaacagggtatgctctctgatgtcccgtacctgctggtgt**

**Street14E**  **agccagtctcatagagaagacggtattctcccttgattgggaacagggtatgctctctgatgtcccgtacctgctggtgt**

**GauthierE**  **agccagtctcatagagaagacggtattctcccttgattgggaacagggtatgctctctgatgtcccgtacctgctggtgt**

**SamoaDE**  **agccagtctcatagagaagacggtattctcccttgattgggaacagggtatgctctctgatgtcccgtacctgctggtgt**

**CDC2E**  **agccagtctcatagagaagacggtattctcccttgattgggaacagggtatgctctctgatgtcccgtacctgctggtgt**

**BosniaAE**  **agccagtctcatagagaagacggtattctcccttgattgggaacagggtatgctctctgatgtcccgtacctgctggtgt**

**IraqBE**  **agccagtctcatagagaagacggtattctcccttgattgggaacagggtatgctctctgatgtcccgtacctgctggtgt**

**Fribourg-BlanCE** **agccagtctcatagagaagacggtattctcccttgattgggaacagggtatgctctctgatgtcccgtacctgctggtgt**

**CuniculiAE**  **agccagtctcatagagaaggcggtgttctcccttgattgggaacagggtatgctctctgatgtcccgtacctgctggtat**

**2250 2260 2270 2280 2290**

**....|....|....|....|....|....|....|....|....|....|....|....**

**NicholsE**  **ccgagtgcctcacccagggaatcggccgcatcgtgtgcggcgtcaccctctcctggtag**

**Bal3E**  **ccgagtgcctcacccagggaatcggccgcatcgtgtgcggcgtcaccctctcctggtag**

**Sea81-4E**  **ccgagtgcctcacccagggaatcggccgcatcgtgtgcggcgtcaccctctcctggtag**

**MexicoAE**  **ccgagtgcctcacccagggaatcggccgcatcgtgtgcggcgtcaccctctcctggtag**

**Street14E**  **ccgagtgcctcacccagggaatcggccgcatcgtgtgcggcgtcaccctctcctggtag**

**GauthierE**  **ccgagtgcctcacccagggaatcggccgcatcgtgtgcggcgtcaccctctcctggtag**

**SamoaDE**  **ccgagtgcctcacccagggaatcggccgcatcgtgtgcggcgtcaccctctcctggtag**

**CDC2E**  **ccgagtgcctcacccagggaatcggccgcatcgtgtgcggcgtcaccctctcctggtag**

**BosniaAE**  **ccgagtgcctcacccagggaatcggccgcatcgtgtgcggcgtcaccctctcctggtag**

**IraqBE**  **ccgagtgcctcacccagggaatcggccgcatcgtgtgcggcgtcaccctctcctggtag**

**Fribourg-BlanCE** **ccgagtgcctcacccagggaatcggccgcatcgtgtgcggcgtcaccctctcctggtag**

**CuniculiAE**  **ccgagtgcctcacccagggaatcggccgcatcgtgtgcggcgtcaccctctcctggtag**

***1.5 tprF* and *tprI* loci**

10 20 30 40 50 60 70 80

....|....|....|....|....|....|....|....|....|....|....|....|....|....|....|....|

**NicholsF**  **MGRQVMQAGVLAGMVCAASGYAGVLTPQVSGTAQLQWGIAFQKNPRTGPGKHTHGFRTTNSLTISLPLVSKHTHTRRGEA**

**Bal3F**  **MGRQVMQAGVLAGMVCAASGYAGVLTPQVSGTAQLQWGIAFQKNPRTGPGKHTHGFRTTNSLTISLPLVSKHTHTRRGEA**

**Sea81-4F**  **MGRQVMQAGVLAGMVCAASGYAGVLTPQVSGTAQLQWGIAFQKNPRTGPGKHTHGFRTTNSLTISLPLVSKHTHTRRGEA**

**MexicoAF**  **MGRQVMQAGVLAGMVCAASGYAGVLTPQVSGTAQLQWGIAFQKNPRTGPGKHTHGFRTTNSLTISLPLVSKHTHTRRGEA**

**Street14F MGRQVMQAGVLAGMVCAASGYAGVLTPQVSGTAQLQWGIAFQKNPRTGPGKHTHGFRTTNSLTISLPLVSKHTHTRRGEA**

**NicholsI**  **MGRQVMQAGVLAGMVCAASGYAGVLTPQVSGTAQLQWGIAFQKNPRTGPGKHTHGFRTTNSLTISLPLVSKHTHTRRGEA**

**Bal3I MGRQVMQAGVLAGMVCAASGYAGVLTPQVSGTAQLQWGIAFQKNPRTGPGKHTHGFRTTNSLTISLPLVSKHTHTRRGEA**

**Sea81-4I**  **MGRQVMQAGVLAGMVCAASGYAGVLTPQVSGTAQLQWGIAFQKNPRTGPGKHTHGFRTTNSLTISLPLVSKHTHTRRGEA**

**MexicoAI**  **MGRQVMQAGVLAGMVCAASGYAGVLTPQVSGTAQLQWGIAFQKNPRTGPGKHTHGFRTTNSLTISLPLVSKHTHTRRGEA**

**Street14I MGRQVMQAGVLAGMVCAASGYAGVLTPQVSGTAQLQWGIAFQKNPRTGPGKHTHGFRTTNSLTISLPLVSKHTHTRRGEA**

**GauthierF**  **MGRQVMQAGVLAGMVCAASGYAGVLTPQVSGTAQLQWGIAFQKNPHTVPGEHTHGFRTTNSLTISLPLVSKHTHTRRGEA**

**GauthierI**  **MGRQVMQAGVLAGMVCAASGYAGVLTPQVSGTAQLQWGIAFQKNPHTVPGEHTHGFRTTNSLTISLPLVSKHTHTRRGEA**

**SamoaDF**  **MGRQVMQAGVLAGMVCAASGYAGVLTPQVSGTAQLQWGIAFQKNPHTVPGEHTHGFRTTNSLTISLPLVSKHTHTRRGEA**

**SamoaDI MGRQVMQAGVLAGMVCAASGYAGVLTPQVSGTAQLQWGIAFQKNPHTVPGEHTHGFRTTNSLTISLPLVSKHTHTRRGEA**

**CDC2F**  **MGRQVMQAGVLAGMVCAASGYAGVLTPQVSGTAQLQWGIAFQKNPHTVPGEHTHGFRTTNSLTISLPLVSKHTHTRRGEA**

**CDC2I**  **MGRQVMQAGVLAGMVCAASGYAGVLTPQVSGTAQLQWGIAFQKNPHTVPGEHTHGFRTTNSLTISLPLVSKHTHTRRGEA**

**BosniaAI MGRQVMQAGVLAGMVCAASGYAGVLTPQVSGTAQLQWGIAFQKNPRTVPGEHTHGFRTTNSLTISLPLVSKHTHTRRGEA**

**IraqBI MGRQVMQAGVLAGMVCAASGYAGVLTPQVSGTAQLQWGIAFQKNPRTVPGEHTHGFRTTNSLTISLPLVSKHTHTRRGEA**

**Fribourg-BlancF** **MGRQVMQAGVLAGMVCAASGYAGVLTPQVSGTAQLQWGIAFQKNPHTVPGEHTHGFRTTNSLTISLPLVSKHTHTRRGEA**

**Fribourg-BlancI** **MGRQVMQAGVLAGMVCAASGYAGVLTPQVSGTAQLQWGIAFQKNPHTVPGEHTHGFRTTNSLTISLPLVSKHTHTRRGEA**

90 100 110 120 130 140 150 160

....|....|....|....|....|....|....|....|....|....|....|....|....|....|....|....|

**NicholsF**  **RSGVWAQLQLKDLAVELASSKSSTALSFTKPTASFQATLHCYGAYLTVGTSPSCVVNFAQLWKPFVTRAYSEKDTRYAPG**

**Bal3F**  **RSGVWAQLQLKDLAVELASSKSSTALSFTKPTASFQATLHCYGAYLTVGTSPSCVVNFAQLWKPFVTRAYSEKDTRYAPG**

**Sea81-4F**  **RSGVWAQLQLKDLAVELASSKSSTALSFTKPTASFQATLHCYGAYLTVGTSPSCVVNFAQLWKPFVTRAYSEKDTRYAPG**

**MexicoAF**  **RSGVWAQLQLKDLAVELASSKSSTALSFTKPTASFQATLHCYGAYLTVGTSPSCVVNFAQLWKPFVTRAYSEKDTRYAPG**

**Street14F RSGVWAQLQLKDLAVELASSKSSTALSFTKPTASFQATLHCYGAYLTVGTSPSCVVNFAQLWKPFVTRAYSEKDTRYAPG**

**NicholsI**  **RSGVWAQLQLKDLAVELASSKSSTALSFTKXTASFQATLHCYGAYLTVGTSPSCVVNFAQLWKPFVTRAYSEKDTRYAPG**

**Bal3I RSGVWAQLQLKDLAVELASSKSSTALSFTKPTASFQATLHCYGAYLTVGTSPSCVVNFAQLWKPFVTRAYSEKDTRYAPG**

**Sea81-4I**  **RSGVWAQLQLKDLAVELASSKSSTALSFTKPTASFQATLHCYGAYLTVGTSPSCVVNFAQLWKPFVTRAYSEKDTRYAPG**

**MexicoAI**  **RSGVWAQLQLKDLAVELASSKSSTALSFTKPTASFQATLHCYGAYLTVGTSPSCVVNFAQLWKPFVTRAYSEKDTRYAPG**

**Street14I RSGVWAQLQLKDLAVELASSKSSTALSFTKXTASFQATLHCYGAYLTVGTSPSCVVNFAQLWKPFVTRAYSEKDTRYAPG**

**GauthierF**  **RLGVWAQLQLKDLAVELASSKSSTALSFTKPTASFQATLHCYGAYLTVGTSPSCVVNFAQLWKPFVTRAYSEKDTRYAPG**

**GauthierI**  **RLGVWAQLQLKDLAVELASSKSSTALSFTKPTASFQATLHCYGAYLTVGTSPSCVVNFAQLWKPFVTRAYSEKDTRYAPG**

**SamoaDF**  **RSGVWAQLQLKDLAVELASSKSSTALSFTKPTASFQATLHCYGAYLTVGTSPSCVVNFAQLWKPFVTRAYSEKDTRYAPG**

**SamoaDI RSGVWAQLQLKDLAVELASSKSSTALSFTKPTASFQATLHCYGAYLTVGTSPSCVVNFAQLWKPFVTRAYSEKDTRYAPG**

**CDC2F**  **RSGVWAQLQLKDLAVELASSKSSTALSFTKPTASFQATLHCYGAYLTVGTSPSCVVNFAQLWKPFVTRAYSEKDTRYAPG**

**CDC2I**  **RSGVWAQLQLKDLAVELASSKSSTALSFTKPTASFQATLHCYGAYLTVGTSPSCVVNFAQLWKPFVTRAYSEKDTRYAPG**

**BosniaAI RSGVWAQLQLKDLAVELASSKSSTALSFTKPTASFQATLHCYGAYLTVGTSPSCVVNFAQLWKPFVTRAYSEKDTRYAPG**

**IraqBI RSGVWAQLQLKDLAVELASSKSSTALSFTKPTASFQATLHCYGAYLTVGTSPSCVVNFAQLWKPFVTRAYSEKDTRYAPG**

**Fribourg-BlancF** **RSGVWAQLQLKDLAVELASSKSSTALSFTKPTASFQATLHCYGAYLTVGTSPSCVVNFAQLWKPFVTRAYSEKDTRYAPG**

**Fribourg-BlancI** **RSGVWAQLQLKDLAVELASSKSSTALSFTKPTASFQATLHCYGAYLTVGTSPSCVVNFAQLWKPFVTRAYSEKDTRYAPG**

170 180 190 200 210 220 230 240

....|....|....|....|....|....|....|....|....|....|....|....|....|....|....|....|

**NicholsF**  **FSGSGAKLGYQAHNVGNSGVDVDIGFLSFLSNGAWDSTDTTHSKYGFGADATLSYGVDRQRLLTLELAGNATLEQHYRKG**

**Bal3F**  **FSGSGAKLGYQAHNVGNSGVDVDIGFLSFLSNGAWDSTDTTHSKYGFGADATLSYGVDRQRLLTLELAGNATLEQHYRKG**

**Sea81-4F**  **FSGSGAKLGYQAHNVGNSGVDVDIGFLSFLSNGAWDSTDTTHSKYGFGADATLSYGVDRQRLLTLELAGNATLEQHYRKG**

**MexicoAF**  **FSGSGAKLGYQAHNVGNSGVDVDIGFLSFLSNGAWDSTDTTHSKYGFGADATLSYGVDRQRLLTLELAGNATLEQHYRKG**

**Street14F FSGSGAKLGYQAHNVGNSGVDVDIGFLSFLSNGAWDSTDTTHSKYGFGADATLSYGVDRQRLLTLELAGNATLEQHYRKG**

**NicholsI**  **FSXSGAKLGYQAHNVGNSGVDVDIGFLSFLSNGAWDSTDTTHSKYGFGADATLSYGVDRQRLLTLELAGNATLEQHYRKG**

**Bal3I FSGSGAKLGYQAHNVGNSGVDVDIGFLSFLSNGAWDSTDTTHSKYGFGADATLSYGVDRQRLLTLELAGNATLEQHYRKG**

**Sea81-4I**  **FSGSGAKLGYQAHNVGNSGVDVDIGFLSFLSNGAWDSTDTTHSKYGFGADATLSYGVDRQRLLTLELAGNATLEQHYRKG**

**MexicoAI**  **FSGSGAKLGYQAHNVGNSGVDVDIGFLSFLSNGAWDSTDTTHSKYGFGADATLSYGVDRQRLLTLELAGNATLEQHYRKG**

**Street14I FSXSGAKLGYQAHNVGNSGVDVDIGFLSFLSNGAWDSTDTTHSKYGFGADATLSYGVDRQRLLTLELAGNATLDQNYVKG**

**GauthierF**  **FSGSGAKLGYQAHNVGNSGVDVDIGFLSFLSNGAWDSTDTTHSKYGFGADATLSYGVDRQRLLTLELAGNATLEQHYRKG**

**GauthierI**  **FSGSGAKLGYQAHNVGNSGVDVDIGFLSFLSNGAWDSTDTTHSKYGFGADATLSYGVDRQRLLTLELAGNATLEQHYRKG**

**SamoaDF**  **FSGSGAKLGYQAHNVGNSGVDVDIGFLSFLSNGAWDSTDTTHSKYGFGADATLSYGVDRQRLLTLELAGNATLEQHYRKG**

**SamoaDI FSGSGAKLGYQAHNVGNSGVDVDTGFLSFLSNGAWDSTDPTHSKYGFGADATLSYGVDRQRLLTLELAGNATLEQHYRKG**

**CDC2F**  **FSGSGAKLGYQAHNVGNSGVDVDIGFLSFLSNGAWDSTDTTHSKYGFGADATLSYGVDRQRLLTLELAGNATLEQHYRKG**

**CDC2I**  **FSGSGAKLGYQAHNVGNSGIDVDIGFLSFLSNGAWDSTDTTHSKYGFGADATLSYGVDRQRLLTLELAGNATLEQHYRKG**

**BosniaAI FSGSGAKLGYQAHNVGNSGVDVDIGFLSFLSNGAWDSTDTMHSKYGFGADATLSYGVDRQRLLTLELAGNATLEQHYRKG**

**IraqBI FSGSGAKLGYQAHNVGNSGVDVDIGFLSFLSNGAWDSTDTMHSKYGFGADATLSYGVDRQRLLTLELAGNATLEQHYRKG**

**Fribourg-BlancF** **FSGSGAKLGYQAHNVGNSGVDVDIGFLSFLSNGAWDSTDATHSKYGFGADATLSYGVDRQRLLTLELAGNATLEQHYRKG**

**Fribourg-BlancI** **FSGSGAKLGYQAHNVGNSGVDVDTGFLSFLSNGAWDSTDPTHSKYGFGADATLSYGVDRQRLLTLELAGNATLEQHYRKG**

250 260 270 280 290 300 310 320

....|....|....|....|....|....|....|....|....|....|....|....|....|....|....|....|

**NicholsF**  **TEDSTNENKTALLWGVGGRLTLEPGAGFRFSFALDAGNQHQDPADAGNRLLATGSSREKFDSAFDALRVEQYRVKDKYLE**

**Bal3F**  **TEDSTNENKTALLWGVGGRLTLEPGAGFRFSFALDAGNQHQDPADAGNRLLATGSSREKFDSAFDALRVEQYRVKDKYLE**

**Sea81-4F**  **TEDSTNENKTALLWGVGGRLTLEPGAGFRFSFALDAGNQHQDPADAGNRLLATGSSREKFDSAFDALRVEQYRVKDKYLE**

**MexicoAF**  **TEDSTNENKTALLWGVGGRLTLEPGAGFRFSFALDAGNQHQDPADAGNRLLATGSSREKFDSAFDALRVEQYRVKDKYLE**

**Street14F TEDSTNENKTALLWGVGGRLTLEPGAGFRFSFALDAGNQHQDPADAGNRLLATGSSREKFDSAFDALRVEQYRVKDKYLE**

**NicholsI**  **TEDSTNENKTALLWGVGGRLTLEPGAGFRFSFALDAGNQHQDPADAGNRLLATGSSREKFDSAFDALRVEQYRVKDKYLE**

**Bal3I TEDSTNENKTALLWGVGGRLTLEPGAGFRFSFALDAGNQHQDPADAGNRLLATGSSREKFDSAFDALRVEQYRVKDKYLE**

**Sea81-4I**  **TEDSTNENKTALLWGVGGRLTLEPGAGFRFSFALDAGNQHQDPADAGNRLLATGSSREKFDSAFDALRVEQYRVKDKYLE**

**MexicoAI**  **TEDSTNENKTALLWGVGGRLTLEPGAGFRFSFALDAGNQHQDPADAGNRLLATGSSREKFDSAFDALRVEQYRVKDKYLE**

**Street14I TEDSTNENKTALLWGVGGRLTLEPGAGFRFSFALDAGNQHQDPADAGNRLLATGSSREKFDSAFDALRVEQYRVKDKYLE**

**GauthierF**  **TEDSPNENKTALPWGVGGRLTLEPGAGFRFSFALDAGNQHQSNADADCRLPATGNSREKFDRAFDALRVEQYRVKDKYLE**

**GauthierI**  **TEDSPNENKTALPWGVGGRLTLEPGAGFRFSFALDAGNQHQSNADADCRLPATGNSREKFDRAFDALRVEQYRVKDKYLE**

**SamoaDF**  **TEDSTNENKTALLWGVGGRLTLEPGAGFRFSFALDAGNQHQSNADADCRLPATGSSREKFDRAFDALRVEQYRVKDKYLE**

**SamoaDI TEDSTNENKTALLWGVGGRLTLEPGAGFRFSFALDAGNQHQSNADADCRLPATGSSREKFDRAFDALRVEQYRVKDKYLE**

**CDC2F**  **TEDSTNENKTALLWGVGGRLTLEPGAGFRFSFALDAGNQHQSNADADCRLPATGSSREKFDRAFDALRVEQYRVKDKYLE**

**CDC2I**  **TEDSTNENKTALLWGVGGRLTLEPGAGFRFSFALDAGNQHQSNADADCRLPATGSSREKFDRAFDALRVEQYRVKDKYLE**

**BosniaAI TEDSTNENKTALLWGVGGRLTLEPGAGFRFSFALDAGNQHQSNADADCRLPATGSSREKFDRAFDALRVEQYRVKDKYLE**

**IraqBI TEDSTNENKTALLWGVGGRLTLEPGAGFRFSFALDAGNQHQSNADADCRLPATGSSREKFDRAFDALRVEQYRVKDKYLE**

**Fribourg-BlancF** **TEDSTNENKTALLWGVGGRLTLEPGAGFRFSFALDAGNQHQSDADADCRLPATGSSREKFDRAFDALRVEQYYVKDKYLE**

**Fribourg-BlancI** **TEDSTNENKTALLWGVGGRLTLEPGAGFRFSFALDAGNQHQSDADADCRLPATGSSREKFDRAFDALRVEQYYVKDKYLE**

330 340 350 360 370 380 390 400

....|....|....|....|....|....|....|....|....|....|....|....|....|....|....|....|

**NicholsF**  **FLLGQMAESSILERVGLALTLQDGTLVSTLTKVATDSGAQRPVGTGGAC**

**Bal3F**  **FLLGQMAESSILERVGLALTLQDGTLVSTLTKVATDSGAQRPVGTGGAC**

**Sea81-4F**  **FLLGQMAESSILERVGLALTLQDGTLVSTLTKVATDSGAQRPVGTGGAC**

**MexicoAF**  **FLLGQMAESSILERVGLALTLQDGTLVSTLTKVATDSGAQRPVGTGGAC**

**Street14F FLLGQMAESSILERVGLALTLQDGTLVSTLTKVATDSGAQRPVGTGGAC**

**NicholsI**  **FLLGQMAESSILERVGLALTLQDGTLVSTLTKVATDSGDRFIQMALVKLLPQRAQAEQRLQEIVAPSQSDIVLIMLLTWL**

**Bal3I FLLGQMAESSILERVGLALTLQDGTLVSTLTKVATDSGDRFIQMALVKLLPQRAQAEQRLQEIVAPSQSDIVLIMLLTWL**

**Sea81-4I**  **FLLGQMAESSILERVGLALTLQDGTLVSTLTKVATDSGDRFIQMALVKLLPQRAQAEQRLQEIVAPSQSDIVLIMLLTWL**

**MexicoAI**  **FLLGQMAESSILERVGLALTLQDGTLVSTLTKVATDSGDRFIQMALVKLLPQRAQAEQRLQEIVAPSQSDIVLIMLLTWL**

**Street14I FLLGQMAESSILERVGLALTLQDGTLVSTLTKVATDSGDRFIQMALVKLLPQRAQAEQRLQEIVAPSQSDIVLIMLLTWL**

**GauthierF**  **FLLGQTAESSILERVGLALTLQDGTLVSTLTKVATDSGDRFIQMALVKLLPQRAQAEQGLREIVAPSQSDIVLIMLLTWL**

**GauthierI**  **FLLGQTAESSILERVGLALTLQDGTLVSTLTKVATDSGDRFIQMALVKLLPQRAQAEQGLREIVAPSQSDIVLIMLLTWL**

**SamoaDF**  **FLLGQMAESSILERVGLALTLQDGTLVSTLTKVATDSGDWFIQMALVKLLPQRAQAEQGLREIVAPSQSDIVLIMLLTWL**

**SamoaDI FLLGQMAESSILERVGLALTLQDGTLVSTLTKVATDSGDRFIQMALVKLLPQRAQAEQGLREIVAPSQSDIVLIMLLTWL**

**CDC2F**  **FLLGQMAESSILERVGLALTLQDGTLVSTLTKVATDSGDWFIQMALVKLLPQRAQAEQGLREIVAPSQSDIVLIMLLTWL**

**CDC2I**  **FLLGQMAESSILERVGLALTLQDGTLVSTLTKVATDSGDWFIQMALVKLLPQRAQAEQGLREIVAPSQSDIVLIMLLTWL**

**BosniaAI FLLGQMAESSILERVGLALTLQDGTLVSTLTKVATDSGDRFIQMALVKLLPQRAQAEQGLREIVAPSQSDIVLIMLLTWL**

**IraqBI FLLGQMAESSILERVGLALTLQDGTLVSTLTKVATDSGDRFIQMALVKLLPQRAQAEQGLREIVAPSQSDIVLIMLLTWL**

**Fribourg-BlancF** **FLLGQMAESSILERVGLALTLQDGTLVSTLTKVATDSGDRFIQMALVKLLPQRAQAEQGLREIVAPSQSDIVLIMLLTWL**

**Fribourg-BlancI** **FLLGQMAESSILERVGLALTLQDGTLVSTLTKVATDSGDQFIQMALVKLLPQRAQAEQGLREIVAPSQSDIVLIMLLTWL**

410 420 430 440 450 460 470 480

....|....|....|....|....|....|....|....|....|....|....|....|....|....|....|....|

**NicholsF**

**Bal3F**

**Sea81-4F**

**MexicoAF**

**Street14F**

**NicholsI**  **ERARLDRFNADALLTAQWTYVSAGLYGATAGTNVFGKRVLPALRSWHFDFAGFLKLETKSGDPYTHLLTGLNAGVEARVY**

**Bal3I ERARLDRFNADALLTAQWTYVSAGLYGATAGTNVFGKRVLPALRSWHFDFAGFLKLETKSGDPYTHLLTGLNAGVEARVY**

**Sea81-4I**  **ERARLDRFNADALLTAQWTYVSAGLYGATAGTNVFGKRVLPALRSWHFDFAGFLKLETKSGDPYTHLLTGLNAGVEARVY**

**MexicoAI**  **ERARLDRFNADALLTAQWTYVSAGLYGATAGTNVFGKRVLPALRSWHFDFAGFLKLETKSGDPYTHLLTGLNAGVEARVY**

**Street14I ERARLDRFNADALLTAQWTYVSAGLYGATAGTNVFGKRVLPALRSWHFDFAGFLKLETKSGDPYTHLLTGLNAGVEARVY**

**GauthierF**  **ERARLDRFNADALLTAQWTYVSAGLYGATAGTNVFGKRVLPALQSWHFDFAGFLKLETKSGDPYTHLLTGLNAGVEARVY**

**GauthierI**  **ERARLDRFNADALLTAQWTYVSAGLYGATAGTNVFGKRVLPALQSWHFDFAGFLKLETKSGDPYTHLLTGLNAGVEARVY**

**SamoaDF**  **ERARLDRFNADALLTAQWTYVSAGLYGATAGTNVFGKRVLPALQSWHFDFAGFLKLETKSGDPYTHLLTGLNAGVEARVY**

**SamoaDI ERARLDRFNADALLTAQWTYVSAGLYGATAGTNVFGKRVLPALQSWHFDFAGFLKLETKSGDPYTHLLTGLNAGVEARVY**

**CDC2F**  **ERARLDRFNADALLTAQWTYVSAGLYGATAGTNVFGKRVLPALQSWHFDFAGFLKLETKSGDPYTHLLTGLNAGVEARVY**

**CDC2I**  **ERARLDRFNADALLTAQWTYVSAGLYGATAGTNVFGKRVLPALQSWHFDFAGFLKLETKSGDPYTHLLTGLNAGVEARVY**

**BosniaAI ERARLDRFNADALLTAQWTYVSAGLYGATAGTNVFGKRVLPALQSWHFDFAGFLKLETKSGDPYTHLLTGLNAGVEARVY**

**IraqBI ERARLDRFNADALLTAQWTYVSAGLYGATAGTNVFGKRVLPALQSWHFDFAGFLKLETKSGDPYTHLLTGLNAGVEARVY**

**Fribourg-BlancF** **ERARLDRFNADALLTAQWTYVSAGLYGATAGTNVFGKRVLPALQSWHFDFAGFLKLETKSGDPYTHLLTGLNAGVEARVY**

**Fribourg-BlancI** **ERARLDRFNADALLTAQWTYVSAGLYGATAGTNVFGKRVLPALQSWHFDFAGFLKLETKSGDPYTHLLTGLNAGVEARVY**

490 500 510 520 530 540 550 560

....|....|....|....|....|....|....|....|....|....|....|....|....|....|....|....|

**NicholsF**

**Bal3F**

**Sea81-4F**

**MexicoAF**

**Street14F**

**NicholsI**  **IPLTYIRYRNNGGYELNGAVPPGTINMPILGKAWCSYRIPLGSHAWLTPHTSVLGTTNRFNVINPAYTLLNERALQYQVG**

**Bal3I IPLTYIRYRNNGGYELNGAVPPGTINMPILGKAWCSYRIPLGSHAWLTPHTSVLGTTNRFNVINPAYTLLNERALQYQVG**

**Sea81-4I**  **IPLTYIRYRNNGGYELNGAVPPGTINMPILGKAWCSYRIPLGSHAWLTPHTSVLGTTNRFNVINPAYTLLNERALQYQVG**

**MexicoAI**  **IPLTYIRYRNNGGYELNGAVPPGTINMPILGKAWCSYRIPLGSHAWLTPHTSVLGTTNRFNVINPAYTLLNERALQYQVG**

**Street14I IPLTYIRYRNNGGYELNGAVPPGTINMPILGKAWCSYRIPLGSHAWLTPHTSVLGTTNRFNVINPAYTLLNERALQYQVG**

**GauthierF**  **IPLTYIRYRNNGGYPLNGVVPPGTINMPILGKAWCSYRIPLGSHAWLTPHTSVLGTTNRFNVINPAHTLLNERALQYQVG**

**GauthierI**  **IPLTYIRYRNNGGYPLNGVVPPGTINMPILGKAWCSYRIPLGSHAWLTPHTSVLGTTNRFNVINPAHTLLNERALQYQVG**

**SamoaDF**  **IPLTYIRYRNNGGYPLNGVVPPGTINMPILGKAWCSYRIPLGSHAWLTPHTSVLGTTNRFNVINPAYTLLNERALQYQVG**

**SamoaDI IPLTYIRYRNNGGYPLNGVVPPGTINMPILGKAWCSYRIPLGSHAWLTPHTSVLGTTNRFNVINPAYTLLNERALQYQVG**

**CDC2F**  **IPLTYIRYRNNGGYPLNGVVPPGTINMPILGKAWCSYRIPLGSHAWLTPHTSVLGTTNRFNVINPAYTLLNERALQYQVG**

**CDC2I**  **IPLTYIRYRNNGGYPLNGVVPPGTINMPILGKAWCSYRIPLGSHAWLTPHTSVLGTTNRFNVINPAYTLLNERALQYQVG**

**BosniaAI IPLTYIRYRNNGGYPLNGVVPPGTINMPILGKAWCSYRIPLGSHAWLTPHTSVLGTTNRFNVINPAYTLLNERALQYQVG**

**IraqBI IPLTYIRYRNNGGYPLNGVVPPGTINMPILGKAWCSYRIPLGSHAWLTPHTSVLGTTNRFNVINPAYTLLNERALQYQVG**

**Fribourg-BlancF** **IPLTYIRYRNNGGYLLDGVVPPGTINMPILGKAWCSYRIPLGSHAWLTPHTSVLGTTNRFNVINPAYTLLNERALQYQVG**

**Fribourg-BlancI** **IPLTYIRYRNNGGYLLDGVVPPGTINMPILGKAWCSYRIPLGSHAWLTPHTSVLGTTNRFNVINPAYTLLNERALQYQVG**

570 580 590 600

....|....|....|....|....|....|....|....|....|....

**NicholsF**

**Bal3F**

**Sea81-4F**

**MexicoAF**

**Street14F**

**NicholsI**  **LTFSPFEKVELSAQWEQGVLADAPYMGIAESMWSERYFGTFICGVKVVW**

**Bal3I LTFSPFEKVELSAQWEQGVLADAPYMGIAESMWSERYFGTFICGVKVVW**

**Sea81-4I**  **LTFSPFEKVELSAQWEQGVLADAPYMGIAESMWSERYFGTFICGVKVVW**

**MexicoAI**  **LTFSPFEKVELSAQWEQGVLADAPYMGIAESMWSERYFGTFICGVKVVW**

**Street14I LTFSPFEKVELSAQWEQGVLADAPYMGIAESMWSERYFGTFICGVKVVW**

**GauthierF**  **LTFSPFEKVELSAQWEQGVLADAPYMGIAEGMWSERYFGTFICGVKVVW**

**GauthierI**  **LTFSPFEKVELSAQWEQGVLADAPYMGIAEGMWSERYFGTFICGVKVVW**

**SamoaDF**  **LTFSPFEKVELSAQWEQGVLADAPYMGIAESKWSERYFGTFICGVNVGW**

**SamoaDI LTFSPFEKVELSAQWEQGVLADAPYMGIAESMWSERYFGTFICGVKVVW**

**CDC2F**  **LTFSPFEKVELSAQWEQGVLADAPYMGIAESKWSERYFGTFICGVNVGW**

**CDC2I**  **LTFSPFEKVELSAQWEQGVLADAPYMGIAESMWSERYFGTFICGVKVVW**

**BosniaAI LTFSPFEKVELSAQWEQGVLSDVPYMGIAESMWSERYFGTFICGVKVVW**

**IraqBI LTFSPFEKVELSAQWEQGVLSDVPYMGIAESMWSERYFGTFICGVKVVW**

**Fribourg-BlancF** **LTFSPFXKVELSAQWEQGVLADAPYMGIAESMWSERYFGTFICGVKVVW**

**Fribourg-BlancI** **LTFSPFEKVELSAQWEQGVLADAPYMGIAESMWSERYFGTFICGVKVVW**

10 20 30 40 50 60 70 80

....|....|....|....|....|....|....|....|....|....|....|....|....|....|....|....|

**NicholsF**  **gtgggcaggcaggtgatgcaagcgggggtacttgcgggcatggtatgtgctgcttctggttatgcaggcgtactcactcc**

**Bal3F**  **gtgggcaggcaggtgatgcaagcgggggtacttgcgggcatggtatgtgctgcttctggttatgcaggcgtactcactcc**

**Sea81-4F**  **gtgggcaggcaggtgatgcaagcgggggtacttgcgggcatggtatgtgctgcttctggttatgcaggcgtactcactcc**

**MexicoAF**  **gtgggcaggcaggtgatgcaagcgggggtacttgcgggcatggtatgtgctgcttctggttatgcaggcgtactcactcc**

**Street14F gtgggcaggcaggtgatgcaagcgggggtacttgcgggcatggtatgtgctgcttctggttatgcaggcgtactcactcc**

**NicholsI**  **gtgggcaggcaggtgatgcaagcgggggtacttgcgggcatggtatgtgctgcttctggttatgcaggcgtactcactcc**

**Bal3I gtgggcaggcaggtgatgcaagcgggggtacttgcgggcatggtatgtgctgcttctggttatgcaggcgtactcactcc**

**Sea81-4I**  **gtgggcaggcaggtgatgcaagcgggggtacttgcgggcatggtatgtgctgcttctggttatgcaggcgtactcactcc**

**MexicoAI**  **gtgggcaggcaggtgatgcaagcgggggtacttgcgggcatggtatgtgctgcttctggttatgcaggcgtactcactcc**

**Street14I gtgggcaggcaggtgatgcaagcgggggtacttgcgggcatggtatgtgctgcttctggttatgcaggcgtactcactcc**

**GauthierF**  **gtgggcaggcaggtgatgcaagcgggggtacttgcgggcatggtatgtgctgcttctggttatgcaggcgtactcactcc**

**GauthierI**  **gtgggcaggcaggtgatgcaagcgggggtacttgcgggcatggtatgtgctgcttctggttatgcaggcgtactcactcc**

**SamoaDF**  **gtgggcaggcaggtgatgcaagcgggggtacttgcgggcatggtatgtgctgcttctggttatgcaggcgtactcactcc**

**SamoaDI gtgggcaggcaggtgatgcaagcgggggtacttgcgggcatggtatgtgctgcttctggttatgcaggcgtactcactcc**

**CDC2F**  **gtgggcaggcaggtgatgcaagcgggggtacttgcgggcatggtatgtgctgcttctggttatgcaggcgtactcactcc**

**CDC2I**  **gtgggcaggcaggtgatgcaagcgggggtacttgcgggcatggtatgtgctgcttctggttatgcaggcgtactcactcc**

**BosniaAI gtgggcaggcaggtgatgcaagcgggggtacttgcgggcatggtatgtgctgcttctggttatgcaggcgtactcactcc**

**IraqBI gtgggcaggcaggtgatgcaagcgggggtacttgcgggcatggtatgtgctgcttctggttatgcaggcgtactcactcc**

**Fribourg-BlancF** **gtgggcaggcaggtgatgcaagcgggggtacttgcgggcatggtatgtgctgcttctggttatgcaggcgtactcactcc**

**Fribourg-BlancI** **gtgggcaggcaggtgatgcaagcgggggtacttgcgggcatggtatgtgctgcttctggttatgcaggcgtactcactcc**

90 100 110 120 130 140 150 160

....|....|....|....|....|....|....|....|....|....|....|....|....|....|....|....|

**NicholsF**  **gcaggtcagtggcacagcccagctccagtggggcattgcgttccagaagaatccacgcactggcccgggcaagcacaccc**

**Bal3F**  **gcaggtcagtggcacagcccagctccagtggggcattgcgttccagaagaatccacgcactggcccgggcaagcacaccc**

**Sea81-4F**  **gcaggtcagtggcacagcccagctccagtggggcattgcgttccagaagaatccacgcactggcccgggcaagcacaccc**

**MexicoAF**  **gcaggtcagtggcacagcccagctccagtggggcattgcgttccagaagaatccacgcactggcccgggcaagcacaccc**

**Street14F gcaggtcagtggcacagcccagctccagtggggcattgcgttccagaagaatccacgcactggcccgggcaagcacaccc**

**NicholsI**  **gcaggtcagtggcacagcccagctccagtggggcattgcgttccagaagaatccacgcactggcccgggcaagcacaccc**

**Bal3I gcaggtcagtggcacagcccagctccagtggggcattgcgttccagaagaatccacgcactggcccgggcaagcacaccc**

**Sea81-4I**  **gcaggtcagtggcacagcccagctccagtggggcattgcgttccagaagaatccacgcactggcccgggcaagcacaccc**

**MexicoAI**  **gcaggtcagtggcacagcccagctccagtggggcattgcgttccagaagaatccacgcactggcccgggcaagcacaccc**

**Street14I gcaggtcagtggcacagcccagctccagtggggcattgcgttccagaagaatccacgcactggcccgggcaagcacaccc**

**GauthierF**  **gcaggtcagtggcacagcccagctccagtggggcattgcgttccagaagaatccacacactgtcccgggcgagcacaccc**

**GauthierI**  **gcaggtcagtggcacagcccagctccagtggggcattgcgttccagaagaatccacacactgtcccgggcgagcacaccc**

**SamoaDF**  **gcaggtcagtggcacagcccagctccagtggggcattgcgttccagaagaatccacacactgtcccgggcgagcacaccc**

**SamoaDI gcaggtcagtggcacagcccagctccagtggggcattgcgttccagaagaatccacacactgtcccgggcgagcacaccc**

**CDC2F**  **gcaggtcagtggcacagcccagctccagtggggcattgcgttccagaagaatccacacactgtcccgggcgagcacaccc**

**CDC2I**  **gcaggtcagtggcacagcccagctccagtggggcattgcgttccagaagaatccacacactgtcccgggcgagcacaccc**

**BosniaAI gcaggtcagtggcacagcccagctccagtggggcattgcgttccagaagaatccacgcactgtcccgggcgagcacaccc**

**IraqBI gcaggtcagtggcacagcccagctccagtggggcattgcgttccagaagaatccacgcactgtcccgggcgagcacaccc**

**Fribourg-BlancF** **gcaggtcagtggcacagcccagctccagtggggcattgcgttccagaagaatccacacactgtcccgggcgagcacaccc**

**Fribourg-BlancI** **gcaggtcagtggcacagcccagctccagtggggcattgcgttccagaagaatccacacactgtcccgggcgagcacaccc**

170 180 190 200 210 220 230 240

....|....|....|....|....|....|....|....|....|....|....|....|....|....|....|....|

**NicholsF**  **atgggtttcgcactaccaatagtctgactatttccctgccgttggtgtcaaagcacacccacacccgccgaggggaggca**

**Bal3F**  **atgggtttcgcactaccaatagtctgactatttccctgccgttggtgtcaaagcacacccacacccgccgaggggaggca**

**Sea81-4F**  **atgggtttcgcactaccaatagtctgactatttccctgccgttggtgtcaaagcacacccacacccgccgaggggaggca**

**MexicoAF**  **atgggtttcgcactaccaatagtctgactatttccctgccgttggtgtcaaagcacacccacacccgccgaggggaggca**

**Street14F atgggtttcgcactaccaatagtctgactatttccctgccgttggtgtcaaagcacacccacacccgccgaggggaggca**

**NicholsI**  **atgggtttcgcactaccaatagtctgactatttccctgccgttggtgtcaaagcacacccacacccgccgaggggaggca**

**Bal3I atgggtttcgcactaccaatagtctgactatttccctgccgttggtgtcaaagcacacccacacccgccgaggggaggca**

**Sea81-4I**  **atgggtttcgcactaccaatagtctgactatttccctgccgttggtgtcaaagcacacccacacccgccgaggggaggca**

**MexicoAI**  **atgggtttcgcactaccaatagtctgactatttccctgccgttggtgtcaaagcacacccacacccgccgaggggaggca**

**Street14I atgggtttcgcactaccaatagtctgactatttccctgccgttggtgtcaaagcacacccacacccgccgaggggaggca**

**GauthierF**  **atgggtttcgcactaccaatagtctgactatttccctgccgttggtgtcaaagcacacccacacccgccgaggggaggca**

**GauthierI**  **atgggtttcgcactaccaatagtctgactatttccctgccgttggtgtcaaagcacacccacacccgccgaggggaggca**

**SamoaDF**  **atgggtttcgcactaccaatagtctgactatttccctgccgttggtgtcaaagcacacccacacccgccgaggggaggca**

**SamoaDI atgggtttcgcactaccaatagtctgactatttccctgccgttggtgtcaaagcacacccacacccgccgaggggaggca**

**CDC2F**  **atgggtttcgcactaccaatagtctgactatttccctgccgttggtgtcaaagcacacccacacccgccgaggggaggca**

**CDC2I**  **atgggtttcgcactaccaatagtctgactatttccctgccgttggtgtcaaagcacacccacacccgccgaggggaggca**

**BosniaAI atgggtttcgcactaccaatagtctgactatttccctgccgttggtgtcaaagcacacccacacccgccgaggggaggca**

**IraqBI atgggtttcgcactaccaatagtctgactatttccctgccgttggtgtcaaagcacacccacacccgccgaggggaggca**

**Fribourg-BlancF** **atgggtttcgcactaccaatagtctgactatttccctgccgttggtgtcaaagcacacccacacccgccgaggggaggca**

**Fribourg-BlancI** **atgggtttcgcactaccaatagtctgactatttccctgccgttggtgtcaaagcacacccacacccgccgaggggaggca**

250 260 270 280 290 300 310 320

....|....|....|....|....|....|....|....|....|....|....|....|....|....|....|....|

**NicholsF**  **cgctcaggggtgtgggcacagctgcagctgaaggacctggcagtagagcttgcgtcttctaaaagctcaacggccctgtc**

**Bal3F**  **cgctcaggggtgtgggcacagctgcagctgaaggacctggcagtagagcttgcgtcttctaaaagctcaacggccctgtc**

**Sea81-4F**  **cgctcaggggtgtgggcacagctgcagctgaaggacctggcagtagagcttgcgtcttctaaaagctcaacggccctgtc**

**MexicoAF**  **cgctcaggggtgtgggcacagctgcagctgaaggacctggcagtagagcttgcgtcttctaaaagctcaacggccctgtc**

**Street14F cgctcaggggtgtgggcacagctgcagctgaaggacctggcagtagagcttgcgtcttctaaaagctcaacggccctgtc**

**NicholsI**  **cgctcaggggtgtgggcacagctgcagctgaaggacctggcagtagagcttgcgtcttctaaaagctcaacggccctgtc**

**Bal3I cgctcaggggtgtgggcacagctgcagctgaaggacctggcagtagagcttgcgtcttctaaaagctcaacggccctgtc**

**Sea81-4I**  **cgctcaggggtgtgggcacagctgcagctgaaggacctggcagtagagcttgcgtcttctaaaagctcaacggccctgtc**

**MexicoAI**  **cgctcaggggtgtgggcacagctgcagctgaaggacctggcagtagagcttgcgtcttctaaaagctcaacggccctgtc**

**Street14I cgctcaggggtgtgggcacagctgcagctgaaggacctggcagtagagcttgcgtcttctaaaagctcaacggccctgtc**

**GauthierF**  **cgcttgggggtgtgggcacagctgcagctgaaggacctggcagtagagcttgcgtcttctaaaagctcaacggccctgtc**

**GauthierI**  **cgcttgggggtgtgggcacagctgcagctgaaggacctggcagtagagcttgcgtcttctaaaagctcaacggccctgtc**

**SamoaDF**  **cgctcaggggtgtgggcacagctgcagctgaaggacctggcagtagagcttgcgtcttctaaaagctcaacggccctgtc**

**SamoaDI cgctcaggggtgtgggcacagctgcagctgaaggacctggcagtagagcttgcgtcttctaaaagctcaacggccctgtc**

**CDC2F**  **cgctcaggggtgtgggcacagctgcagctgaaggacctggcagtagagcttgcgtcttctaaaagctcaacggccctgtc**

**CDC2I**  **cgctcaggggtgtgggcacagctgcagctgaaggacctggcagtagagcttgcgtcttctaaaagctcaacggccctgtc**

**BosniaAI cgctcaggggtgtgggcacagctgcagctgaaggacctggcagtagagcttgcgtcttctaaaagctcaacggccctgtc**

**IraqBI cgctcaggggtgtgggcacagctgcagctgaaggacctggcagtagagcttgcgtcttctaaaagctcaacggccctgtc**

**Fribourg-BlancF** **cgctcaggggtgtgggcacagctgcagctgaaggacctggcagtagagcttgcgtcttctaaaagctcaacggccctgtc**

**Fribourg-BlancI** **cgctcaggggtgtgggcacagctgcagctgaaggacctggcagtagagcttgcgtcttctaaaagctcaacggccctgtc**

330 340 350 360 370 380 390 400

....|....|....|....|....|....|....|....|....|....|....|....|....|....|....|....|

**NicholsF**  **ctttaccaaacctaccgcttccttccaggcaaccctgcactgttatggggcctacctgacagtgggtaccagtccttcct**

**Bal3F**  **ctttaccaaacctaccgcttccttccaggcaaccctgcactgttatggggcctacctgacagtgggtaccagtccttcct**

**Sea81-4F**  **ctttaccaaacctaccgcttccttccaggcaaccctgcactgttatggggcctacctgacagtgggtaccagtccttcct**

**MexicoAF**  **ctttaccaaacctaccgcttccttccaggcaaccctgcactgttatggggcctacctgacagtgggtaccagtccttcct**

**Street14F ctttaccaaacctaccgcttccttccaggcaaccctgcactgttatggggcctacctgacagtgggtaccagtccttcct**

**NicholsI**  **ctttaccaaacytaccgcttccttccaggcaaccctgcactgttatggggcctacctgacagtgggtaccagtccttcct**

**Bal3I ctttaccaaacctaccgcttccttccaggcaaccctgcactgttatggggcctacctgacagtgggtaccagtccttcct**

**Sea81-4I**  **ctttaccaaacctaccgcttccttccaggcaaccctgcactgttatggggcctacctgacagtgggtaccagtccttcct**

**MexicoAI**  **ctttaccaaacctaccgcttccttccaggcaaccctgcactgttatggggcctacctgacagtgggtaccagtccttcct**

**Street14I ctttaccaaacytaccgcttccttccaggcaaccctgcactgttatggggcctacctgacagtgggtaccagtccttcct**

**GauthierF**  **ctttaccaaacctaccgcttccttccaggcaaccctgcactgttatggggcctacctgacagtgggtaccagtccttcct**

**GauthierI**  **ctttaccaaacctaccgcttccttccaggcaaccctgcactgttatggggcctacctgacagtgggtaccagtccttcct**

**SamoaDF**  **ctttaccaaacctaccgcttccttccaggcaaccctgcactgttatggggcctacctgacagtgggtaccagtccttcct**

**SamoaDI ctttaccaaacctaccgcttccttccaggcaaccctgcactgttatggggcctacctgacagtgggtaccagtccttcct**

**CDC2F**  **ctttaccaaacctaccgcttccttccaggcaaccctgcactgttatggggcctacctgacagtgggtaccagtccttcct**

**CDC2I**  **ctttaccaaacctaccgcttccttccaggcaaccctgcactgttatggggcctacctgacagtgggtaccagtccttcct**

**BosniaAI ctttaccaaacctaccgcttccttccaggcaaccctgcactgttatggggcctacctgacagtgggtaccagtccttcct**

**IraqBI ctttaccaaacctaccgcttccttccaggcaaccctgcactgttatggggcctacctgacagtgggtaccagtccttcct**

**Fribourg-BlancF** **ctttaccaaacctaccgcttccttccaggcaaccctgcactgttatggggcctacctgacagtgggtaccagtccttcct**

**Fribourg-BlancI** **ctttaccaaacctaccgcttccttccaggcaaccctgcactgttatggggcctacctgacagtgggtaccagtccttcct**

410 420 430 440 450 460 470 480

....|....|....|....|....|....|....|....|....|....|....|....|....|....|....|....|

**NicholsF**  **gtgtggttaactttgcccagctgtggaaaccctttgtcacccgtgcctattcagaaaaggacactcgctatgcccctggt**

**Bal3F**  **gtgtggttaactttgcccagctgtggaaaccctttgtcacccgtgcctattcagaaaaggacactcgctatgcccctggt**

**Sea81-4F**  **gtgtggttaactttgcccagctgtggaaaccctttgtcacccgtgcctattcagaaaaggacactcgctatgcccctggt**

**MexicoAF**  **gtgtggttaactttgcccagctgtggaaaccctttgtcacccgtgcctattcagaaaaggacactcgctatgcccctggt**

**Street14F gtgtggttaactttgcccagctgtggaaaccctttgtcacccgtgcctattcagaaaaggacactcgctatgcccctggt**

**NicholsI**  **gtgtggttaactttgcccagctgtggaaaccctttgtcacccgtgcctattcagaaaaggacactcgctatgcccctggt**

**Bal3I gtgtggttaactttgcccagctgtggaaaccctttgtcacccgtgcctattcagaaaaggacactcgctatgcccctggt**

**Sea81-4I**  **gtgtggttaactttgcccagctgtggaaaccctttgtcacccgtgcctattcagaaaaggacactcgctatgcccctggt**

**MexicoAI**  **gtgtggttaactttgcccagctgtggaaaccctttgtcacccgtgcctattcagaaaaggacactcgctatgcccctggt**

**Street14I gtgtggttaactttgcccagctgtggaaaccctttgtcacccgtgcctattcagaaaaggacactcgctatgcccctggt**

**GauthierF**  **gtgtggttaactttgcccagctgtggaaaccctttgtcacccgtgcctattcagaaaaggacactcgctatgcccctggt**

**GauthierI**  **gtgtggttaactttgcccagctgtggaaaccctttgtcacccgtgcctattcagaaaaggacactcgctatgcccctggt**

**SamoaDF**  **gtgtggttaactttgcccagctgtggaaaccctttgtcacccgtgcctattcagaaaaggacactcgctatgcccctggt**

**SamoaDI gtgtggttaactttgcccagctgtggaaaccctttgtcacccgtgcctattcagaaaaggacactcgctatgcccctggt**

**CDC2F**  **gtgtggttaactttgcccagctgtggaaaccctttgtcacccgtgcctattcagaaaaggacactcgctatgcccctggt**

**CDC2I**  **gtgtggttaactttgcccagctgtggaaaccctttgtcacccgtgcctattcagaaaaggacactcgctatgcccctggt**

**BosniaAI gtgtggttaactttgcccagctgtggaaaccctttgtcacccgtgcctattcagaaaaggacactcgctatgcccctggt**

**IraqBI gtgtggttaactttgcccagctgtggaaaccctttgtcacccgtgcctattcagaaaaggacactcgctatgcccctggt**

**Fribourg-BlancF** **gtgtggttaactttgcccagctgtggaaaccctttgtcacccgtgcctattcagaaaaggacactcgctatgcccctggt**

**Fribourg-BlancI** **gtgtggttaactttgcccagctgtggaaaccctttgtcacccgtgcctattcagaaaaggacactcgctatgcccctggt**

490 500 510 520 530 540 550 560

....|....|....|....|....|....|....|....|....|....|....|....|....|....|....|....|

**NicholsF**  **ttctccggctccggggcaaaactcggctaccaggcccacaatgtgggaaacagcggagtagatgtggacatcggtttcct**

**Bal3F**  **ttctccggctccggggcaaaactcggctaccaggcccacaatgtgggaaacagcggagtagatgtggacatcggtttcct**

**Sea81-4F**  **ttctccggctccggggcaaaactcggctaccaggcccacaatgtgggaaacagcggagtagatgtggacatcggtttcct**

**MexicoAF**  **ttctccggctccggggcaaaactcggctaccaggcccacaatgtgggaaacagcggagtagatgtggacatcggtttcct**

**Street14F ttctccggctccggggcaaaactcggctaccaggcccacaatgtgggaaacagcggagtagatgtggacatcggtttcct**

**NicholsI**  **ttctccggytccggggcaaaactcggctaccaggcccacaatgtgggaaacagcggagtagatgtggacatcggtttcct**

**Bal3I ttctccggctccggggcaaaactcggctaccaggcccacaatgtgggaaacagcggagtagatgtggacatcggtttcct**

**Sea81-4I**  **ttctccggctccggggcaaaactcggctaccaggcccacaatgtgggaaacagcggagtagatgtggacatcggtttcct**

**MexicoAI**  **ttctccggctccggggcaaaactcggctaccaggcccacaatgtgggaaacagcggagtagatgtggacatcggtttcct**

**Street14I ttctccggytccggggcaaaactcggctaccaggcccacaatgtgggaaacagcggagtagatgtggacatcggtttcct**

**GauthierF**  **ttctccggctccggggcaaaactcggctaccaggcccacaatgtgggaaacagcggagtagatgtggacatcggtttcct**

**GauthierI**  **ttctccggctccggggcaaaactcggctaccaggcccacaatgtgggaaacagcggagtagatgtggacatcggtttcct**

**SamoaDF**  **ttctccggctccggggcaaaactcggctaccaggcccacaatgtgggaaacagcggagtagatgtggacatcggtttcct**

**SamoaDI ttctccggctccggggcaaaactcggctaccaggcccacaatgtgggaaacagcggagtagatgtggacaccggtttcct**

**CDC2F**  **ttctccggctccggggcaaaactcggctaccaggcccacaatgtgggaaacagcggagtagatgtggacatcggtttcct**

**CDC2I**  **ttctccggctccggggcaaaactcggctaccaggcccacaatgtgggaaacagcggaatagatgtggacatcggtttcct**

**BosniaAI ttctccggctccggggcaaaactcggctaccaggcccacaatgtgggaaacagcggagtagatgtggacatcggtttcct**

**IraqBI ttctccggctccggggcaaaactcggctaccaggcccacaatgtgggaaacagcggagtagatgtggacatcggtttcct**

**Fribourg-BlancF** **ttctccggctccggggcaaaactcggctaccaggcccacaatgtgggaaacagcggagtagatgtggacatcggtttcct**

**Fribourg-BlancI** **ttctccggctccggggcaaaactcggctaccaggcccacaatgtgggaaacagcggagtagatgtggacaccggtttcct**

570 580 590 600 610 620 630 640

....|....|....|....|....|....|....|....|....|....|....|....|....|....|....|....|

**NicholsF**  **ctccttcctttccaatggtgcctgggatagtactgacaccacgcacagcaagtatggcttcggggccgatgcaacgcttt**

**Bal3F**  **ctccttcctttccaatggtgcctgggatagtactgacaccacgcacagcaagtatggcttcggggccgatgcaacgcttt**

**Sea81-4F**  **ctccttcctttccaatggtgcctgggatagtactgacaccacgcacagcaagtatggcttcggggccgatgcaacgcttt**

**MexicoAF**  **ctccttcctttccaatggtgcctgggatagtactgacaccacgcacagcaagtatggcttcggggccgatgcaacgcttt**

**Street14F ctccttcctttccaatggtgcctgggatagtactgacaccacgcacagcaagtatggcttcggggccgatgcaacgcttt**

**NicholsI**  **ctccttcctttccaatggtgcctgggatagtactgacaccacgcacagcaagtatggcttcggggccgatgcaacgcttt**

**Bal3I ctccttcctttccaatggtgcctgggatagtactgacaccacgcacagcaagtatggcttcggggccgatgcaacgcttt**

**Sea81-4I**  **ctccttcctttccaatggtgcctgggatagtactgacaccacgcacagcaagtatggcttcggggccgatgcaacgcttt**

**MexicoAI**  **ctccttcctttccaatggtgcctgggatagtactgacaccacgcacagcaagtatggcttcggggccgatgcaacgcttt**

**Street14I ctccttcctttccaatggtgcctgggatagtactgacaccacgcacagcaagtatggcttcggggccgatgcaacgcttt**

**GauthierF**  **ctccttcctttccaatggtgcctgggatagtactgacaccacgcacagcaagtatggcttcggggccgatgcaacgcttt**

**GauthierI**  **ctccttcctttccaatggtgcctgggatagtactgacaccacgcacagcaagtatggcttcggggccgatgcaacgcttt**

**SamoaDF**  **ctccttcctttccaatggtgcctgggatagtactgacaccacgcacagcaagtatggcttcggggccgatgcaacgcttt**

**SamoaDI ctccttcctttccaatggtgcctgggatagtactgaccccacgcacagtaagtatggctttggggccgatgcaacgcttt**

**CDC2F**  **ctccttcctttccaatggtgcctgggatagtactgacaccacgcacagcaagtatggcttcggggccgatgcaacgcttt**

**CDC2I**  **ctccttcctttccaatggtgcctgggatagtactgacaccacgcacagcaagtatggcttcggggccgatgcaacgcttt**

**BosniaAI ctccttcctttccaatggtgcctgggatagtactgacaccatgcacagcaagtatggcttcggggccgatgcaacgcttt**

**IraqBI ctccttcctttccaatggtgcctgggatagtactgacaccatgcacagcaagtatggcttcggggccgatgcaacgcttt**

**Fribourg-BlancF** **ctccttcctttccaatggtgcctgggatagtactgacgccacgcacagcaagtatggcttcggggccgatgcaacgcttt**

**Fribourg-BlancI** **ctccttcctttccaatggtgcctgggatagtactgaccccacgcacagcaagtatggcttcggggccgatgcaacgcttt**

650 660 670 680 690 700 710 720

....|....|....|....|....|....|....|....|....|....|....|....|....|....|....|....|

**NicholsF**  **cctatggcgtcgaccgtcagcggctgcttacgttggagctggcagggaatgccacactggagcagcactaccgtaagggt**

**Bal3F**  **cctatggcgtcgaccgtcagcggctgcttacgttggagctggcagggaatgccacactggagcagcactaccgtaagggt**

**Sea81-4F**  **cctatggcgtcgaccgtcagcggctgcttacgttggagctggcagggaatgccacactggagcagcactaccgtaagggt**

**MexicoAF**  **cctatggcgtcgaccgtcagcggctgcttacgttggagctggcagggaatgccacactggagcagcactaccgtaagggt**

**Street14F cctatggcgtcgaccgtcagcggctgcttacgttggagctggcagggaatgccacactggagcagcactaccgtaagggt**

**NicholsI**  **cctatggcgtcgaccgtcagcggctgcttacgttggagctggcagggaatgccacactggagcagcactaccgtaagggt**

**Bal3I cctatggcgtcgaccgtcagcggctgcttacgttggagctggcagggaatgccacactggagcagcactaccgtaagggt**

**Sea81-4I**  **cctatggcgtcgaccgtcagcggctgcttacgttggagctggcagggaatgccacactggagcagcactaccgtaagggt**

**MexicoAI**  **cctatggcgtcgaccgtcagcggctgcttacgttggagctggcagggaatgccacactggagcagcactaccgtaagggt**

**Street14I cctatggcgtcgaccgtcagcggctgcttacgttggagctggcagggaatgccacactggaccagaactacgttaagggt**

**GauthierF**  **cctatggcgtcgaccgtcagcggctgcttacgttggagctggcagggaatgccacactggagcagcactaccgtaagggt**

**GauthierI**  **cctatggcgtcgaccgtcagcggctgcttacgttggagctggcagggaatgccacactggagcagcactaccgtaagggt**

**SamoaDF**  **cctatggcgtcgaccgtcagcggctgcttacgttggagctggcagggaatgccacactggagcagcactaccgtaagggt**

**SamoaDI cctatggcgtcgaccgtcagcggctgcttacgttggagctggcagggaatgccacactggagcagcactaccgtaagggt**

**CDC2F**  **cctatggcgtcgaccgtcagcggctgcttacgttggagctggcagggaatgccacactggagcagcactaccgtaagggt**

**CDC2I**  **cctatggcgtcgaccgtcagcggctgcttacgttggagctggcagggaatgccacactggagcagcactaccgtaagggt**

**BosniaAI cctatggcgtcgaccgtcagcggctgcttacgttggagctggcagggaatgccacactggagcagcactaccgtaagggt**

**IraqBI cctatggcgtcgaccgtcagcggctgcttacgttggagctggcagggaatgccacactggagcagcactaccgtaagggt**

**Fribourg-BlancF** **cctatggcgtcgaccgtcagcggctgcttacgttggagctggcagggaatgccacactggagcagcactaccgtaagggt**

**Fribourg-BlancI** **cctatggcgtcgaccgtcagcggctgcttacgttggagctggcagggaatgccacactggagcagcactaccgtaagggt**

730 740 750 760 770 780 790 800

....|....|....|....|....|....|....|....|....|....|....|....|....|....|....|....|

**NicholsF**  **accgaagactccacgaacgaaaacaaaacagcactcctgtggggagtaggaggccgactcaccctcgaaccaggcgccgg**

**Bal3F**  **accgaagactccacgaacgaaaacaaaacagcactcctgtggggagtaggaggccgactcaccctcgaaccaggcgccgg**

**Sea81-4F**  **accgaagactccacgaacgaaaacaaaacagcactcctgtggggagtaggaggccgactcaccctcgaaccaggcgccgg**

**MexicoAF**  **accgaagactccacgaacgaaaacaaaacagcactcctgtggggagtaggaggccgactcaccctcgaaccaggcgccgg**

**Street14F accgaagactccacgaacgaaaacaaaacagcactcctgtggggagtaggaggccgactcaccctcgaaccaggcgccgg**

**NicholsI**  **accgaagactccacgaacgaaaacaaaacagcactcctgtggggagtaggaggccgactcaccctcgaaccaggcgccgg**

**Bal3I accgaagactccacgaacgaaaacaaaacagcactcctgtggggagtaggaggccgactcaccctcgaaccaggcgccgg**

**Sea81-4I**  **accgaagactccacgaacgaaaacaaaacagcactcctgtggggagtaggaggccgactcaccctcgaaccaggcgccgg**

**MexicoAI**  **accgaagactccacgaacgaaaacaaaacagcactcctgtggggagtaggaggccgactcaccctcgaaccaggcgccgg**

**Street14I accgaagactccacgaacgaaaacaaaacagcactcctgtggggagtaggaggccgactcaccctcgaaccaggcgccgg**

**GauthierF**  **accgaagactccccgaacgaaaacaaaacagcactcccgtggggagtaggaggccgactcaccctcgaaccaggcgccgg**

**GauthierI**  **accgaagactccccgaacgaaaacaaaacagcactcccgtggggagtaggaggccgactcaccctcgaaccaggcgccgg**

**SamoaDF**  **accgaagactccacgaacgaaaacaaaacagcactcctgtggggagtaggaggccgactcaccctcgaaccaggcgccgg**

**SamoaDI accgaagactccacgaacgaaaacaaaacagcactcctgtggggagtaggaggccgactcaccctcgaaccaggcgccgg**

**CDC2F**  **accgaagactccacgaacgaaaacaaaacagcactcctgtggggagtaggaggccgactcaccctcgaaccaggcgccgg**

**CDC2I**  **accgaagactccacgaacgaaaacaaaacagcactcctgtggggagtaggaggccgactcaccctcgaaccaggcgccgg**

**BosniaAI accgaagactccacgaacgaaaacaaaacagcactcctgtggggagtaggaggccgactcaccctcgaaccaggcgccgg**

**IraqBI accgaagactccacgaacgaaaacaaaacagcactcctgtggggagtaggaggccgactcaccctcgaaccaggcgccgg**

**Fribourg-BlancF** **accgaagactccacgaacgaaaacaaaacagcactcctgtggggagtaggaggccgactcaccctcgaaccaggcgccgg**

**Fribourg-BlancI** **accgaagactccacgaacgaaaacaaaacagcactcctgtggggagtaggaggccgactcaccctcgaaccaggcgccgg**

810 820 830 840 850 860 870 880

....|....|....|....|....|....|....|....|....|....|....|....|....|....|....|....|

**NicholsF**  **cttccgcttctccttcgccctcgacgccggtaaccaacaccaggaccctgccgatgcaggtaatcgccttctggcaacgg**

**Bal3F**  **cttccgcttctccttcgccctcgacgccggtaaccaacaccaggaccctgccgatgcaggtaatcgccttctggcaacgg**

**Sea81-4F**  **cttccgcttctccttcgccctcgacgccggtaaccaacaccaggaccctgccgatgcaggtaatcgccttctggcaacgg**

**MexicoAF**  **cttccgcttctccttcgccctcgacgccggtaaccaacaccaggaccctgccgatgcaggtaatcgccttctggcaacgg**

**Street14F cttccgcttctccttcgccctcgacgccggtaaccaacaccaggaccctgccgatgcaggtaatcgccttctggcaacgg**

**NicholsI**  **cttccgcttctccttcgccctcgacgccggtaaccaacaccaggaccctgccgatgcaggtaatcgccttctggcaacgg**

**Bal3I cttccgcttctccttcgccctcgacgccggtaaccaacaccaggaccctgccgatgcaggtaatcgccttctggcaacgg**

**Sea81-4I**  **cttccgcttctccttcgccctcgacgccggtaaccaacaccaggaccctgccgatgcaggtaatcgccttctggcaacgg**

**MexicoAI**  **cttccgcttctccttcgccctcgacgccggtaaccaacaccaggaccctgccgatgcaggtaatcgccttctggcaacgg**

**Street14I cttccgcttctccttcgccctcgacgccggtaaccaacaccaggaccctgccgatgcaggtaatcgccttctggcaacgg**

**GauthierF**  **cttccgcttctccttcgccctcgacgccggtaaccaacaccagagtaacgcagatgcagactgtcgccttccggcaacgg**

**GauthierI**  **cttccgcttctccttcgccctcgacgccggtaaccaacaccagagtaacgcagatgcagactgtcgccttccggcaacgg**

**SamoaDF**  **cttccgcttctccttcgccctcgacgccggtaaccaacaccagagtaacgcagatgcagactgtcgccttccggcaacgg**

**SamoaDI cttccgcttctccttcgccctcgacgccggtaaccaacaccagagtaacgcagatgcagactgtcgccttccggcaacgg**

**CDC2F**  **cttccgcttctccttcgccctcgacgccggtaaccaacaccagagtaacgcagatgcagactgtcgccttccggcaacgg**

**CDC2I**  **cttccgcttctccttcgccctcgacgccggtaaccaacaccagagtaacgcagatgcagactgtcgccttccggcaacgg**

**BosniaAI cttccgcttctccttcgccctcgacgccggtaaccaacaccagagtaacgcagatgcagactgtcgccttccggcaacgg**

**IraqBI cttccgcttctccttcgccctcgacgccggtaaccaacaccagagtaacgcagatgcagactgtcgccttccggcaacgg**

**Fribourg-BlancF** **cttccgcttctccttcgccctcgacgccggtaaccaacaccagagtgacgcagatgcagactgtcgccttccggcaacgg**

**Fribourg-BlancI** **cttccgcttctccttcgccctcgacgccggtaaccaacaccagagtgacgcagatgcagactgtcgccttccggcaacgg**

890 900 910 920 930 940 950 960

....|....|....|....|....|....|....|....|....|....|....|....|....|....|....|....|

**NicholsF**  **ggagctcacgggagaagtttgacagcgcgttcgatgccctcagggtggagcaataccgtgtaaaggataagtatcttgaa**

**Bal3F**  **ggagctcacgggagaagtttgacagcgcgttcgatgccctcagggtggagcaataccgtgtaaaggataagtatcttgaa**

**Sea81-4F**  **ggagctcacgggagaagtttgacagcgcgttcgatgccctcagggtggagcaataccgtgtaaaggataagtatcttgaa**

**MexicoAF**  **ggagctcacgggagaagtttgacagcgcgttcgatgccctcagggtggagcaataccgtgtaaaggataagtatcttgaa**

**Street14F ggagctcacgggagaagtttgacagcgcgttcgatgccctcagggtggagcaataccgtgtaaaggataagtatcttgaa**

**NicholsI**  **ggagctcacgggagaagtttgacagcgcgttcgatgccctcagggtggagcaataccgtgtaaaggataagtatcttgaa**

**Bal3I ggagctcacgggagaagtttgacagcgcgttcgatgccctcagggtggagcaataccgtgtaaaggataagtatcttgaa**

**Sea81-4I**  **ggagctcacgggagaagtttgacagcgcgttcgatgccctcagggtggagcaataccgtgtaaaggataagtatcttgaa**

**MexicoAI**  **ggagctcacgggagaagtttgacagcgcgttcgatgccctcagggtggagcaataccgtgtaaaggataagtatcttgaa**

**Street14I ggagctcacgggagaagtttgacagcgcgttcgatgccctcagggtggagcaataccgtgtaaaggataagtatcttgaa**

**GauthierF**  **ggaactcacgggagaagtttgacagggcgttcgatgccctcagggtggagcaataccgtgtaaaggataagtatcttgaa**

**GauthierI**  **ggaactcacgggagaagtttgacagggcgttcgatgccctcagggtggagcaataccgtgtaaaggataagtatcttgaa**

**SamoaDF**  **ggagctcacgggagaagtttgacagggcgttcgatgccctcagggtggagcaataccgtgtaaaggataagtatcttgaa**

**SamoaDI ggagctcacgggagaagtttgacagggcgttcgatgccctcagggtggagcaataccgtgtaaaggataagtatcttgaa**

**CDC2F**  **ggagctcacgggagaagtttgacagggcgttcgatgccctcagggtggagcaataccgtgtaaaggataagtatcttgaa**

**CDC2I**  **ggagctcacgggagaagtttgacagggcgttcgatgccctcagggtggagcaataccgtgtaaaggataagtatcttgaa**

**BosniaAI ggagctcacgggagaagtttgacagggcgttcgatgccctcagggtggagcaataccgtgtaaaggataagtatcttgaa**

**IraqBI ggagctcacgggagaagtttgacagggcgttcgatgccctcagggtggagcaataccgtgtaaaggataagtatcttgaa**

**Fribourg-BlancF** **ggagctcacgggagaagtttgacagggcgttcgatgccctcagggtggagcaatactatgtaaaggataagtatcttgaa**

**Fribourg-BlancI** **ggagctcacgggagaagtttgacagggcgttcgatgccctcagggtggagcaatactatgtaaaggataagtatcttgaa**

970 980 990 1000 1010 1020 1030 1040

....|....|....|....|....|....|....|....|....|....|....|....|....|....|....|....|

**NicholsF**  **tttttgctgggacagatggcggagtcctcgattctcgagcgggtggggcttgccctcacgctgcaggacggtacgctcgt**

**Bal3F**  **tttttgctgggacagatggcggagtcctcgattctcgagcgggtggggcttgccctcacgctgcaggacggtacgctcgt**

**Sea81-4F**  **tttttgctgggacagatggcggagtcctcgattctcgagcgggtggggcttgccctcacgctgcaggacggtacgctcgt**

**MexicoAF**  **tttttgctgggacagatggcggagtcctcgattctcgagcgggtggggcttgccctcacgctgcaggacggtacgctcgt**

**Street14F tttttgctgggacagatggcggagtcctcgattctcgagcgggtggggcttgccctcacgctgcaggacggtacgctcgt**

**NicholsI**  **tttttgctgggacagatggcggagtcctcgattctcgagcgggtggggcttgccctcacgctgcaggacggtacgctcgt**

**Bal3I tttttgctgggacagatggcggagtcctcgattctcgagcgggtggggcttgccctcacgctgcaggacggtacgctcgt**

**Sea81-4I**  **tttttgctgggacagatggcggagtcctcgattctcgagcgggtggggcttgccctcacgctgcaggacggtacgctcgt**

**MexicoAI**  **tttttgctgggacagatggcggagtcctcgattctcgagcgggtggggcttgccctcacgctgcaggacggtacgctcgt**

**Street14I tttttgctgggacagatggcggagtcctcgattctcgagcgggtggggcttgccctcacgctgcaggacggtacgctcgt**

**GauthierF**  **tttttgctgggacagacggcggagtcctcgattctcgagcgggtggggcttgccctcacgctgcaggacggtacgctcgt**

**GauthierI**  **tttttgctgggacagacggcggagtcctcgattctcgagcgggtggggcttgccctcacgctgcaggacggtacgctcgt**

**SamoaDF**  **tttttgctgggacagatggcggagtcctcgattctcgagcgggtggggcttgccctcacgctgcaggacggtacgctcgt**

**SamoaDI tttttgctgggacagatggcggagtcctcgattctcgagcgggtggggcttgccctcacgctgcaggacggtacgctcgt**

**CDC2F**  **tttttgctgggacagatggcggagtcctcgattctcgagcgggtggggcttgccctcacgctgcaggacggtacgctcgt**

**CDC2I**  **tttttgctgggacagatggcggagtcctcgattctcgagcgggtggggcttgccctcacgctgcaggacggtacgctcgt**

**BosniaAI tttttgctgggacagatggcggagtcctcgattctcgagcgggtggggcttgccctcacgctgcaggacggtacgctcgt**

**IraqBI tttttgctgggacagatggcggagtcctcgattctcgagcgggtggggcttgccctcacgctgcaggacggtacgctcgt**

**Fribourg-BlancF** **tttttgctgggacagatggcggagtcctcgattctcgagcgggtggggcttgccctcacgctgcaggacggtacgctcgt**

**Fribourg-BlancI** **tttttgctgggacagatggcggagtcctcgattctcgagcgggtggggcttgccctcacgctgcaggacggtacgctcgt**

1050 1060 1070 1080 1090 1100 1110 1120

....|....|....|....|....|....|....|....|....|....|....|....|....|....|....|....|

**NicholsF**  **ctctacgctgacgaaggttgccactgatagtggagctcag----------------------------------------**

**Bal3F**  **ctctacgctgacgaaggttgccactgatagtggagctcag----------------------------------------**

**Sea81-4F**  **ctctacgctgacgaaggttgccactgatagtggagctcag----------------------------------------**

**MexicoAF**  **ctctacgctgacgaaggttgccactgatagtggagctcag----------------------------------------**

**Street14F ctctacgctgacgaaggttgccactgatagtggagctcag----------------------------------------**

**NicholsI**  **ctctacgctgacgaaggttgccactgatagtggagatcggtttatccaaatggcgttggtaaaactcttgccccagaggg**

**Bal3I ctctacgctgacgaaggttgccactgatagtggagatcggtttatccaaatggcgttggtaaaactcttgccccagaggg**

**Sea81-4I**  **ctctacgctgacgaaggttgccactgatagtggagatcggtttatccaaatggcgttggtaaaactcttgccccagaggg**

**MexicoAI**  **ctctacgctgacgaaggttgccactgatagtggagatcggtttatccaaatggcgttggtaaaactcttgccccagaggg**

**Street14I ctctacgctgacgaaggttgccactgatagtggagatcggtttatccaaatggcgttggtaaaactcttgccccagaggg**

**GauthierF**  **ctctacgctgacgaaggttgccactgatagtggagatcggtttatccaaatggcgttggtaaaactcttgccccagaggg**

**GauthierI**  **ctctacgctgacgaaggttgccactgatagtggagatcggtttatccaaatggcgttggtaaaactcttgccccagaggg**

**SamoaDF**  **ctctacgctgacgaaggttgccactgatagtggagattggtttatccaaatggcgttggtaaaactcttgccccagaggg**

**SamoaDI**  **ctctacgctgacgaaggttgccactgatagtggagatcggtttatccaaatggcgttggtaaaactcttgccccagaggg**

**CDC2F**  **ctctacgctgacgaaggttgccactgatagtggagattggtttatccaaatggcgttggtaaaactcttgccccagaggg**

**CDC2I**  **ctctacgctgacgaaggttgccactgatagtggagattggtttatccaaatggcgttggtaaaactcttgccccagaggg**

**BosniaAI**  **ctctacgctgacgaaggttgccactgatagtggagatcggtttatccaaatggcgttggtaaaactcttgccccagaggg**

**IraqBI**  **ctctacgctgacgaaggttgccactgatagtggagatcggtttatccaaatggcgttggtaaaactcttgccccagaggg**

**Fribourg-BlancF** **ctctacgctgacgaaggttgccactgatagtggagatcggtttatccaaatggcgttggtaaaactcttgccccagaggg**

**Fribourg-BlancI** **ctctacgctgacgaaggttgccactgatagtggagatcagtttatccaaatggcgttggtaaaactcttgccccagaggg**

1130 1140 1150 1160 1170 1180 1190 1200

....|....|....|....|....|....|....|....|....|....|....|....|....|....|....|....|

**NicholsF**  **--------------------------------------------------------------------------------**

**Bal3F**  **--------------------------------------------------------------------------------**

**Sea81-4F**  **--------------------------------------------------------------------------------**

**MexicoAF**  **--------------------------------------------------------------------------------**

**Street14F --------------------------------------------------------------------------------**

**NicholsI**  **cgcaggcggagcagagactacaggagattgtggcgccgagtcagtcggacatcgtgcttatcatgctgctaacctggctt**

**Bal3I cgcaggcggagcagagactacaggagattgtggcgccgagtcagtcggacatcgtgcttatcatgctgctaacctggctt**

**Sea81-4I**  **cgcaggcggagcagagactacaggagattgtggcgccgagtcagtcggacatcgtgcttatcatgctgctaacctggctt**

**MexicoAI**  **cgcaggcggagcagagactacaggagattgtggcgccgagtcagtcggacatcgtgcttatcatgctgctaacctggctt**

**Street14I cgcaggcggagcagagactacaggagattgtggcgccgagtcagtcggacatcgtgcttatcatgctgctaacctggctt**

**GauthierF**  **cgcaagctgagcagggcctacgggagattgtggcgccgagtcagtcggacatcgtgcttatcatgctgctaacctggctt**

**GauthierI**  **cgcaagctgagcagggcctacgggagattgtggcgccgagtcagtcggacatcgtgcttatcatgctgctaacctggctt**

**SamoaDF**  **cgcaagcggagcagggcctacgggagattgtggcgccgagtcagtcggacatcgtgcttatcatgctgctaacctggctt**

**SamoaDI cgcaagcggagcagggcctacgggagattgtggcgccgagtcagtcggacatcgtgcttatcatgctgctaacctggctt**

**CDC2F**  **cgcaagcggagcagggcctacgggagattgtggcgccgagtcagtcggacatcgtgcttatcatgctgctaacctggctt**

**CDC2I**  **cgcaagcggagcagggcctacgggagattgtggcgccgagtcagtcggacatcgtgcttatcatgctgctaacctggctt**

**BosniaAI cgcaagcggagcagggcctacgggagattgtggcgccgagtcagtcggacatcgtgcttatcatgctgctaacctggctt**

**IraqBI cgcaagcggagcagggcctacgggagattgtggcgccgagtcagtcggacatcgtgcttatcatgctgctaacctggctt**

**Fribourg-BlancF** **cgcaagcggagcagggcctacgggagattgtggcgccgagtcagtcggacatcgtgcttatcatgctgctaacctggctt**

**Fribourg-BlancI** **cgcaagcggagcagggcctacgggagattgtggcgccgagtcagtcggacatcgtgcttatcatgctgctaacctggctt**

1210 1220 1230 1240 1250 1260 1270 1280

....|....|....|....|....|....|....|....|....|....|....|....|....|....|....|....|

**NicholsF**  **--------------------------------------------------------------------------------**

**Bal3F**  **--------------------------------------------------------------------------------**

**Sea81-4F**  **--------------------------------------------------------------------------------**

**MexicoAF**  **--------------------------------------------------------------------------------**

**Street14F --------------------------------------------------------------------------------**

**NicholsI**  **gagcgtgcacggctggaccggttcaatgctgatgcgctgcttacggcgcagtggacctatgtgtcggctggactgtatgg**

**Bal3I gagcgtgcacggctggaccggttcaatgctgatgcgctgcttacggcgcagtggacctatgtgtcggctggactgtatgg**

**Sea81-4I**  **gagcgtgcacggctggaccggttcaatgctgatgcgctgcttacggcgcagtggacctatgtgtcggctggactgtatgg**

**MexicoAI**  **gagcgtgcacggctggaccggttcaatgctgatgcgctgcttacggcgcagtggacctatgtgtcggctggactgtatgg**

**Street14I gagcgtgcacggctggaccggttcaatgctgatgcgctgcttacggcgcagtggacctatgtgtcggctggactgtatgg**

**GauthierF**  **gagcgtgcacggctggaccggttcaatgctgatgcgctgcttacggcgcagtggacctatgtgtcggctggactgtatgg**

**GauthierI**  **gagcgtgcacggctggaccggttcaatgctgatgcgctgcttacggcgcagtggacctatgtgtcggctggactgtatgg**

**SamoaDF**  **gagcgtgcacggctggaccggttcaatgctgatgcgctgcttacggcgcagtggacctatgtgtcggctggactgtatgg**

**SamoaDI gagcgtgcacggctggaccggttcaatgctgatgcgctgcttacggcgcagtggacctatgtgtcggctggactgtatgg**

**CDC2F**  **gagcgtgcacggctggaccggttcaatgctgatgcgctgcttacggcgcagtggacctatgtgtcggctggactgtatgg**

**CDC2I**  **gagcgtgcacggctggaccggttcaatgctgatgcgctgcttacggcgcagtggacctatgtgtcggctggactgtatgg**

**BosniaAI gagcgtgcacggctggaccggttcaatgctgatgcgctgcttacggcgcagtggacctatgtgtcggctggactgtatgg**

**IraqBI gagcgtgcacggctggaccggttcaatgctgatgcgctgcttacggcgcagtggacctatgtgtcggctggactgtatgg**

**Fribourg-BlancF** **gagcgtgcacggctggaccggttcaatgctgatgcgctgcttacggcgcagtggacctatgtgtcggctggactgtatgg**

**Fribourg-BlancI** **gagcgtgcacggctggaccggttcaatgctgatgcgctgcttacggcgcagtggacctatgtgtcggctggactgtatgg**

1290 1300 1310 1320 1330 1340 1350 1360

....|....|....|....|....|....|....|....|....|....|....|....|....|....|....|....|

**NicholsF**  **--------------------------------------------------------------------------------**

**Bal3F**  **--------------------------------------------------------------------------------**

**Sea81-4F**  **--------------------------------------------------------------------------------**

**MexicoAF**  **--------------------------------------------------------------------------------**

**Street14F --------------------------------------------------------------------------------**

**NicholsI**  **ggcgacggcgggtaccaatgtatttggtaagcgcgtgctgcctgcgctgcggtcctggcattttgattttgccggattcc**

**Bal3I ggcgacggcgggtaccaatgtatttggtaagcgcgtgctgcctgcgctgcggtcctggcattttgattttgccggattcc**

**Sea81-4I**  **ggcgacggcgggtaccaatgtatttggtaagcgcgtgctgcctgcgctgcggtcctggcattttgattttgccggattcc**

**MexicoAI**  **ggcgacggcgggtaccaatgtatttggtaagcgcgtgctgcctgcgctgcggtcctggcattttgattttgccggattcc**

**Street14I ggcgacggcgggtaccaatgtatttggtaagcgcgtgctgcctgcgctgcggtcctggcattttgattttgccggattcc**

**GauthierF**  **ggcgacggcgggtaccaatgtatttggtaagcgcgtgctgcctgcgctgcagtcctggcattttgattttgctggattcc**

**GauthierI**  **ggcgacggcgggtaccaatgtatttggtaagcgcgtgctgcctgcgctgcagtcctggcattttgattttgctggattcc**

**SamoaDF**  **ggcgacggcgggtaccaatgtatttggtaagcgcgtgctgcctgcgctgcagtcctggcattttgattttgctggattcc**

**SamoaDI ggcgacggcgggtaccaatgtatttggtaagcgcgtgctgcctgcgctgcagtcctggcattttgattttgctggattcc**

**CDC2F**  **ggcgacggcgggtaccaatgtatttggtaagcgcgtgctgcctgcgctgcagtcctggcattttgattttgctggattcc**

**CDC2I**  **ggcgacggcgggtaccaatgtatttggtaagcgcgtgctgcctgcgctgcagtcctggcattttgattttgctggattcc**

**BosniaAI ggcgacggcgggtaccaatgtatttggtaagcgcgtgctgcctgcgctgcagtcctggcattttgattttgctggattcc**

**IraqBI ggcgacggcgggtaccaatgtatttggtaagcgcgtgctgcctgcgctgcagtcctggcattttgattttgctggattcc**

**Fribourg-BlancF** **ggcgacggcgggtaccaatgtatttggtaagcgcgtgctgcctgcgctgcagtcctggcattttgattttgctggattcc**

**Fribourg-BlancI** **ggcgacggcgggtaccaatgtatttggtaagcgcgtgctgcctgcgctgcagtcctggcattttgattttgctggattcc**

1370 1380 1390 1400 1410 1420 1430 1440

....|....|....|....|....|....|....|....|....|....|....|....|....|....|....|....|

**NicholsF**  **--------------------------------------------------------------------------------**

**Bal3F**  **--------------------------------------------------------------------------------**

**Sea81-4F**  **--------------------------------------------------------------------------------**

**MexicoAF**  **--------------------------------------------------------------------------------**

**Street14F --------------------------------------------------------------------------------**

**NicholsI**  **tcaaactcgaaaccaaaagcggtgacccctacacccacctgctcaccggcctgaacgccggcgtcgaagcacgcgtgtac**

**Bal3I tcaaactcgaaaccaaaagcggtgacccctacacccacctgctcaccggcctgaacgccggcgtcgaagcacgcgtgtac**

**Sea81-4I**  **tcaaactcgaaaccaaaagcggtgacccctacacccacctgctcaccggcctgaacgccggcgtcgaagcacgcgtgtac**

**MexicoAI**  **tcaaactcgaaaccaaaagcggtgacccctacacccacctgctcaccggcctgaacgccggcgtcgaagcacgcgtgtac**

**Street14I tcaaactcgaaaccaaaagcggtgacccctacacccacctgctcaccggcctgaacgccggcgtcgaagcacgcgtgtac**

**GauthierF**  **ttaagctcgaaactaagagcggtgacccctacacccacctgctcaccggcctgaacgccggcgtcgaagcacgcgtgtac**

**GauthierI**  **ttaagctcgaaactaagagcggtgacccctacacccacctgctcaccggcctgaacgccggcgtcgaagcacgcgtgtac**

**SamoaDF**  **ttaagctcgaaactaagagcggtgacccctacacccacctgctcaccggcctgaacgccggcgtcgaagcacgcgtgtac**

**SamoaDI ttaagctcgaaactaagagcggtgacccctacacccacctgctcaccggcctgaacgccggcgtcgaagcacgcgtgtac**

**CDC2F**  **ttaagctcgaaactaagagcggtgacccctacacccacctgctcaccggcctgaacgccggcgtcgaagcacgcgtgtac**

**CDC2I**  **ttaagctcgaaactaagagcggtgacccctacacccacctgctcaccggcctgaacgccggcgtcgaagcacgcgtgtac**

**BosniaAI ttaagctcgaaactaagagcggtgacccctacacccacctgctcaccggcctgaacgccggcgtcgaagcacgcgtgtac**

**IraqBI ttaagctcgaaactaagagcggtgacccctacacccacctgctcaccggcctgaacgccggcgtcgaagcacgcgtgtac**

**Fribourg-BlancF** **ttaagctcgaaactaagagcggtgacccctacacccacctgctcaccggcctgaacgccggcgtcgaagcacgcgtgtac**

**Fribourg-BlancI** **ttaagctcgaaactaagagcggtgacccctacacccacctgctcaccggcctgaacgccggcgtcgaagcacgcgtgtac**

1450 1460 1470 1480 1490 1500 1510 1520

....|....|....|....|....|....|....|....|....|....|....|....|....|....|....|....|

**NicholsF**  **--------------------------------------------------------------------------------**

**Bal3F**  **--------------------------------------------------------------------------------**

**Sea81-4F**  **--------------------------------------------------------------------------------**

**MexicoAF**  **--------------------------------------------------------------------------------**

**Street14F --------------------------------------------------------------------------------**

**NicholsI**  **atccccctcacctacatccgttacagaaataacggagggtacgaactgaatggagctgtgccccctgggactatcaatat**

**Bal3I atccccctcacctacatccgttacagaaataacggagggtacgaactgaatggagctgtgccccctgggactatcaatat**

**Sea81-4I**  **atccccctcacctacatccgttacagaaataacggagggtacgaactgaatggagctgtgccccctgggactatcaatat**

**MexicoAI**  **atccccctcacctacatccgttacagaaataacggagggtacgaactgaatggagctgtgccccctgggactatcaatat**

**Street14I atccccctcacctacatccgttacagaaataacggagggtacgaactgaatggagctgtgccccctgggactatcaatat**

**GauthierF**  **atccccctcacctacatccgttacagaaataacggagggtacccactgaatggggttgtgccccctgggactatcaatat**

**GauthierI**  **atccccctcacctacatccgttacagaaataacggagggtacccactgaatggggttgtgccccctgggactatcaatat**

**SamoaDF**  **atccccctcacctacatccgttacagaaataacggagggtacccactgaatggagttgtgccccctgggactatcaatat**

**SamoaDI atccccctcacctacatccgttacagaaataacggagggtacccactgaatggagttgtgccccctgggactatcaatat**

**CDC2F**  **atccccctcacctacatccgttacagaaataacggagggtacccactgaatggagttgtgccccctgggactatcaatat**

**CDC2I**  **atccccctcacctacatccgttacagaaataacggagggtacccactgaatggagttgtgccccctgggactatcaatat**

**BosniaAI atccccctcacctacatccgttacagaaataacggagggtacccactgaatggagttgtgccccctgggactatcaatat**

**IraqBI atccccctcacctacatccgttacagaaataacggagggtacccactgaatggagttgtgccccctgggactatcaatat**

**Fribourg-BlancF** **atccccctcacctacatccgttacagaaataacggagggtacctactggatggagttgtgccccctgggactatcaatat**

**Fribourg-BlancI** **atccccctcacctacatccgttacagaaataacggagggtacctactggatggagttgtgccccctgggactatcaatat**

1530 1540 1550 1560 1570 1580 1590 1600

....|....|....|....|....|....|....|....|....|....|....|....|....|....|....|....|

**NicholsF**  **--------------------------------------------------------------------------------**

**Bal3F**  **--------------------------------------------------------------------------------**

**Sea81-4F**  **--------------------------------------------------------------------------------**

**MexicoAF**  **--------------------------------------------------------------------------------**

**Street14F --------------------------------------------------------------------------------**

**NicholsI**  **gccaattttggggaaggcgtggtgcagctatcgcatccccctcggttcccacgcctggcttacaccgcatacatccgtgc**

**Bal3I gccaattttggggaaggcgtggtgcagctatcgcatccccctcggttcccacgcctggcttacaccgcatacatccgtgc**

**Sea81-4I**  **gccaattttggggaaggcgtggtgcagctatcgcatccccctcggttcccacgcctggcttacaccgcatacatccgtgc**

**MexicoAI**  **gccaattttggggaaggcgtggtgcagctatcgcatccccctcggttcccacgcctggcttacaccgcatacatccgtgc**

**Street14I gccaattttggggaaggcgtggtgcagctatcgcatccccctcggttcccacgcctggcttacaccgcatacatccgtgc**

**GauthierF**  **gccgattttggggaaggcgtggtgcagctatcgcatccccctcggttcccacgcctggcttacaccgcatacatccgtgc**

**GauthierI**  **gccgattttggggaaggcgtggtgcagctatcgcatccccctcggttcccacgcctggcttacaccgcatacatccgtgc**

**SamoaDF**  **gccgattttggggaaggcgtggtgcagctatcgcatccccctcggttcccacgcctggcttacaccgcatacatccgtgc**

**SamoaDI gccgattttggggaaggcgtggtgcagctatcgcatccccctcggttcccacgcctggcttacaccgcatacatccgtgc**

**CDC2F**  **gccgattttggggaaggcgtggtgcagctatcgcatccccctcggttcccacgcctggcttacaccgcatacatccgtgc**

**CDC2I**  **gccgattttggggaaggcgtggtgcagctatcgcatccccctcggttcccacgcctggcttacaccgcatacatccgtgc**

**BosniaAI gccgattttggggaaggcgtggtgcagctatcgcatccccctcggttcccacgcctggcttacaccgcatacatccgtgc**

**IraqBI gccgattttggggaaggcgtggtgcagctatcgcatccccctcggttcccacgcctggcttacaccgcatacatccgtgc**

**Fribourg-BlancF** **gccgattttggggaaggcgtggtgcagctatcgcatccccctcggttcccacgcctggcttacaccgcatacatccgtgc**

**Fribourg-BlancI** **gccgattttggggaaggcgtggtgcagctatcgcatccccctcggttcccacgcctggcttacaccgcatacatccgtgc**

1610 1620 1630 1640 1650 1660 1670 1680

....|....|....|....|....|....|....|....|....|....|....|....|....|....|....|....|

**NicholsF**  **--------------------------------------------------------------------------------**

**Bal3F**  **--------------------------------------------------------------------------------**

**Sea81-4F**  **--------------------------------------------------------------------------------**

**MexicoAF**  **--------------------------------------------------------------------------------**

**Street14F --------------------------------------------------------------------------------**

**NicholsI**  **tcggcacaaccaatcgctttaacgttattaaccccgcgtacaccctgttgaatgaacgagcgctccagtaccaggtggga**

**Bal3I tcggcacaaccaatcgctttaacgttattaaccccgcgtacaccctgttgaatgaacgagcgctccagtaccaggtggga**

**Sea81-4I**  **tcggcacaaccaatcgctttaacgttattaaccccgcgtacaccctgttgaatgaacgagcgctccagtaccaggtggga**

**MexicoAI**  **tcggcacaaccaatcgctttaacgttattaaccccgcgtacaccctgttgaatgaacgagcgctccagtaccaggtggga**

**Street14I tcggcacaaccaatcgctttaacgttattaaccccgcgtacaccctgttgaatgaacgagcgctccagtaccaggtggga**

**GauthierF**  **tcggcacaaccaatcgctttaacgttattaaccccgcgcacaccctgttgaatgaacgagcgctccagtaccaggtggga**

**GauthierI**  **tcggcacaaccaatcgctttaacgttattaaccccgcgcacaccctgttgaatgaacgagcgctccagtaccaggtggga**

**SamoaDF**  **tcggcacaaccaatcgctttaacgttattaaccccgcgtacaccctgttgaatgaacgagcgctccagtaccaggtggga**

**SamoaDI tcggcacaaccaatcgctttaacgttattaaccccgcgtacaccctgttgaatgaacgagcgctccagtaccaggtggga**

**CDC2F**  **tcggcacaaccaatcgctttaacgttattaaccccgcgtacaccctgttgaatgaacgagcgctccagtaccaggtggga**

**CDC2I**  **tcggcacaaccaatcgctttaacgttattaaccccgcgtacaccctgttgaatgaacgagcgctccagtaccaggtggga**

**BosniaAI tcggcacaaccaatcgctttaacgttattaaccccgcgtacaccctgttgaatgaacgagcgctccagtaccaggtggga**

**IraqBI tcggcacaaccaatcgctttaacgttattaaccccgcgtacaccctgttgaatgaacgagcgctccagtaccaggtggga**

**Fribourg-BlancF** **tcggcacaaccaatcgctttaacgttattaaccccgcgtacaccctgttgaatgaacgagcgctccagtaccaggtggga**

**Fribourg-BlancI** **tcggcacaaccaatcgctttaacgttattaaccccgcgtacaccctgttgaatgaacgagcgctccagtaccaggtggga**

1690 1700 1710 1720 1730 1740 1750 1760

....|....|....|....|....|....|....|....|....|....|....|....|....|....|....|....|

**NicholsF**  **-----------------------------------cgcccagtgggaacagggggtgcttgctgacgctccttacatggg**

**Bal3F**  **-----------------------------------cgcccagtgggaacagggggtgcttgctgacgctccttacatggg**

**Sea81-4F**  **-----------------------------------cgcccagtgggaacagggggtgcttgctgacgctccttacatggg**

**MexicoAF**  **-----------------------------------cgcccagtgggaacagggggtgcttgctgacgctccttacatggg**

**Street14F -----------------------------------cgcccagtgggaacagggggtgcttgctgacgctccttacatggg**

**NicholsI**  **ctgacgttcagtcccttcgagaaggtggagctcagcgcccagtgggaacagggggtgcttgctgacgctccttacatggg**

**Bal3I ctgacgttcagtcccttcgagaaggtggagctcagcgcccagtgggaacagggggtgcttgctgacgctccttacatggg**

**Sea81-4I**  **ctgacgttcagtcccttcgagaaggtggagctcagcgcccagtgggaacagggggtgcttgctgacgctccttacatggg**

**MexicoAI**  **ctgacgttcagtcccttcgagaaggtggagctcagcgcccagtgggaacagggggtgcttgctgacgctccttacatggg**

**Street14I ctgacgttcagtcccttcgagaaggtggagctcagcgcccagtgggaacagggggtgcttgctgacgctccttacatggg**

**GauthierF**  **ctgacgttcagtcccttcgagaaggtggagctcagcgcccagtgggaacagggggtgcttgctgacgctccttacatggg**

**GauthierI**  **ctgacgttcagtcccttcgagaaggtggagctcagcgcccagtgggaacagggggtgcttgctgacgctccttacatggg**

**SamoaDF**  **ctgacgttcagtcccttcgagaaggtggagctcagcgcccagtgggaacagggggtgcttgctgacgctccttacatggg**

**SamoaDI ctgacgttcagtcccttcgagaaggtggagctcagcgcccagtgggaacagggggtgcttgctgacgctccttacatggg**

**CDC2F**  **ctgacgttcagtcccttcgagaaggtggagctcagcgcccagtgggaacagggggtgcttgctgacgctccttacatggg**

**CDC2I**  **ctgacgttcagtcccttcgagaaggtggagctcagcgcccagtgggaacagggggtgcttgctgacgctccttacatggg**

**BosniaAI ctgacgttcagtcccttcgagaaggtggagctcagcgcccagtgggaacagggggtgctctccgatgttccctacatggg**

**IraqBI ctgacgttcagtcccttcgagaaggtggagctcagcgcccagtgggaacagggggtgctctccgatgttccctacatggg**

**Fribourg-BlancF** **ctgacgttcagtcccttcgrgaaggtggagctcagcgcccagtgggaacagggggtgcttgctgacgctccttacatggg**

**Fribourg-BlancI** **ctgacgttcagtcccttcgagaaggtggagctcagcgcccagtgggaacagggggtgcttgctgacgctccttacatggg**

1770 1780 1790 1800 1810 1820 1830

....|....|....|....|....|....|....|....|....|....|....|....|....|....|

**NicholsF**  **tattgccgagagtatgtggtctgagcgttactttggcacgtttatctgtggggtgaaggtggtttggtga**

**Bal3F**  **tattgccgagagtatgtggtctgagcgttactttggcacgtttatctgtggggtgaaggtggtttggtga**

**Sea81-4F**  **tattgccgagagtatgtggtctgagcgttactttggcacgtttatctgtggggtgaaggtggtttggtga**

**MexicoAF**  **tattgccgagagtatgtggtctgagcgttactttggcacgtttatctgtggggtgaaggtggtttggtga**

**Street14F tattgccgagagtatgtggtctgagcgttactttggcacgtttatctgtggggtgaaggtggtttggtga**

**NicholsI**  **tattgccgagagtatgtggtctgagcgttactttggcacgtttatctgtggggtgaaggtggtttggtga**

**Bal3I tattgccgagagtatgtggtctgagcgttactttggcacgtttatctgtggggtgaaggtggtttggtga**

**Sea81-4I**  **tattgccgagagtatgtggtctgagcgttactttggcacgtttatctgtggggtgaaggtggtttggtga**

**MexicoAI**  **tattgccgagagtatgtggtctgagcgttactttggcacgtttatctgtggggtgaaggtggtttggtga**

**Street14I tattgccgagagtatgtggtctgagcgttactttggcacgtttatctgtggggtgaaggtggtttggtga**

**GauthierF**  **tattgccgagggtatgtggtctgagcgttactttggcacgtttatctgtggggtgaaggtggtttggtga**

**GauthierI**  **tattgccgagggtatgtggtctgagcgttactttggcacgtttatctgtggggtgaaggtggtttggtga**

**SamoaDF**  **tattgccgagagtaagtggtctgagcgttactttggcacgtttatctgtggggtgaacgtgggttggtga**

**SamoaDI tattgccgagagtatgtggtctgagcgttactttggcacgtttatctgtggggtgaaggtggtttggtga**

**CDC2F**  **tattgccgagagtaagtggtctgagcgttactttggcacgtttatctgtggggtgaacgtgggttggtga**

**CDC2I**  **tattgccgagagtatgtggtctgagcgttactttggcacgtttatctgtggggtgaaggtggtttggtga**

**BosniaAI cattgccgagagtatgtggtctgagcgttactttggcacgtttatctgtggggtgaaggtggtttggtga**

**IraqBI cattgccgagagtatgtggtctgagcgttactttggcacgtttatctgtggggtgaaggtggtttggtga**

**Fribourg-BlancF** **tattgccgagagtatgtggtctgagcgttactttggcacgtttatctgtggggtgaaggtggtttggtga**

**Fribourg-BlancI** **tattgccgagagtatgtggtctgagcgttactttggcacgtttatctgtggggtgaaggtggtttggtga**

***1.6.1 tprG* Locus containing *tprG* genes or *tprJI* chimeras**

10 20 30 40 50 60 70 80

....|....|....|....|....|....|....|....|....|....|....|....|....|....|....|....|

**NicholsG**  **MGCMRWGSVLCVVVGVGASGGVLGQEFSPKLTGSATLEWGISYGKGVGSHGQAPGAVMGTGPYNLKHGFRTTNTVGVSFP**

**Street14G MGCMRWGSVLCVVVGVGASGGVLGQEFSPKLTGSATLEWGISYGKGVGSHGQAPGAVMGTGPYNLKHGFRTTNTVGVSFP**

**Bal3G**  **MGCMRWGSVLCVVVGVGASGGVLGQEFSPKLTGSATLEWGISYGKGVGSHGQAPGAVMGTGPYNLKHGFRTTNTVGVSFP**

**MexicoAG**  **MGCMRWGSVLCVVVGVGASGGVLGQEFSPKLTGSATLEWGISYGKGVGSHGQAPGAVMGTGPYNLKHGFRTTNTVGVSFP**

**GauthierG** **MGCMRWGSVLCVVVGVGASGGXLGQEFSPKLTGSATLEWGISYGKGVGSHGQAPGAVMGTGPYNLRHGFRTTNTVGVSFP**

**CDC2G**  **MGCMRWGSVLCVVVGVGASGGVLGQEFSPKLTGSATLEWGISYGKGVGSHGQAPGAVMGTGPYNLKHGFRTTNTVGVSFP**

**Sea81-4G**  **MGCMRWGSVLCVVVGVGASGGVLGQEFPPKLTGSATLEWGISYGKGVGSHGQAPGAVMGTGPYNLKHGFRTTNTVGVSFP**

90 100 110 120 130 140 150 160

....|....|....|....|....|....|....|....|....|....|....|....|....|....|....|....|

**NicholsG**  **LVMRTTHTRRGQHPALYAELKVADLQADLSQGKAGFAVKRKGKVEATLHCYGAYLTIGKNPTFLTNFARLWKPWVTAQYQ**

**Street14G LVMRTTHTRRGQHPALYAELKVADLQADLSQGKAGFAVKRKGKVEATLHCYGAYLTIGKNPTFLTNFARLWKPWVTAQYQ**

**Bal3G**  **LVMRTTHTRRGQHPALYAELKVADLQADLSQGKAGFAVKRKGKVEATLHCYGAYLTIGKNPTFLTNFARLWKPWVTAQYQ**

**MexicoAG**  **LVMRTTHTRRGQHPALYAELKVADLQADLSQGKAGFAVKRKGKVEATLHCYGAYLTIGKNPTFLTNFARLWKPWVTAQYQ**

**GauthierG** **LVMRTTHTRRGQHPALYAELKVADLQADLSQGKAGFAVKRKGKVEATLHCYGAYLTIGKNPTFLTNFARLWKPWVTAQYQ**

**CDC2G**  **LVMRTTHTRRGQHPALYAELKVADLQADLSQGKAGFAVKRKGKVEATLHCYGAYLTIGKNPTFLTNFARLWKPWVTAQYQ**

**Sea81-4G**  **LVMRTTHTRRGQHPALYAELKVADLQADLSQGKAGFAVKRKGKVEATLHCYGAYLTIGKNPTFLTNFARLWKPWVTAQYQ**

170 180 190 200 210 220 230 240

....|....|....|....|....|....|....|....|....|....|....|....|....|....|....|....|

**NicholsG**  **EDAVQYAPGFGGLGGKVGYRAQDIGGSGVSLDVGFLSFASNGAWDSTDPTHSKYGFGADLKLMYARAGHPLCTVELASNV**

**Street14G EDAVQYAPGFGGLGGKVGYRAQDIGGSGVSLDVGFLSFASNGAWDSTDPTHSKYGFGADLKLMYARAGHPLCTVELASNV**

**Bal3G**  **EDAVQYAPGFGGLGGKVGYRAQDIGGSGVSLDVGFLSFASNGAWDSTDPTHSKYGFGADLKLMYARAGHPLCTVELASNV**

**MexicoAG**  **EDAVQYAPGFGGLGGKVGYRAQDIGGSGVSLDVGFLSFASNGAWDSTDPTHSKYGFGADLKLMYARAGHPLCTVELASNV**

**GauthierG** **EDAVQYAPGFGGLGGKVGYRAQDIGGSGVSLDVGFLSFASNGAWDSTDPTHSKYGFGADLKLMYARAGHPLCTVELASNV**

**CDC2G**  **EDAVQYAPGFGGLGGKVGYRAQDIGGSGVSLDVGFLSFASNGAWDSTDPTHSKYGFGADLKLMYARAGHPLCTVELASNV**

**Sea81-4G**  **EDAVQYAPGFGGLGGKVGYRAQDIGGSGVSLDVGFLSFASNGAWDSTDPTHSKYGFGADLKLMYARAGHPLCTVELASNV**

250 260 270 280 290 300 310 320

....|....|....|....|....|....|....|....|....|....|....|....|....|....|....|....|

**NicholsG**  **TLEDGYLIGAQKDANNQNKDKLLWNVGGRLTLEPGAGFRFSFALDAGNQHQSAQDFQNRTQRAQSELTALSNNLFQGESQ**

**Street14G TLEDGYLIGAQKDANNQNKDKLLWNVGGRLTLEPGAGFRFSFALDAGNQHQSAQDFQNRTQRAQSELTALSNNLFQGESQ**

**Bal3G**  **TLEDGYLIGAQKDANNQNKDKLLWNVGGRLTLEPGAGFRFSFALDAGNQHQSAQDFQNRTQRAQSELTALSNNLFQGESQ**

**MexicoAG**  **TLEDGYLIGAQKDANNQNKDKLLWNVGGRLTLEPGAGFRFSFALDAGNQHQSAQDFQNRTQRAQSELTALSNNLFQGESQ**

**GauthierG** **TLEDGYLIGAQKDANNQNKDKLLWNVGGRLTLEPGAGFRFSFALDAGNQHQSAQDFQNRTQRAQSELTALSNNLFQGESQ**

**CDC2G**  **TLEDGYLIGAQKDANNQNKDKLLWNVGGRLTLEPGAGFRFSFALDAGNQHQSAQDFQNRTQRAQSELTALSNNLFQGESQ**

**Sea81-4G**  **TLEDGYLIGAQKDANNQNKDKLLWNVGGRLTLEPGAGFRFSFALDAGNQHQSAQDFQNRTQRAQSELTALSNNLFQGESQ**

330 340 350 360 370 380 390 400

....|....|....|....|....|....|....|....|....|....|....|....|....|....|....|....|

**NicholsG**  **KQEAWLDEYAKKVLDAVTAATETALQSRGNAYITAVSNVKVTPPVAATLLTNLKVFITDPPTPSPLPALPAFSLMGQVLL**

**Street14G KQEAWLDEYAKKVLDAVTAATETALQSRGNAYITAVSNVKVTPPVAATLLTNLKVFITDPPTPSPLPALPAFSLMGQVLL**

**Bal3G**  **KQEAWLDEYAKKVLDAVTAATETALQSRGNAYITAVSNVKVTPPVAATLLTNLKVFITDPPTPSPLPALPAFSLMGQVLP**

**MexicoAG**  **KQEAWLDEYAKKVLDAVTAATETALQSRGNAYITAVSNVKVTPPVAATLLTNLKVFITDPPTPSPLPALPAFSLMGQVLL**

**GauthierG** **KQEAWLDEYAKKVLDAVTAATETALQSRGNAYITAVSNVKVTPPVAATLLTNLKVFITDPPTPSPLPALPAFSLMGQVLL**

**CDC2G**  **KQEAWLDEYAKKVLDAVTAATETALQSRGNAYITAVSNVKVTPPVAATLLTNLKVFITDPPTPSPLPALPAFSLMGQVLL**

**Sea81-4G**  **KQEAWLDEYAKKVLDAVTAATETALQSRGNAYITAVSNVKVTPPVAATLLTNLKVFITDPPTPSPLPALPAFSLMGQVLL**

410 420 430 440 450 460 470 480

....|....|....|....|....|....|....|....|....|....|....|....|....|....|....|....|

**NicholsG**  **QYDAEQVVKGFEQVQTQIVAEINQKVQAAVAQSKAAAQAFINGLTKAIEDVADALLAPHKGNPMSLFNLPDQQKLLKDDL**

**Street14G QYDAEQVVKGFEQVQTQIVAEINQKVQAAVAQSKAAAQAFINGLTKVIEDVADALLAPHKGNPMSLFNLPDQQKLLKDDL**

**Bal3G**  **QYDAEQVVKGFEQVQTQIVAEINQKVQAAVAQSKAAAQAFINGLTKAIEDVADALLAPHKGNPMSLFNLPDQQKLLKDDL**

**MexicoAG**  **QYDAEQVVKGFEQVQTQIVAEINQKVQAAVAQSKAAAQAFINGLTKVIEDVADALLAPHKGNPMSLFNLPDQQKLLKDDL**

**GauthierG** **QYDAEQVVKGFEQVQTQIVAEINQKVQAAVAQSKAAAQAFINGLTKAIEDVADALLAPHKGNPMSLFNLPDQQKLLKDDL**

**CDC2G**  **QYDAEQVVKGFEQVQTQIVAEINQKVQAAVAQSKAAAQAFINGLTKAIEDVADALLAPHKGNPMSLFNLPDQQKLLKDDL**

**Sea81-4G**  **QYDAEQVVKGFEQVQTQIVAEINQKVQAAVAQSKAAAQAFINGLTKAIEDVADALLAPHKGNPMSLFNLPDQQKLLKDDL**

490 500 510 520 530 540 550 560

....|....|....|....|....|....|....|....|....|....|....|....|....|....|....|....|

**NicholsG**  **ADLIPKLTAEATKFFTEGQTFVTEEVKKKTDALDAGQQIRQAIQNLRASAWRAFLMGVSAVCLYLDTYNVAFDALFTAQW**

**Street14G ADLIPKLTAEATKFFTEGQTFVTEEVKKKTDALDAGQQIRQAIQNLRASAWRAFLMGVSAVCLYLDTYNVAFDALFTAQW**

**Bal3G**  **ADLIPKLTAEATKFFTEGQTFVTEEVKKKTDALDAGQQIRQAIQNLRASAWRAFLMGVSAVCLYLDTYNVAFDALFTAQW**

**MexicoAG**  **ADLIPKLTAEATKFFTEGQTFVTEEVKKKTDALDAGQQIRQAIQNLRASAWRAFLMGVSAVCLYLDTYNVAFDALFTAQW**

**GauthierG** **ADLIPKLTAEATKFFTEGQTFVTEEVKKKTDALDAGQQIRQAIQNLRASAWRAFLMGVSAVCLYLDTYNVAFDALLTAQW**

**CDC2G**  **ADLIPKLTAEATKFFTEGQTFVTEEVKKKTDALDAGQQIRQAIQNLRASAWRAFLMGVSAVCLYLDTYNVAFDALLTAQW**

**Sea81-4G**  **ADLIPKLTAEATKFFTEGQTFVTEEVKKKTDALDAGQQIRQAIQNLRASAWRAFLMGVSAVCLYLDTYNVAFDALFTAQW**

570 580 590 600 610 620 630 640

....|....|....|....|....|....|....|....|....|....|....|....|....|....|....|....|

**NicholsG**  **KWLSSGIYFATAPANVFGTRVLDNTIASCGDFAGFLKLETKSGDPYTHLLTGLDAGVETRVYIPLTYALYKNNGGTAVRG**

**Street14G KWLSSGIYFATAPANVFGTRVLDNTIASCGDFAGFLKLETKSGDPYTHLLTGLDAGVETRVYIPLTYALYKNNGGTAVRG**

**Bal3G**  **KWLSSGIYFATAPANVFGTRVLDNTIASCGDFAGFLKLETKSGDPYTHLLTGLDAGVETRVYIPLTHDLYKNNNGNPLPS**

**MexicoAG**  **KWLSSGIYVATAPANVFGTRVLDNTIASCGDFAGFLKLETKSGDPYTHLLTGLDAGVETRVYIPLTHDLYKNNNGNPLPS**

**GauthierG** **KWLSSGIYFATAPANVFGTRVLDNTIASCGDFAGFLKLETKSGDPYTHLLTGLDAGVETRVYIPLTHDLYKNNNGNPLPS**

**CDC2G**  **KWLSSGIYFATAPANVFGTRVLDNTIASCGDFAGFLKLETKSGDPYTHLLTGLDAGVETRVYIPLTHDLYKNNNGNPLPS**

**Sea81-4G**  **KWLSSGIYVATAPANVFGTRVLDNTIASCGDFAGFLKLETKSGDPYTHLLTGLDAGVETRVYIPLTHDAIQK**

650 660 670 680 690 700 710 720

....|....|....|....|....|....|....|....|....|....|....|....|....|....|....|....|

**NicholsG**  **IQEKEYIRPPVVGKAWCSYRIPVQDYGWVKPSVTVHASTNRAHLNAPAAGGAVGATYLTKEYCAQLRAGISASLIEKTVF**

**Street14G IQEKEYIRPPVVGKAWCSYRIPVQDYGWVKPSVTVHASTNRAHLNAPAAGGAVGATYLTKEYCAQLRAGISASLIEKTVF**

**Bal3G**  **GGSSGHIGLPVVGKAWCSYRIPVQDYGWVKPSVTVHASTNRAHLNAPAAGGAVGATYLTKEYCAQLRAGISASLIEKTVF**

**MexicoAG**  **GGSSGHIGLPVVGKAWCSYRIPVQDYGWVKPSVTVHASTNRAHLNAPAAGGAVGATYLTKEYCAQLRAGVSASLIEKTVF**

**GauthierG** **GGSSGHIGLPVVGKAWCSYRIPVQDYGWVKPSVTVHASTNRAHLNAPAAGGAVGATYLTKEYCAQLRAGVSASLIEKTVF**

**CDC2G**  **GGSSGHIGLPVVGKAWCSYRIPVQDYGWVKPSVTVHASTNRAHLNAPAAGGAVGATYLTKEYCAQLRAGVSASLIEKTVF**

**Sea81-4G**

730 740 750

....|....|....|....|....|....|....|.

**NicholsG**  **SLDWEQGMLSDVPYLLVSECLTQGIGRIVCGVTLSW**

**Street14G SLDWEQGMLSDVPYLLVSECLTQGIGRIVCGVTLSW**

**Bal3G**  **SLDWEQGMLSDVPYLLVSECLTQGIGRIVCGVTLSW**

**MexicoAG**  **SLDWEQGMLSDVPYLLVSECLTQGIGRIVCGVTLSW**

**GauthierG** **SLDWEQGMLSDVPYLLVSECLTQGIGRIVCGVTLSW**

**CDC2G**  **SLDWEQGMLSDVPYLLVSECLTQGIGRIVCGVTLSW**

**Sea81-4G**

10 20 30 40 50 60 70 80

....|....|....|....|....|....|....|....|....|....|....|....|....|....|....|....|

**NicholsG**  **atggggtgcatgcggtgggggagtgtgctgtgtgtggtggtgggggtaggagcgagcgggggagtgctcggacaggagtt**

**Street14G atggggtgcatgcggtgggggagtgtgctgtgtgtggtggtgggggtaggagcgagcgggggagtgctcggacaggagtt**

**Bal3G**  **atggggtgcatgcggtgggggagtgtgctgtgtgtggtggtgggggtaggagcgagcgggggagtgctcggacaggagtt**

**MexicoAG**  **atggggtgcatgcggtgggggagtgtgctgtgtgtggtggtgggggtaggagcgagcgggggagtgctcggacaggagtt**

**GauthierG** **atggggtgcatgcggtgggggagtgtgctgtgtgtggtggtgggggtaggagcgagcgggggantgctcggacaggagtt**

**CDC2G**  **atggggtgcatgcggtgggggagtgtgctgtgtgtggtggtgggggtaggagcgagcgggggagtgctcggacaggagtt**

**Sea81-4G**  **atggggtgcatgcggtgggggagtgtgctgtgtgtggtggtgggggtaggagcgagcgggggagtgctcggacaggagtt**

90 100 110 120 130 140 150 160

....|....|....|....|....|....|....|....|....|....|....|....|....|....|....|....|

**NicholsG**  **ttccccgaagctaactggctctgccacacttgagtggggcatcagctatggcaagggggtaggcagtcatggccaggccc**

**Street14G ttccccgaagctaactggctctgccacacttgagtggggcatcagctatggcaagggggtaggcagtcatggccaggccc**

**Bal3G**  **ttccccgaagctaactggctctgccacacttgagtggggcatcagctatggcaagggggtaggcagtcatggccaggccc**

**MexicoAG**  **ttccccgaagctaactggctctgccacacttgagtggggcatcagctatggcaagggggtaggcagtcatggccaggccc**

**GauthierG** **ttccccgaagctaactggctctgccacacttgagtggggcatcagctatggcaagggggtaggcagtcatggccaggccc**

**CDC2G**  **ttccccgaagctaactggctctgccacacttgagtggggcatcagctatggcaagggggtaggcagtcatggccaggccc**

**Sea81-4G**  **tcccccgaagctaactggctctgccacacttgagtggggcatcagctatggcaagggggtaggcagtcatggccaggccc**

170 180 190 200 210 220 230 240

....|....|....|....|....|....|....|....|....|....|....|....|....|....|....|....|

**NicholsG**  **ctggtgcagttatgggcaccggtccctacaatctgaagcacgggtttcgtactaccaacacggtgggagtatcctttccc**

**Street14G ctggtgcagttatgggcaccggtccctacaatctgaagcacgggtttcgtactaccaacacggtgggagtatcctttccc**

**Bal3G**  **ctggtgcagttatgggcaccggtccctacaatctgaagcacgggtttcgtactaccaacacggtgggagtatcctttccc**

**MexicoAG**  **ctggtgcagttatgggcaccggtccctacaatctgaagcacgggtttcgtactaccaacacggtgggagtatcctttccc**

**GauthierG** **ctggtgcagttatgggcaccggtccctacaatctgaggcacgggtttcgtactaccaacacggtgggagtatcctttccc**

**CDC2G**  **ctggtgcagttatgggcaccggtccctacaatctgaagcacgggtttcgtactaccaacacggtgggagtatcctttccc**

**Sea81-4G**  **ctggtgcagttatgggcaccggtccctacaatctgaagcacgggtttcgtactaccaacacggtgggagtatcctttccc**

250 260 270 280 290 300 310 320

....|....|....|....|....|....|....|....|....|....|....|....|....|....|....|....|

**NicholsG**  **ctggttatgcgcaccacccacacgcgccgtgggcagcacccggcactgtatgcggagctgaaggtggcggacctgcaggc**

**Street14G ctggttatgcgcaccacccacacgcgccgtgggcagcacccggcactgtatgcggagctgaaggtggcggacctgcaggc**

**Bal3G**  **ctggttatgcgcaccacccacacgcgccgtgggcagcacccggcactgtatgcggagctgaaggtggcggacctgcaggc**

**MexicoAG**  **ctggttatgcgcaccacccacacgcgccgtgggcagcacccggcactgtatgcggagctgaaggtggcggacctgcaggc**

**GauthierG** **ctggttatgcgcaccacccacacgcgccgtgggcagcacccggcactgtatgcggagctgaaggtggcggacctgcaggc**

**CDC2G**  **ctggttatgcgcaccacccacacgcgccgtgggcagcacccggcactgtatgcggagctgaaggtggcggacctgcaggc**

**Sea81-4G**  **ctggttatgcgcaccacccacacgcgccgtgggcagcacccggcactgtatgcggagctgaaggtggcggacctgcaggc**

330 340 350 360 370 380 390 400

....|....|....|....|....|....|....|....|....|....|....|....|....|....|....|....|

**NicholsG**  **ggacctgagtcaggggaaggcaggttttgccgttaagcgcaaggggaaggtagaggcgacactacactgttatggggcct**

**Street14G ggacctgagtcaggggaaggcaggttttgccgttaagcgcaaggggaaggtagaggcgacactacactgttatggggcct**

**Bal3G**  **ggacctgagtcaggggaaggcaggttttgccgttaagcgcaaggggaaggtagaggcgacactacactgttatggggcct**

**MexicoAG**  **ggacctgagtcaggggaaggcaggttttgccgttaagcgcaaggggaaggtagaggcgacactacactgttatggggcct**

**GauthierG** **ggacctgagtcaggggaaggcaggttttgccgttaagcgcaaggggaaggtagaggcgacactacactgttatggggcct**

**CDC2G**  **ggacctgagtcaggggaaggcaggttttgccgttaagcgcaaggggaaggtagaggcgacactacactgttatggggcct**

**Sea81-4G**  **ggacctgagtcaggggaaggcaggttttgccgttaagcgcaaggggaaggtagaggcgacactacactgttatggggcct**

410 420 430 440 450 460 470 480

....|....|....|....|....|....|....|....|....|....|....|....|....|....|....|....|

**NicholsG**  **acctgacgattgggaagaaccccacgtttctgacgaactttgcccggctgtggaagccgtgggtgacagcgcagtaccag**

**Street14G acctgacgattgggaagaaccccacgtttctgacgaactttgcccggctgtggaagccgtgggtgacagcgcagtaccag**

**Bal3G**  **acctgacgattgggaagaaccccacgtttctgacgaactttgcccggctgtggaagccgtgggtgacagcgcagtaccag**

**MexicoAG**  **acctgacgattgggaagaaccccacgtttctgacgaactttgcccggctgtggaagccgtgggtgacagcgcagtaccag**

**GauthierG** **acctgacgattgggaagaaccccacgtttctgacgaactttgcccggctgtggaagccgtgggtgacagcgcagtaccag**

**CDC2G**  **acctgacgattgggaagaaccccacgtttctgacgaactttgcccggctgtggaagccgtgggtgacagcgcagtaccag**

**Sea81-4G**  **acctgacgattgggaagaaccccacgtttctgacgaactttgcccggctgtggaagccgtgggtgacagcgcagtaccag**

490 500 510 520 530 540 550 560

....|....|....|....|....|....|....|....|....|....|....|....|....|....|....|....|

**NicholsG**  **gaggatgcggtacagtatgcgccggggtttgggggtttaggcggcaaggttgggtatcgggcacaggacattgggggcag**

**Street14G gaggatgcggtacagtatgcgccggggtttgggggtttaggcggcaaggttgggtatcgggcacaggacattgggggcag**

**Bal3G**  **gaggatgcggtacagtatgcgccggggtttgggggtttaggcggcaaggttgggtatcgggcacaggacattgggggcag**

**MexicoAG**  **gaggatgcggtacagtatgcgccggggtttgggggtttaggcggcaaggttgggtatcgggcacaggacattgggggcag**

**GauthierG** **gaggatgcggtacagtatgcgccggggtttgggggtttaggcggcaaggttgggtatcgggcacaggacattgggggcag**

**CDC2G**  **gaggatgcggtacagtatgcgccggggtttgggggtttaggcggcaaggttgggtatcgggcacaggacattgggggcag**

**Sea81-4G**  **gaggatgcggtacagtatgcgccggggtttgggggtttaggcggcaaggttgggtatcgggcacaggacattgggggcag**

570 580 590 600 610 620 630 640

....|....|....|....|....|....|....|....|....|....|....|....|....|....|....|....|

**NicholsG**  **tggggtcagccttgatgtggggtttctctcctttgcctctaacggtgcctgggatagtactgaccccacgcacagtaagt**

**Street14G tggggtcagccttgatgtggggtttctctcctttgcctctaacggtgcctgggatagtactgaccccacgcacagtaagt**

**Bal3G**  **tggggtcagccttgatgtggggtttctctcctttgcctctaacggtgcctgggatagtactgaccccacgcacagtaagt**

**MexicoAG**  **tggggtcagccttgatgtggggtttctctcctttgcctctaacggtgcctgggatagtactgaccccacgcacagtaagt**

**GauthierG** **tggggtcagccttgatgtggggtttctctcctttgcctctaacggtgcctgggatagtactgaccccacgcacagtaagt**

**CDC2G**  **tggggtcagccttgatgtggggtttctctcctttgcctctaacggtgcctgggatagtactgaccccacgcacagtaagt**

**Sea81-4G**  **tggggtcagccttgatgtggggtttctctcctttgcctctaacggtgcctgggatagtactgaccccacgcacagtaagt**

650 660 670 680 690 700 710 720

....|....|....|....|....|....|....|....|....|....|....|....|....|....|....|....|

**NicholsG**  **atggctttggggcagacttgaagctaatgtatgcgcgtgcaggacaccctctgtgcacggtagagcttgccagcaatgtt**

**Street14G atggctttggggcagacttgaagctaatgtatgcgcgtgcaggacaccctctgtgcacggtagagcttgccagcaatgtt**

**Bal3G**  **atggctttggggcagacttgaagctaatgtatgcgcgtgcaggacaccctctgtgcacggtagagcttgccagcaatgtt**

**MexicoAG**  **atggctttggggcagacttgaagctaatgtatgcgcgtgcaggacaccctctgtgcacggtagagcttgccagcaatgtt**

**GauthierG** **atggctttggggcagacttgaagctaatgtatgcgcgtgcaggacaccctctgtgcacggtagagcttgccagcaatgtt**

**CDC2G**  **atggctttggggcagacttgaagctaatgtatgcgcgtgcaggacaccctctgtgcacggtagagcttgccagcaatgtt**

**Sea81-4G**  **atggctttggggcagacttgaagctaatgtatgcgcgtgcaggacaccctctgtgcacggtagagcttgccagcaatgtt**

730 740 750 760 770 780 790 800

....|....|....|....|....|....|....|....|....|....|....|....|....|....|....|....|

**NicholsG**  **acgctagaagacggatacctcatcggtgcacagaaggacgcaaacaatcagaacaaggataaactgctgtggaatgtagg**

**Street14G acgctagaagacggatacctcatcggtgcacagaaggacgcaaacaatcagaacaaggataaactgctgtggaatgtagg**

**Bal3G**  **acgctagaagacggatacctcatcggtgcacagaaggacgcaaacaatcagaacaaggataaactgctgtggaatgtagg**

**MexicoAG**  **acgctagaagacggatacctcatcggtgcacagaaggacgcaaacaatcagaacaaggataaactgctgtggaatgtagg**

**GauthierG** **acgctagaagacggatacctcatcggtgcacagaaggacgcaaacaatcagaacaaggataaactgctgtggaatgtagg**

**CDC2G**  **acgctagaagacggatacctcatcggtgcacagaaggacgcaaacaatcagaacaaggataaactgctgtggaatgtagg**

**Sea81-4G**  **acgctagaagacggatacctcatcggtgcacagaaggacgcaaacaatcagaacaaggataaactgctgtggaatgtagg**

810 820 830 840 850 860 870 880

....|....|....|....|....|....|....|....|....|....|....|....|....|....|....|....|

**NicholsG**  **gggccgactcaccctcgaaccaggcgccggcttccgcttctccttcgccctcgacgccggtaaccaacaccagagtgcac**

**Street14G gggccgactcaccctcgaaccaggcgccggcttccgcttctccttcgccctcgacgccggtaaccaacaccagagtgcac**

**Bal3G**  **gggccgactcaccctcgaaccaggcgccggcttccgcttctccttcgccctcgacgccggtaaccaacaccagagtgcac**

**MexicoAG**  **gggccgactcaccctcgaaccaggcgccggcttccgcttctccttcgccctcgacgccggtaaccaacaccagagtgcac**

**GauthierG** **gggccgactcaccctcgaaccaggcgccggcttccgcttctccttcgccctcgacgccggcaaccaacaccagagtgcac**

**CDC2G**  **gggccgactcaccctcgaaccaggcgccggcttccgcttctccttcgccctcgacgccggtaaccaacaccagagtgcac**

**Sea81-4G**  **gggccgactcaccctcgaaccaggcgccggcttccgcttctccttcgccctcgacgccggtaaccaacaccagagtgcac**

890 900 910 920 930 940 950 960

....|....|....|....|....|....|....|....|....|....|....|....|....|....|....|....|

**NicholsG**  **aggactttcaaaatcgcacacagagggcgcagagtgaactcaccgccctctcaaataacctcttccagggagaaagtcaa**

**Street14G aggactttcaaaatcgcacacagagggcgcagagtgaactcaccgccctctcaaataacctcttccagggagaaagtcaa**

**Bal3G**  **aggactttcaaaatcgcacacagagggcgcagagtgaactcaccgccctctcaaataacctcttccagggagaaagtcaa**

**MexicoAG**  **aggactttcaaaatcgcacacagagggcgcagagtgaactcaccgccctctcaaataacctcttccagggagaaagtcaa**

**GauthierG** **aggactttcaaaatcgcacacagagggcgcagagtgaactcaccgccctctcaaataacctcttccagggagaaagtcaa**

**CDC2G**  **aggactttcaaaatcgcacacagagggcgcagagtgaactcaccgccctctcaaataacctcttccagggagaaagtcaa**

**Sea81-4G**  **aggactttcaaaatcgcacacagagggcgcagagtgaactcaccgccctctcaaataacctcttccagggagaaagtcaa**

970 980 990 1000 1010 1020 1030 1040

....|....|....|....|....|....|....|....|....|....|....|....|....|....|....|....|

**NicholsG**  **aaacaggaagcctggctggacgaatatgcaaagaaggtgcttgatgccgtaacggcagccaccgaaaccgcccttcagtc**

**Street14G aaacaggaagcctggctggacgaatatgcaaagaaggtgcttgatgccgtaacggcagccaccgaaaccgcccttcagtc**

**Bal3G**  **aaacaggaagcctggctggacgaatatgcaaagaaggtgcttgatgccgtaacggcagccaccgaaaccgcccttcagtc**

**MexicoAG**  **aaacaggaagcctggctggacgaatatgcaaagaaggtgcttgatgccgtaacggcagccaccgaaaccgcccttcagtc**

**GauthierG** **aaacaggaagcctggctggacgaatatgcaaagaaggtgcttgatgccgtaacggcagccaccgaaaccgcccttcagtc**

**CDC2G**  **aaacaggaagcctggctggacgaatatgcaaagaaggtgcttgatgccgtaacggcagccaccgaaaccgcccttcagtc**

**Sea81-4G**  **aaacaggaagcctggctggacgaatatgcaaagaaggtgcttgatgccgtaacggcagccaccgaaaccgcccttcagtc**

1050 1060 1070 1080 1090 1100 1110 1120

....|....|....|....|....|....|....|....|....|....|....|....|....|....|....|....|

**NicholsG**  **gaggggaaacgcgtacataacggcagtgtcaaacgtaaaagtcacccctccggtagctgccacgcttttgacgaacctga**

**Street14G gaggggaaacgcgtacataacggcagtgtcaaacgtaaaagtcacccctccggtagctgccacgcttttgacgaacctga**

**Bal3G**  **gaggggaaacgcgtacataacggcagtgtcaaacgtaaaagtcacccctccggtagctgccacgcttttgacgaacctga**

**MexicoAG**  **gaggggaaacgcgtacataacggcagtgtcaaacgtaaaagtcacccctccggtagctgccacgcttttgacgaacctga**

**GauthierG** **gaggggaaacgcgtacataacggcagtgtcaaacgtaaaagtcacccctccggtagctgccacgcttttgacgaacctga**

**CDC2G**  **gaggggaaacgcgtacataacggcagtgtcaaacgtaaaagtcacccctccggtagctgccacgcttttgacgaacctga**

**Sea81-4G**  **gaggggaaacgcgtacataacggcagtgtcaaacgtaaaagtcacccctccggtagctgccacgcttttgacgaacctga**

1130 1140 1150 1160 1170 1180 1190 1200

....|....|....|....|....|....|....|....|....|....|....|....|....|....|....|....|

**NicholsG**  **aggtgttcattaccgaccctcctacaccgtcaccgcttcccgcgcttcctgcattttccctgatggggcaggttttgctg**

**Street14G aggtgttcattaccgaccctcctacaccgtcaccgcttcccgcgcttcctgcattttccctgatggggcaggttttgctg**

**Bal3G**  **aggtgttcattaccgaccctcctacaccgtcaccgcttcccgcgcttcctgcattttccctgatggggcaggttttgccg**

**MexicoAG**  **aggtgttcattaccgaccctcctacaccgtcaccgcttcccgcgcttcctgcattttccctgatggggcaggttttgctg**

**GauthierG** **aggtgttcattaccgaccctcctacaccgtcaccgcttcccgcgcttcctgcattttccctgatggggcaggttttgctg**

**CDC2G**  **aggtgttcattaccgaccctcctacaccgtcaccgcttcccgcgcttcctgcattttccctgatggggcaggttttgctg**

**Sea81-4G**  **aggtgttcattaccgaccctcctacaccgtcaccgcttcccgcgcttcctgcattttccctgatggggcaggttttgctg**

1210 1220 1230 1240 1250 1260 1270 1280

....|....|....|....|....|....|....|....|....|....|....|....|....|....|....|....|

**NicholsG**  **cagtacgatgcggagcaggtggtgaaggggtttgagcaggtacagacgcaaatcgttgctgaaattaaccagaaagtgca**

**Street14G cagtacgatgcggagcaggtggtgaaggggtttgagcaggtacagacgcaaatcgttgctgaaattaaccagaaagtgca**

**Bal3G**  **cagtacgatgcggagcaggtggtgaaggggtttgagcaggtacagacgcaaatcgttgctgaaattaaccagaaagtgca**

**MexicoAG**  **cagtacgatgcggagcaggtggtgaaggggtttgagcaggtacagacgcaaatcgttgctgaaattaaccagaaagtgca**

**GauthierG** **cagtacgatgcggagcaggtggtgaaggggtttgagcaggtacagacgcaaatcgttgctgaaattaaccagaaagtgca**

**CDC2G**  **cagtacgatgcggagcaggtggtgaaggggtttgagcaggtacagacgcaaatcgttgctgaaattaaccagaaagtgca**

**Sea81-4G**  **cagtacgatgcggagcaggtggtgaaggggtttgagcaggtacagacgcaaatcgttgctgaaattaaccagaaagtgca**

1290 1300 1310 1320 1330 1340 1350 1360

....|....|....|....|....|....|....|....|....|....|....|....|....|....|....|....|

**NicholsG**  **agcggctgtggctcagagcaaggctgcagcacaggcattcatcaacggtcttaccaaggcaatagaagacgtggctgatg**

**Street14G agcggctgtggctcagagcaaggctgcagcacaggcattcatcaacggtcttaccaaggtaatagaagacgtggctgatg**

**Bal3G**  **agcggctgtggctcagagcaaggctgcagcacaggcattcatcaacggtcttaccaaggcaatagaagacgtggctgatg**

**MexicoAG**  **agcggctgtggctcagagcaaggctgcagcacaggcattcatcaacggtcttaccaaggtaatagaagacgtggctgatg**

**GauthierG** **agcggctgtggctcagagcaaggctgcagcacaggcattcatcaacggtcttaccaaggcaatagaagacgtggctgatg**

**CDC2G**  **agcggctgtggctcagagcaaggctgcagcacaggcattcatcaacggtcttaccaaggcaatagaagacgtggctgatg**

**Sea81-4G**  **agcggctgtggctcagagcaaggctgcagcacaggcattcatcaacggtcttaccaaggcaatagaagacgtggctgatg**

1370 1380 1390 1400 1410 1420 1430 1440

....|....|....|....|....|....|....|....|....|....|....|....|....|....|....|....|

**NicholsG**  **cgttgcttgcaccgcataagggaaatccgatgagcctcttcaaccttccggatcaacaaaaattactgaaggacgatctc**

**Street14G cgttgcttgcaccgcataagggaaatccgatgagcctcttcaaccttccggatcaacaaaaattactgaaggacgatctc**

**Bal3G**  **cgttgcttgcaccgcataagggaaatccgatgagcctcttcaaccttccggatcaacaaaaattactgaaggacgatctc**

**MexicoAG**  **cgttgcttgcaccgcataagggaaatccgatgagcctcttcaaccttccggatcaacaaaaattactgaaggacgatctc**

**GauthierG** **cgttgcttgcaccgcataagggaaatccgatgagcctcttcaaccttccggatcaacaaaaattactgaaggacgatctc**

**CDC2G**  **cgttgcttgcaccgcataagggaaatccgatgagcctcttcaaccttccggatcaacaaaaattactgaaggacgatctc**

**Sea81-4G**  **cgttgcttgcaccgcataagggaaatccgatgagcctcttcaaccttccggatcaacaaaaattactgaaggacgatctc**

1450 1460 1470 1480 1490 1500 1510 1520

....|....|....|....|....|....|....|....|....|....|....|....|....|....|....|....|

**NicholsG**  **gccgatcttattccaaagcttacggctgaggctacaaagtttttcactgagggtcagacgtttgtaaccgaagaagtgaa**

**Street14G gccgatcttattccaaagcttacggctgaggctacaaagtttttcactgagggtcagacgtttgtaaccgaagaagtgaa**

**Bal3G**  **gccgatcttattccaaagcttacggctgaggctacaaagtttttcactgagggtcagacgtttgtaaccgaagaagtgaa**

**MexicoAG**  **gccgatcttattccaaagcttacggctgaggctacaaagtttttcactgagggtcagacgtttgtaaccgaagaagtgaa**

**GauthierG** **gccgatcttattccaaagcttacggctgaggctacaaagtttttcactgagggtcagacgtttgtaaccgaagaagtgaa**

**CDC2G**  **gccgatcttattccaaagcttacggctgaggctacaaagtttttcactgagggtcagacgtttgtaaccgaagaagtgaa**

**Sea81-4G**  **gccgatcttattccaaagcttacggctgaggctacaaagtttttcactgagggtcagacgtttgtaaccgaagaagtgaa**

1530 1540 1550 1560 1570 1580 1590 1600

....|....|....|....|....|....|....|....|....|....|....|....|....|....|....|....|

**NicholsG**  **gaagaagacggatgcgttggacgcggggcagcagatacgtcaggctatacagaacctgcgtgcgtctgcatggcgtgcct**

**Street14G gaagaagacggatgcgttggacgcggggcagcagatacgtcaggctatacagaacctgcgtgcgtctgcatggcgtgcct**

**Bal3G**  **gaagaagacggatgcgttggacgcggggcagcagatacgtcaggctatacagaacctgcgtgcgtctgcatggcgtgcct**

**MexicoAG**  **gaagaagacggatgcgttggacgcggggcagcagatacgtcaggctatacagaacctgcgtgcgtctgcatggcgtgcct**

**GauthierG** **gaagaagacggatgcgttggacgcggggcagcagatacgtcaggctatacagaacctgcgtgcgtctgcatggcgtgcct**

**CDC2G**  **gaagaagacggatgcgttggacgcggggcagcagatacgtcaggctatacagaacctgcgtgcgtctgcatggcgtgcct**

**Sea81-4G**  **gaagaagacggatgcgttggacgcggggcagcagatacgtcaggctatacagaacctgcgtgcgtctgcatggcgtgcct**

1610 1620 1630 1640 1650 1660 1670 1680

....|....|....|....|....|....|....|....|....|....|....|....|....|....|....|....|

**NicholsG**  **ttctaatgggagtcagcgccgtgtgtctgtatcttgacacctacaatgtcgccttcgatgcgctgtttacggcgcagtgg**

**Street14G ttctaatgggagtcagcgccgtgtgtctgtatcttgacacctacaatgtcgccttcgatgcgctgtttacggcgcagtgg**

**Bal3G**  **ttctaatgggagtcagcgccgtgtgtctgtatcttgacacctacaatgtcgccttcgatgcgctgtttacggcgcagtgg**

**MexicoAG**  **ttctaatgggagtcagcgccgtgtgtctgtatcttgacacctacaatgtcgccttcgatgcgctgtttacggcgcagtgg**

**GauthierG** **ttctaatgggagtcagcgccgtgtgtctgtatcttgacacctacaatgtcgccttcgatgcactgcttacagcgcagtgg**

**CDC2G**  **ttctaatgggagtcagcgccgtgtgtctgtatcttgacacctacaatgtcgccttcgatgcactgcttacagcgcagtgg**

**Sea81-4G**  **ttctaatgggagtcagcgccgtgtgtctgtatcttgacacctacaatgtcgccttcgatgcgctgtttacggcgcagtgg**

1690 1700 1710 1720 1730 1740 1750 1760

....|....|....|....|....|....|....|....|....|....|....|....|....|....|....|....|

**NicholsG**  **aagtggctgtcttctggcatatactttgccacagcaccggcaaacgtttttggcaccagggtgttagataacaccatcgc**

**Street14G aagtggctgtcttctggcatatactttgccacagcaccggcaaacgtttttggcaccagggtgttagataacaccatcgc**

**Bal3G**  **aagtggctgtcttctggcatatactttgccacagcaccggcaaacgtttttggcaccagggtgttagataacaccatcgc**

**MexicoAG**  **aagtggctgtcttctggcatatacgttgccacagcaccggcaaacgtttttggcaccagggtgttagataacaccatcgc**

**GauthierG** **aagtggctgtcttctggcatatactttgccacagcaccggcaaacgtttttggcaccagggtgttagataacaccatcgc**

**CDC2G**  **aagtggctgtcttctggcatatactttgccacagcaccggcaaacgtttttggcaccagggtgttagataacaccatcgc**

**Sea81-4G**  **aagtggctgtcttctggcatatacgttgccacagcaccggcaaacgtttttggcaccagggtgttagataacaccatcgc**

1770 1780 1790 1800 1810 1820 1830 1840

....|....|....|....|....|....|....|....|....|....|....|....|....|....|....|....|

**NicholsG**  **aagctgtggcgactttgccggattccttaagctcgaaactaagagcggtgacccctacacccacctgctcaccggcctgg**

**Street14G aagctgtggcgactttgccggattccttaagctcgaaactaagagcggtgacccctacacccacctgctcaccggcctgg**

**Bal3G**  **aagctgtggcgactttgccggattccttaagctcgaaactaagagcggtgacccctacacccacctgctcaccggcctgg**

**MexicoAG**  **aagctgtggcgactttgccggattccttaagctcgaaactaagagcggtgacccctacacccacctgctcaccggcctgg**

**GauthierG** **aagctgtggcgactttgccggattcctcaaactcgaaactaagagcggtgacccctacacccacctgctcaccggcctgg**

**CDC2G**  **aagctgtggcgactttgccggattcctcaaactcgaaactaagagcggtgacccctacacccacctgctcaccggcctgg**

**Sea81-4G**  **aagctgtggcgactttgccggattccttaagctcgaaactaagagcggtgacccctacacccacctgctcaccggcctgg**

1850 1860 1870 1880 1890 1900 1910 1920

....|....|....|....|....|....|....|....|....|....|....|....|....|....|....|....|

**NicholsG**  **acgccggcgttgaaacacgcgtgtacatccccctcacctatgcg-ctatacaaaaataacgggggga-------------**

**Street14G acgccggcgttgaaacacgcgtgtacatccccctcacctatgcg-ctatacaaaaataacgggggga-------------**

**Bal3G**  **acgccggcgttgaaacacgcgtgtacatccccctcacccatgac-ctgtacaaaaataataacgggaa---ccctctccc**

**MexicoAG**  **acgccggcgttgaaacacgcgtgtacatccccctcacccatgac-ctgtacaaaaataataacgggaa---ccctctccc**

**GauthierG** **acgccggcgttgaaacacgcgtgtacatccccctcacccatgac-ctgtacaaaaataataacgggaa---ccctctccc**

**CDC2G**  **acgccggcgttgaaacacgcgtgtacatccccctcacccatgac-ctgtacaaaaataataacgggaa---ccctctccc**

**Sea81-4G**  **acgccggcgttgaaacacgcgtgtacatccccctcacccatgacgctatacaaaaataataacgggggaaccgctctccc**

1930 1940 1950 1960 1970 1980 1990 2000

....|....|....|....|....|....|....|....|....|....|....|....|....|....|....|....|

**NicholsG**  **--cggctgtgcgtggcattcaggaaaaggagtata-tccgtccaccggtggtggggaaggcgtggtgtagctatcgcatc**

**Street14G --cggctgtgcgtggcattcaggaaaaggagtata-tccgtccaccggtggtggggaaggcgtggtgtagctatcgcatc**

**Bal3G**  **ttccggcg-----gttcctcagggcaca--------ttggcctgccggtggtggggaaggcgtggtgtagctatcgcatc**

**MexicoAG**  **ttccggcg-----gttcctcagggcaca--------ttggcctgccggtggtggggaaggcgtggtgtagctatcgcatc**

**GauthierG** **ttccggcg-----gttcctcagggcaca--------ttggcctgccggtggtggggaaggcgtggtgtagctatcgcatc**

**CDC2G**  **ttccggcg-----gttcctcagggcaca--------ttggcctgccggtggtggggaaggcgtggtgtagctatcgcatc**

**Sea81-4G**  **ttccggca-----gttcctcaggaaaagcactatatccggccagccggtggtggggaaggcgtggtgtagctatcgcatc**

2010 2020 2030 2040 2050 2060 2070 2080

....|....|....|....|....|....|....|....|....|....|....|....|....|....|....|....|

**NicholsG**  **ccggtgcaggattacggctgggtgaagccaagcgttacggtccatgcctctaccaaccgtgcacacctgaatgcccctgc**

**Street14G ccggtgcaggattacggctgggtgaagccaagcgttacggtccatgcctctaccaaccgtgcacacctgaatgcccctgc**

**Bal3G**  **ccggtgcaggattacggctgggtgaagccaagcgttacggtccatgcctctaccaaccgtgcacacctgaatgcccctgc**

**MexicoAG**  **ccggtgcaggattacggctgggtgaagccaagcgttacggtccatgcctctaccaaccgtgcacacctgaatgcccctgc**

**GauthierG** **ccggtgcaggattacggctgggtgaagccaagcgttacggtccatgcctctaccaaccgtgcacacctgaatgcccctgc**

**CDC2G**  **ccggtgcaggattacggctgggtgaagccaagcgttacggtccatgcctctaccaaccgtgcacacctgaatgcccctgc**

**Sea81-4G**  **ccggtgcaggattacggctgggtgaagccaagcgttacggtccatgcctctaccaaccgtgcacacctgaatgcccctgc**

2090 2100 2110 2120 2130 2140 2150 2160

....|....|....|....|....|....|....|....|....|....|....|....|....|....|....|....|

**NicholsG**  **tgcaggtggagcagtaggagctacctatctaaccaaggagtactgtgcacagctgcgtgctggtatttcagccagtctca**

**Street14G tgcaggtggagcagtaggagctacctatctaaccaaggagtactgtgcacagctgcgtgctggtatttcagccagtctca**

**Bal3G**  **tgcaggtggagcagtaggagctacctatctaaccaaggagtactgtgcacagctgcgtgctggtatttcagccagtctca**

**MexicoAG**  **tgcaggtggagcagtaggagctacctatctaactaaggagtactgtgcacagctgcgtgctggtgtttcagccagtctca**

**GauthierG** **tgcaggtggagcagtaggagctacctatctaactaaggagtactgtgcacagctgcgtgctggtgtttcagccagtctca**

**CDC2G**  **tgcaggtggagcagtaggagctacctatctaactaaggagtactgtgcacagctgcgtgctggtgtttcagccagtctca**

**Sea81-4G**  **tgcaggtggagcagtaggagctacctatctaaccaaggagtactgtgcacagctgcgtgctggtatttcagccagtctca**

2170 2180 2190 2200 2210 2220 2230 2240

....|....|....|....|....|....|....|....|....|....|....|....|....|....|....|....|

**NicholsG**  **tagagaagacggtattctcccttgattgggaacagggtatgctctctgatgtcccgtacctgctggtgtccgagtgcctc**

**Street14G tagagaagacggtattctcccttgattgggaacagggtatgctctctgatgtcccgtacctgctggtgtccgagtgcctc**

**Bal3G**  **tagagaagacggtattctcccttgattgggaacagggtatgctctctgatgtcccgtacctgctggtgtccgagtgcctc**

**MexicoAG**  **tagagaagacggtattctcccttgattgggaacagggtatgctctctgatgtcccgtacctgctggtgtccgagtgcctc**

**GauthierG** **tagagaagacggtattctcccttgattgggaacagggtatgctctctgatgtcccgtacctgctggtgtccgagtgcctc**

**CDC2G**  **tagagaagacggtattctcccttgattgggaacagggtatgctctctgatgtcccgtacctgctggtgtccgagtgcctc**

**Sea81-4G**  **tagagaagacggtattctcccttgattgggaacagggtatgctctctgatgtcccgtacctgctggtgtccgagtgcctc**

2250 2260 2270 2280

....|....|....|....|....|....|....|....|....|...

**NicholsG**  **acccagggaatcggccgcatcgtgtgcggcgtcaccctctcctggtag**

**Street14G acccagggaatcggccgcatcgtgtgcggcgtcaccctctcctggtag**

**Bal3G**  **acccagggaatcggccgcatcgtgtgcggcgtcaccctctcctggtag**

**MexicoAG**  **acccagggaatcggccgcatcgtgtgcggcgtcaccctctcctggtag**

**GauthierG** **acccagggaatcggccgcatcgtgtgcggcgtcaccctctcctggtgg**

**CDC2G**  **acccagggaatcggccgcatcgtgtgcggcgtcaccctctcctggtag**

**Sea81-4G**  **acccagggaatcggccgcatcgtgtgcggcgtcaccctctcctggtag**

***1.6.2 tprG* locus containing *tprGI* chimeras**

10 20 30 40 50 60 70 80

....|....|....|....|....|....|....|....|....|....|....|....|....|....|....|....|

**BosniaAG**  **MGCMRWGSVLCVVVGVGASGGVLGQEFSPKLTGSATLEWGISYGEGVGSYGQAPGAVMGTGPYNLKHGFRTTNTVGVSFP**

**IraqBG**  **MGCMRWGSVLCVVVGVGASGGVLGQEFSPKLTGSATLEWGISYGEGVGSYGQAPGAVMGTGPYNLKHGFRTTNTVGVSFP**

**Fribourg-BlancG** **MGCMRWGSVLCVVVGVGASGGVLGQEFSPKLTGSATLEWGISYGKGVGSHDQAPGAVMGTGPYNLKHGFRTTNTVGVSFP**

**CuniculiAG**  **MGCMRWGSVLCVVVGVGASGGVLGQEFSPKLTGSATLEWGISYGKGVGSHDQAPGAVMGTGPYNLKHGFRTTNTVGVSFP**

90 100 110 120 130 140 150 160

....|....|....|....|....|....|....|....|....|....|....|....|....|....|....|....|

**BosniaAG**  **LVMRTTHTRRGQHPALYAELKVADLQADLSQGKVGFAVKRKGKVEATLHCYGAYLTIGKNPTFLTNFARLWKPWVTAQYQ**

**IraqBG**  **LVMRTTHTRRGQHPALYAELKVADLQADLSQGKVGFAVKRKGKVEATLHCYGAYLTIGKNPTFLTNFARLWKPWVTAQYQ**

**Fribourg-BlancG** **LVMRTTHTRRGQHPALYAELKVADLQADLSQGKAGFDFKRKGKVEATLHCYGAYLTIGKNPTFLTNFARLWKPWVTAQYQ**

**CuniculiAG**  **LVMRTTHTRRGQHPALYAELKVADLQADLSQGKAGFDFKRKGKVEATLHCYGAYLTIGKNPTFLTNFARLWKPWVTAQYQ**

170 180 190 200 210 220 230 240

....|....|....|....|....|....|....|....|....|....|....|....|....|....|....|....|

**BosniaAG**  **EDAVQYAPGFGGLGGKVGYRAQDIGGSGVSLDVGFLSFASNGAWDSTDPTHSKYGFGADLKLMYARAGHPLCTVELASNV**

**IraqBG**  **EDAVQYAPGFGGLGGKVGYRAQDIGGSGVSLDVGFLSFASNGAWDSTDPTHSKYGFGADLKLMYARAGHPLCTVELASNV**

**Fribourg-BlancG** **EDEVQYAPGFGGLGGKVGYRAQDIGGSGVSLDVGFLSFASNGAWDSTDTTHSKYGFGGRLEANVCACRTPSVHGRACQQC**

**CuniculiAG**  **EDEVQYAPGFGGLGGKVGYRAQDIGGSGVSLDVGFLSFASNGAWDSTDTTHSKYGFGGRLEANVCACRTPSVHGRACQQC**

250 260 270 280 290 300 310 320

....|....|....|....|....|....|....|....|....|....|....|....|....|....|....|....|

**BosniaAG**  **TLEDGYLIGAQKDANNQNKDKLLWNVGGRLTLEPGAGFRFSFALDAGNQHQSNADADCRLPATGSSREKFDRAFDALRVE**

**IraqBG**  **TLEDGYLIGAQKDANNQNKDKLLWNVGGRLTLEPGAGFRFSFALDAGNQHQSNADADCRLPATGSSREKFDRAFDALRVE**

**Fribourg-BlancG** **YAGRRIPHRCAEGRKQSEQG**

**CuniculiAG**  **YAGRRIPHRCAEGRKQSEQG**

330 340 350 360 370 380 390 400

....|....|....|....|....|....|....|....|....|....|....|....|....|....|....|....|

**BosniaAG**  **QYRVKDKYLEFLLGQMAESSILERVGLALTLQDGTLVSTLTKVATDSGDRFIQMALVKLLPQRAQAEQGLREIVAPSQSD**

**IraqBG**  **QYRVKDKYLEFLLGQMAESSILERVGLALTLQDGTLVSTLTKVATDSGDRFIQMALVKLLPQRAQAEQGLREIVAPSQSD**

**Fribourg-BlancG**

**CuniculiAG**

410 420 430 440 450 460 470 480

....|....|....|....|....|....|....|....|....|....|....|....|....|....|....|....|

**BosniaAG**  **IVLIMLLTWLERARLDRFNADALLTAQWTYVSAGLYGATAGTNVFGKRVLPALQSWHFDFAGFLKLETKSGDPYTHLLTG**

**IraqBG**  **IVLIMLLTWLERARLDRFNADALLTAQWTYVSAGLYGATAGTNVFGKRVLPALQSWHFDFAGFLKLETKSGDPYTHLLTG**

**Fribourg-BlancG**

**CuniculiAG**

490 500 510 520 530 540 550 560

....|....|....|....|....|....|....|....|....|....|....|....|....|....|....|....|

**BosniaAG**  **LNAGVEARVYIPLTYIRYRNNGGYPLNGVVPPGTINMPILGKAWCSYRIPLGSHAWLTPHTSVLGTTNRFNVINPAYTLL**

**IraqBG**  **LNAGVEARVYIPLTYIRYRNNGGYPLNGVVPPGTINMPILGKAWCSYRIPLGSHAWLTPHTSVLGTTNRFNVINPAYTLL**

**Fribourg-BlancG**

**CuniculiAG**

570 580 590

....|....|....|....|....|....|.

**BosniaAG**  **NERALQYQVGLTFSPFEKVELSAQWEQGVLA**

**IraqBG**  **NERALQYQVGLTFSPFEKVELSAQWEQGVLA**

**Fribourg-BlancG**

**CuniculiAG**

10 20 30 40 50 60 70 80

....|....|....|....|....|....|....|....|....|....|....|....|....|....|....|....|

**BosniaAG**  **atggggtgcatgcggtgggggagtgtgctgtgtgtggtggtgggggtaggagcgagcgggggagtgctcggacaggagtt**

**IraqBG**  **atggggtgcatgcggtgggggagtgtgctgtgtgtggtggtgggggtaggagcgagcgggggagtgctcggacaggagtt**

**Fribourg-BlancG** **atggggtgcatgcggtgggggagtgtgctgtgtgtggtggtgggggtaggagcgagcgggggagtgcttggacaggagtt**

**CuniculiAG**  **atggggtgcatgcggtgggggagtgtgctgtgtgtggtggtgggggtaggagcgagcgggggagtgcttggacaggagtt**

90 100 110 120 130 140 150 160

....|....|....|....|....|....|....|....|....|....|....|....|....|....|....|....|

**BosniaAG**  **ttccccgaagctaactggctctgccacacttgagtggggcatcagctatggcgagggggtaggcagttatggccaggccc**

**IraqBG**  **ttccccgaagctaactggctctgccacacttgagtggggcatcagctatggcgagggggtaggcagttatggccaggccc**

**Fribourg-BlancG** **ttccccgaagctaactggctctgccacacttgagtggggcatcagctatggcaagggggtaggcagtcacgaccaggccc**

**CuniculiAG**  **ttccccgaagctaactggctctgccacacttgagtggggcatcagctatggcaagggggtaggcagtcacgaccaggccc**

170 180 190 200 210 220 230 240

....|....|....|....|....|....|....|....|....|....|....|....|....|....|....|....|

**BosniaAG**  **ctggtgcagttatgggcaccggtccctacaatctgaagcacgggtttcgtactaccaacacggtgggagtatcctttccc**

**IraqBG**  **ctggtgcagttatgggcaccggtccctacaatctgaagcacgggtttcgtactaccaacacggtgggagtatcctttccc**

**Fribourg-BlancG** **ctggtgcagttatgggcaccggtccctacaatctgaagcatgggtttcgtactaccaacacggtgggagtatcctttccc**

**CuniculiAG**  **ctggtgcagttatgggcaccggtccctacaatctgaagcatgggtttcgtactaccaacacggtgggagtatcctttccc**

250 260 270 280 290 300 310 320

....|....|....|....|....|....|....|....|....|....|....|....|....|....|....|....|

**BosniaAG**  **ctggttatgcgcaccacccacacgcgccgtgggcagcacccggcactgtatgcggagctgaaggtggcggacctgcaggc**

**IraqBG**  **ctggttatgcgcaccacccacacgcgccgtgggcagcacccggcactgtatgcggagctgaaggtggcggacctgcaggc**

**Fribourg-BlancG** **ctggttatgcgcaccacccacacgcgccgtgggcagcacccggcactgtatgcggagctgaaggtagcggacctgcaggc**

**CuniculiAG**  **ctggttatgcgcaccacccacacgcgccgtgggcagcacccggcactgtatgcggagctgaaggtagcggacctgcaggc**

330 340 350 360 370 380 390 400

....|....|....|....|....|....|....|....|....|....|....|....|....|....|....|....|

**BosniaAG**  **ggacctgagtcaggggaaggtaggttttgccgttaagcgcaaggggaaggtagaggcgacactacactgttatggggcct**

**IraqBG**  **ggacctgagtcaggggaaggtaggttttgccgttaagcgcaaggggaaggtagaggcgacactacactgttatggggcct**

**Fribourg-BlancG** **ggacctgagtcaggggaaggcaggttttgactttaagcgcaaggggaaggtggaggcgacgctgcactgttatggggcct**

**CuniculiAG**  **ggacctgagtcaggggaaggcaggttttgactttaagcgcaaggggaaggtggaggcgacgctgcactgttatggggcct**

410 420 430 440 450 460 470 480

....|....|....|....|....|....|....|....|....|....|....|....|....|....|....|....|

**BosniaAG**  **acctgacgattgggaagaaccccacgtttctgacgaactttgcccggctgtggaagccgtgggtgacagcgcagtaccag**

**IraqBG**  **acctgacgattgggaagaaccccacgtttctgacgaactttgcccggctgtggaagccgtgggtgacagcgcagtaccag**

**Fribourg-BlancG** **acctgacgattgggaagaaccccacgtttctgacgaactttgcccggctgtggaagccgtgggtgacagcgcagtaccag**

**CuniculiAG**  **acctgacgattgggaagaaccccacgtttctgacgaactttgcccggctgtggaagccgtgggtgacagcgcagtaccag**

490 500 510 520 530 540 550 560

....|....|....|....|....|....|....|....|....|....|....|....|....|....|....|....|

**BosniaAG**  **gaggatgcggtacagtatgcgccggggtttgggggtttaggcggcaaggttgggtatcgggcacaggacattgggggcag**

**IraqBG**  **gaggatgcggtacagtatgcgccggggtttgggggtttaggcggcaaggttgggtatcgggcacaggacattgggggcag**

**Fribourg-BlancG** **gaggatgaggtacagtatgcgccggggtttgggggtttaggcggcaaggttgggtatcgagcacaggacattgggggcag**

**CuniculiAG**  **gaggatgaggtacagtatgcgccggggtttgggggtttaggcggcaaggttgggtatcgagcacaggacattgggggcag**

570 580 590 600 610 620 630 640

....|....|....|....|....|....|....|....|....|....|....|....|....|....|....|....|

**BosniaAG**  **tggggtcagccttgatgtggggtttctctcctttgcctctaacggtgcctgggatagtactgaccccacgcacagtaagt**

**IraqBG**  **tggggtcagccttgatgtggggtttctctcctttgcctctaacggtgcctgggatagtactgaccccacgcacagtaagt**

**Fribourg-BlancG** **tggggtcagccttgatgtggggtttctctcctttgcctctaacggtgcctgggatagtactgacaccacgcacagcaagt**

**CuniculiAG**  **tggggtcagccttgatgtggggtttctctcctttgcctctaacggtgcctgggatagtactgacaccacgcacagcaagt**

650 660 670 680 690 700 710 720

....|....|....|....|....|....|....|....|....|....|....|....|....|....|....|....|

**BosniaAG**  **atggctttgggg-cagacttgaagctaatgtatgcgcgtgcaggacaccctctgtgcacggtagagcttgccagcaatgt**

**IraqBG**  **atggctttgggg-cagacttgaagctaatgtatgcgcgtgcaggacaccctctgtgcacggtagagcttgccagcaatgt**

**Fribourg-BlancG** **atggctttgggggcagacttgaagctaatgtatgcgcgtgcaggacaccctctgtgcacggtagagcttgccagcaatgt**

**CuniculiAG**  **atggctttgggggcagacttgaagctaatgtatgcgcgtgcaggacaccctctgtgcacggtagagcttgccagcaatgt**

730 740 750 760 770 780 790 800

....|....|....|....|....|....|....|....|....|....|....|....|....|....|....|....|

**BosniaAG**  **tacgctagaagacggatacctcatcggtgcacagaaggacgcaaacaatcagaacaaggataaactgctgtggaatgtag**

**IraqBG**  **tacgctagaagacggatacctcatcggtgcacagaaggacgcaaacaatcagaacaaggataaactgctgtggaatgtag**

**Fribourg-BlancG** **tacgctggaagacggatacctcatcggtgcgcagaaggacgcaaacaatcagaacaaggataaactgctgtggaatgtag**

**CuniculiAG**  **tacgctggaagacggatacctcatcggtgcgcagaaggacgcaaacaatcagaacaaggataaactgctgtggaatgtag**

810 820 830 840 850 860 870 880

....|....|....|....|....|....|....|....|....|....|....|....|....|....|....|....|

**BosniaAG**  **gaggccgactcaccctcgaaccaggcgccggcttccgcttctccttcgccctcgacgccggtaaccaacaccagagtaac**

**IraqBG**  **gaggccgactcaccctcgaaccaggcgccggcttccgcttctccttcgccctcgacgccggtaaccaacaccagagtaac**

**Fribourg-BlancG** **gaggccgactcaccctcgaaccaggcgccggcttccgcttctccttcgccctcgacgccggtaaccaacaccaggaccct**

**CuniculiAG**  **gaggccgactcaccctcgaaccaggcgccggcttccgcttctccttcgccctcgacgccggtaaccaacaccaggaccct**

890 900 910 920 930 940 950 960

....|....|....|....|....|....|....|....|....|....|....|....|....|....|....|....|

**BosniaAG**  **gcagatgcagactgtcgccttccggcaacggggagctcacgggagaagtttgacagggcgttcgatgccctcagggtgga**

**IraqBG**  **gcagatgcagactgtcgccttccggcaacggggagctcacgggagaagtttgacagggcgttcgatgccctcagggtgga**

**Fribourg-BlancG** **gccggtgcaggcggtcgccttctggcaacagggagctcgcgggagaagtttggcaaggcgttcgatgccctcagggtgca**

**CuniculiAG**  **gccggtgcaggcggtcgccttctggcaacagggagctcgcgggagaagtttggcaaggcgttcgatgccctcagggtgca**

970 980 990 1000 1010 1020 1030 1040

....|....|....|....|....|....|....|....|....|....|....|....|....|....|....|....|

**BosniaAG**  **gcaataccgtgtaaagga---taagtatcttgaatttttgctgggacagatggcggagtcctcgattctcgagcgggtgg**

**IraqBG**  **gcaataccgtgtaaagga---taagtatcttgaatttttgctgggacagatggcggagtcctcgattctcgagcgggtgg**

**Fribourg-BlancG** **gcaattccacgtgaaagaggccgagcatcttgaatttttgctgggacagatggtggagtcctcgattctcgagcgggtgg**

**CuniculiAG**  **gcaattccacgtgaaagaggccgagcatcttgaatttttgctgggacagatggtggagtcctcgattctcgagcgggtgg**

1050 1060 1070 1080 1090 1100 1110 1120

....|....|....|....|....|....|....|....|....|....|....|....|....|....|....|....|

**BosniaAG**  **ggcttgccctcacgctgcaggacggtacgctcgtctctacgctgacgaaggttgccactgatagtggagatcggtttatc**

**IraqBG**  **ggcttgccctcacgctgcaggacggtacgctcgtctctacgctgacgaaggttgccactgatagtggagatcggtttatc**

**Fribourg-BlancG** **ggcttgctctcacgctgcaggacggtacgctcgtccctacgctgacgaaggttgccactgatagtggggatcagttcatc**

**CuniculiAG**  **ggcttgctctcacgctgcaggacggtacgctcgtccctacgctgacgaaggttgccactgatagtggggatcagttcatc**

1130 1140 1150 1160 1170 1180 1190 1200

....|....|....|....|....|....|....|....|....|....|....|....|....|....|....|....|

**BosniaAG**  **caaatggcgttggtaaaactcttgccccagagggcgcaagcggagcagggcctacgggagattgtggcgccgagtcagtc**

**IraqBG**  **caaatggcgttggtaaaactcttgccccagagggcgcaagcggagcagggcctacgggagattgtggcgccgagtcagtc**

**Fribourg-BlancG** **caagaggcgttggtaaaactcttgccccagagggcgcaggcggagcaggggctacaggagattgtggcgccgagtcagtc**

**CuniculiAG**  **caagaggcgttggtaaaactcttgccccagagggcgcaggcggagcaggggctacaggagattgtggcgccgagccagtc**

1210 1220 1230 1240 1250 1260 1270 1280

....|....|....|....|....|....|....|....|....|....|....|....|....|....|....|....|

**BosniaAG**  **ggacatcgtgcttatcatgctgctaacctggcttgagcgtgcacggctggaccggttcaatgctgatgcgctgcttacgg**

**IraqBG**  **ggacatcgtgcttatcatgctgctaacctggcttgagcgtgcacggctggaccggttcaatgctgatgcgctgcttacgg**

**Fribourg-BlancG** **ggacatcgtgcttatcatgctgctgacctggcttgagcgtgcgtggctggaccggttcaatgctgatgcgctgcttacgg**

**CuniculiAG**  **ggacatcgtgcttatcatgctgctgacctggcttgagcgtgcgtggctggaccggttcaatgctgatgcgctgcttacgg**

1290 1300 1310 1320 1330 1340 1350 1360

....|....|....|....|....|....|....|....|....|....|....|....|....|....|....|....|

**BosniaAG**  **cgcagtggacctatgtgtcggctggactgtatggggcgacggcgggtaccaatgtatttggtaagcgcgtgctgcctgcg**

**IraqBG**  **cgcagtggacctatgtgtcggctggactgtatggggcgacggcgggtaccaatgtatttggtaagcgcgtgctgcctgcg**

**Fribourg-BlancG** **cgcagtggacctatgtgtcggctggactgtatggggcgacggcgggtactcatgtatttggcaagcgcgtgctacctacg**

**CuniculiAG**  **cgcagtggacctatgtgtcggctggactgtatggggcgacggcgggtactcatgtatttggcaagcgcgtgctacctacg**

1370 1380 1390 1400 1410 1420 1430 1440

....|....|....|....|....|....|....|....|....|....|....|....|....|....|....|....|

**BosniaAG**  **ctgcagtcctggcattttgattttgctggattccttaagctcgaaactaagagcggtgacccctacacccacctgctcac**

**IraqBG**  **ctgcagtcctggcattttgattttgctggattccttaagctcgaaactaagagcggtgacccctacacccacctgctcac**

**Fribourg-BlancG** **gtgcggtcctaccattttgatctggccagtttttttaaggtggaaattaaaagcggcgacccctatgcccacctgctcac**

**CuniculiAG**  **gtgcggtcctaccattttgatctggccagtttttttaaggtggaaattaaaagcggcgacccctatgcccacctgctcac**

1450 1460 1470 1480 1490 1500 1510 1520

....|....|....|....|....|....|....|....|....|....|....|....|....|....|....|....|

**BosniaAG**  **cggcctgaacgccggcgtcgaagcacgcgtgtacatccccctcacctacatccgttacagaaataacggagggtacccac**

**IraqBG**  **cggcctgaacgccggcgtcgaagcacgcgtgtacatccccctcacctacatccgttacagaaataacggagggtacccac**

**Fribourg-BlancG** **cggcctggacgccggcgttgaaacacgcgtgtacatcccgcttacgcacgtattttatgtaaacaacgggtcgcagccgt**

**CuniculiAG**  **cggcctggacgccggcgttgaaacacgcgtgtacatcccgcttacgcacgtattttatgtaaacaacgggtcgcagccgt**

1530 1540 1550 1560 1570 1580 1590 1600

....|....|....|....|....|....|....|....|....|....|....|....|....|....|....|....|

**BosniaAG**  **tgaatggagttgtgccccctgggactatcaatatgccgattttggggaaggcgtggtgcagctatcgcatccccctcggt**

**IraqBG**  **tgaatggagttgtgccccctgggactatcaatatgccgattttggggaaggcgtggtgcagctatcgcatccccctcggt**

**Fribourg-BlancG** **accaggggatggacagcaccggatacatcaacctgccgatagtgagcagggcgtggtgtagctatcgcatccccctcggc**

**CuniculiAG**  **accaggggatggacagcaccggatacatcaacctgccgatagtgagcagggcgtggtgtagctatcgcatccccctcggc**

1610 1620 1630 1640 1650 1660 1670 1680

....|....|....|....|....|....|....|....|....|....|....|....|....|....|....|....|

**BosniaAG**  **tcccacgcctggcttacaccgcatacatccgtgctcggcacaaccaatcgctttaacgttattaaccccgcgtacaccct**

**IraqBG**  **tcccacgcctggcttacaccgcatacatccgtgctcggcacaaccaatcgctttaacgttattaaccccgcgtacaccct**

**Fribourg-BlancG** **gctcacgtttgggtgaagccctatgcggtgttgaacgagaatactaatcgtgtcaaccgcaatgcgaacggggatgcgct**

**CuniculiAG**  **gctcacgtttgggtgaagccctatgcggtgttgaacgagaatactaatcgtgtcaaccgcaatgcgaacggggatgcgct**

1690 1700 1710 1720 1730 1740 1750 1760

....|....|....|....|....|....|....|....|....|....|....|....|....|....|....|....|

**BosniaAG**  **gttgaatgaacgagcgctccagtaccaggtgggactgacgttcagtcccttcgagaaggtggagctcagcgcccagtggg**

**IraqBG**  **gttgaatgaacgagcgctccagtaccaggtgggactgacgttcagtcccttcgagaaggtggagctcagcgcccagtggg**

**Fribourg-BlancG** **cctgcgtgagcatgcgcttcagtaccaggtgggactgacgttcagtccctt-----------------------------**

**CuniculiAG**  **cctgcgtgagcatgcgcttcagtaccaggtgggactgacgttcagtcccttcgagaaggtggagctcagcgcccagtggg**

1770 1780 1790 1800 1810 1820 1830 1840

....|....|....|....|....|....|....|....|....|....|....|....|....|....|....|....|

**BosniaAG**  **aacagggggtgcttgctg**

**IraqBG**  **aacagggggtgcttgctg**

**Fribourg-BlancG** **------------------**

**CuniculiAG**  **aacagggggtgctctccgatgttccctacatgggtattgccgagagtatgtggtctgagcgttactttggcacgtttatc**

1850 1860

....|....|....|....|....

**BosniaAG**

**IraqBG**

**Fribourg-BlancG**

**CuniculiAG**  **tgtggggtgaaggtggtctggtga**

***1.7 tprH* locus**

10 20 30 40 50 60 70 80

....|....|....|....|....|....|....|....|....|....|....|....|....|....|....|....|

**NicholsH**  **MKGVRCPCLYACRVLYSQSKIRRRRTLLSPSAAVRSSCHDPPGHSAGRRVRSVGTWALLFMSSAAGLCAETRLHTLASTP**

**Bal3H**  **MKGVRCPCLYACRVLYSQSKIRRRRTLLSPSAAVRSSCHDPPGHSAGRRVRSVGTWALLFMSSAAGLCAETRLHTLASTP**

**Sea81-4H**  **MKGVRCPCLYACRVLYSQSKIRRRRTLLSPSAAVRSSCHDPPGHSAGRRVRSVGTWALLFMSSAAGLCAETRLHTLASTP**

**MexicoAH**  **MKGVRCPCLYACRVLYSQSKIRRRRTLLSPSAAVRSSCHDPPGHSAGRRVRSVGTWALLFMSSAAGLCAETRLHTLASTP**

**Street14H MKGVRCPCLYACRVLYSQSKIRRRRTLLSPSAAVRSSCHDPPGHSAGRRVRSVGTWALLFMSSAAGLCAETRLHTLASTP**

**GauthierH**  **MKGVRCPCLYACRVLYSQSKIRRRRTLLSPSAAVRSSCHDPPGHSAGRRVRSVGTWALLFMSSAAGLCGETRLHTLASTP**

**SamoaDH**  **MKGVRCPCLYACRVLYSQSKIRRRRTLLSPSAAVRSSCHDPPGHSAGRRVRSVGTWALLFMSSAAGLCGETRLHTLASTP**

**CDC2H**  **MKGVRCPCLYACRVLYSQSKIRRRRTLLSPSAAVRSSCHDPPGHSAGRRVRSVGTWALLFMSSAAGLCGETRLHTLASTP**

**BosniaAH**  **MKGVRCPCLYACRVLYSQSKIRRRRTLLSPSAAVRSSCHDPPGHSAGRRVRSVGTWALLFMSSAAGLCGETRLHTLASTP**

**IraqBH**  **MKGVRCPCLYACRVLYSQSKIRRRRTLLSPSAAVRSSCHDPPGHSAGRRVRSVGTWALLFMSSAAGLCGETRLHTLASTP**

**Fribourg-BlancH** **MKGVRCPCLYACRVLYSQSKIRRRRTLLSPSAAVRSSCHDPPGHSAGRRVRSVGTWALLFMSSAAGLCGETRLHTLASTP**

**CuniculiAH**  **MKGVRCPCLYACRVLYSQSKIRRRRTLLSPSAAVRSSCHDPPGHSAGRRVRSVGTWALLFMSSAAGLCAETRLHTLASTP**

90 100 110 120 130 140 150 160

....|....|....|....|....|....|....|....|....|....|....|....|....|....|....|....|

**NicholsH**  **RISGFARLQWGITLPYDPAVGPPPPVPPRENGDGDDRDVVNVQIQGNGAQGQEVEESEKEKARIRQITHGFRSTTHLCVT**

**Bal3H**  **RISGFARLQWGITLPYDPAVGPPPPVPPRENGDGDDRDVVNVQIQGNGAQGQEVEESEKEKARIRQITHGFRSTTHLCVT**

**Sea81-4H**  **RISGFARLQWGITLPYDPAVGPPPPVPPRENGDGDDSDVVNVQIQGNGAQGQEVEESEKEKARIRQITHGFRSTAHLCVT**

**MexicoAH**  **RISGFARLQWGITLPYDPAVGPPPPVPPRENGDGDDSDVVNVQIQGNGAQGQEVEESEKEKARIRQITHGFRSTTHLCVT**

**Street14H RISGFARLQWGITLPYDPAVGPPPPVPPRENGDGDDSDVVNVQIQGNGAQGQEVEESEKEKARIRQITHGFRSTTHLCVT**

**GauthierH**  **RISGFARLQWGITLPYDPAVGPPPPVPPRENGDGDDSDVVNVQIQGNGAQGQEVEESEKEKARIRQITHGFRSTTHLCVT**

**SamoaDH**  **RISGFARLQWGITLPYDPAVGPPPPVPPRENGDGDDSDVVNVQIQGNGAQGQEVEESEKEKARIRQITHGFRSTTHLCVT**

**CDC2H**  **RISGFARLQWGITLPYDPAVGPPPPVPPRENGDGDDSDVVNVQIQGNGAQGQEVEESEKEKARIRQITHGFRSTTHLCVT**

**BosniaAH**  **RISGFARLQWGITLPYDPAVGPPPPVPPRENGDGDDSDVVNVQIQGNGAQGQEVEESEKEKARIRQITHGFRSTTHLCVT**

**IraqBH**  **RISGFARLQWGITLPYDPAVGPPPPVPPRENGDGDDSDVVNVQIQGNGAQGQEVEESEKEKARIRQITHGFRSTTHLCVT**

**Fribourg-BlancH** **RISGFARLQWGITLPYDPAVGPPPPVPPRENGDGDDSDVVNVQIQGNGAQGQEVEESEKEKARIRQITHGFRSTTHLCVT**

**CuniculiAH**  **RISGFARLQWGITLPYDPAVGPPPPVPPRENGDGDDSDVVNVQIQGNGAQGQEVEESEKEKARIRQITHGFRSTTHLCVT**

170 180 190 200 210 220 230 240

....|....|....|....|....|....|....|....|....|....|....|....|....|....|....|....|

**NicholsH**  **VPLFLKSDRIRRAGTLSDGGLWTEISIKDLEVNFQTKKPGEPFTLVTEETAIEATLHCFGAYMTIGTAPLFRANFAQLWK**

**Bal3H**  **VPLFLKSDRIRRAGTLSDGGLWTEISIKDLEVNFQTKKPGEPFTLVTEETAIEATLHCFGAYMTIGTAPLFRANFAQLWK**

**Sea81-4H**  **VPLFLKSDRIRRAGTLSDGGLWTEISIKDLEVNFQTKKPGEPFTLVTEETAIEATLHCFGAYMTIGTAPLFRANFAQLWK**

**MexicoAH**  **VPLFLKSDRIRRAGTLSDGGLWTEISIKDLEVNFQTKKPGEPFTLVTEETAIEATLHCFGAYMTIGTAPLFRANFAQLWK**

**Street14H VPLFLKSDRIRRAGTLSDGGLWTEISIKDLEVNFQTKKPGEPFTLVTEETAIEATLHCFGAYMTIGTAPLFRANFAQLWK**

**GauthierH**  **VPLFLKSDRIRRAGTLSDGGLWTEISIKDLEVNFQTKKPGEPFTLVTEETAIEATLHCFGAYMTIGTAPLFRANFAQLWK**

**SamoaDH**  **VPLFLKSDRIRRAGTLSDGGLWTEISIKDLEVNFQTKKPGEPFTLVTEETAIEATLHCFGAYMTIGTAPLFRANFAQLWK**

**CDC2H**  **VPLFLKSDRIRRAGTLSDGGLWTEISIKDLEVNFQTKKPGEPFTLVTEETAIEATLHCFGAYMTMGTAPFFRANFAQLWK**

**BosniaAH**  **VPLFLKSDRIRRAGTLSDGGLWTEISIKDLEVNFQTKKPGEPFTLVTEETAIEATLHCFGAYMTIGTAPLFRANFAQLWK**

**IraqBH**  **VPLFLKSDRIRRAGTLSDGGLWTEISIKDLEVNFQTKKPGEPFTLVTEETAIEATLHCFGAYMTIGTAPLFRANFAQLWK**

**Fribourg-BlancH** **VPLFLKSDRIRRAGTLSDGGLWTEISIKDLEVNFQTKKPGEPFTLVTEETAIEATLHCFGAYMTIGTAPFFRANFAQLWK**

**CuniculiAH**  **VPLFLKSDRIRRAGTLSDGGLWTEISIKDLEVNFQTKKPGEPFTLVTEETAIEATLHCFGAYMTIGTAPLFRANFAQLWK**

250 260 270 280 290 300 310 320

....|....|....|....|....|....|....|....|....|....|....|....|....|....|....|....|

**NicholsH**  **PFLADLYKEEEVRFAPGFDGIGGRLGYRAQNVGSSGVSLDICFLSFASNGSWDAPAPAPAPPGGAAEEALHSKYGFGADA**

**Bal3H**  **PFLADLYKEEEVRFAPGFDGIGGRLGYRAQNVGSSGVSLDICFLSFASNGSWDAPAPAPAPPGGAAEEALHSKYGFGADA**

**Sea81-4H**  **PFLADLYKEEEVRFAPGFDGIGGRLGYRAQNVGSSGVSLDICFLSFASNGSWDAPAPAPAPPGGAAEEALHSKYGFGADA**

**MexicoAH**  **PFLADLYKEEEVRFAPGFDGIGGRLGYRAQNVGSSGVSLDICFLSFASNGSWDAPAPAPAPPGGAAEEALHSKYGFGADA**

**Street14H PFLADLYKEEEVRFAPGFDGIGGRLGYRAQNVGSSGVSLDICFLSFASNGSWDAPAPAPAPPGGAAEEALHSKYGFGADA**

**GauthierH**  **PFLADLYKEEEVRFAPGFDGIGGRLGYRAQNVGSSGVSLDICFLSFASNGSWDAPAPAPAPPGGAAEEALHSKYGFGADA**

**SamoaDH**  **PFLADLYKEEEVRFAPGFDGIGGRLGYRAQNVGSSGVSLDICFLSFASNGSWDAPAPAPAPPGGAAEEALHSKYGFGADA**

**CDC2H**  **PFLADLYKEEEVRFAPGFDGIGGRLGYRAQNVGSSGVSLDICFLSFASNGSWDAPAPAPAPPGGAAEEALHSKYGFGADA**

**BosniaAH**  **PFLADLYKEEEVRFAPGFDGIGGRLGYRAQNVGSSGVSLDICFLSFASNGSWDAPAPAPAPPGGAAEEALHSKYGFGADA**

**IraqBH**  **PFLTDLYKEEEVRFAPGFDGIGGRLGYRAQNVGSSGVSLDICFLSFASNGSWDAPAPAPAPPGGAAEEALHSKYGFGADA**

**Fribourg-BlancH** **PFLADLYKEEEVRFAPGFDGIGGRLGYRAQNVGSSGVSLDICFLSFASNGSWDAPAPAPAPPGGAAEEALHSKYGFGADA**

**CuniculiAH**  **PLLADLYKEEEVRFAPGFDGIGGRLGYRAQNVGSSGVSLDICFLSFASNGSWDAPAPAPAPPGGAAEEALHSKYGFGADA**

330 340 350 360 370 380 390 400

....|....|....|....|....|....|....|....|....|....|....|....|....|....|....|....|

**NicholsH**  **TLSYAPWKRELVRAEVAASATLGKGYKRVSHKQKRHYRQNDVLWNAGARITLTPLSDFKVVLALDMGNHYAGRKTLDYLA**

**Bal3H**  **TLSYAPWKRELVRAEVAASATLGKGYKRVSHKQKRHYRQNDVLWNAGARITLTPLSDFKVVLALDMGNHYAGRKTLDYLA**

**Sea81-4H**  **TLSYAPWKRELVRAEVAASATLGKGYKRVSHKQKRHYRQNDVLWNAGARITLTPLSDFKVVLALDMGNHYAGRKTLDYLA**

**MexicoAH**  **TLSYAPWKRELVRAEVAASATLGKGYKRVSHKQKRHYRQNDVLWNAGARITLTPLSDFKVVLALDMGNHYAGRKTLDYLA**

**Street14H TLSYAPWKRELVRAEVAASATLGKGYKRVSHKQKRHYRQNDVLWNAGARITLTPLSDFKVVLALDMGNHYAGRKTLDYLA**

**GauthierH**  **TLSYAPWKRELVRAEVAASATLGKGYKRVSHKQKRHYRQNDVLWNAGARITLTPLSDFKVVLALDMGNHYAGRKTLDYLA**

**SamoaDH**  **TLSYAPWKRELVRAEVAASATLGKGYKRVSHKQKRHYRQNDVLWNAGARITLTPLSDFKVVLALDMGNHYAGRKTLDYLA**

**CDC2H**  **TLSYAPWKRELVRAEVAASATLGKGYKRVSHKQKRHYRQNDVLWNAGARITLTPLSDFKVVLALDMGNHYAGRKTLDYLA**

**BosniaAH**  **TLSYAPWKRELVRAEVAASATLGKGYKRVSHKQKRHYRQNDVLWNAGARITLTPLSDFKVVLALDMGNHYAGRKTLDYLA**

**IraqBH**  **TLSYAPWKRELVRAEVAASATLGKGYKRVSHKQKRHYRQNDVLWNAGARITLTPLSDFKVVLALDMGNHYAGRKTLDYLA**

**Fribourg-BlancH** **TLSYAPWKRELVRAEVAASATLGKGYKRVSHKQKRHYRQNDVLWNAGARITLTPLSAFKVVLALDMGNHYAGRKTLDYLA**

**CuniculiAH**  **TLSHAPWKRELVRAEVAASATLGKGYKRVSHKQKRHYRQNDVLWNAGARITLTPLSDFKVVLALDMGNHYAGRKTLDYLA**

410 420 430 440 450 460 470 480

....|....|....|....|....|....|....|....|....|....|....|....|....|....|....|....|

**NicholsH**  **PILIDMEKTKVTPGGPVAYAIAQRVLQLPEYAQKLDSVKNGMSANGSSVRDIATKIVQAEQTNPTVSSNPLLAALLTVLW**

**Bal3H**  **PILIDMEKTKVTPGGPVAYAIAQRVLQLPEYAQKLDSVKNGMSANGSSVRDIATKIVQAEQTNPTVSSNPLLAALLTVLW**

**Sea81-4H**  **PILIDMEKTKVTPGGPVAYAIAQRVLQLPEYAQKLDSVKNGMSANGSSVRDIATKIVQAEQTNPTVSSNPLLAALLTVLW**

**MexicoAH**  **PILIDMEKTKVTPGGPVAYAIAQRVLQLPEYAQKLDSVKNGMSANGSSVRDIATKIVQAEQTNPTVSSNPLLAALLTVLW**

**Street14H PILIDMEKTKVTPGGPVAYAIAQRVLQLPEYAQKLDSVKNGMSANGSSVRDIATKIVQAEQTNPTVSSNPLLAALLTVLW**

**GauthierH**  **PILIDMEKTKVTPGGPVAYAIAQRVLQLPEYAQKLDSVKNGMSANGSSVRDIATKIVQAEQTNPTVSSNPLLAALLTVLW**

**SamoaDH**  **PILIDMEKTKVTPGGPVAYAIAQRVLQLPEYAQKLDSVKNGMSANGSSVRDIATKIVQAEQTNPTVSSNPLLAALLTVLW**

**CDC2H**  **PILIDMEKTKVTPGGPVAYAIAQRVLQLPEYAQKLDSVKNGMSANGSSVRDIATKIVQAEQTNPTVSSNPLLAALLTVLW**

**BosniaAH**  **PILIDMEKTKVTPGGPVAYAIAQRVLQLPEYAQKLDSVKNGMSANGSSVRDIATKIVQAEQTNPTVSSNPLLAALLTVLW**

**IraqBH**  **PILIDMEKTKVTPGGPVAYAIAQRVLQLPEYAQKLDSVKNGMSANGSSVRDIATKIVQAEQTNPTVSSNPLLAALLTVLW**

**Fribourg-BlancH** **PILIDMEKTKVTPGGPVAYAIAQRVLQLPEYAQKLDSVKNGMSANGSSVRDIATKVVQAEQTNPTVSSNPLLAALLTVLW**

**CuniculiAH**  **LILIDMEKTKVTPGGPVAYAIAQRVLQLPEYAQKLDSVKNGMSANGSSVRDIATKIVQAEQTNPTVSSNPLLAALLTVLW**

490 500 510 520 530 540 550 560

....|....|....|....|....|....|....|....|....|....|....|....|....|....|....|....|

**NicholsH**  **QQALDTYALDALLTLQWRWFACGVYVATAPASVFGAMVFPTYGSTHTDGGGFLRVETKAGDAYTHLIDGLEAGMDVRCYI**

**Bal3H**  **QQALDTYALDALLTLQWRWFACGVYVATAPASVFGAMVFPTYGSTHTDGGGFLRVETKAGDAYTHLIDGLEAGMDVRCYI**

**Sea81-4H**  **QQALDTYALDALLTLQWRWFACGVYVATAPASVFGAMVFPTYGSTHTDGGGFLRVETKAGDAYTHLIDGLEAGMDVRCYI**

**MexicoAH**  **QQALDTYALDALLTLQWRWFACGVYVATAPASVFGAMVFPTYGSTHTDGGGFLRVETKAGDAYTHLIDGLEAGMDVRCYI**

**Street14H QQALDTYALDALLTLQWRWFACGVYVATAPASVFGAMVFPTYGSTHTDGGGFLRVETKAGDAYTHLIDGLEAGMDVRCYI**

**GauthierH**  **QQALDTYALDALLTLQWRWFACGVYVATAPASVFGAMVFPTYGSTHMDGGGFLRVETKAGDAYTHLIDGLEAGMDVRCYI**

**SamoaDH**  **QQALDTYALDALLTLQWRWFACGVYVATAPASVFGAMVFPTYGSTHMDGGGFLRVETKAGDAYTHLIDGLEAGMDVRCYI**

**CDC2H**  **QQALDTYALDALLTLQWRWFACGVYVATAPASVFGAMVFPTYGSTHMDGGGFLRVETKAGDAYTHLIDGLEAGMDVRCYI**

**BosniaAH**  **QQALDTYALDALLTLQWRWFACGVYVATAPASVFGAMVFPTYGSTHMDGGGFLRVETKAGDAYTHLIDGLEAGMDVRCYI**

**IraqBH**  **QQALDTYALDALLTLQWRWFACGVYVATAPASVFGAMVFPTYGSTHMDGGGFLRVETKAGDAYTHLIDGLEAGMDVRCYI**

**Fribourg-BlancH** **QQALDTYVLDALLTLQWRWFACGVYVATAPASVFGAMVFPTYGSTHMDGGGFLRVETKAGDAYTHLIDGLEAGMDVRCYI**

**CuniculiAH**  **QQALDTYALDALLTLQWRWFACGVYVATAPASVFGAMVFPTYGSTHTDGGGFLRVETKAGDAYTHLIDGLEAGMDVRCYI**

570 580 590 600 610 620 630 640

....|....|....|....|....|....|....|....|....|....|....|....|....|....|....|....|

**NicholsH**  **PLTHGLYIDNGKGYYVSPMGVWKLPDTHINLPVMGKVWARYLIPLGETAWLKPSLAVYGTTNRFNYNLKTENLVHERCVQ**

**Bal3H**  **PLTHGLYIDNGKGYYVSPMGVWKLPDTHINLPVMGKVWARYLIPLGETAWLKPSLAVYGTTNRFNYNLKTENLVHERCVQ**

**Sea81-4H**  **PLTHGLYIDNGKGYYVSPMGVWKLPDTHINLPVMGKVWARYLIPLGETAWLKPSLAVYGTTNRFNYNLKTENLVHERCVQ**

**MexicoAH**  **PLTHGLYIDNGKGYYVSPMGVWKLPDTHINLPVMGKVWARYLIPLGETAWLKPSLAVYGTTNRFNYNLKTENLVHERCVQ**

**Street14H PLTHGLYIDNGKGYYVSPMGVWKLPDTHINLPVMGKVWARYLIPLGETAWLKPSLAVYGTTNRFNYNLKTENLVHERCVQ**

**GauthierH**  **PLTHGLYIDNGKGYYVSPMGVWKLPDTHINLPVMGKVWARYLIPLGETAWLKPSLAVYGTTNRFNYNLKTENLVHERCVQ**

**SamoaDH**  **PLTHGLYIDNGKGYYVSPMGVWKLPDTHINLPVMGKVWARYLIPLGETAWLKPSLAVYGTTNRFNYNLKTENLVHERCVQ**

**CDC2H**  **PLTHGLYIDNGKGYYVSPMGVWKLPDTHINLPVMGKVWARYLIPLGETAWLKPSLAVYGTTNRFNYNLKTENLVHERCVQ**

**BosniaAH**  **PLTHGLYIDNGKGYYVSPMGVWKLPDTHINLPVMGKVWARYLIPLGETAWLKPSLAVYGTTNRFNYNLKTENLVHERCVQ**

**IraqBH**  **PLTHGLYIDNGKGYYVSPMGVWKLPDTHINLPVMGKVWARYLIPLGETAWLKPSLAVYGTTNRFNYNIKTENLVHERCVQ**

**Fribourg-BlancH** **PLTHGLYIDNGKGYYVSPMGVWKLPDTHIDLPVMGKVWARYLIPLGETAWLKPSLAVYGTTNRFNYNLKTENLVHERCVQ**

**CuniculiAH**  **PLTHGLYIDNGKGYYVSPMGVWKLPDTHINLPVMGKVWARYLIPLGETAWLKPSLAVYGTTNRFNYNLKTENLVRERCVQ**

650 660 670 680 690

....|....|....|....|....|....|....|....|....|....|...

**NicholsH**  **YQVGLTLSPIEKVTLHAQWEQGQLEPTPYMVITETVTSRRYFGTFVCGMTINW**

**Bal3H**  **YQVGLTLSPIEKVTLHAQWEQGQLEPTPYMVITETVTSRRYFGTFVCGMTINW**

**Sea81-4H**  **YQVGLTLSPIEKVTLHAQWEQGQLEPTPYMVITETVTSRRYFGTFVCGMTINW**

**MexicoAH**  **YQVGLTLSPIEKVTLHAQWEQGQLEPTPYMVITETVTSRRYFGTFVCGMTINW**

**Street14H YQVGLTLSPIEKVTLHAQWEQGQLEPTPYMVITETVTSRRYFGTFVCGMTINW**

**GauthierH**  **YQVGLTLSPIEKVTLHAQWEQGQLEPTPYMVITETVTSRRYFGTFVCGMTINW**

**SamoaDH**  **YQVGLTLSPIEKVTLHAQWEQGQLEPTPYMVITETVTSRRYFGTFVCGMTINW**

**CDC2H**  **YQVGLTLSPIEKVTLHAQWEQGQLEPTPYMVITETVTSRRYFGTFVCGMTINW**

**BosniaAH**  **YQVGLTLSPIEKVTLHAQWEQGQLEPTPYMVITETVTSRRYFGTFVCGMTINW**

**IraqBH**  **YQVGLTLSPIEKVTLHAQWEQGQLEPTPYMVITETVTSRRYFGTFVCGMTINW**

**Fribourg-BlancH** **YQVGLTLSPIEKVTLHAQWEQGQLEPTPYMVITETVTSRRYFGTFVCGMTINW**

**CuniculiAH**  **YQVGLTLSPIEKVTLHAQWEQGQLEPTPYMVITETVTSRRYFGTFVCGMTINW**

10 20 30 40 50 60 70 80

....|....|....|....|....|....|....|....|....|....|....|....|....|....|....|....|

**NicholsH**  **atgaaaggagtacgctgtccctgtctttatgcctgcagggtgttgtactctcaaagcaaaatccggcggcgacgcacgct**

**Bal3H**  **atgaaaggagtacgctgtccctgtctttatgcctgcagggtgttgtactctcaaagcaaaatccggcggcgacgcacgct**

**Sea81-4H**  **atgaaaggagtacgctgtccctgtctttatgcctgcagggtgttgtactctcaaagcaaaatccggcggcgacgcacgct**

**MexicoAH**  **atgaaaggagtacgctgtccctgtctttatgcctgcagggtgttgtactctcaaagcaaaatccggcggcgacgcacgct**

**Street14H atgaaaggagtacgctgtccctgtctttatgcctgcagggtgttgtactctcaaagcaaaatccggcggcgacgcacgct**

**GauthierH**  **atgaaaggagtacgctgtccctgtctttatgcctgcagggtgttgtactctcaaagcaaaatccggcggcgacgcacgct**

**SamoaDH**  **atgaaaggagtacgctgtccctgtctttatgcctgcagggtgttgtactctcaaagcaaaatccggcggcgacgcacgct**

**CDC2H**  **atgaaaggagtacgctgtccctgtctttatgcctgcagggtgttgtactctcaaagcaaaatccggcggcgacgcacgct**

**BosniaAH**  **atgaaaggagtacgctgtccctgtctttatgcctgcagggtgttgtactctcaaagcaaaatccggcggcgacgcacgct**

**IraqBH**  **atgaaaggagtacgctgtccctgtctttatgcctgcagggtgttgtactctcaaagcaaaatccggcggcgacgcacgct**

**Fribourg-BlancH** **atgaaaggagtacgctgtccctgtctttatgcctgcagggtgttgtactctcaaagcaaaatccggcggcgacgcacgct**

**CuniculiAH**  **atgaaaggagtacgctgtccctgtctttatgcctgcagggtgttgtactctcaaagcaaaatccggcggcgacgcacgct**

90 100 110 120 130 140 150 160

....|....|....|....|....|....|....|....|....|....|....|....|....|....|....|....|

**NicholsH**  **gctgtctccgtcggcggctgtgcgctcctcctgccatgatccccctggacactcagcagggaggcgtgtcaggtcagttg**

**Bal3H**  **gctgtctccgtcggcggctgtgcgctcctcctgccatgatccccctggacactcagcagggaggcgtgtcaggtcagttg**

**Sea81-4H**  **gctgtctccgtcggcggctgtgcgctcctcctgccatgatccccctggacactcagcagggaggcgtgtcaggtcagttg**

**MexicoAH**  **gctgtctccgtcggcggctgtgcgctcctcctgccatgatccccctggacactcagcagggaggcgtgtcaggtcagttg**

**Street14H gctgtctccgtcggcggctgtgcgctcctcctgccatgatccccctggacactcagcagggaggcgtgtcaggtcagttg**

**GauthierH**  **gctgtctccgtcggcggctgtgcgctcctcctgccatgatccccctggacactcagcagggaggcgtgtcaggtcagttg**

**SamoaDH**  **gctgtctccgtcggcggctgtgcgctcctcctgccatgatccccctggacactcagcagggaggcgtgtcaggtcagttg**

**CDC2H**  **gctgtctccgtcggcggctgtgcgctcctcctgccatgatccccctggacactcagcagggaggcgtgtcaggtcagttg**

**BosniaAH**  **gctgtctccgtcggcggctgtgcgctcctcctgccatgatccccctggacactcagcagggaggcgtgtcaggtcagttg**

**IraqBH**  **gctgtctccgtcggcggctgtgcgctcctcctgccatgatccccctggacactcagcagggaggcgtgtcaggtcagttg**

**Fribourg-BlancH** **gctgtctccgtcggcggctgtgcgctcctcctgccatgatccccctggacactcagcagggaggcgtgtcaggtcagttg**

**CuniculiAH**  **gctgtctccgtcggcggctgtgcgctcctcctgccatgatccccctggacactcagcagggaggcgtgtcaggtcagttg**

170 180 190 200 210 220 230 240

....|....|....|....|....|....|....|....|....|....|....|....|....|....|....|....|

**NicholsH**  **gcacgtgggcgctcctctttatgtcgtccgcagcaggactgtgtgcagaaaccagactgcacaccttagcaagtacgccg**

**Bal3H**  **gcacgtgggcgctcctctttatgtcgtccgcagcaggactgtgtgcagaaaccagactgcacaccttagcaagtacgccg**

**Sea81-4H**  **gcacgtgggcgctcctctttatgtcgtccgcagcaggactgtgtgcagaaaccagactgcacaccttagcaagtacgccg**

**MexicoAH**  **gcacgtgggcgctcctctttatgtcgtccgcagcaggactgtgtgcagaaaccagactgcacaccttagcaagtacgccg**

**Street14H gcacgtgggcgctcctctttatgtcgtccgcagcaggactgtgtgcagaaaccagactgcacaccttagcaagtacgccg**

**GauthierH**  **gcacgtgggcgctcctctttatgtcgtccgcagcagggctgtgtggagaaaccagactgcacaccttagcaagtacgccg**

**SamoaDH**  **gcacgtgggcgctcctctttatgtcgtccgcagcagggctgtgtggagaaaccagactgcacaccttagcaagtacgccg**

**CDC2H**  **gcacgtgggcgctcctctttatgtcgtccgcagcagggctgtgtggagaaaccagactgcacaccttagcaagtacgccg**

**BosniaAH**  **gcacgtgggcgctcctctttatgtcgtccgcagcagggctgtgtggagaaaccagactgcacaccttagcaagtacgccg**

**IraqBH**  **gcacgtgggcgctcctctttatgtcgtccgcagcagggctgtgtggagaaaccagactgcacaccttagcaagtacgccg**

**Fribourg-BlancH** **gcacgtgggcgctcctctttatgtcgtccgcagcagggctgtgtggagaaacgagactgcacaccttagcaagtacgccg**

**CuniculiAH**  **gcacgtgggcgctcctctttatgtcgtccgcagcagggctgtgtgcagaaaccagactgcacaccttagcaagtacgccg**

250 260 270 280 290 300 310 320

....|....|....|....|....|....|....|....|....|....|....|....|....|....|....|....|

**NicholsH**  **cggatttcaggctttgcccggttgcagtggggtatcacgctcccctacgaccccgcagtaggtccgccgcctcccgtgcc**

**Bal3H**  **cggatttcaggctttgcccggttgcagtggggtatcacgctcccctacgaccccgcagtaggtccgccgcctcccgtgcc**

**Sea81-4H**  **cggatttcaggctttgcccggttgcagtggggtatcacgctcccctacgaccccgcagtaggtccgccgcctcccgtgcc**

**MexicoAH**  **cggatttcaggctttgcccggttgcagtggggtatcacgctcccctacgaccccgcagtaggtccgccgcctcccgtgcc**

**Street14H cggatttcaggctttgcccggttgcagtggggtatcacgctcccctacgaccccgcagtaggtccgccgcctcccgtgcc**

**GauthierH**  **cggatttcaggctttgcccggttgcagtggggtatcacgctcccctacgaccccgcagtaggtccgccgcctcccgtgcc**

**SamoaDH**  **cggatttcaggctttgcccggttgcagtggggtatcacgctcccctacgaccccgcagtaggtccgccgcctcccgtgcc**

**CDC2H**  **cggatttcaggctttgcccggttgcagtggggtatcacgctcccctacgaccccgcagtaggtccgccgcctcccgtgcc**

**BosniaAH**  **cggatttcaggctttgcccggttgcagtggggtatcacgctcccctacgaccccgcagtaggtccgccgcctcccgtgcc**

**IraqBH**  **cggatttcaggctttgcccggttgcagtggggtatcacgctcccctacgaccccgcagtaggtccgccgcctcccgtgcc**

**Fribourg-BlancH** **cggatttcaggctttgcccggttgcagtggggtatcacgctcccctacgaccccgcagtaggtccgccgcctcccgtgcc**

**CuniculiAH**  **cggatttcaggctttgcccggttgcagtggggtatcacgctcccctacgaccccgcagtaggtccgccgcctcccgtgcc**

330 340 350 360 370 380 390 400

....|....|....|....|....|....|....|....|....|....|....|....|....|....|....|....|

**NicholsH**  **gccccgcgagaatggagatggcgacgacagagatgtggtgaacgtacagatacagggaaatggagcgcagggacaggaag**

**Bal3H**  **gccccgcgagaatggagatggcgacgacagagatgtggtgaacgtacagatacagggaaatggagcgcagggacaggaag**

**Sea81-4H**  **gccccgcgagaatggagatggcgacgacagcgatgtggtgaacgtacagatacagggaaatggagcgcagggacaggaag**

**MexicoAH**  **gccccgcgagaatggagatggcgacgacagcgatgtggtgaacgtacagatacagggaaatggagcgcagggacaggaag**

**Street14H gccccgcgagaatggagatggcgacgacagcgatgtggtgaacgtacagatacagggaaatggagcgcagggacaggaag**

**GauthierH**  **gccccgcgagaatggagatggcgacgacagcgatgtggtgaacgtacagatacagggaaatggagcgcagggacaggaag**

**SamoaDH**  **gccccgcgagaatggagatggcgacgacagcgatgtggtgaacgtacagatacagggaaatggagcgcagggacaggaag**

**CDC2H**  **gccccgcgagaatggagatggcgacgacagcgatgtggtgaacgtacagatacagggaaatggagcgcagggacaggaag**

**BosniaAH**  **gccccgcgagaatggagatggcgacgacagcgatgtggtgaacgtacagatacagggaaatggagcgcagggacaggaag**

**IraqBH**  **gccccgcgagaatggagatggcgacgacagcgatgtggtgaacgtacagatacagggaaatggagcgcagggacaggaag**

**Fribourg-BlancH** **gccccgcgagaatggagatggcgacgacagcgatgtggtgaacgtacagatacagggaaatggagcgcagggacaggaag**

**CuniculiAH**  **gccccgcgagaatggagatggcgacgacagcgatgtggtgaacgtacagatacagggaaatggagcgcagggacaggaag**

410 420 430 440 450 460 470 480

....|....|....|....|....|....|....|....|....|....|....|....|....|....|....|....|

**NicholsH**  **tagaagagagtgagaaggaaaaagcgcgcattcgtcagataacgcacgggttccgcagtaccacacacttgtgtgtaact**

**Bal3H**  **tagaagagagtgagaaggaaaaagcgcgcattcgtcagataacgcacgggttccgcagtaccacacacttgtgtgtaact**

**Sea81-4H**  **tagaagagagtgagaaggaaaaagcgcgcattcgtcagataacgcacgggttccgcagtaccgcacacttgtgtgtaact**

**MexicoAH**  **tagaagagagtgagaaggaaaaagcgcgcattcgtcagataacgcacgggttccgcagtaccacacacttgtgtgtaact**

**Street14H tagaagagagtgagaaggaaaaagcgcgcattcgtcagataacgcacgggttccgcagtaccacacacttgtgtgtaact**

**GauthierH**  **tagaagagagtgagaaggaaaaagcgcgcattcgtcagataacgcacgggttccgcagtaccacacacttgtgtgtaact**

**SamoaDH**  **tagaagagagtgagaaggaaaaagcgcgcattcgtcagataacgcacgggttccgcagtaccacacacttgtgtgtaact**

**CDC2H**  **tagaagagagtgagaaggaaaaagcgcgcattcgtcagataacgcacgggttccgcagtaccacacacttgtgtgtaact**

**BosniaAH**  **tagaagagagtgagaaggaaaaagcgcgcattcgtcagataacgcacgggttccgcagtaccacacacttgtgtgtaact**

**IraqBH**  **tagaagagagtgagaaggaaaaagcgcgcattcgtcagataacgcacgggttccgcagtaccacacacttgtgtgtaact**

**Fribourg-BlancH** **tagaagagagtgagaaggaaaaagcgcgcattcgtcagataacgcacgggttccgcagtaccacacacttgtgtgtaact**

**CuniculiAH**  **tagaagagagtgagaaggaaaaagcgcgcattcgtcagataacgcacgggttccgcagtaccacacacttgtgtgtaact**

490 500 510 520 530 540 550 560

....|....|....|....|....|....|....|....|....|....|....|....|....|....|....|....|

**NicholsH**  **gttccgttgtttttaaaaagtgaccgcataaggcgcgcaggtacattatctgacggtggcttgtggactgaaatttctat**

**Bal3H**  **gttccgttgtttttaaaaagtgaccgcataaggcgcgcaggtacattatctgacggtggcttgtggactgaaatttctat**

**Sea81-4H**  **gttccgttgtttttaaaaagtgaccgcataaggcgcgcaggtacattatctgacggtggcttgtggactgaaatttctat**

**MexicoAH**  **gttccgttgtttttaaaaagtgaccgcataaggcgcgcaggtacattatctgacggtggcttgtggactgaaatttctat**

**Street14H gttccgttgtttttaaaaagtgaccgcataaggcgcgcaggtacattatctgacggtggcttgtggactgaaatttctat**

**GauthierH**  **gttccgttgtttttaaaaagtgaccgcataaggcgcgcaggtacattatctgacggtggcttgtggactgaaatttctat**

**SamoaDH**  **gttccgttgtttttaaaaagtgaccgcataaggcgcgcaggtacattatctgacggtggcttgtggactgaaatttctat**

**CDC2H**  **gttccgttgtttttaaaaagtgaccgcataaggcgcgcaggtacattatctgacggtggcttgtggactgaaatttctat**

**BosniaAH**  **gttccgttgtttttaaaaagtgaccgcataaggcgcgcaggtacattatctgacggtggcttgtggactgaaatttctat**

**IraqBH**  **gttccgttgtttttaaaaagtgaccgcataaggcgcgcaggtacattatctgacggtggcttgtggactgaaatttctat**

**Fribourg-BlancH** **gttccgttgtttttaaaaagtgaccgcataaggcgcgcaggtacattatctgacggtggcttgtggactgaaatttctat**

**CuniculiAH**  **gttccgttgtttttaaaaagtgaccgcataaggcgcgcaggtacattatctgacggtggcttgtggactgaaatttctat**

570 580 590 600 610 620 630 640

....|....|....|....|....|....|....|....|....|....|....|....|....|....|....|....|

**NicholsH**  **caaggatttggaggtaaattttcagaccaaaaagcctggtgagccttttaccctagtcacagaagaaaccgctattgaag**

**Bal3H**  **caaggatttggaggtaaattttcagaccaaaaagcctggtgagccttttaccctagtcacagaagaaaccgctattgaag**

**Sea81-4H**  **caaggatttggaggtaaattttcagaccaaaaagcctggtgagccttttaccctagtcacagaagaaaccgctattgaag**

**MexicoAH**  **caaggatttggaggtaaattttcagaccaaaaagcctggtgagccttttaccctagtcacagaagaaaccgctattgaag**

**Street14H caaggatttggaggtaaattttcagaccaaaaagcctggtgagccttttaccctagtcacagaagaaaccgctattgaag**

**GauthierH**  **caaggatttggaggtaaattttcagaccaaaaagcctggtgagccttttaccctagtcacagaagaaaccgctattgaag**

**SamoaDH**  **caaggatttggaggtaaattttcagaccaaaaagcctggtgagccttttaccctagtcacagaagaaaccgctattgaag**

**CDC2H**  **caaggatttggaggtaaattttcagaccaaaaagcctggtgagccttttaccctagtcacagaagaaaccgctattgaag**

**BosniaAH**  **caaggatttggaggtaaattttcagaccaaaaagcctggtgagccttttaccctagtcacagaagaaaccgctattgaag**

**IraqBH**  **caaggatttggaggtaaattttcagaccaaaaagcctggtgagccttttaccctagtcacagaagaaaccgctattgaag**

**Fribourg-BlancH** **caaggatttggaggtaaattttcagaccaaaaagcctggtgagccttttaccctagtcacagaagaaaccgctattgaag**

**CuniculiAH**  **caaggatttggaggtgaattttcagaccaaaaagcctggtgagccttttaccctagtcacagaagaaaccgctattgaag**

650 660 670 680 690 700 710 720

....|....|....|....|....|....|....|....|....|....|....|....|....|....|....|....|

**NicholsH**  **cgacgctacactgctttggtgcgtatatgacgatcggcaccgctcccctctttcgcgccaattttgcccaactgtggaaa**

**Bal3H**  **cgacgctacactgctttggtgcgtatatgacgatcggcaccgctcccctctttcgcgccaattttgcccaactgtggaaa**

**Sea81-4H**  **cgacgctacactgctttggtgcgtatatgacgatcggcaccgctcccctctttcgcgccaattttgcccaactgtggaaa**

**MexicoAH**  **cgacgctacactgctttggtgcgtatatgacgatcggcaccgctcccctctttcgcgccaattttgcccaactgtggaaa**

**Street14H cgacgctacactgctttggtgcgtatatgacgatcggcaccgctcccctctttcgcgccaattttgcccaactgtggaaa**

**GauthierH**  **cgacgctacactgctttggtgcgtatatgacgatcggcaccgctcccctctttcgcgccaattttgcccaactgtggaaa**

**SamoaDH**  **cgacgctacactgctttggtgcgtatatgacgatcggcaccgctcccctctttcgcgccaattttgcccaactgtggaaa**

**CDC2H**  **cgacgctacactgctttggtgcgtatatgacgatgggcaccgctcccttctttcgcgccaattttgcccaactgtggaaa**

**BosniaAH**  **cgacgctacactgctttggtgcgtatatgacgatcggcaccgctcccctctttcgcgccaattttgcccaactgtggaaa**

**IraqBH**  **cgacgctacactgctttggtgcgtatatgacgatcggcaccgctcccctctttcgcgccaattttgcccaactgtggaaa**

**Fribourg-BlancH** **cgacgctacactgctttggtgcgtatatgacgatcggcaccgctcccttctttcgcgccaattttgcccaactgtggaaa**

**CuniculiAH**  **cgacgctacactgctttggtgcgtatatgacgatcggcaccgctcccctctttcgcgccaattttgcccaactgtggaaa**

730 740 750 760 770 780 790 800

....|....|....|....|....|....|....|....|....|....|....|....|....|....|....|....|

**NicholsH**  **ccttttcttgcagacctctacaaggaagaggaggtgcggtttgcgccagggttcgatggcatcggcggcaggctcggcta**

**Bal3H**  **ccttttcttgcagacctctacaaggaagaggaggtgcggtttgcgccagggttcgatggcatcggcggcaggctcggcta**

**Sea81-4H**  **ccttttcttgcagacctctacaaggaagaggaggtgcggtttgcgccagggttcgatggcatcggcggcaggctcggcta**

**MexicoAH**  **ccttttcttgcagacctctacaaggaagaggaggtgcggtttgcgccagggttcgatggcatcggcggcaggctcggcta**

**Street14H ccttttcttgcagacctctacaaggaagaggaggtgcggtttgcgccagggttcgatggcatcggcggcaggctcggcta**

**GauthierH**  **ccttttcttgcagacctctacaaggaagaggaggtgcggtttgcgccagggttcgatggcatcggcggcaggctcggcta**

**SamoaDH**  **ccttttcttgcagacctctacaaggaagaggaggtgcggtttgcgccagggttcgatggcatcggcggcaggctcggcta**

**CDC2H**  **ccttttcttgcagacctctacaaggaagaggaggtgcggtttgcgccagggttcgatggcatcggcggcaggctcggcta**

**BosniaAH**  **ccttttcttgcagacctctacaaggaagaggaggtgcggtttgcgccagggttcgatggcatcggcggcaggctcggcta**

**IraqBH**  **ccttttcttacagacctctacaaggaagaggaggtgcggtttgcgccagggttcgatggcatcggcggcaggctcggcta**

**Fribourg-BlancH** **ccttttcttgcagacctctacaaggaagaggaggtgcggtttgcgccagggttcgatggcatcggcggcaggctcggcta**

**CuniculiAH**  **cctcttcttgcagacctctacaaggaagaggaggtgcggtttgcgccagggttcgatggcatcggcggcaggctcggcta**

810 820 830 840 850 860 870 880

....|....|....|....|....|....|....|....|....|....|....|....|....|....|....|....|

**NicholsH**  **ccgtgcacagaacgtaggctcaagtggagtgagcttggatatttgctttctttcctttgcctcaaatggttcctgggacg**

**Bal3H**  **ccgtgcacagaacgtaggctcaagtggagtgagcttggatatttgctttctttcctttgcctcaaatggttcctgggacg**

**Sea81-4H**  **ccgtgcacagaacgtaggctcaagtggagtgagcttggatatttgctttctttcctttgcctcaaatggttcctgggacg**

**MexicoAH**  **ccgtgcacagaacgtaggctcaagtggagtgagcttggatatttgctttctttcctttgcctcaaatggttcctgggacg**

**Street14H ccgtgcacagaacgtaggctcaagtggagtgagcttggatatttgctttctttcctttgcctcaaatggttcctgggacg**

**GauthierH**  **ccgtgcacagaacgtaggctcaagtggagtgagcttggatatttgctttctttcctttgcctcaaatggttcctgggacg**

**SamoaDH**  **ccgtgcacagaacgtaggctcaagtggagtgagcttggatatttgctttctttcctttgcctcaaatggttcctgggacg**

**CDC2H**  **ccgtgcacagaacgtaggctcaagtggagtgagcttggatatttgctttctttcctttgcctcaaatggttcctgggacg**

**BosniaAH**  **ccgtgcacagaacgtaggctcaagtggagtgagcttggatatttgctttctttcctttgcctcaaatggttcctgggacg**

**IraqBH**  **ccgtgcacagaacgtaggctcaagtggagtgagcttggatatttgctttctttcctttgcctcaaatggttcctgggacg**

**Fribourg-BlancH** **ccgtgcacagaacgtaggctcaagtggagtgagcttggatatttgctttctttcctttgcctcaaatggttcctgggacg**

**CuniculiAH**  **ccgtgcacagaacgtaggctcaagtggagtgagcttggatatttgctttctttcctttgcctcaaatggttcctgggacg**

890 900 910 920 930 940 950 960

....|....|....|....|....|....|....|....|....|....|....|....|....|....|....|....|

**NicholsH**  **cgcctgcccctgctcctgctccccctggaggtgcggcggaagaggctctgcatagcaagtatggtttcggggcagacgcg**

**Bal3H**  **cgcctgcccctgctcctgctccccctggaggtgcggcggaagaggctctgcatagcaagtatggtttcggggcagacgcg**

**Sea81-4H**  **cgcctgcccctgctcctgctccccctggaggtgcggcggaagaggctctgcatagcaagtatggtttcggggcagacgcg**

**MexicoAH**  **cgcctgcccctgctcctgctccccctggaggtgcggcggaagaggctctgcatagcaagtatggtttcggggcagacgcg**

**Street14H cgcctgcccctgctcctgctccccctggaggtgcggcggaagaggctctgcatagcaagtatggtttcggggcagacgcg**

**GauthierH**  **cgcctgcccctgctcctgctccccctggaggtgcggcggaagaggctctgcatagcaagtatggtttcggggcagacgcg**

**SamoaDH**  **cgcctgcccctgctcctgctccccctggaggtgcggcggaagaggctctgcatagcaagtatggtttcggggcagacgcg**

**CDC2H**  **cgcctgcccctgctcctgctccccctggaggtgcggcggaagaggctctgcatagcaagtatggtttcggggcagacgcg**

**BosniaAH**  **cgcctgcccctgctcctgctccccctggaggtgcggcggaagaggctctgcatagcaagtatggtttcggggcagacgcg**

**IraqBH**  **cgcctgcccctgctcctgctccccctggaggtgcggcggaagaggctctgcatagcaagtatggtttcggggcagacgcg**

**Fribourg-BlancH** **cgcctgcccctgctcctgctccccctggaggtgcggcggaagaggctctgcatagcaagtatggtttcggggcagacgcg**

**CuniculiAH**  **cgcctgcccctgctcctgctccccctggaggtgcggcggaagaggctctgcatagcaagtatggtttcggggcagacgcg**

970 980 990 1000 1010 1020 1030 1040

....|....|....|....|....|....|....|....|....|....|....|....|....|....|....|....|

**NicholsH**  **acgctcagctatgcgccgtggaaacgcgagttagtacgggcagaagtggcagccagcgcgacactgggaaaaggctataa**

**Bal3H**  **acgctcagctatgcgccgtggaaacgcgagttagtacgggcagaagtggcagccagcgcgacactgggaaaaggctataa**

**Sea81-4H**  **acgctcagctatgcgccgtggaaacgcgagttagtacgggcagaagtggcagccagcgcgacactgggaaaaggctataa**

**MexicoAH**  **acgctcagctatgcgccgtggaaacgcgagttagtacgggcagaagtggcagccagcgcgacactgggaaaaggctataa**

**Street14H acgctcagctatgcgccgtggaaacgcgagttagtacgggcagaagtggcagccagcgcgacactgggaaaaggctataa**

**GauthierH**  **acgctcagctatgcgccgtggaaacgcgagttagtacgggcagaagtggcagccagcgcgacactgggaaaaggctataa**

**SamoaDH**  **acgctcagctatgcgccgtggaaacgcgagttagtacgggcagaagtggcagccagcgcgacactgggaaaaggctataa**

**CDC2H**  **acgctcagctatgcgccgtggaaacgcgagttagtacgggcagaagtggcagccagcgcgacactgggaaaaggctataa**

**BosniaAH**  **acgctcagctatgcgccgtggaaacgcgagttagtacgggcagaagtggcagccagcgcgacactgggaaaaggctataa**

**IraqBH**  **acgctcagctatgcgccgtggaaacgcgagttagtacgggcagaagtggcagccagcgcgacactgggaaaaggctataa**

**Fribourg-BlancH** **acgctcagctatgcgccgtggaaacgcgagttagtacgggcagaagtggcagccagcgcgacactgggaaaaggctataa**

**CuniculiAH**  **acgctcagccatgcgccgtggaaacgcgagttagtacgggcagaagtggcagccagcgcgacactgggaaaaggctataa**

1050 1060 1070 1080 1090 1100 1110 1120

....|....|....|....|....|....|....|....|....|....|....|....|....|....|....|....|

**NicholsH**  **aagggtcagccataaacagaaaaggcactataggcagaatgacgtcctgtggaatgcaggcgcgcgcataacgctcactc**

**Bal3H**  **aagggtcagccataaacagaaaaggcactataggcagaatgacgtcctgtggaatgcaggcgcgcgcataacgctcactc**

**Sea81-4H**  **aagggtcagccataaacagaaaaggcactataggcagaatgacgtcctgtggaatgcaggcgcgcgcataacgctcactc**

**MexicoAH**  **aagggtcagccataaacagaaaaggcactataggcagaatgacgtcctgtggaatgcaggcgcgcgcataacgctcactc**

**Street14H aagggtcagccataaacagaaaaggcactataggcagaatgacgtcctgtggaatgcaggcgcgcgcataacgctcactc**

**GauthierH**  **aagggtcagccataaacagaaaaggcactataggcagaatgacgtcctgtggaatgcaggcgcgcgcataacgctcactc**

**SamoaDH**  **aagggtcagccataaacagaaaaggcactataggcagaatgacgtcctgtggaatgcaggcgcgcgcataacgctcactc**

**CDC2H**  **aagggtcagccataaacagaaaaggcactataggcagaatgacgtcctgtggaatgcaggcgcgcgcataacgctcactc**

**BosniaAH**  **aagggtcagccataaacagaaaaggcactataggcagaatgacgtcctgtggaatgcaggcgcgcgcataacgctcactc**

**IraqBH**  **aagggtcagccataaacagaaaaggcactataggcagaatgacgtcctgtggaatgcaggcgcgcgcataacgctcactc**

**Fribourg-BlancH** **aagggtcagccataaacagaaaaggcactataggcagaatgacgtcctgtggaatgcaggcgcgcgcataacgctcactc**

**CuniculiAH**  **aagggtcagccataaacagaaaaggcactataggcagaatgacgtcctgtggaatgcaggcgcgcgcataacgctcactc**

1130 1140 1150 1160 1170 1180 1190 1200

....|....|....|....|....|....|....|....|....|....|....|....|....|....|....|....|

**NicholsH**  **ctctttcggacttcaaggtggtgttggctctggacatgggtaaccattatgcaggtcggaaaacgctcgactatcttgcc**

**Bal3H**  **ctctttcggacttcaaggtggtgttggctctggacatgggtaaccattatgcaggtcggaaaacgctcgactatcttgcc**

**Sea81-4H**  **ctctttcggacttcaaggtggtgttggctctggacatgggtaaccattatgcaggtcggaaaacgctcgactatcttgcc**

**MexicoAH**  **ctctttcggacttcaaggtggtgttggctctggacatgggtaaccattatgcaggtcggaaaacgctcgactatcttgcc**

**Street14H ctctttcggacttcaaggtggtgttggctctggacatgggtaaccattatgcaggtcggaaaacgctcgactatcttgcc**

**GauthierH**  **ctctttcggacttcaaggtggtgttggctctggacatgggtaaccattatgcaggtcggaaaacgctcgactatcttgcc**

**SamoaDH**  **ctctttcggacttcaaggtggtgttggctctggacatgggtaaccattatgcaggtcggaaaacgctcgactatcttgcc**

**CDC2H**  **ctctttcggacttcaaggtggtgttggctctggacatgggtaaccattatgcaggtcggaaaacgctcgactatcttgcc**

**BosniaAH**  **ctctttcggacttcaaggtggtgttggctctggacatgggtaaccattatgcaggtcggaaaacgctcgactatcttgcc**

**IraqBH**  **ctctttcggacttcaaggtggtgttggctctggacatgggtaaccattatgcaggtcggaaaacgctcgactatcttgcc**

**Fribourg-BlancH** **ctctttcggccttcaaggtggtgttggctctggacatgggtaaccattatgcaggtcggaaaacgctcgactatcttgcc**

**CuniculiAH**  **ctctttcggacttcaaggtggtgttggctctggacatgggtaaccattatgcaggtcggaaaacgctcgactatcttgcc**

1210 1220 1230 1240 1250 1260 1270 1280

....|....|....|....|....|....|....|....|....|....|....|....|....|....|....|....|

**NicholsH**  **ccgatccttatcgatatggaaaaaaccaaggtcacccccggagggccggtggcgtatgccattgcacagcgcgtgttgca**

**Bal3H**  **ccgatccttatcgatatggaaaaaaccaaggtcacccccggagggccggtggcgtatgccattgcacagcgcgtgttgca**

**Sea81-4H**  **ccgatccttatcgatatggaaaaaaccaaggtcacccccggagggccggtggcgtatgccattgcacagcgcgtgttgca**

**MexicoAH**  **ccgatccttatcgatatggaaaaaaccaaggtcacccccggagggccggtggcgtatgccattgcacagcgcgtgttgca**

**Street14H ccgatccttatcgatatggaaaaaaccaaggtcacccccggagggccggtggcgtatgccattgcacagcgcgtgttgca**

**GauthierH**  **ccgatccttatcgatatggaaaaaaccaaggtcacccccggagggccggtggcgtatgccattgcacagcgcgtgttgca**

**SamoaDH**  **ccgatccttatcgatatggaaaaaaccaaggtcacccccggagggccggtggcgtatgccattgcacagcgcgtgttgca**

**CDC2H**  **ccgatccttatcgatatggaaaaaaccaaggtcacccccggagggccggtggcgtatgccattgcacagcgcgtgttgca**

**BosniaAH**  **ccgatccttatcgatatggaaaaaaccaaggtcacccccggagggccggtggcgtatgccattgcacagcgcgtgttgca**

**IraqBH**  **ccgatccttatcgatatggaaaaaaccaaggtcacccccggagggccggtggcgtatgccattgcacagcgcgtgttgca**

**Fribourg-BlancH** **ccgatccttatcgatatggaaaaaaccaaggtcacccccggagggccggtggcgtatgccattgcacagcgcgtgttgca**

**CuniculiAH**  **ctgatccttatcgatatggaaaaaaccaaggtcacccccggagggccggtggcgtatgccattgcacagcgcgtgttgca**

1290 1300 1310 1320 1330 1340 1350 1360

....|....|....|....|....|....|....|....|....|....|....|....|....|....|....|....|

**NicholsH**  **gctgcctgagtacgcgcagaagctcgatagtgtcaagaacggaatgtccgctaacggatcctctgtgcgggatattgcaa**

**Bal3H**  **gctgcctgagtacgcgcagaagctcgatagtgtcaagaacggaatgtccgctaacggatcctctgtgcgggatattgcaa**

**Sea81-4H**  **gctgcctgagtacgcgcagaagctcgatagtgtcaagaacggaatgtccgctaacggatcctctgtgcgggatattgcaa**

**MexicoAH**  **gctgcctgagtacgcgcagaagctcgatagtgtcaagaacggaatgtccgctaacggatcctctgtgcgggatattgcaa**

**Street14H gctgcctgagtacgcgcagaagctcgatagtgtcaagaacggaatgtccgctaacggatcctctgtgcgggatattgcaa**

**GauthierH**  **gctgcctgagtacgcgcagaagctcgatagtgtcaagaacggaatgtccgctaacggatcctctgtgcgggatattgcaa**

**SamoaDH**  **gctgcctgagtacgcgcagaagctcgatagtgtcaagaacggaatgtccgctaacggatcctctgtgcgggatattgcaa**

**CDC2H**  **gctgcctgagtacgcgcagaagctcgatagtgtcaagaacggaatgtccgctaacggatcctctgtgcgggatattgcaa**

**BosniaAH**  **gctgcctgagtacgcgcagaagctcgatagtgtcaagaacggaatgtccgctaacggatcctctgtgcgggatattgcaa**

**IraqBH**  **gctgcctgagtacgcgcagaagctcgatagtgtcaagaacggaatgtccgctaacggatcctctgtgcgggatattgcaa**

**Fribourg-BlancH** **gctgcctgagtacgcgcagaagctcgatagtgtcaagaacggaatgtccgctaacggatcctctgtgcgggatattgcaa**

**CuniculiAH**  **gctgcctgagtacgcgcagaagctcgatagtgtcaagaacggaatgtccgctaacggatcctctgtgcgggatattgcaa**

1370 1380 1390 1400 1410 1420 1430 1440

....|....|....|....|....|....|....|....|....|....|....|....|....|....|....|....|

**NicholsH**  **ccaaaatcgtacaagcagaacagacgaacccgacagttagttcaaaccccttgcttgcagcgctgttgacagtgctctgg**

**Bal3H**  **ccaaaatcgtacaagcagaacagacgaacccgacagttagttcaaaccccttgcttgcagcgctgttgacagtgctctgg**

**Sea81-4H**  **ccaaaatcgtacaagcagaacagacgaacccgacagttagttcaaaccccttgcttgcagcgctgttgacagtgctctgg**

**MexicoAH**  **ccaaaatcgtacaagcagaacagacgaacccgacagttagttcaaaccccttgcttgcagcgctgttgacagtgctctgg**

**Street14H ccaaaatcgtacaagcagaacagacgaacccgacagttagttcaaaccccttgcttgcagcgctgttgacagtgctctgg**

**GauthierH**  **ccaaaatcgtacaagcagaacagacgaacccgacagttagttcaaaccccttgcttgcagcgctgttgacagtgctctgg**

**SamoaDH**  **ccaaaatcgtacaagcagaacagacgaacccgacagttagttcaaaccccttgcttgcagcgctgttgacagtgctctgg**

**CDC2H**  **ccaaaatcgtacaagcagaacagacgaacccgacagttagttcaaaccccttgcttgcagcgctgttgacagtgctctgg**

**BosniaAH**  **ccaaaatcgtacaagcagaacagacgaacccgacagttagttcaaaccccttgcttgcagcgctgttgacagtgctctgg**

**IraqBH**  **ccaaaatcgtacaagcagaacagacgaacccgacagttagttcaaaccccttgcttgcagcgctgttgacagtgctctgg**

**Fribourg-BlancH** **ccaaagtcgtacaagcagaacagacgaacccgacagttagttcaaaccccttgcttgcagcgctgttgacagtgctctgg**

**CuniculiAH**  **ccaaaatcgtacaagcagaacagacgaacccgacagttagttcaaaccccttgcttgcagcgctgttgacagtgctctgg**

1450 1460 1470 1480 1490 1500 1510 1520

....|....|....|....|....|....|....|....|....|....|....|....|....|....|....|....|

**NicholsH**  **caacaagcgctggacacctacgcgctcgatgcactcctgactctgcaatggcgctggtttgcctgcggcgtgtacgtggc**

**Bal3H**  **caacaagcgctggacacctacgcgctcgatgcactcctgactctgcaatggcgctggtttgcctgcggcgtgtacgtggc**

**Sea81-4H**  **caacaagcgctggacacctacgcgctcgatgcactcctgactctgcaatggcgctggtttgcctgcggcgtgtacgtggc**

**MexicoAH**  **caacaagcgctggacacctacgcgctcgatgcactcctgactctgcaatggcgctggtttgcctgcggcgtgtacgtggc**

**Street14H caacaagcgctggacacctacgcgctcgatgcactcctgactctgcaatggcgctggtttgcctgcggcgtgtacgtggc**

**GauthierH**  **caacaagcgctggacacctacgcgctcgatgcactcctgactctgcaatggcgctggtttgcctgcggcgtgtacgtggc**

**SamoaDH**  **caacaagcgctggacacctacgcgctcgatgcactcctgactctgcaatggcgctggtttgcctgcggcgtgtacgtggc**

**CDC2H**  **caacaagcgctggacacctacgcgctcgatgcactcctgactctgcaatggcgctggtttgcctgcggcgtgtacgtggc**

**BosniaAH**  **caacaagcgctggacacctacgcgctcgatgcactcctgactctgcaatggcgctggtttgcctgcggcgtgtacgtggc**

**IraqBH**  **caacaagcgctggacacctacgcgctcgatgcactcctgactctgcaatggcgctggtttgcctgcggcgtgtacgtggc**

**Fribourg-BlancH** **caacaagcgctggacacctacgtgctcgatgcactcctgactctgcaatggcgctggtttgcctgcggcgtgtacgtggc**

**CuniculiAH**  **caacaagcgctggacacctacgcgctcgatgcactcctgactctgcaatggcgctggtttgcctgcggcgtgtacgtggc**

1530 1540 1550 1560 1570 1580 1590 1600

....|....|....|....|....|....|....|....|....|....|....|....|....|....|....|....|

**NicholsH**  **cactgctcctgcaagcgtgtttggggccatggtctttcctacgtatgggagcacacacacggacggcggcggctttctgc**

**Bal3H**  **cactgctcctgcaagcgtgtttggggccatggtctttcctacgtatgggagcacacacacggacggcggcggctttctgc**

**Sea81-4H**  **cactgctcctgcaagcgtgtttggggccatggtctttcctacgtatgggagcacacacacggacggcggcggctttctgc**

**MexicoAH**  **cactgctcctgcaagcgtgtttggggccatggtctttcctacgtatgggagcacacacacggacggcggcggctttctgc**

**Street14H cactgctcctgcaagcgtgtttggggccatggtctttcctacgtatgggagcacacacacggacggcggcggctttctgc**

**GauthierH**  **cactgctcctgcaagcgtgtttggggccatggtctttcctacgtatgggagcacacacatggacggcggcggctttctgc**

**SamoaDH**  **cactgctcctgcaagcgtgtttggggccatggtctttcctacgtatgggagcacacacatggacggcggcggctttctgc**

**CDC2H**  **cactgctcctgcaagcgtgtttggggccatggtctttcctacgtatgggagcacacacatggacggcggcggctttctgc**

**BosniaAH**  **cactgctcctgcaagcgtgtttggggccatggtctttcctacgtatgggagcacacacatggacggcggcggctttctgc**

**IraqBH**  **cactgctcctgcaagcgtgtttggggccatggtctttcctacgtatgggagcacacacatggacggcggcggctttctgc**

**Fribourg-BlancH** **cactgctcctgcaagcgtgtttggggccatggtctttcctacgtatgggagcacacacatggacggcggcggctttctgc**

**CuniculiAH**  **cactgctcctgcaagcgtgtttggggccatggtctttcctacgtatgggagcacacacacggacggcggcggctttctgc**

1610 1620 1630 1640 1650 1660 1670 1680

....|....|....|....|....|....|....|....|....|....|....|....|....|....|....|....|

**NicholsH**  **gggtagaaaccaaagcgggagacgcgtatacacaccttatagacggactggaagcagggatggacgtgcggtgctatatc**

**Bal3H**  **gggtagaaaccaaagcgggagacgcgtatacacaccttatagacggactggaagcagggatggacgtgcggtgctatatc**

**Sea81-4H**  **gggtagaaaccaaagcgggagacgcgtatacacaccttatagacggactggaagcagggatggacgtgcggtgctatatc**

**MexicoAH**  **gggtagaaaccaaagcgggagacgcgtatacacaccttatagacggactggaagcagggatggacgtgcggtgctatatc**

**Street14H gggtagaaaccaaagcgggagacgcgtatacacaccttatagacggactggaagcagggatggacgtgcggtgctatatc**

**GauthierH**  **gggtagaaaccaaagcgggagacgcgtatacacaccttatagacggactggaagcagggatggacgtgcggtgctatatc**

**SamoaDH**  **gggtagaaaccaaagcgggagacgcgtatacacaccttatagacggactggaagcagggatggacgtgcggtgctatatc**

**CDC2H**  **gggtagaaaccaaagcgggagacgcgtatacacaccttatagacggactggaagcagggatggacgtgcggtgctatatc**

**BosniaAH**  **gggtagaaaccaaagcgggagacgcgtatacacaccttatagacggactggaagcagggatggacgtgcggtgctatatc**

**IraqBH**  **gggtagaaaccaaagcgggagacgcgtatacacaccttatagacggactggaagcagggatggacgtgcggtgctatatc**

**Fribourg-BlancH** **gggtagaaaccaaagcgggagacgcgtatacacaccttatagacggactggaagcagggatggacgtgcggtgctatatc**

**CuniculiAH**  **gggtagaaaccaaagcaggagacgcgtatacacaccttatagacggactggaagcagggatggacgtgcggtgctatatt**

1690 1700 1710 1720 1730 1740 1750 1760

....|....|....|....|....|....|....|....|....|....|....|....|....|....|....|....|

**NicholsH**  **ccgcttacccacggcctgtacatagacaatggaaaaggatactacgtctctccaatgggtgtgtggaaactccctgacac**

**Bal3H**  **ccgcttacccacggcctgtacatagacaatggaaaaggatactacgtctctccaatgggtgtgtggaaactccctgacac**

**Sea81-4H**  **ccgcttacccacggcctgtacatagacaatggaaaaggatactacgtctctccaatgggtgtgtggaaactccctgacac**

**MexicoAH**  **ccgcttacccacggcctgtacatagacaatggaaaaggatactacgtctctccaatgggtgtgtggaaactccctgacac**

**Street14H ccgcttacccacggcctgtacatagacaatggaaaaggatactacgtctctccaatgggtgtgtggaaactccctgacac**

**GauthierH**  **ccgcttacccacggcctgtacatagacaatggaaaaggatactacgtctctccaatgggtgtgtggaaactccctgacac**

**SamoaDH**  **ccgcttacccacggcctgtacatagacaatggaaaaggatactacgtctctccaatgggtgtgtggaaactccctgacac**

**CDC2H**  **ccgcttacccacggcctgtacatagacaatggaaaaggatactacgtctctccaatgggtgtgtggaaactccctgacac**

**BosniaAH**  **ccgcttacccacggcctgtacatagacaatggaaaaggatactacgtctctccaatgggtgtgtggaaactccctgacac**

**IraqBH**  **ccgcttacccacggcctgtacatagacaatggaaaaggatactacgtctctccaatgggtgtgtggaaactccctgacac**

**Fribourg-BlancH** **ccgcttacccacggcctgtacatagacaatggaaaaggatactacgtctctccaatgggtgtgtggaaactccctgacac**

**CuniculiAH**  **ccgcttacccacggcctgtacatagacaatggaaaaggatactacgtctctccaatgggtgtgtggaaactccctgacac**

1770 1780 1790 1800 1810 1820 1830 1840

....|....|....|....|....|....|....|....|....|....|....|....|....|....|....|....|

**NicholsH**  **ccatatcaacttgcccgttatggggaaggtgtgggcgcgctaccttattccgcttggcgaaactgcatggcttaaacctt**

**Bal3H**  **ccatatcaacttgcccgttatggggaaggtgtgggcgcgctaccttattccgcttggcgaaactgcatggcttaaacctt**

**Sea81-4H**  **ccatatcaacttgcccgttatggggaaggtgtgggcacgctaccttattccgcttggcgaaactgcatggcttaaacctt**

**MexicoAH**  **ccatatcaacttgcccgttatggggaaggtgtgggcgcgctaccttattccgcttggcgaaactgcatggcttaaacctt**

**Street14H ccatatcaacttgcccgttatggggaaggtgtgggcgcgctaccttattccgcttggcgaaactgcatggcttaaacctt**

**GauthierH**  **ccatatcaacttgcccgttatggggaaggtgtgggcgcgctaccttattccgcttggcgaaactgcatggcttaaacctt**

**SamoaDH**  **ccatatcaacttgcccgttatggggaaggtgtgggcgcgctaccttattccgcttggcgaaactgcatggcttaaacctt**

**CDC2H**  **ccatatcaacttgcccgttatggggaaggtgtgggcgcgctaccttattccgcttggcgaaactgcatggcttaaacctt**

**BosniaAH**  **ccatatcaacttgcccgttatggggaaggtgtgggcgcgctaccttattccgcttggcgaaactgcatggcttaaacctt**

**IraqBH**  **ccatatcaacttgcccgttatggggaaggtgtgggcgcgctaccttattccgcttggcgaaactgcatggcttaaacctt**

**Fribourg-BlancH** **ccatatcgacttgcccgttatggggaaggtgtgggcgcgctaccttattccgcttggcgaaactgcatggcttaaacctt**

**CuniculiAH**  **ccatatcaacttgcccgttatggggaaggtgtgggcgcgctaccttattccgcttggcgaaactgcatggcttaaacctt**

1850 1860 1870 1880 1890 1900 1910 1920

....|....|....|....|....|....|....|....|....|....|....|....|....|....|....|....|

**NicholsH**  **cgcttgccgtatacggcaccacgaatcgcttcaactataatcttaagacagaaaatctagttcatgagcgttgcgtgcag**

**Bal3H**  **cgcttgccgtatacggcaccacgaatcgcttcaactataatcttaagacagaaaatctagttcatgagcgttgcgtgcag**

**Sea81-4H**  **cgcttgccgtatacggcaccacgaatcgcttcaactataatcttaagacagaaaatctagttcatgagcgttgcgtgcag**

**MexicoAH**  **cgcttgccgtatacggcaccacgaatcgcttcaactataatcttaagacagaaaatctagttcatgagcgttgcgtgcag**

**Street14H cgcttgccgtatacggcaccacgaatcgcttcaactataatcttaagacagaaaatctagttcatgagcgttgcgtgcag**

**GauthierH**  **cgcttgccgtatacggcaccacgaatcgcttcaactataatcttaagacagaaaatctagttcatgagcgttgcgtgcag**

**SamoaDH**  **cgcttgccgtatacggcaccacgaatcgcttcaactataatcttaagacagaaaatctagttcatgagcgttgcgtgcag**

**CDC2H**  **cgcttgccgtatacggcaccacgaatcgcttcaactataatcttaagacagaaaatctagttcatgagcgttgcgtgcag**

**BosniaAH**  **cgcttgccgtatacggcaccacgaatcgcttcaactataatcttaagacagaaaatctagttcatgagcgttgcgtgcag**

**IraqBH**  **cgcttgccgtatacggcaccacgaatcgcttcaactataatattaagacagaaaatctagttcatgagcgttgcgtgcag**

**Fribourg-BlancH** **cgcttgccgtatacggcaccacgaatcgcttcaactataatcttaagacagaaaatctagttcatgagcgttgcgtgcag**

**CuniculiAH**  **cgcttgccgtatacggcaccacgaatcgcttcaactataatcttaagacagaaaatctagttcgtgagcgttgcgtgcag**

1930 1940 1950 1960 1970 1980 1990 2000

....|....|....|....|....|....|....|....|....|....|....|....|....|....|....|....|

**NicholsH**  **taccaggtgggcctgacgctctctcccattgaaaaagtgacgcttcacgcacagtgggagcagggacagttggaaccaac**

**Bal3H**  **taccaggtgggcctgacgctctctcccattgaaaaagtgacgcttcacgcacagtgggagcagggacagttggaaccaac**

**Sea81-4H**  **taccaggtgggcctgacgctctctcccattgaaaaagtgacgcttcacgcacagtgggagcagggacagttggaaccaac**

**MexicoAH**  **taccaggtgggcctgacgctctctcccattgaaaaagtgacgcttcacgcacagtgggagcagggacagttggaaccaac**

**Street14H taccaggtgggcctgacgctctctcccattgaaaaagtgacgcttcacgcacagtgggagcagggacagttggaaccaac**

**GauthierH**  **taccaggtgggcctgacgctctctcccattgaaaaagtgacgcttcacgcacagtgggagcagggacagttggaaccaac**

**SamoaDH**  **taccaggtgggcctgacgctctctcccattgaaaaagtgacgcttcacgcacagtgggagcagggacagttggaaccaac**

**CDC2H**  **taccaggtgggcctgacgctctctcccattgaaaaagtgacgcttcacgcacagtgggagcagggacagttggaaccaac**

**BosniaAH**  **taccaggtgggcctgacgctctctcccattgaaaaagtgacgcttcacgcacagtgggagcagggacagttggaaccaac**

**IraqBH**  **taccaggtgggcctgacgctctctcccattgaaaaagtgacgcttcacgcacagtgggagcagggacagttggaaccaac**

**Fribourg-BlancH** **taccaggtgggcctgacgctctctcccattgaaaaagtgacgcttcacgcacagtgggagcagggacagttggaaccaac**

**CuniculiAH**  **taccaggtgggcctgacgctctctcccattgaaaaagtgacgcttcacgcacagtgggagcagggacagttggaaccaac**

2010 2020 2030 2040 2050 2060 2070 2080

....|....|....|....|....|....|....|....|....|....|....|....|....|....|....|....|

**NicholsH**  **gccctacatggttattacggaaaccgttacttcccgtaggtattttggcaccttcgtgtgcggaatgactattaactggt**

**Bal3H**  **gccctacatggttattacggaaaccgttacttcccgtaggtattttggcaccttcgtgtgcggaatgactattaactggt**

**Sea81-4H**  **gccctacatggttattacggaaaccgttacttcccgtaggtattttggcaccttcgtgtgcggaatgactattaactggt**

**MexicoAH**  **gccctacatggttattacggaaaccgttacttcccgtaggtattttggcaccttcgtgtgcggaatgactattaactggt**

**Street14H gccctacatggttattacggaaaccgttacttcccgtaggtattttggcaccttcgtgtgcggaatgactattaactggt**

**GauthierH**  **gccctacatggttattacggaaaccgttacttcccgtaggtattttggcaccttcgtgtgcggaatgactattaactggt**

**SamoaDH**  **gccctacatggttattacggaaaccgttacttcccgtaggtattttggcaccttcgtgtgcggaatgactattaactggt**

**CDC2H**  **gccctacatggttattacggaaaccgttacttcccgtaggtattttggcaccttcgtgtgcggaatgactattaactggt**

**BosniaAH**  **gccctacatggttattacggaaaccgttacttcccgtaggtattttggcaccttcgtgtgcggaatgactattaactggt**

**IraqBH**  **gccctacatggttattacggaaaccgttacttcccgtaggtattttggcaccttcgtgtgcggaatgactattaactggt**

**Fribourg-BlancH** **gccctacatggttattacggaaaccgttacttcccgtaggtattttggcaccttcgtgtgcggaatgactattaactggt**

**CuniculiAH**  **gccctacatggttattacggaaaccgttacttcccgtaggtactttggcaccttcgtgtgcggaatgactattaactggt**

..

**NicholsH**  **ag**

**Bal3H**  **ag**

**Sea81-4H**  **ag**

**MexicoAH**  **ag**

**Street14H ag**

**GauthierH**  **ag**

**SamoaDH**  **ag**

**CDC2H**  **ag**

**BosniaAH**  **ag**

**IraqBH**  **ag**

**Fribourg-BlancH** **ag**

**CuniculiAH**  **ag**

***1.8 tprJ* Locus**

10 20 30 40 50 60 70 80

....|....|....|....|....|....|....|....|....|....|....|....|....|....|....|....|

**NicholsJ**  **MGCMRWGSVLCVVVGVGASGGVLGQEFSPKLTGSATLEWGISYGKGVGSHGQAPGAVMGTGPYNLKHGFRTTNTVGVSFP**

**Street14J MGCMRWGSVLCVVVGVGASGGVLGQEFSPKLTGSATLEWGISYGKGVGSHGQAPGAVMGTGPYNLKHGFRTTNTVGVSFP**

**Bal3J**  **MGCMRWGSVLCVVVGVGASGGVLGQEFSPKLTGSATLEWGISYGKGVGSHGQAPGAVMGTGPYNLKHGFRTTNTVGVSFP**

**MexicoAJ**  **MGCMRWGSVLCVVVGVGASGGVLGQEFSPKLTGSATLEWGISYGKGVGSHGQAPGAVMGTGPYNLKHGFRTTNTVGVSFP**

**Sea81-4J**  **MGCMRWGSVLCVVVGVGASGGVLGQEFSPKLTGSATLEWGISYGKGVGSHGQAPGAVMGTGPYNLKHGFRTTNTVGVSFP**

**GauthierJ**  **MGCMRWGSVLCVVVGVGASGGVLGQEFSPKLTGSATLEWGISYGKGVGSHGQAPGAVMGTGPYNLKHGFRTTNTVGVSFP**

**SamoaDJ**  **MGCMRWGSVLCVVVGVGASGGVLGQEFSPKLTGSATLEWGISYGKGVGSHGQAPGAVMGTGPYNLKHGFRTTNTVGVSFP**

**CDC2J**  **MGCMRWGSVLCVVVGVGASGGVLGQEFSPKLTGSATLEWGISYGKGVGSHGQAPGAVMGTGPYNLKHGFRTTNTVGVSFP**

**Fribourg-BlancJ** **MGCMRWGSVLCVVVGVGASGGVLGQEFSPKLTGSATLEWGISYGKGVGSHGQAPGAVMGTGPYNLKHGFRTTNTVGVSFP**

**IraqBJ**  **MGCMRWGSVLCVVVGVGASGGVLGQEFSPKLTGSATLEWGISYGKGVGSHGQAPGAVMGTGPYNLKHGFRTTNTVGVSFP**

**BosniaAJ**  **MGCMRWGSVLCVVVGVGASGGVLGQEFSPKLTGSATLEWGISYGKGVGSHGQAPGAVMGTGPYNLKHGFRTTNTVGVSFP**

**CuniculiAJ**  **MGCMRWGSVLCVVVGVGASGGVLGQEFSPKLTGSATLEWGISYGKGVGSHDQAPGAVMGTGPYNLKHGFRTTNTVGVSFP**

90 100 110 120 130 140 150 160

....|....|....|....|....|....|....|....|....|....|....|....|....|....|....|....|

**NicholsJ**  **LVMRTTHTRRGQHPALYAELKVADLQADLSQGKAGFAVKRKGKVEATLHCYGAYLTIGKNPTFLTNFARLWKPWVTAQYQ**

**Street14J LVMRTTHTRRGQHPALYAELKVADLQADLSQGKAGFAVKRKGKVEATLHCYGAYLTIGKNPTFLTNFARLWKPWVTAQYQ**

**Bal3J**  **LVMRTTHTRRGQHPALYAELKVADLQADLSQGKAGFAVKRKGKVEATLHCYGAYLTIGKNPTFLTNFARLWKPWVTAQYQ**

**MexicoAJ**  **LVMRTTHTRRGQHPALYAELKVADLQADLSQGKAGFAVKRKGKVEATLHCYGAYLTIGKNPTFLTNFARLWKPWVTAQYQ**

**Sea81-4J**  **LVMRTTHTRRGQHPALYAELKVADLQADLSQGKAGFAIKRKGKVEATLHCYGAYLTIGKNPTFLTNFARLWKPWVTAQYQ**

**GauthierJ**  **LVMRTTHTRRGQHPALYAELKVADLQADLSQGKAGFAVKRKGKVEATLHCYGAYLTIGKNPTFLTNFARLWKPWVTAQYQ**

**SamoaDJ**  **LVMRTTHTRRGQHPALYAELKVADLQADLSQGKAGFAVKRKGKVEATLHCYGAYLTIGKNPTFLTNFARLWKPWVTAQYQ**

**CDC2J**  **LVMRTTHTRRGQHPALYAELKVADLQADLSQGKAGFAVKRKGKVEATLHCYGAYLTIGKNPTFLTNFARLWKPWVTAQYQ**

**Fribourg-BlancJ** **LVMRTTHTRRGQHPALYAELKVADLQADLSQGKAGFAVKRKGKVEATLHCYGAYLTIGKNPTFLTNFARLWKPWVTAQYQ**

**IraqBJ**  **LVMRTTHTRRGQHPALYAELKVADLQADLSQGKVGFAVKRKGKVEATLHCYGAYLTIGKNPTFLTNFARLWKPWVTAQYQ**

**BosniaAJ**  **LVMRTTHTRRGQHPALYAELKVADLQADLSQGKVGFAVKRKGKVEATLHCYGAYLTIGKNPTFLTNFARLWKPWVTAQYQ**

**CuniculiAJ**  **LVMRTTHTRRGQHPALYAELKVADLQADLSQGKAGFDFKRKGKVEATLHCYGAYLTIGKNPTFLTNFARLWKPWVTAQYQ**

170 180 190 200 210 220 230 240

....|....|....|....|....|....|....|....|....|....|....|....|....|....|....|....|

**NicholsJ**  **EDAVQYAPGFGGLGGKVGYRAQDIGGSGVSLDVGFLSFASNGAWDSTDPTHSKYGFGADLKLMYARAGHPLCTVELASNV**

**Street14J EDAVQYAPGFGGLGGKVGYRAQDIGGSGVSLDVGFLSFASNGAWDSTDPTHSKYGFGADLKLMYARAGHPLCTVELASNV**

**Bal3J**  **EDAVQYAPGFGGLGGKVGYRAQDIGGSGVSLDVGFLSFASNGAWDSTDPTHSKYGFGADLKLMYARAGHPLCTVELASNV**

**MexicoAJ**  **EDAVQYAPGFGGLGGKVGYRAQDIGGSGVSLDVGFLSFASNGAWDSTDPTHSKYGFGADLKLMYARAGHPLCTVELASNV**

**Sea81-4J**  **EDAVQYAPGFGGLGGKVGYRAQDIGGSGVSLDVGFLSFASNGAWDSTDPTHSKYGFGADLKLMYARAGHPLCTVELASNV**

**GauthierJ**  **EDAVQYAPGFGGLGGKVGYRAQDIGGSGVSLDVGFLSFASNGAWDSTDPTHSKYGFGADLKLMYARAGHPLCTVELASNV**

**SamoaDJ**  **EDAVQYAPGFGGLGGKVGYRAQDIGGSGVSLDVGFLSFASNGAWDSTDPTHSKYGFGADLKLMYARAGHPLCTVELASNV**

**CDC2J**  **EDAVQYAPGFGGLGGKVGYRAQDIGGSGVSLDVGFLSFASNGAWDSTDPTHSKYGFGADLKLMYARAGHPLCTVELASNV**

**Fribourg-BlancJ** **EDAVQYAPGFGGLGGKVGYRAQDIGGSGVSLDVGFLSFASNGAWDSTDPTHSKYGFGADLKLMYARAGHPLCTVELASNV**

**IraqBJ**  **EDAVQYAPGFGGLGGKVGYRAQDIGGSGVSLDVGFLSFASNGAWDSTDPTHSKYGFGADLKLMYARAGHPLCTVELASNV**

**BosniaAJ**  **EDAVQYAPGFGGLGGKVGYRAQDIGGSGVSLDVGFLSFASNGAWDSTDPTHSKYGFGADLKLMYARAGHPLCTVELASNV**

**CuniculiAJ**  **EDEVQYAPGFGGLGGKVGYRAQDIGGSGVSLDVGFLSFASNGAWDSTDTTHSKYGFGGRLEANVCACRTPSVHGRACQQC**

250 260 270 280 290 300 310 320

....|....|....|....|....|....|....|....|....|....|....|....|....|....|....|....|

**NicholsJ**  **TLEDGYLIGAQKDANNQNKDKLLWNVGGRLTLEPGAGFRFSFALDAGNQHQSAQDFQNRTQRAQSELTALSNNLFQGESQ**

**Street14J TLEDGYLIGAQKDANNQNKDKLLWNVGGRLTLEPGAGFRFSFALDAGNQHQSAQDFQNRTQRAQSELTALSNNLFQGESQ**

**Bal3J**  **TLEDGYLIGAQKDANNQNKDKLLWNVGGRLTLEPGAGFRFSFALDAGNQHQSAQDFQNRTQRAQSELTALSNNLFQGESQ**

**MexicoAJ**  **TLEDGYLIGAQKDANNQNKDKLLWNVGGRLTLEPGAGFRFSFALDAGNQHQSAQDFQNRTQRAQSELTALSNNLFQGESQ**

**Sea81-4J**  **TLEDGYLIGAQKDANNQNKDKLLWNVGGRLTLEPGAGFRFSFALDAGNQHQSAQDFQNRTQRAQSELTALSNNLFQGESQ**

**GauthierJ**  **TLEDGYLIGAQKDANNQNKDKLLWNVGGRLTLEPGAGFRFSFALDAGNQHQSAQDFQNRTQRAQSELTALSNNLFQGESQ**

**SamoaDJ**  **TLEDGYLIGAQKDANNQNKDKLLWNVGGRLTLEPGAGFRFSFALDAGNQHQSAQDFQNRTQRAQSELTALSNNLFQGESQ**

**CDC2J**  **TLEDGYLIGAQKDANNQNKDKLLWNVGGRLTLEPGAGFRFSFALDAGNQHQSAQDFQNRTQRAQSELTALSNNLFQGESQ**

**Fribourg-BlancJ** **TLEDGYLIGAQKDANNQNKDKLLWNVGGRLTLEPGAGFRFSFALDAGNQHQSAQDFQNRTQRAQSELTALSNNLFQGESQ**

**IraqBJ**  **TLEDGYLIGAQKDANNQNKDKLLWNVGGRLTLEPGAGFRFSFALDAGNQHQSAQDFQNRTQRAQSELTALSNNLFQGESQ**

**BosniaAJ**  **TLEDGYLIGAQKDANNQNKDKLLWNVGGRLTLEPGAGFRFSFALDAGNQHQSAQDFQNRTQRAQSELTALSNNLFQGESQ**

**CuniculiAJ**  **YAGRRIPHRCAEGRKQSEQG------------------------------------------------------------**

330 340 350 360 370 380 390 400

....|....|....|....|....|....|....|....|....|....|....|....|....|....|....|....|

**NicholsJ**  **KQEAWVTQVVQQATQTVTAGVRSALESRGTTYINALEAVQPNPAKPTGKVVQNLHTPQGSPPNLPPLPALPAFSLMGQVL**

**Street14J KQEAWVTQVVQQATQTVTAGVRSALESRGTTYINALEAVQPNPAKPTGKVVQNLHTPQGSPPNLPPLPALPAFSLMGQVL**

**Bal3J**  **KQEAWVTQVVQQATQTVTAGVRSALESRGTTYINALEAVQPNPAKPTGKVVQNLHTPQGSPPNLPPLPALPAFSLMGQVL**

**MexicoAJ**  **KQEAWVTQVVQQATQTVTAGVRSALESRGTTYINALEAVQPNPAKPTGKVVQNLHTPQGSPPNLPPLPALPAFSLMGQVL**

**Sea81-4J**  **KQEAWLDEYAKKVLDAVTAATETALQSRGNAYITAVSNVKVTPPV-AATLLTNLKVFITDPPTPSPLPALPAFSLMGQVL**

**GauthierJ**  **KQEAWLDEYAKKVLDAVTAATETALQSRGNAYITAVSNVKVTPPV-AATLLTNLKVFITDPPTPSPLPALPAFSLMGQVL**

**SamoaDJ**  **KQEAWLDEYAKKVLDAVTAATETALQSRGNAYITAVSNVKVTPPV-AATLLTNLKVFITDPPTPSPLPALPAFSLMGQVL**

**CDC2J**  **KQEAWLDEYAKKVLDAVTAATETALQSRGNAYITAVSNVKVTPPV-AATLLTNLKVFITDPPTPSPLPALPAFSLMGQVL**

**Fribourg-BlancJ** **KQEAWLDEYAKKVLDAVTAATETALQSRGNAYITAVSNVKVTPPV-AATLLTNLKVFITDPPTPSPLPALPAFSLMGQVL**

**IraqBJ**  **KQEAWLDEYAKKVLDAVTAATETALQSRGNAYITAVSNVKVTPPV-AATLLTNLKVFITDPPTPSPLPALPAFSLMGQVL**

**BosniaAJ**  **KQEAWLDEYAKKVLDAVTAATETALQSRGNAYITAVSNVKVTPPV-AATLLTNLKVFITDPPTPSPLPALPAFSLMGQVL**

**CuniculiAJ**  **--------------------------------------------------------------------------------**

410 420 430 440 450 460 470 480

....|....|....|....|....|....|....|....|....|....|....|....|....|....|....|....|

**NicholsJ**  **LQYDAEQVVKGFEQVQTQIVTEINQKVQAAVAKNNANMQAVGGSLGDTARMVGEALIKQQLSRKQNSILTMVSVQDEVKQ**

**Street14J LQYDAEQVVKGFEQVQTQIVTEINQKVQAAVAKNNANMQAVGGSLGDTARMVGEALIKQQLSRKQNSILTMVSVQDEVKQ**

**Bal3J**  **LQYDAEQVVKGFEQVQTQIVTEINQKVQAAVAKNNANMQAVGGSLGDTARMVGEALIKQQLSRKQNSILTMVSVQDEVKQ**

**MexicoAJ**  **LQYDAEQVVKGFEQVQTQIVTEINQKVQAAVAKNNANMQAVGGSLGDTARMVGEALIKQQLSRKQNSILTMVSVQDEVKQ**

**Sea81-4J**  **LQYDAEQVVKGFEQVQTQIVAEINQKVQAAVAQSKAAAQAFINGLTKAIEDVADALLAPH-KGNPMSLFNLPDQQKLLKD**

**GauthierJ**  **LQYDAEQVVKGFEQVQTQIVAEINQKVQAAVAQSKAAAQAFINGLTKAIEDVADALLAPH-KGNPMSLFNLPDQQKLLKD**

**SamoaDJ**  **LQYDAEQVVKGFEQVQTQIVAEINQKVQAAVAQSKAAAQAFINGLTKAIEDVADALLAPH-KGNPMSLFNLPDQQKLLKD**

**CDC2J**  **LQYDAEQVVKGFEQVQTQIVAEINQKVQAAVAQSKAAAQAFINGLTKAIEDVADALLAPH-KGNPMSLFNLPDQQKLLKD**

**Fribourg-BlancJ** **LQYDAEQVVKGFEQVQTQIVAEINQKVQAAVAQSKAAAQAFINGLTKAIEDVADALLAPH-KGNPMSLFNLPDQQKLLKD**

**IraqBJ**  **LQYDAEQVVKGFEQVQTQIVAEINQKVQAAVAQSKAAAQAFINGLTKAIEDVADALLAPH-KGNPMSLFNLPDQQKLLKD**

**BosniaAJ**  **LQYDAEQVVKGFEQVQTQIVAEINQKVQAAVAQSKAAAQAFINGLTKAIEDVADALLAPH-KGNPMSLFNLPDQQKLLKD**

**CuniculiAJ**  **--------------------------------------------------------------------------------**

490 500 510 520 530 540 550 560

....|....|....|....|....|....|....|....|....|....|....|....|....|....|....|....|

**NicholsJ**  **DLADLVPMMRTEITAFFASVQQHITEEVKKKTDALNAGQQIRQAIQNLRASAWRAFLMGVSAVCLYLDTYNVAFDALFTA**

**Street14J DLADLVPMMRTEITAFFASVQQHITEEVKKKTDALNAGQQIRQAIQNLRASAWRAFLMGVSAVCLYLDTYNVAFDALFTA**

**Bal3J**  **DLADLVPMMRTEITAFFASVQQHITEEVKKKTDALNAGQQIRQAIQNLRASAWRAFLMGVSAVCLYLDTYNVAFDALFTA**

**MexicoAJ**  **DLADLVPMMRTEITAFFASVQQHITEEVKKKTDALNAGQQIRQAIQNLRASAWRAFLMGVSAVCLYLDTYNVAFDALLTA**

**Sea81-4J**  **DLADLIPKLTAEATKFFTEGQTFVTEEVKKKTDALNAGQQIRQAIQNLRASAWRAFLMGVSAVCLYLDTYNVAFDALLTA**

**GauthierJ**  **DLADLIPKLTAEATKFFTEGQTFVTEEVKKKTDALDAGQQIRQAIQNLRASAWRAFLMGVSAVCLYLDTYNVAFDALLTA**

**SamoaDJ**  **DLADLIPKLTAEATKFFTEGQTFVTEEVKKKTDALDAGQQIRQAIQNLRASAWRAFLMGVSAVCLYLDTYNVAFDALLTA**

**CDC2J**  **DLADLIPKLTAEATKFFTEGQTFVTEEVKKKTDALDAGQQIRQAIQNLRASAWRAFLMGVSAVCLYLDTYNVAFDALLTA**

**Fribourg-BlancJ** **DLADLIPKLTAEATKFFTEGQTFVTEEVKKKTDALDAGQQIRQAIQNLRASAWRAFLMGVSAVCLYLDTYNVAFDALLTA**

**IraqBJ**  **DLADLIPKLTAEATKFFTEGQTFVTEEVKKKTDALDAGQQIRQAIQNLRASAWRAFLMGVSAVCLYLDTYNVAFDALLTA**

**BosniaAJ**  **DLADLIPKLTAEATKFFTEGQTFVTEEVKKKTDALDAGQQIRQAIQNLRASAWRAFLMGVSAVCLYLDTYNVAFDALLTA**

**CuniculiAJ**  **--------------------------------------------------------------------------------**

570 580 590 600 610 620 630 640

....|....|....|....|....|....|....|....|....|....|....|....|....|....|....|....|

**NicholsJ**  **QWKWLSSGIYFATAPANVFGTRVLDNTIASCGDFAGFLKLETKSGDPYTHLLTGLDAGVETRVYIPLTHDLYKNNNGNPL**

**Street14J QWKWLSSGIYFATAPANVFGTRVLDNTIASCGDFAGFLKLETKSGDPYTHLLTGLDAGVETRVYIPLTHDLYKNNNGNPL**

**Bal3J**  **QWKWLSSGIYVATAPANVFGTRVLDNTIASCGDFAGFLKLETKSGDPYTHLLTGLDAGVETRVYIPLTHDLYKNNNGNPL**

**MexicoAJ**  **QWKWLSSGIYVATAPANVFGTRVLDNTIASCGDFAGFLKLETKSGDPYTHLLTGLDAGVETRVYIPLTHDLYKNNNGNPL**

**Sea81-4J**  **QWKWLSSGIYVATAPANVFGTRVLDNTIASCGDFAGFLKLETKSGDPYTHLLTGLDAGVETRVYIPLTHDLYKNNNGNPL**

**GauthierJ**  **QWKWLSSGIYFATAPANVFGTRVLDNTIASCGDFAGFLKLETKSGDPYTHLLTGLDAGVETRVYIPLTHDLYKNNNGNPL**

**SamoaDJ**  **QWKWLSSGIYFATAPANVFGTRVLDNTIASCGDFAGFLKLETKSGDPYTHLLTGLDAGVETRVYIPLTHDLYKNNNGNPL**

**CDC2J**  **QWKWLSSGIYFATAPANVFGTRVLDNTIASCGDFAGFLKLETKSGDPYTHLLTGLDAGVETRVYIPLTHDLYKNNNGNPL**

**Fribourg-BlancJ** **QWKWLSSGIYFATAPANVFGTRVLDNTIASCGDFAGFLKLETKSGDPYTHLLTGLDAGVETRVYIPLTHDLYKNNNGNPL**

**IraqBJ**  **QWKWLSSGIYFATAPANVFGTRVLDNTIASCGDFAGFLKLETKSGDPYTHLLTGLDAGVETRVYIPLTHDLYKNNNGNPL**

**BosniaAJ**  **QWKWLSSGIYFATAPANVFGTRVLDNTIASCGDFAGFLKLETKSGDPYTHLLTGLDAGVETRVYIPLTHDLYKNNNGNPL**

**CuniculiAJ**  **--------------------------------------------------------------------------------**

650 660 670 680 690 700 710 720

....|....|....|....|....|....|....|....|....|....|....|....|....|....|....|....|

**NicholsJ**  **PSGGSSGHIGLPVVGKAWCSYRIPVQDYGWVKPSVTVHASTNRAHLNAPAAGGAVGATYLTKEYCAQLRAGISASLIEKT**

**Street14J PSGGSSGHIGLPVVGKAWCSYRIPVQDYGWVKPSVTVHASTNRAHLNAPAAGGAVGATYLTKEYCAQLRAGISASLIEKT**

**Bal3J**  **PSGGSSGHIGLPVVGKAWCSYRIPVQDYGWVKPSVTVHASTNRAHLNAPAAGGAVGATYLTKEYCAQLRAGISASLIEKT**

**MexicoAJ**  **PSGGSSGHIGLPVVGKAWCSYRIPVQDYGWVKPSVTVHASTNRAHLNAPAAGGAVGATYLTKEYCAQLRAGISASLIEKT**

**Sea81-4J**  **PSGGSSGHIGLPVVGKAWCSYRIPVQDYGWVKPSVTVHASTNRAHLNAPAAGGAVGATYLTKEYCAQLRAGISASLIEKT**

**GauthierJ**  **PSGGSSGHIGLPVVGKAWCSYRIPVQDYGWVKPSVTVHASTNRAHLNAPAAGGAVGATYLTKEYCAQLRAGVSASLIEKT**

**SamoaDJ**  **PSGGSSGHIGLPVVGKAWCSYRIPVQDYGWVKPSVTVHASTNRAHLNAPAAGGAVGATYLTKEYCAQLRAGVSASLIEKT**

**CDC2J**  **PSGGSSGHIGLPVVGKAWCSYRIPVQDYGWVKPSVTVHASTNRAHLNAPAAGGAVGATYLTKEYCAQLRAGVSASLIEKT**

**Fribourg-BlancJ** **PSGGSSGHIGLPVVGKAWCSYRIPVQDYGWVKPSVTVHASTNRAHLNAPAAGGAVGATYLTKEYCAQLRAGVSASLIEKT**

**IraqBJ**  **PSGGSSGHIGLPVVGKAWCSYRIPVQDYGWVKPSVTVHASTNRAHLNAPAAGGAVGATYLTKEYCAQLRAGVSASLIEKT**

**BosniaAJ**  **PSGGSSGHIGLPVVGKAWCSYRIPVQDYGWVKPSVTVHASTNRAHLNAPAAGGAVGATYLTKEYCAQLRAGVSASLIEKT**

**CuniculiAJ**  **--------------------------------------------------------------------------------**

730 740 750

....|....|....|....|....|....|....|...

**NicholsJ**  **VFSLDWEQGMLSDVPYLLVSECLTQGIGRIVCGVTLSW**

**Street14J VFSLDWEQGMLSDVPYLLVSECLTQGIGRIVCGVTLSW**

**Bal3J**  **VFSLDWEQGMLSDVPYLLVSECLTQGIGRIVCGVTLSW**

**MexicoAJ**  **VFSLDWEQGMLSDVPYLLVSECLTQGIGRIVCGVTLSW**

**Sea81-4J**  **VFSLDWEQGMLSDVPYLLVSECLTQGIGRIVCGVTLSW**

**GauthierJ**  **VFSLDWEQGMLSDVPYLLVSECLTQGIGRIVCGVTLSW**

**SamoaDJ**  **VFSLDWEQGMLSDVPYLLVSECLTQGIGRIVCGVTLSW**

**CDC2J**  **VFSLDWEQGMLSDVPYLLVSECLTQGIGRIVCGVTLSW**

**Fribourg-BlancJ** **VFSLDWEQGMLSDVPYLLVSECLTQGIGRIVCGVTLSW**

**IraqBJ**  **VFSLDWEQGMLSDVPYLLVSECLTQGIGRIVCGVTLSW**

**BosniaAJ**  **VFSLDWEQGMLSDVPYLLVSECLTQGIGRIVCGVTLSW**

**CuniculiAJ**  **--------------------------------------**

10 20 30 40 50 60 70 80

....|....|....|....|....|....|....|....|....|....|....|....|....|....|....|....|

**NicholsJ**  **atggggtgcatgcggtgggggagtgtgctgtgtgtggtggtgggggtaggagcgagcgggggagtgctcggacaggagtt**

**Street14J atggggtgcatgcggtgggggagtgtgctgtgtgtggtggtgggggtaggagcgagcgggggagtgctcggacaggagtt**

**Bal3J**  **atggggtgcatgcggtgggggagtgtgctgtgtgtggtggtgggggtaggagcgagcgggggagtgctcggacaggagtt**

**MexicoAJ**  **atggggtgcatgcggtgggggagtgtgctgtgtgtggtggtgggggtaggagcgagcgggggagtgctcggacaggagtt**

**Sea81-4J**  **atggggtgcatgcggtgggggagtgtgctgtgtgtggtggtgggggtaggagcgagcgggggagtgctcggacaggagtt**

**GauthierJ**  **atggggtgcatgcggtgggggagtgtgctgtgtgtggtggtgggggtaggagcgagcgggggagtgctcggacaggagtt**

**CDC2J**  **atggggtgcatgcggtgggggagtgtgctgtgtgtggtggtgggggtaggagcgagcgggggagtgctcggacaggagtt**

**Fribourg-BlancJ** **atggggtgcatgcggtgggggagtgtgctgtgtgtggtggtgggggtaggagcgagcgggggagtgctcggacaggagtt**

**IraqBJ**  **atggggtgcatgcggtgggggagtgtgctgtgtgtggtggtgggggtaggagcgagcgggggagtgctcggacaggagtt**

**BosniaAJ**  **atggggtgcatgcggtgggggagtgtgctgtgtgtggtggtgggggtaggagcgagcgggggagtgctcggacaggagtt**

**CuniculiAJ**  **atggggtgcatgcggtgggggagtgtgctgtgtgtggtggtgggggtaggagcgagcgggggagtgcttggacaggagtt**

90 100 110 120 130 140 150 160

....|....|....|....|....|....|....|....|....|....|....|....|....|....|....|....|

**NicholsJ**  **ttccccgaagctaactggctctgccacacttgagtggggcatcagctatggcaagggggtaggcagtcatggccaggccc**

**Street14J ttccccgaagctaactggctctgccacacttgagtggggcatcagctatggcaagggggtaggcagtcatggccaggccc**

**Bal3J**  **ttccccgaagctaactggctctgccacacttgagtggggcatcagctatggcaagggggtaggcagtcatggccaggccc**

**MexicoAJ**  **ttccccgaagctaactggctctgccacacttgagtggggcatcagctatggcaagggggtaggcagtcatggccaggccc**

**Sea81-4J**  **ttccccgaagctaactggctctgccacacttgagtggggcatcagctatggcaagggggtaggcagtcatggccaggccc**

**GauthierJ**  **ttccccgaagctaactggctctgccacacttgagtggggcatcagctatggcaagggggtaggcagtcatggccaggccc**

**CDC2J**  **ttccccgaagctaactggctctgccacacttgagtggggcatcagctatggcaagggggtaggcagtcatggccaggccc**

**Fribourg-BlancJ** **ttccccgaagctaactggctctgccacacttgagtggggcatcagctatggcaagggggtaggcagtcatggccaggccc**

**IraqBJ**  **ttccccgaagctaactggctctgccacacttgagtggggcatcagctatggcaagggggtaggcagtcatggccaggccc**

**BosniaAJ**  **ttccccgaagctaactggctctgccacacttgagtggggcatcagctatggcaagggggtaggcagtcatggccaggccc**

**CuniculiAJ**  **ttccccgaagctaactggctccgccacacttgagtggggcatcagctatggcaagggggtaggcagtcacgaccaggccc**

170 180 190 200 210 220 230 240

....|....|....|....|....|....|....|....|....|....|....|....|....|....|....|....|

**NicholsJ**  **ctggtgcagttatgggcaccggtccctacaatctgaagcacgggtttcgtactaccaacacggtgggagtatcctttccc**

**Street14J ctggtgcagttatgggcaccggtccctacaatctgaagcacgggtttcgtactaccaacacggtgggagtatcctttccc**

**Bal3J**  **ctggtgcagttatgggcaccggtccctacaatctgaagcacgggtttcgtactaccaacacggtgggagtatcctttccc**

**MexicoAJ**  **ctggtgcagttatgggcaccggtccctacaatctgaagcacgggtttcgtactaccaacacggtgggagtatcctttccc**

**Sea81-4J**  **ctggtgcagttatgggcaccggtccctacaatctgaagcacgggtttcgtactaccaacacggtgggagtatcctttccc**

**GauthierJ**  **ctggtgcagttatgggcaccggtccctacaatctgaagcacgggtttcgtactaccaacacggtgggagtatcctttccc**

**CDC2J**  **ctggtgcagttatgggcaccggtccctacaatctgaagcacgggtttcgtactaccaacacggtgggagtatcctttccc**

**Fribourg-BlancJ** **ctggtgcagttatgggcaccggtccctacaatctgaagcacgggtttcgtactaccaacacggtgggagtatcctttccc**

**IraqBJ**  **ctggtgcagttatgggcaccggtccctacaatctgaagcacgggtttcgtactaccaacacggtgggagtatcctttccc**

**BosniaAJ**  **ctggtgcagttatgggcaccggtccctacaatctgaagcacgggtttcgtactaccaacacggtgggagtatcctttccc**

**CuniculiAJ**  **ctggtgcagttatgggcaccggtccctacaatctgaagcatgggtttcgtactaccaacacggtgggagtatcctttccc**

250 260 270 280 290 300 310 320

....|....|....|....|....|....|....|....|....|....|....|....|....|....|....|....|

**NicholsJ**  **ctggttatgcgcaccacccacacgcgccgtgggcagcacccggcactgtatgcggagctgaaggtggcggacctgcaggc**

**Street14J ctggttatgcgcaccacccacacgcgccgtgggcagcacccggcactgtatgcggagctgaaggtggcggacctgcaggc**

**Bal3J**  **ctggttatgcgcaccacccacacgcgccgtgggcagcacccggcactgtatgcggagctgaaggtggcggacctgcaggc**

**MexicoAJ**  **ctggttatgcgcaccacccacacgcgccgtgggcagcacccggcactgtatgcggagctgaaggtggcggacctgcaggc**

**Sea81-4J**  **ctggttatgcgcaccacccacacgcgccgtgggcagcacccggcactgtatgcggagctgaaggtggcggacctgcaggc**

**GauthierJ**  **ctggttatgcgcaccacccacacgcgccgtgggcagcacccggcactgtatgcggagctgaaggtggcggacctgcaggc**

**CDC2J**  **ctggttatgcgcaccacccacacgcgccgtgggcagcacccggcactgtatgcggagctgaaggtggcggacctgcaggc**

**Fribourg-BlancJ** **ctggttatgcgcaccacccacacgcgccgtgggcagcacccggcactgtatgcggagctgaaggtggcggacctgcaggc**

**IraqBJ**  **ctggttatgcgcaccacccacacgcgccgtgggcagcacccggcactgtatgcggagctgaaggtggcggacctgcaggc**

**BosniaAJ**  **ctggttatgcgcaccacccacacgcgccgtgggcagcacccggcactgtatgcggagctgaaggtggcggacctgcaggc**

**CuniculiAJ**  **ctggttatgcgcaccacccacacgcgccgtgggcagcacccggcactgtatgcggagctgaaggtagcggacctgcaggc**

330 340 350 360 370 380 390 400

....|....|....|....|....|....|....|....|....|....|....|....|....|....|....|....|

**NicholsJ**  **ggacctgagtcaggggaaggcaggttttgccgttaagcgcaaggggaaggtagaggcgacactacactgttatggggcct**

**Street14J ggacctgagtcaggggaaggcaggttttgccgttaagcgcaaggggaaggtagaggcgacactacactgttatggggcct**

**Bal3J**  **ggacctgagtcaggggaaggcaggttttgccgttaagcgcaaggggaaggtagaggcgacactacactgttatggggcct**

**MexicoAJ**  **ggacctgagtcaggggaaggcaggttttgccgttaagcgcaaggggaaggtagaggcgacactacactgttatggggcct**

**Sea81-4J**  **ggacctgagtcaggggaaggcaggttttgccattaagcgcaaggggaaggtagaggcgacactacactgttatggggcct**

**GauthierJ**  **ggacctgagtcaggggaaggcaggttttgccgttaagcgcaaggggaaggtagaggcgacactacactgttatggggcct**

**CDC2J**  **ggacctgagtcaggggaaggcaggttttgccgttaagcgcaaggggaaggtagaggcgacactacactgttatggggcct**

**Fribourg-BlancJ** **ggacctgagtcaggggaaggcaggttttgccgttaagcgcaaggggaaggtagaggcgacactacactgttatggggcct**

**IraqBJ**  **ggacctgagtcaggggaaggtaggttttgccgttaagcgcaaggggaaggtagaggcgacactacactgttatggggcct**

**BosniaAJ**  **ggacctgagtcaggggaaggtaggttttgccgttaagcgcaaggggaaggtagaggcgacactacactgttatggggcct**

**CuniculiAJ**  **ggacctgagtcaggggaaggcaggttttgactttaagcgcaaggggaaggtggaggcgacgctgcactgttatggggcct**

410 420 430 440 450 460 470 480

....|....|....|....|....|....|....|....|....|....|....|....|....|....|....|....|

**NicholsJ**  **acctgacgattgggaagaaccccacgtttctgacgaactttgcccggctgtggaagccgtgggtgacagcgcagtaccag**

**Street14J acctgacgattgggaagaaccccacgtttctgacgaactttgcccggctgtggaagccgtgggtgacagcgcagtaccag**

**Bal3J**  **acctgacgattgggaagaaccccacgtttctgacgaactttgcccggctgtggaagccgtgggtgacagcgcagtaccag**

**MexicoAJ**  **acctgacgattgggaagaaccccacgtttctgacgaactttgcccggctgtggaagccgtgggtgacagcgcagtaccag**

**Sea81-4J**  **acctgacgattgggaagaaccccacgtttctgacgaactttgcccggctgtggaagccgtgggtgacagcgcagtaccag**

**GauthierJ**  **acctgacgattgggaagaaccccacgtttctgacgaactttgcccggctgtggaagccgtgggtgacagcgcagtaccag**

**CDC2J**  **acctgacgattgggaagaaccccacgtttctgacgaactttgcccggctgtggaagccgtgggtgacagcgcagtaccag**

**Fribourg-BlancJ** **acctgacgattgggaagaaccccacgtttctgacgaactttgcccggctgtggaagccgtgggtgacagcgcagtaccag**

**IraqBJ**  **acctgacgattgggaagaaccccacgtttctgacgaactttgcccggctgtggaagccgtgggtgacagcgcagtaccag**

**BosniaAJ**  **acctgacgattgggaagaaccccacgtttctgacgaactttgcccggctgtggaagccgtgggtgacagcgcagtaccag**

**CuniculiAJ**  **acctgacgattgggaagaaccccacgtttctgacgaactttgcccggctgtggaagccgtgggtgacagcgcagtaccag**

490 500 510 520 530 540 550 560

....|....|....|....|....|....|....|....|....|....|....|....|....|....|....|....|

**NicholsJ**  **gaggatgcggtacagtatgcgccggggtttgggggtttaggcggcaaggttgggtatcgggcacaggacattgggggcag**

**Street14J gaggatgcggtacagtatgcgccggggtttgggggtttaggcggcaaggttgggtatcgggcacaggacattgggggcag**

**Bal3J**  **gaggatgcggtacagtatgcgccggggtttgggggtttaggcggcaaggttgggtatcgggcacaggacattgggggcag**

**MexicoAJ**  **gaggatgcggtacagtatgcgccggggtttgggggtttaggcggcaaggttgggtatcgggcacaggacattgggggcag**

**Sea81-4J**  **gaggatgcggtacagtatgcgccggggtttgggggtttaggcggcaaggttgggtatcgggcacaggacattgggggcag**

**GauthierJ**  **gaggatgcggtacagtatgcgccggggtttgggggtttaggcggcaaggttgggtatcgggcacaggacattgggggcag**

**CDC2J**  **gaggatgcggtacagtatgcgccggggtttgggggtttaggcggcaaggttgggtatcgggcacaggacattgggggcag**

**Fribourg-BlancJ** **gaggatgcggtacagtatgcgccggggtttgggggtttaggcggcaaggttgggtatcgggcacaggacattgggggcag**

**IraqBJ**  **gaggatgcggtacagtatgcgccggggtttgggggtttaggcggcaaggttgggtatcgggcacaggacattgggggcag**

**BosniaAJ**  **gaggatgcggtacagtatgcgccggggtttgggggtttaggcggcaaggttgggtatcgggcacaggacattgggggcag**

**CuniculiAJ**  **gaggatgaggtacagtatgcgccggggtttgggggtttaggcggcaaggttgggtatcgagcacaggacattgggggcag**

570 580 590 600 610 620 630 640

....|....|....|....|....|....|....|....|....|....|....|....|....|....|....|....|

**NicholsJ**  **tggggtcagccttgatgtggggtttctctcctttgcctctaacggtgcctgggatagtactgaccccacgcacagtaagt**

**Street14J tggggtcagccttgatgtggggtttctctcctttgcctctaacggtgcctgggatagtactgaccccacgcacagtaagt**

**Bal3J**  **tggggtcagccttgatgtggggtttctctcctttgcctctaacggtgcctgggatagtactgaccccacgcacagtaagt**

**MexicoAJ**  **tggggtcagccttgatgtggggtttctctcctttgcctctaacggtgcctgggatagtactgaccccacgcacagtaagt**

**Sea81-4J**  **tggggtcagccttgatgtggggtttctctcctttgcctctaacggtgcctgggatagtactgaccccacgcacagtaagt**

**GauthierJ**  **tggggtcagccttgatgtggggtttctctcctttgcctctaacggtgcctgggatagtactgaccccacgcacagtaagt**

**CDC2J**  **tggggtcagccttgatgtggggtttctctcctttgcctctaacggtgcctgggatagtactgaccccacgcacagtaagt**

**Fribourg-BlancJ** **tggggtcagccttgatgtggggtttctctcctttgcctctaacggtgcctgggatagtactgaccccacgcacagtaagt**

**IraqBJ**  **tggggtcagccttgatgtggggtttctctcctttgcctctaacggtgcctgggatagtactgaccccacgcacagtaagt**

**BosniaAJ**  **tggggtcagccttgatgtggggtttctctcctttgcctctaacggtgcctgggatagtactgaccccacgcacagtaagt**

**CuniculiAJ**  **tggggtcagccttgatgtggggtttctctcctttgcctctaacggtgcctgggatagtactgacaccacgcacagcaagt**

650 660 670 680 690 700 710 720

....|....|....|....|....|....|....|....|....|....|....|....|....|....|....|....|

**NicholsJ**  **atggcttt-ggggcagacttgaagctaatgtatgcgcgtgcaggacaccctctgtgcacggtagagcttgccagcaatgt**

**Street14J atggcttt-ggggcagacttgaagctaatgtatgcgcgtgcaggacaccctctgtgcacggtagagcttgccagcaatgt**

**Bal3J**  **atggcttt-ggggcagacttgaagctaatgtatgcgcgtgcaggacaccctctgtgcacggtagagcttgccagcaatgt**

**MexicoAJ**  **atggcttt-ggggcagacttgaagctaatgtatgcgcgtgcaggacaccctctgtgcacggtagagcttgccagcaatgt**

**Sea81-4J**  **atggcttt-ggggcagacttgaagctaatgtatgcgcgtgcaggacaccctctgtgcacggtagagcttgccagcaatgt**

**GauthierJ**  **atggcttt-ggggcagacttgaagctaatgtatgcgcgtgcaggacaccctctgtgcacggtagagcttgccagcaatgt**

**CDC2J**  **atggcttt-ggggcagacttgaagctaatgtatgcgcgtgcaggacaccctctgtgcacggtagagcttgccagcaatgt**

**Fribourg-BlancJ** **atggcttt-ggggcagacttgaagctaatgtatgcgcgtgcaggacaccctctgtgcacggtagagcttgccagcaatgt**

**IraqBJ**  **atggcttt-ggggcagacttgaagctaatgtatgcgcgtgcaggacaccctctgtgcacggtagagcttgccagcaatgt**

**BosniaAJ**  **atggcttt-ggggcagacttgaagctaatgtatgcgcgtgcaggacaccctctgtgcacggtagagcttgccagcaatgt**

**CuniculiAJ**  **atggctttgggggcagacttgaagctaatgtatgcgcgtgcaggacaccctctgtgcacggtagagcttgccagcaatgt**

730 740 750 760 770 780 790 800

....|....|....|....|....|....|....|....|....|....|....|....|....|....|....|....|

**NicholsJ**  **tacgctagaagacggatacctcatcggtgcacagaaggacgcaaacaatcagaacaaggataaactgctgtggaatgtag**

**Street14J tacgctagaagacggatacctcatcggtgcacagaaggacgcaaacaatcagaacaaggataaactgctgtggaatgtag**

**Bal3J**  **tacgctagaagacggatacctcatcggtgcacagaaggacgcaaacaatcagaacaaggataaactgctgtggaatgtag**

**MexicoAJ**  **tacgctagaagacggatacctcatcggtgcacagaaggacgcaaacaatcagaacaaggataaactgctgtggaatgtag**

**Sea81-4J**  **tacgctagaagacggatacctcatcggtgcacagaaggacgcaaacaatcagaacaaggataaactgctgtggaatgtag**

**GauthierJ**  **tacgctagaagacggatacctcatcggtgcacagaaggacgcaaacaatcagaacaaggataaactgctgtggaatgtag**

**CDC2J**  **tacgctagaagacggatacctcatcggtgcacagaaggacgcaaacaatcagaacaaggataaactgctgtggaatgtag**

**Fribourg-BlancJ** **tacgctagaagacggatacctcatcggtgcacagaaggacgcaaacaatcagaacaaggataaactgctgtggaatgtag**

**IraqBJ**  **tacgctagaagacggatacctcatcggtgcacagaaggacgcaaacaatcagaacaaggataaactgctgtggaatgtag**

**BosniaAJ**  **tacgctagaagacggatacctcatcggtgcacagaaggacgcaaacaatcagaacaaggataaactgctgtggaatgtag**

**CuniculiAJ**  **tacgctggaagacggatacctcatcggtgcgcagaaggacgcaaacaatcagaacaaggataaactgctgtggaatgtag**

810 820 830 840 850 860 870 880

....|....|....|....|....|....|....|....|....|....|....|....|....|....|....|....|

**NicholsJ**  **ggggccgactcaccctcgaaccaggcgccggcttccgcttctccttcgccctcgacgccggtaaccaacaccagagtgca**

**Street14J ggggccgactcaccctcgaaccaggcgccggcttccgcttctccttcgccctcgacgccggtaaccaacaccagagtgca**

**Bal3J**  **ggggccgactcaccctcgaaccaggcgccggcttccgcttctccttcgccctcgacgccggtaaccaacaccagagtgca**

**MexicoAJ**  **ggggccgactcaccctcgaaccaggcgccggcttccgcttctccttcgccctcgacgccggtaaccaacaccagagtgca**

**Sea81-4J**  **ggggccgactcaccctcgaaccaggcgccggcttccgcttctccttcgccctcgacgccggtaaccaacaccagagtgca**

**GauthierJ**  **ggggccgactcaccctcgaaccaggcgccggcttccgcttctccttcgccctcgacgccggtaaccaacaccagagtgca**

**CDC2J**  **ggggccgactcaccctcgaaccaggcgccggcttccgcttctccttcgccctcgacgccggtaaccaacaccagagtgca**

**Fribourg-BlancJ** **ggggccgactcaccctcgaaccaggcgccggcttccgcttctccttcgccctcgacgccggtaaccaacaccagagtgca**

**IraqBJ**  **ggggccgactcaccctcgaaccaggcgccggcttccgcttctccttcgccctcgacgccggtaaccaacaccagagtgca**

**BosniaAJ**  **ggggccgactcaccctcgaaccaggcgccggcttccgcttctccttcgccctcgacgccggtaaccaacaccagagtgca**

**CuniculiAJ**  **gaggccgactcaccctcgaaccaggcgccggcttccgcttctccttcgccctcgacgccggtaaccaacgccagagtgca**

890 900 910 920 930 940 950 960

....|....|....|....|....|....|....|....|....|....|....|....|....|....|....|....|

**NicholsJ**  **caggactttcaaaatcgcacacagagggcgcagagtgaactcaccgccctctcaaataacctcttccagggagaaagtca**

**Street14J caggactttcaaaatcgcacacagagggcgcagagtgaactcaccgccctctcaaataacctcttccagggagaaagtca**

**Bal3J**  **caggactttcaaaatcgcacacagagggcgcagagtgaactcaccgccctctcaaataacctcttccagggagaaagtca**

**MexicoAJ**  **caggactttcaaaatcgcacacagagggcgcagagtgaactcaccgccctctcaaataacctcttccagggagaaagtca**

**Sea81-4J**  **caggactttcaaaatcgcacacagagggcgcagagtgaactcaccgccctctcaaataacctcttccagggagaaagtca**

**GauthierJ**  **caggactttcaaaatcgcacacagagggcgcagagtgaactcaccgccctctcaaataacctcttccagggagaaagtca**

**CDC2J**  **caggactttcaaaatcgcacacagagggcgcagagtgaactcaccgccctctcaaataacctcttccagggagaaagtca**

**Fribourg-BlancJ** **caggactttcaaaatcgcacacagagggcgcagagtgaactcaccgccctctcaaataacctcttccagggagaaagtca**

**IraqBJ**  **caggactttcaaaatcgcacacagagggcgcagagtgaactcaccgccctctcaaataacctcttccagggagaaagtca**

**BosniaAJ**  **caggactttcaaaatcgcacacagagggcgcagagtgaactcaccgccctctcaaataacctcttccagggagaaagtca**

**CuniculiAJ**  **caagactttcaaaatcgcacacagagggcgcagaatgaactcaccgccctctcaaataacctcttccagggagaaagtca**

970 980 990 1000 1010 1020 1030 1040

....|....|....|....|....|....|....|....|....|....|....|....|....|....|....|....|

**NicholsJ**  **aaaacaggaagcctgggtaacccaggtagtgcaacaggcgacgcagacagtaacggctggagttcgaagcgcgctggaat**

**Street14J aaaacaggaagcctgggtaacccaggtagtgcaacaggcgacgcagacagtaacggctggagttcgaagcgcgctggaat**

**Bal3J**  **aaaacaggaagcctgggtaacccaggtagtgcaacaggcgacgcagacagtaacggctggagttcgaagcgcgctggaat**

**MexicoAJ**  **aaaacaggaagcctgggtaacccaggtagtgcaacaggcgacgcagacagtaacggctggagttcgaagcgcgctggaat**

**Sea81-4J**  **aaaacaggaagcctggctggacgaatatgcaaagaaggtgcttgatgccgtaacggcagccaccgaaaccgcccttcagt**

**GauthierJ**  **aaaacaggaagcctggctggacgaatatgcaaagaaggtgcttgatgccgtaacggcagccaccgaaaccgcccttcagt**

**CDC2J**  **aaaacaggaagcctggctggacgaatatgcaaagaaggtgcttgatgccgtaacggcagccaccgaaaccgcccttcagt**

**Fribourg-BlancJ** **aaaacaggaagcctggctggacgaatatgcaaagaaggtgcttgatgccgtaacggcagccaccgaaaccgcccttcagt**

**IraqBJ**  **aaaacaggaagcctggctggacgaatatgcaaagaaggtgcttgatgccgtaacggcagccaccgaaaccgcccttcagt**

**BosniaAJ**  **aaaacaggaagcctggctggacgaatatgcaaagaaggtgcttgatgccgtaacggcagccaccgaaaccgcccttcagt**

**CuniculiAJ**  **aaaacaggaagcctggctggacgaatacgcaaagaaggtgcttgatgccgtaacggcagccaccgaaaccgccattcagt**

1050 1060 1070 1080 1090 1100 1110 1120

....|....|....|....|....|....|....|....|....|....|....|....|....|....|....|....|

**NicholsJ**  **ctcgggggactacgtacataaacgcgctagaggcagttcagcctaatcctgctaaacctacc--------ggtaaggttg**

**Street14J ctcgggggactacgtacataaacgcgctagaggcagttcagcctaatcctgctaaacctacc--------ggtaaggttg**

**Bal3J**  **ctcgggggactacgtacataaacgcgctagaggcagttcagcctaatcctgctaaacctacc--------ggtaaggttg**

**MexicoAJ**  **ctcgggggactacgtacataaacgcgctagaggcagttcagcctaatcctgctaaacctacc--------ggtaaggttg**

**Sea81-4J**  **cgaggggaaacgcgtacataacggcagtgtcaaacgtaaaagtcacccctccggtagctgccacgcttttgacgaacctg**

**GauthierJ**  **cgaggggaaacgcgtacataacggcagtgtcaaacgtaaaagtcacccctccggtagctgccacgcttttgacgaacctg**

**CDC2J**  **cgaggggaaacgcgtacataacggcagtgtcaaacgtaaaagtcacccctccggtagctgccacgcttttgacgaacctg**

**Fribourg-BlancJ** **cgaggggaaacgcgtacataacggcagtgtcaaacgtaaaagtcacccctccggtagctgccacgcttttgacgaacctg**

**IraqBJ**  **cgaggggaaacgcgtacataacggcagtgtcaaacgtaaaagtcacccctccggtagctgccacgcttttgacgaacctg**

**BosniaAJ**  **cgaggggaaacgcgtacataacggcagtgtcaaacgtaaaagtcacccctccggtagctgccacgcttttgacgaacctg**

**CuniculiAJ**  **cgaggggaaacgcgtacataacggcagtgtcaaacgtaaaagtcacccctccggtagctgccacgcttttggcgaacctg**

1130 1140 1150 1160 1170 1180 1190 1200

....|....|....|....|....|....|....|....|....|....|....|....|....|....|....|....|

**NicholsJ**  **tgcaaaatcttcacaccccgcagggaagtccgccgaacctgccgccgcttcctgcacttcctgcattttccctgatgggg**

**Street14J tgcaaaatcttcacaccccgcagggaagtccgccgaacctgccgccgcttcctgcacttcctgcattttccctgatgggg**

**Bal3J**  **tgcaaaatcttcacaccccgcagggaagtccgccgaacctgccgccgcttcctgcacttcctgcattttccctgatgggg**

**MexicoAJ**  **tgcaaaatcttcacaccccgcagggaagtccgccgaacctgccgccgcttcctgcacttcctgcattttccctgatgggg**

**Sea81-4J**  **---aaggtgttcattacc--------gaccctcctacaccgtcaccgcttcccgcgcttcctgcattttccctgatgggg**

**GauthierJ**  **---aaggtgttcattacc--------gaccctcctacaccgtcaccgcttcccgcgcttcctgcattttccctgatgggg**

**CDC2J**  **---aaggtgttcattacc--------gaccctcctacaccgtcaccgcttcccgcgcttcctgcattttccctgatgggg**

**Fribourg-BlancJ** **---aaggtgttcattacc--------gaccctcctacaccgtcaccgcttcccgcgcttcctgcattttccctgatgggg**

**IraqBJ**  **---aaggtgttcattacc--------gaccctcctacaccgtcaccgcttcccgcgcttcctgcattttccctgatgggg**

**BosniaAJ**  **---aaggtgttcattacc--------gaccctcctacaccgtcaccgcttcccgcgcttcctgcattttccctgatgggg**

**CuniculiAJ**  **---aaggtgttcattacc--------gaccctcctacaccgtcaccgcttcccgcgcttcctgcattttccctgatgggg**

1210 1220 1230 1240 1250 1260 1270 1280

....|....|....|....|....|....|....|....|....|....|....|....|....|....|....|....|

**NicholsJ**  **caggttttgctgcagtacgatgcggagcaggtggtgaaggggtttgagcaggtacagacgcaaatcgtcactgaaattaa**

**Street14J caggttttgctgcagtacgatgcggagcaggtggtgaaggggtttgagcaggtacagacgcaaatcgtcactgaaattaa**

**Bal3J**  **caggttttgttgcagtacgatgcggagcaggtggtgaaggggtttgagcaggtacagacgcaaatcgtcactgaaattaa**

**MexicoAJ**  **caggttttgctgcagtacgatgcggagcaggtggtgaaggggtttgagcaggtacagacgcaaatcgtcactgaaattaa**

**Sea81-4J**  **caggttttgctgcagtacgatgcggagcaggtggtgaaggggtttgagcaggtacagacgcaaatcgttgctgaaattaa**

**GauthierJ**  **caggttttgctgcagtacgatgcggagcaggtggtgaaggggtttgagcaggtacagacgcaaatcgttgctgaaattaa**

**CDC2J**  **caggttttgctgcagtacgatgcggagcaggtggtgaaggggtttgagcaggtacagacgcaaatcgttgctgaaattaa**

**Fribourg-BlancJ** **caggttttgctgcagtacgatgcggagcaggtggtgaaggggtttgagcaggtacagacgcaaatcgttgctgaaattaa**

**IraqBJ**  **caggttttgctgcagtacgatgcggagcaggtggtgaaggggtttgagcaggtacagacgcaaatcgttgctgaaattaa**

**BosniaAJ**  **caggttttgctgcagtacgatgcggagcaggtggtgaaggggtttgagcaggtacagacgcaaatcgttgctgaaattaa**

**CuniculiAJ**  **caggttttgctgcagtatggcgtagagcaggtggtgaaggggtttgagcaggtacagacgcaaatcgtcgctgaaattaa**

1290 1300 1310 1320 1330 1340 1350 1360

....|....|....|....|....|....|....|....|....|....|....|....|....|....|....|....|

**NicholsJ**  **tcagaaagtgcaagcggctgtggcaaaaaataatgcaaacatgcaagcggtcgggggtagtctaggcgatactgcgagaa**

**Street14J tcagaaagtgcaagcggctgtggcaaaaaataatgcaaacatgcaagcggtcgggggtagtctaggcgatactgcgagaa**

**Bal3J**  **tcagaaagtgcaagcggctgtggcaaaaaataatgcaaacatgcaagcggtcgggggtagtctaggcgatactgcgagaa**

**MexicoAJ**  **tcagaaagtgcaagcggctgtggcaaaaaataatgcaaacatgcaagcggtcgggggtagtctaggcgatactgcgagaa**

**Sea81-4J**  **ccagaaagtgcaagcggctgtggctcagagcaaggctgcagcacaggcattcatcaacggtcttaccaaggcaatagaag**

**GauthierJ**  **ccagaaagtgcaagcggctgtggctcagagcaaggctgcagcacaggcattcatcaacggtcttaccaaggcaatagaag**

**CDC2J**  **ccagaaagtgcaagcggctgtggctcagagcaaggctgcagcacaggcattcatcaacggtcttaccaaggcaatagaag**

**Fribourg-BlancJ** **ccagaaagtgcaagcggctgtggctcagagcaaggctgcagcacaggcattcatcaacggtcttaccaaggcaatagaag**

**IraqBJ**  **ccagaaagtgcaagcggctgtggctcagagcaaggctgcagcacaggcattcatcaacggtcttaccaaggcaatagaag**

**BosniaAJ**  **ccagaaagtgcaagcggctgtggctcagagcaaggctgcagcacaggcattcatcaacggtcttaccaaggcaatagaag**

**CuniculiAJ**  **tcagaaagtgcaaatagccgtgggtcggagtagaactgcagcgcagacattctcaaatggcctcgcccaaacggtagcac**

1370 1380 1390 1400 1410 1420 1430 1440

....|....|....|....|....|....|....|....|....|....|....|....|....|....|....|....|

**NicholsJ**  **tggtaggcgaagcgctcattaagcagcaactatcacgtaagcagaacagcattctgaccatggtgagcgtgcaagatgag**

**Street14J tggtaggcgaagcgctcattaagcagcaactatcacgtaagcagaacagcattctgaccatggtgagcgtgcaagatgag**

**Bal3J**  **tggtaggcgaagcgctcattaagcagcaactatcacgtaagcagaacagcattctgaccatggtgagcgtgcaagatgag**

**MexicoAJ**  **tggtaggcgaagcgctcattaagcagcaactatcacgtaagcagaacagcattctgaccatggtgagcgtgcaagatgag**

**Sea81-4J**  **acgtggctgatgcgttgcttgcaccgcataa---gggaaatccgatgagcctcttcaaccttccggatcaacaaaaatta**

**GauthierJ**  **acgtggctgatgcgttgcttgcaccgcataa---gggaaatccgatgagcctcttcaaccttccggatcaacaaaaatta**

**CDC2J**  **acgtggctgatgcgttgcttgcaccgcataa---gggaaatccgatgagcctcttcaaccttccggatcaacaaaaatta**

**Fribourg-BlancJ** **acgtggctgatgcgttgcttgcaccgcataa---gggaaatccgatgagcctcttcaaccttccggatcaacaaaaatta**

**IraqBJ**  **acgtggctgatgcgttgcttgcaccgcataa---gggaaatccgatgagcctcttcaaccttccggatcaacaaaaatta**

**BosniaAJ**  **acgtggctgatgcgttgcttgcaccgcataa---gggaaatccgatgagcctcttcaaccttccggatcaacaaaaatta**

**CuniculiAJ**  **gcatagcagacaagttgcttatgcctcacat---aggaaatctacgcagccttttcaatgatccaaatcaacaggcaacg**

1450 1460 1470 1480 1490 1500 1510 1520

....|....|....|....|....|....|....|....|....|....|....|....|....|....|....|....|

**NicholsJ**  **gtgaaacaggatctggcagatttagtgccgatgatgcgaacggaaataacggcgtttttcgcgagtgtccagcaacacat**

**Street14J gtgaaacaggatctggcagatttagtgccgatgatgcgaacggaaataacggcgtttttcgcgagtgtccagcaacacat**

**Bal3J**  **gtgaaacaggatctggcagatttagtgccgatgatgcgaacggaaataacggcgtttttcgcgagtgtccagcaacacat**

**MexicoAJ**  **gtgaaacaggatctggcagatttagtgccgatgatgcgaacggaaataacggcgtttttcgcgagtgtccagcaacacat**

**Sea81-4J**  **ctgaaggacgatctcgctgatcttattccaaagcttacggctgaggctacaaagtttttcactgagggtcagacgtttgt**

**GauthierJ**  **ctgaaggacgatctcgccgatcttattccaaagcttacggctgaggctacaaagtttttcactgagggtcagacgtttgt**

**CDC2J**  **ctgaaggacgatctcgccgatcttattccaaagcttacggctgaggctacaaagtttttcactgagggtcagacgtttgt**

**Fribourg-BlancJ** **ctgaaggacgatctcgccgatcttattccaaagcttacggctgaggctacaaagtttttcactgagggtcagacgtttgt**

**IraqBJ**  **ctgaaggacgatctcgccgatcttattccaaagcttacggctgaggctacaaagtttttcactgagggtcagacgtttgt**

**BosniaAJ**  **ctgaaggacgatctcgccgatcttattccaaagcttacggctgaggctacaaagtttttcactgagggtcagacgtttgt**

**CuniculiAJ**  **ttgcggcaagatcttacggatcttattccaaagcttacggctgaggctacaaagtttttcaccgaagggcagacgtttgt**

1530 1540 1550 1560 1570 1580 1590 1600

....|....|....|....|....|....|....|....|....|....|....|....|....|....|....|....|

**NicholsJ**  **aaccgaagaagtgaagaagaagacggatgcgttgaatgcggggcagcagatacgtcaggctatacagaacctgcgtgcgt**

**Street14J aaccgaagaagtgaagaagaagacggatgcgttgaatgcggggcagcagatacgtcaggctatacagaacctgcgtgcgt**

**Bal3J**  **aaccgaagaagtgaagaagaagacggatgcgttgaatgcggggcagcagatacgtcaggctatacagaacctgcgtgcgt**

**MexicoAJ**  **aaccgaagaagtgaagaagaagacggatgcgttgaatgcggggcagcagatacgtcaggctatacagaacctgcgtgcgt**

**Sea81-4J**  **aaccgaagaagtgaagaagaagacggatgcgttgaatgcggggcagcagatacgtcaggctatacagaacctgcgtgcgt**

**GauthierJ**  **aaccgaagaagtgaagaagaagacggatgcgttggacgcggggcagcagatacgtcaggctatacagaacctgcgtgcgt**

**CDC2J**  **aaccgaagaagtgaagaagaagacggatgcgttggacgcggggcagcagatacgtcaggctatacagaacctgcgtgcgt**

**Fribourg-BlancJ** **aaccgaagaagtgaagaagaagacggatgcgttggacgcggggcagcagatacgtcaggctatacagaacctgcgtgcgt**

**IraqBJ**  **aaccgaagaagtgaagaagaagacggatgcgttggacgcggggcagcagatacgtcaggctatacagaacctgcgtgcgt**

**BosniaAJ**  **aaccgaagaagtgaagaagaagacggatgcgttggacgcggggcagcagatacgtcaggctatacagaacctgcgtgcgt**

**CuniculiAJ**  **aaccgaagaagtgaagaagaagacggatgcgttggacgcggggaagcagatacgtcaggctatacagaacctgcgtgcgt**

1610 1620 1630 1640 1650 1660 1670 1680

....|....|....|....|....|....|....|....|....|....|....|....|....|....|....|....|

**NicholsJ**  **ctgcatggcgtgcctttctaatgggagtcagcgccgtgtgtctgtatcttgacacctacaatgtcgccttcgatgcgctg**

**Street14J ctgcatggcgtgcctttctaatgggagtcagcgccgtgtgtctgtatcttgacacctacaatgtcgccttcgatgcgctg**

**Bal3J**  **ctgcatggcgtgcctttctaatgggagtcagcgccgtgtgtctgtatcttgacacctacaatgtcgccttcgatgcgctg**

**MexicoAJ**  **ctgcatggcgtgcctttctaatgggagtcagcgccgtgtgtctgtatcttgacacctacaatgtcgccttcgatgcactg**

**Sea81-4J**  **ctgcatggcgtgcctttctaatgggagtcagcgccgtgtgtctgtatcttgacacctacaatgtcgccttcgatgcactg**

**GauthierJ**  **ctgcatggcgtgcctttctaatgggagtcagcgccgtgtgtctgtatcttgacacctacaatgtcgccttcgatgcactg**

**CDC2J**  **ctgcatggcgtgcctttctaatgggagtcagcgccgtgtgtctgtatcttgacacctacaatgtcgccttcgatgcactg**

**Fribourg-BlancJ** **ctgcatggcgtgcctttctaatgggagtcagcgccgtgtgtctgtatcttgacacctacaatgtcgccttcgatgcactg**

**IraqBJ**  **ctgcatggcgtgcctttctaatgggagtcagcgccgtgtgtctgtatcttgacacctacaatgtcgccttcgatgcactg**

**BosniaAJ**  **ctgcatggcgtgcctttctaatgggagtcagcgccgtgtgtctgtatcttgacacctacaatgtcgccttcgatgcactg**

**CuniculiAJ**  **ctgcatggcgtgcctttctaatgggagtcagtgccgtgtgtctgtatcttgacacctacaatgtcgccttcgatgcactg**

1690 1700 1710 1720 1730 1740 1750 1760

....|....|....|....|....|....|....|....|....|....|....|....|....|....|....|....|

**NicholsJ**  **tttacggcgcagtggaagtggctgtcttctggcatatactttgccacagcaccggcaaacgtttttggcaccagggtgtt**

**Street14J tttacggcgcagtggaagtggctgtcttctggcatatactttgccacagcaccggcaaacgtttttggcaccagggtgtt**

**Bal3J**  **tttacggcgcagtggaagtggctgtcttctggcatatacgttgccacagcaccggcaaacgtttttggcaccagggtgtt**

**MexicoAJ**  **cttacagcgcagtggaagtggctgtcttctggcatatacgttgccacagcaccggcaaacgtttttggcaccagggtgtt**

**Sea81-4J**  **cttacagcgcagtggaagtggctgtcttctggcatatacgttgccacagcaccggcaaacgtttttggcaccagggtgtt**

**GauthierJ**  **cttacagcgcagtggaagtggctgtcttctggcatatactttgccacagcaccggcaaacgtttttggcaccagggtgtt**

**CDC2J**  **cttacagcgcagtggaagtggctgtcttctggcatatactttgccacagcaccggcaaacgtttttggcaccagggtgtt**

**Fribourg-BlancJ** **cttacagcgcagtggaagtggctgtcttctggcatatactttgccacagcaccggcaaacgtttttggcaccagggtgtt**

**IraqBJ**  **cttacagcgcagtggaagtggctgtcttctggcatatactttgccacagcaccggcaaacgtttttggcaccagggtgtt**

**BosniaAJ**  **cttacagcgcagtggaagtggctgtcttctggcatatactttgccacagcaccggcaaacgtttttggcaccagggtgtt**

**CuniculiAJ**  **cttacagcgcagtggaagtggctgtcttctggcatatactttgccacagcaccggcaaacgtttttggcaccagggtgtt**

1770 1780 1790 1800 1810 1820 1830 1840

....|....|....|....|....|....|....|....|....|....|....|....|....|....|....|....|

**NicholsJ**  **agataacaccatcgcaagctgtggcgactttgccggattccttaagctcgaaactaagagcggtgacccctacacccacc**

**Street14J agataacaccatcgcaagctgtggcgactttgccggattccttaagctcgaaactaagagcggtgacccctacacccacc**

**Bal3J**  **agataacaccatcgcaagctgtggcgactttgccggattccttaagctcgaaactaagagcggtgacccctacacccacc**

**MexicoAJ**  **agataacaccatcgcaagctgtggcgactttgccggattcctcaaactcgaaactaagagcggtgacccctacacccacc**

**Sea81-4J**  **agataacaccatcgcaagctgtggcgactttgccggattcctcaaactcgaaactaagagcggtgacccctacacccacc**

**GauthierJ**  **agataacaccatcgcaagctgtggcgactttgccggattcctcaaactcgaaactaagagcggtgacccctacacccacc**

**CDC2J**  **agataacaccatcgcaagctgtggcgactttgccggattcctcaaactcgaaactaagagcggtgacccctacacccacc**

**Fribourg-BlancJ** **agataacaccatcgcaagctgtggcgactttgccggattcctcaaactcgaaactaagagcggtgacccctacacccacc**

**IraqBJ**  **agataacaccatcgcaagctgtggcgactttgccggattcctcaaactcgaaactaagagcggtgacccctacacccacc**

**BosniaAJ**  **agataacaccatcgcaagctgtggcgactttgccggattcctcaaactcgaaactaagagcggtgacccctacacccacc**

**CuniculiAJ**  **agacaacaccattgcaagctgtggcgactttgccggattccttaaactcgaaactaaaagcggtgacccctacacccacc**

1850 1860 1870 1880 1890 1900 1910 1920

....|....|....|....|....|....|....|....|....|....|....|....|....|....|....|....|

**NicholsJ**  **tgctcaccggcctggacgccggcgttgaaacacgcgtgtacatccccctcacccatgacctgtacaaaaataataacggg**

**Street14J tgctcaccggcctggacgccggcgttgaaacacgcgtgtacatccccctcacccatgacctgtacaaaaataataacggg**

**Bal3J**  **tgctcaccggcctggacgccggcgttgaaacacgcgtgtacatccccctcacccatgacctgtacaaaaataataacggg**

**MexicoAJ**  **tgctcaccggcctggacgccggcgttgaaacacgcgtgtacatccccctcacccatgacctgtacaaaaataataacggg**

**Sea81-4J**  **tgctcaccggcctggacgccggcgttgaaacacgcgtgtacatccccctcacccatgacctgtacaaaaataataacggg**

**GauthierJ**  **tgctcaccggcctggacgccggcgttgaaacacgcgtgtacatccccctcacccatgacctgtacaaaaataataacggg**

**CDC2J**  **tgctcaccggcctggacgccggcgttgaaacacgcgtgtacatccccctcacccatgacctgtacaaaaataataacggg**

**Fribourg-BlancJ** **tgctcaccggcctggacgccggcgttgaaacacgcgtgtacatccccctcacccatgacctgtacaaaaataataacggg**

**IraqBJ**  **tgctcaccggcctggacgccggcgttgaaacacgcgtgtacatccccctcacccatgacctgtacaaaaataataacggg**

**BosniaAJ**  **tgctcaccggcctggacgccggcgttgaaacacgcgtgtacatccccctcacccatgacctgtacaaaaataataacggg**

**CuniculiAJ**  **tgctcaccggcctggacgccggcgttgaaacacgcgtgtacatccccctcacccatgacccgtacaaaaataataacggg**

1930 1940 1950 1960 1970 1980 1990 2000

....|....|....|....|....|....|....|....|....|....|....|....|....|....|....|....|

**NicholsJ**  **aaccctctcccttccggcggttcctcagggcacattggcctgccggtggtggggaaggcgtggtgtagctatcgcatccc**

**Street14J aaccctctcccttccggcggttcctcagggcacattggcctgccggtggtggggaaggcgtggtgtagctatcgcatccc**

**Bal3J**  **aaccctctcccttccggcggttcctcagggcacattggcctgccggtggtggggaaggcgtggtgtagctatcgcatccc**

**MexicoAJ**  **aaccctctcccttccggcggttcctcagggcacattggcctgccggtggtggggaaggcgtggtgtagctatcgcatccc**

**Sea81-4J**  **aaccctctcccttccggcggttcctcagggcacattggcctgccggtggtggggaaggcgtggtgtagctatcgcatccc**

**GauthierJ**  **aaccctctcccttccggcggttcctcagggcacattggcctgccggtggtggggaaggcgtggtgtagctatcgcatccc**

**CDC2J**  **aaccctctcccttccggcggttcctcagggcacattggcctgccggtggtggggaaggcgtggtgtagctatcgcatccc**

**Fribourg-BlancJ** **aaccctctcccttccggcggttcctcagggcacattggcctgccggtggtggggaaggcgtggtgtagctatcgcatccc**

**IraqBJ**  **aaccctctcccttccggcggttcctcagggcacattggcctgccggtggtggggaaggcgtggtgtagctatcgcatccc**

**BosniaAJ**  **aaccctctcccttccggcggttcctcagggcacattggcctgccggtggtggggaaggcgtggtgtagctatcgcatccc**

**CuniculiAJ**  **aaccctctcccttccggcggctcctcagggcacattagcctgccgatagtgagcagggcgtggtgtagctatcgcatccc**

2010 2020 2030 2040 2050 2060 2070 2080

....|....|....|....|....|....|....|....|....|....|....|....|....|....|....|....|

**NicholsJ**  **ggtgcaggattacggctgggtgaagccaagcgttacggtccatgcctctaccaaccgtgcacacctgaatgcccctgctg**

**Street14J ggtgcaggattacggctgggtgaagccaagcgttacggtccatgcctctaccaaccgtgcacacctgaatgcccctgctg**

**Bal3J**  **ggtgcaggattacggctgggtgaagccaagcgttacggtccatgcctctaccaaccgtgcacacctgaatgcccctgctg**

**MexicoAJ**  **ggtgcaggattacggctgggtgaagccaagcgttacggtccatgcctctaccaaccgtgcacacctgaatgcccctgctg**

**Sea81-4J**  **ggtgcaggattatggctgggtgaagccaagcgttacggtccatgcctctaccaaccgtgcacacctgaatgcccctgctg**

**GauthierJ**  **ggtgcaggattacggctgggtgaagccaagcgttacggtccatgcctctaccaaccgtgcacacctgaatgcccctgctg**

**CDC2J**  **ggtgcaggattacggctgggtgaagccaagcgttacggtccatgcctctaccaaccgtgcacacctgaatgcccctgctg**

**Fribourg-BlancJ** **ggtgcaggattacggctgggtgaagccaagcgttacggtccatgcctctaccaaccgtgcacacctgaatgcccctgctg**

**IraqBJ**  **ggtgcaggattacggctgggtgaagccaagcgttacggtccatgcctctaccaaccgtgcacacctgaatgcccctgctg**

**BosniaAJ**  **ggtgcaggattacggctgggtgaagccaagcgttacggtccatgcctctaccaaccgtgcacacctgaatgcccctgctg**

**CuniculiAJ**  **ggtgcaggattacggctgggtgaagccaagcgttacggtccacgcctctaccaaccgtgcacacctgaatacccctgctg**

2090 2100 2110 2120 2130 2140 2150 2160

....|....|....|....|....|....|....|....|....|....|....|....|....|....|....|....|

**NicholsJ**  **caggtggagcagtaggagctacctatctaaccaaggagtactgtgcacagctgcgtgctggtatttcagccagtctcata**

**Street14J caggtggagcagtaggagctacctatctaaccaaggagtactgtgcacagctgcgtgctggtatttcagccagtctcata**

**Bal3J**  **caggtggagcagtaggagctacctatctaaccaaggagtactgtgcacagctgcgtgctggtatttcagccagtctcata**

**MexicoAJ**  **caggtggagcagtaggagctacctatctaaccaaggagtactgtgcacagctgcgtgctggtatttcagccagtctcata**

**Sea81-4J**  **caggtggagcagtaggagctacctatctaaccaaggagtactgtgcacagctgcgtgctggtatttcagccagtctcata**

**GauthierJ**  **caggtggagcagtaggagctacctatctaactaaggagtactgtgcacagctgcgtgctggtgtttcagccagtctcata**

**CDC2J**  **caggtggagcagtaggagctacctatctaactaaggagtactgtgcacagctgcgtgctggtgtttcagccagtctcata**

**Fribourg-BlancJ** **caggtggagcagtaggagctacctatctaactaaggagtactgtgcacagctgcgtgctggtgtttcagccagtctcata**

**IraqBJ**  **caggtggagcagtaggagctacctatctaactaaggagtactgtgcacagctgcgtgctggtgtttcagccagtctcata**

**BosniaAJ**  **caggtggagcagtaggagctacctatctaactaaggagtactgtgcacagctgcgtgctggtgtttcagccagtctcata**

**CuniculiAJ**  **caggcggagcagtaggagctacctatctaactaaggagtactgtgcacagctgcgtgctggtatttcagccagtctcata**

2170 2180 2190 2200 2210 2220 2230 2240

....|....|....|....|....|....|....|....|....|....|....|....|....|....|....|....|

**NicholsJ**  **gagaagacggtattctcccttgattgggaacagggtatgctctctgatgtcccgtacctgctggtgtccgagtgcctcac**

**Street14J gagaagacggtattctcccttgattgggaacagggtatgctctctgatgtcccgtacctgctggtgtccgagtgcctcac**

**Bal3J**  **gagaagacggtattctcccttgattgggaacagggtatgctctccgatgtcccgtacctgctggtgtccgagtgcctcac**

**MexicoAJ**  **gagaagacggtattctcccttgattgggaacagggtatgctctctgatgtcccgtacctgctggtgtccgagtgcctcac**

**Sea81-4J**  **gagaagacggtattctcccttgattgggaacagggtatgctctctgatgtcccgtacctgctggtgtccgagtgcctcac**

**GauthierJ**  **gagaagacggtattctcccttgattgggaacagggtatgctctctgatgtcccgtacctgctggtgtccgagtgcctcac**

**CDC2J**  **gagaagacggtattctcccttgattgggaacagggtatgctctctgatgtcccgtacctgctggtgtccgagtgcctcac**

**Fribourg-BlancJ** **gagaagacggtattctcccttgattgggaacagggtatgctctctgatgtcccgtacctgctggtgtccgagtgcctcac**

**IraqBJ**  **gagaagacggtattctcccttgattgggaacagggtatgctctctgatgtcccgtacctgctggtgtccgagtgcctcac**

**BosniaAJ**  **gagaagacggtattctcccttgattgggaacagggtatgctctctgatgtcccgtacctgctggtgtccgagtgcctcac**

**CuniculiAJ**  **gagaaggcggtgttctcccttgattgggaacagggtatgctctctgatgtcccgtacctgctggtatccgagtgcctcac**

2250 2260 2270 2280

....|....|....|....|....|....|....|....|....|.

**NicholsJ**  **ccagggaatcggccgcatcgtgtgcggcgtcaccctctcctggtag**

**Street14J ccagggaatcggccgcatcgtgtgcggcgtcaccctctcctggtag**

**Bal3J**  **ccagggaatcggccgcatcgtgtgcggcgtcaccctctcctggtag**

**MexicoAJ**  **ccagggaatcggccgcatcgtgtgcggcgtcaccctctcctggtag**

**Sea81-4J**  **ccagggaatcggccgcatcgtgtgcggcgtcaccctctcctggtag**

**GauthierJ**  **ccagggaatcggccgcatcgtgtgcggcgtcaccctctcctggtag**

**CDC2J**  **ccagggaatcggccgcatcgtgtgcggcgtcaccctctcctggtag**

**Fribourg-BlancJ** **ccagggaatcggccgcatcgtgtgcggcgtcaccctctcctggtag**

**IraqBJ**  **ccagggaatcggccgcatcgtgtgcggcgtcaccctctcctggtag**

**BosniaAJ**  **ccagggaatcggccgcatcgtgtgcggcgtcaccctctcctggtag**

**CuniculiAJ**  **ccagggaatcggccgcatcgtgtgcggcgtcaccctctcctggtag**

- 1. ***tprL* Locus**

The Nichols L sequence shown here is different from the annotation in the genome paper [27], and corresponds to the

newly identified putative start codon, which has typical promoter elements upstream, resulting in an elongated gene and protein.

10 20 30 40 50 60 70 80

....|....|....|....|....|....|....|....|....|....|....|....|....|....|....|....|

**NicholsL**  **MHRAVFFCAGAGALLPGFGALPVFSEQLGIAPQVTGHAQLQWGIKFKKNPSVQPNEYTHGFRTTNDLKISLPLVPKATHL**

**Bal3L MHRAVFFCAGAGALLPGFGALPVFSEQLGIAPQVTGHAQLQWGIKFKKNPSVQPNEYTHGFRTTNDLKISLPLVPKATHL**

**Sea81-4L**  **MHRAVFFCAGAGALLPGFGALPVFSEQLGIAPQVTGHAQLQWGIKFKKNPSVQPNEYTHGFRTTNDLKISLPLVPKATHL**

**Street14L MHRAVFFCAGAGALLPGFGALPVFSEQVGIAPQVTGHAQLQWGIKFKKNPSSQPNQYTHGFRTTNDLKISLPLVPKATHL**

**MexicoAL**  **MHRAVFFCAGAGALLPGFGALPVFSEQLGIAPQVTGHAQLQWGIKFKKNPSSQPNQYTHGFRTTNDLKISLPLVPKATHL**

**CuniculiAL**  **MHRAVFFCAGAGALLPGFGALPVFSEQLGIAPQVTGHAQLQWGIKFKKNPSVQPNEYTHGFRTTNDLKISLPLVPKATHL**

**BosniaAL MHRAVFFCAGAGALLPGFGALPVFSEQLGIAPQVTGHAQLQWGIKFKKNPSVQPNEYTHGFRTTNDLKISLPLVPKATHL**

**IraqBL MHRAVFFCAGAGALLPGFGALPVFSEQLGIAPQVTGHAQLQWGIKFKKNPSVQPNEYTHGFRTTNDLKISLPLVPKATHL**

**GauthierL**  **----------------------------------------------MRCHENGHVRCAAARGARGDTKLPLRTENCKYSA**

**SamoaDL**  **----------------------------------------------MRCHENGHVRCAAARGARGDTKLPLRTENCKYSA**

**CDC2L**  **----------------------------------------------MRCHENGHVRCAAARGARGDTKLPLRTENCKYSA**

**Fribourg-BlancL** **----------------------------------------------MRCHENGHVRCAAARGARGDTKLPLRTENCKYSA**

90 100 110 120 130 140 150 160

....|....|....|....|....|....|....|....|....|....|....|....|....|....|....|....|

**NicholsL**  **RRGGARSGVWAELRLKDLTVDFESPRPGQAFTLKKPKASFEATLHCYNAYLTIGKDPNCFINFAQLWDPFVTSDYKQEDV**

**Bal3L RRGGARSGVWAELRLKDLTVDFESPRPGQAFTLKKPKASFEATLHCYNAYLTIGKDPNCFINFAQLWDPFVTSDYKQEDV**

**Sea81-4L**  **RRGGARSGVWAELRLKDLTVDFESPRPGQAFTLKKPKASFEATLHCYNAYLTIGKDPNCFINFAQLWDPFVTSDYKQEDV**

**Street14L RRGGARSGVWAELRLKELTVDFESPKPGQAFTLKKPKASFEATLHCYNAYLTIGKDPNCFINFAQLWDPFVTSDYKQEDV**

**MexicoAL**  **RRGGARSGVWAELRLKELTVDFESPKPGQAFTLKKPKASFEATLHCYNAYLTIGKDPNCFINFAQLWDPFVTSDYKQEDV**

**CuniculiAL**  **RRGGARSGVWAELRLKDLTVDFESPRPGQAFTLKKPKASFEATLHCYNAYLTIGKDPNCFINFAQLWDPFVTSDYKQEDV**

**BosniaAL RRGGARSGVWAELRLKDLTVDFESPRPGQAFTLKKPKASFEATLHCYNAYLTIGKDPNCFINFAQLWDPFVTSDYKQEDV**

**IraqBL RRGGARSGVWAELRLKDLTVDFESPRPGQAFTLKKPKASFEATLHCYNAYLTIGKDPNCFINFAQLWDPFVTSDYKQEDV**

**GauthierL**  **PPPPCLPEEGAELRLKDLTVDFESPKPGQAFTLKKPKASFEATLHCYNAYLTIGKDPNCFINFAQLWDPFVTSDYKQEDV**

**SamoaDL**  **PPPPCLPEEGAELRLKDLTVDFESPKPGQAFTLKKPKASFEATLHCYNAYLTIGKDPNCFINFAQLWDPFVTSDYKQEDV**

**CDC2L**  **PPPPCLPEEGAELRLKDLTVDFESPKPGQAFTLKKPKASFEATLHCYNAYLTIGKDPNCFINFAQLWDPFVTSDYKQEDV**

**Fribourg-BlancL** **PPPPCLPEEGAELRLKDLTVDFESPKPGQAFTLKKPKASFEATLHCYNAYLTIGKDPNCFINFAQLWDPFVTSDYKQEDV**

170 180 190 200 210 220 230 240

....|....|....|....|....|....|....|....|....|....|....|....|....|....|....|....|

**NicholsL**  **RYAPGFGGYGGKLGYRAQDIGGSGIGLDVGLLSFASNGMWDSGTAHSKYGFGADATLTYTHHRAERIKMELAGNATLEPQ**

**Bal3L RYAPGFGGYGGKLGYRAQDIGGSGIGLDVGLLSFASNGMWDSGTAHSKYGFGADATLTYTHHRAERIKMELAGNATLEPQ**

**Sea81-4L**  **RYAPGFGGYGGKLGYRAQDIGGSGIGLDVGLLSFASNGMWDSGTAHSKYGFGADATLTYTHHRAERIKMELAGNATLEPQ**

**Street14L RYAPGFGGYGGKLGYRAQDIGGSGIGLDVGLLSFASNGMWDSGTAHSKYGFGADATLTYTHHRAERIKMELAGNATLEPQ**

**MexicoAL**  **RYAPGFGGYGGKLGYRAQDIGGSGIGLDVGLLSFASNGMWDSGTAHSKYGFGADATLTYTHHRAERIKMELAGNATLEPQ**

**CuniculiAL**  **RYAPGFGGYGGKLGYRAQDIGGSGIGLDVGLLSFASNGMWDSGTAHSKYGFGADATLTYTHHRAERIKMELAGNATLEPQ**

**BosniaAL RYAPGFGGYGGKLGYRAQDIGGSGIGLDVGLLSFASNGMWDSGTAHSKYGFGADATLTYTHHRAERIKMELAGNATLEPQ**

**IraqBL RYAPGFGGYGGKLGYRAQDIGGSGIGLDVGLLSFASNGMWDSGTAHSKYGFGADATLTYTHHRAERIKMELAGNATLEPQ**

**GauthierL**  **RYAPGFGGYGGKLGYRAQDIGGSGIGLDVGLLSFASNGMWDSGTAHSKYGFGADATLTYTHHRAERIKMELAGNATLEPQ**

**SamoaDL**  **RYAPGFGGYGGKLGYRAQDIGGSGIGLDVGLLSFASNGMWDSGTAHSKYGFGADATLTYTHHRAERIKMELAGNATLEPQ**

**CDC2L**  **RYAPGFGGYGGKLGYRAQDIGGSGIGLDVGLLSFASNGMWDSGTAHSKYGFGADATLTYTHHRAERIKMELAGNATLEPQ**

**Fribourg-BlancL** **RYAPGFGGYGGKLGYRAQDIGGSGIGLDVGLLSFASNGMWDSGTAHSKYGFGADATLTYTHHRAERIKMELAGNATLEPQ**

250 260 270 280 290 300 310 320

....|....|....|....|....|....|....|....|....|....|....|....|....|....|....|....|

**NicholsL**  **YTTGTEQGKNNEQKNRLLWSAGGRLTLTPGYGFRLVLALDVGNIHRSDADIGKTVNVQAKAAEAVSAAVTEFWAQVAQIM**

**Bal3L YTTGTEQGKNNEQKNRLLWSAGGRLTLTPGYGFRLVLALDVGNIHRSDADIGKTVNVQAKAAEAVSAAVTEFWAQVAQIM**

**Sea81-4L**  **YTTGTEQGKNNEQKNRLLWSAGGRLTLTPGYGFRLVLALDVGNIHRSDADIGKTVNVQAKAAEAVSAAVTEFWAQVAQIM**

**Street14L YTTGTEQGKNNEQKNRLLWSAGGRLTLTPGYGFRLVLALDVGNIHRSDADIGKTVNVQAKAAEAVSAAVTEFWAQVAQIM**

**MexicoAL**  **YTTGTEQGKNNEQKNRLLWSAGGRLTLTPGYGFRLVLALDVGNIHRSDADIGKTVNVQAKAAEAVSAAVTEFWAQVAQIM**

**CuniculiAL**  **YTTGTEQGKNNEQKNRLLWSAGGRLTLTPGYGFRLVLALDVGNIHRSDADIGKTVNVQAKAAEAVSAAVTEFWAQVAQIM**

**BosniaAL YTTGTEQGKNNEQKNRLLWSAGGRLTLTPGYGFRLVLALDVGNIHRSDADIGKTVNVQAKAAEAVSAAVTEFWAQVAQIM**

**IraqBL YTTGTEQGKNNEQKNRLLWSAGGRLTLTPGYGFRLVLALDVGNIHRSDADIGKTVNVQAKAAEAVSAAVTEFWAQVAQIM**

**GauthierL**  **YTTGTEQGKNNEQKNRLLWSAGGRLTLTPGYGFRLVLALDVGNIHRSDADIGKTVNVQAKAAEAVSAAVTEFWAQVAQIM**

**SamoaDL**  **YTTGTEQGKNNEQKNRLLWSAGGRLTLTPGYGFRLVLALDVGNIHRSDADIGKTVNVQAKAAEAVSAAVTEFWAQVAQIM**

**CDC2L**  **YTTGTEQGKNNEQKNRLLWSAGGRLTLTPGYGFRLVLALDVGNIHRSDADIGKTVNVQAKAAEAVSAAVTEFWAQVAQIM**

**Fribourg-BlancL** **YTTGTEQGKNNEQKNRLLWSAGGRLTLTPGYGFRLVLALDVGNIHRSDADIGKTVNVQAKAAEAVSAAVTEFWAQVAQIM**

330 340 350 360 370 380 390 400

....|....|....|....|....|....|....|....|....|....|....|....|....|....|....|....|

**NicholsL**  **ANGGVGEFFVKKVRGAALIAQVALVVSHLEGKLSNLLQSTLGLGAVVNQLTQGFAELLKKPDPAIALVTFFAWLHRLHVH**

**Bal3L ANGGVGEFFVKKVRGAALIAQVALVVSHLEGKLSNLLQSTLGLGAVVNQLTQGFAELLKKPDPAIALVTFFAWLHRLHVH**

**Sea81-4L**  **ANGGVGEFFVKKVRGAALIAQVALVVSHLEGKLSNLLQSTLGLGAVVNQLTQGFAELLKKPDPAIALVTFFAWLHRLHVH**

**Street14L ANGGVGEFFVKKVRGATLIQQVALVASYLEGKLPNLLTGAQRLGAVVDQVIQRFAELLAKPDPAIALVTFFAWLHRLHVH**

**MexicoAL**  **ANGGVGEFFVKKVRGATLIQQVALVASYLEGKLPNLLTGAQRLGAVVDQVIQRFAELLAKPDPAIALVTFFAWLHRLHVH**

**CuniculiAL**  **ANGGVGEFFVKKVRGAALIAQVALVVSHLEGKLSNLLQSTLGLGAVVNQLTQGFAELLKKPDPAIALVTFFAWLHRLHVH**

**BosniaAL ANGGVGEFFVKKVRGAALIAQVALVVSHLEGKLSNLLQSTLGLGAVVNQLTQGFAELLKKPDPAIALVTFFAWLHRLHVH**

**IraqBL ANGGVGEFFVKKVRGAALIAQVALVVSHLEGKLSNLLQSTLGLGAVVNQLTQGFAELLKKPDPAIALVTFFAWLHRLHVH**

**GauthierL**  **ANGGVGEFFVKKVRGAALIAQVALVVSHLEGKLSNLLQSTLGLGAVVNQLTQGFAELLKKPDPAIALVTFFAWLHRLHVH**

**SamoaDL**  **ANGGVGEFFVKKVRGAALIAQVALVVSHLEGKLSNLLQSTLGLGAVVNQLTQGFAELLKKPDPAIALVTFFAWLHRLHVH**

**CDC2L**  **ANGGVGEFFVKKVRGAALIAQVALVVSHLEGKLSNLLQSTLGLGAVVNQLTQGFAELLKKPDPAIALVTFFAWLHRLHVH**

**Fribourg-BlancL** **ANGGVGEFFVKKVRGAALIAQVALVVSHLEGKLSNLLQSTLGLGAVVNQLTQGFAELLKKPDPAIALVTFFAWLHRLHVH**

410 420 430 440 450 460 470 480

....|....|....|....|....|....|....|....|....|....|....|....|....|....|....|....|

**NicholsL**  **ELGADALLSMQWKWLSSGAYFATAGANMFGKRVFSRQLTDYLDCAAFLKLETKSGDPYTHLLTGLNAGVEARLYIPFTYT**

**Bal3L ELGADALLSMQWKWLSSGAYFATAGANMFGKRVFSRQLTDYLDCAAFLKLETKSGDPYTHLLTGLNAGVEARLYIPFTYT**

**Sea81-4L**  **ELGADALLSMQWKWLSSGAYFATAGANMFGKRVFSRQLTDYLDCAAFLKLETKSGDPYTHLLTGLNAGVEARLYIPFTYT**

**Street14L ELGADALLSMQWKWLSSGAYFATAGANMFGRRVFSRQCTDYLDCAAFLKLETKSGDPYTHLLTGLNAGVEARLYIPFTYT**

**MexicoAL**  **ELGADALLSMQWKWLSSGAYFATAGANMFGRRVFSRQCTDYLDCAAFLKLETKSGDPYTHLLTGLNAGVEARLYIPFTYT**

**CuniculiAL**  **ELGADALLSMQWKWLSSGAYFATAGANMFGKRVFSRQLTDYLDCAAFLKLETKSGDPYTHLLTGLNAGVEARLYIPFTYT**

**BosniaAL ELGADALLSMQWKWLSSGAYFATAGANMFGRRVFSRQCTDYLDCAAFLKLETKSGDPYTHLLTGLNAGVEARLYIPFTYT**

**IraqBL ELGADALLSMQWKWLSSGAYFATAGANMFGRRVFSRQCTDYLDCAAFLKLETKSGDPYTHLLTGLNAGVEARLYIPFTYT**

**GauthierL**  **ELGADALLSMQWKWLSSGAYFATAGANMFGRRVFSRQCTDYLDCAAFLKLETKSGDPYTHLLTGLNAGVEARLYIPFTYT**

**SamoaDL**  **ELGADALLSMQWKWLSSGAYFATAGANMFGRRVFSRQCTDYLDCAAFLKLETKSGDPYTHLLTGLNAGVEARLYIPFTYT**

**CDC2L**  **ELGADALLSMQWKWLSSGAYFATAGANMFGRRVFSRQCTDYLDCAAFLKLETKSGDPYTHLLTGLNAGVEARLYIPFTYT**

**Fribourg-BlancL** **ELGADALLSMQWKWLSSGAYFATAGANMFGRRVFSRQCTDYLDCAAFLKLETKSGDPYTHLLTGLNAGVEARLYIPFTYT**

490 500 510 520 530 540 550 560

....|....|....|....|....|....|....|....|....|....|....|....|....|....|....|....|

**NicholsL**  **SYVNNGGIDYKKTTMRGPINLPVVGKTWLSYQIALGSHAWLKPYAVVYGTTNRFNTDKANNLLREKAMQYHVGFTVSPIE**

**Bal3L SYVNNGGIDYKKTTMRGPINLPVVGKTWLSYQIALGSHAWLKPYAVVYGTTNRFNTDKANNLLREKAMQYHVGFTVSPIE**

**Sea81-4L**  **SYVNNGGIDYKKTTMRGPINLPVVGKTWLSYQIALGSHAWLKPYAVVYGTTNRFNTDKANNLLREKAMQYHVGFTVSPIE**

**Street14L SYVSNGGIDYKGKTMRDPINLPVVGKTWLSYQIALGSHAWLKPYAVVYGTTNRFNTDKANNLLREKAMQYHVGFTVSPIE**

**MexicoAL**  **SYVSNGGIDYKGKTMRDPINLPVVGKTWLSYQIALGSHAWLKPYAVVYGTTNRFNTDKANNLLREKAMQYHVGFTVSPIE**

**CuniculiAL**  **SYVNNGGIDYKKTTMRGPINLPVVGKTWLSYQIALGSHAWLKPYAVVYGTTNRFNTDKANNLLREKAMQYHVGFTVSPIE**

**BosniaAL SYVNNGGIDYNRTTMRGPINLPVVGKTWLSYQIALGSHAWLKPYAVVYGTTNRFNTDKANNLLREKAMQYHVGFTVSPIE**

**IraqBL SYVNNGGIDYNRTTMRGPINLPVVGKTWLSYQIALGSHAWLKPYAVVYGTTNRFNTDKANNLLREKAMQYHVGFTVSPIE**

**GauthierL**  **SYVNNGGIDYNRTTMRGPINLPVVGKTWLSYQIALGSHAWLKPYAVVYGTTNRFNTDKANNLLREKAMQYHVGFTVSPIE**

**SamoaDL**  **SYVNNGGIDYNRTTMRGPINLPVVGKTWLSYQIALGSHAWLKPYAVVYGTTNRFNTDKANNLLREKAMQYHVGFTVSPIE**

**CDC2L**  **SYVNNGGIDYNRTTMRGPINLPVVGKTWLSYQIALGSHAWLKPYAVVYGTTNRFNTDKANNLLREKAMQYHVGFTVSPIE**

**Fribourg-BlancL** **SYVNNGGIDYNRTTMRGPINLPVVGKTWLSYQIALGSHAWLKPYAVVYGTTNRFNTDKANNLLREKAMQYHVGFTVSPIE**

570 580 590 600

....|....|....|....|....|....|....|....|..

**NicholsL**  **KVEFDARWEQGRLATAPYMLITEDISSDKHFGTFVCGLKIAW**

**Bal3L KVEFDARWEQGRLATAPYMLITEDISSDKHFGTFVCGLKIAW**

**Sea81-4L**  **KVEFDARWEQGRLATAPYMLITEDISSDKHFGTFVCGLKIAW**

**Street14L KVEFDARWEQGRLATAPYMLITEDISSDKHFGTFVCGLKIAW**

**MexicoAL**  **KVEFDARWEQGRLATAPYMLITEDISSDKHFGTFVCGLKIAW**

**CuniculiAL**  **KVEFDARWEQGRLATAPYMLITEDISSDKHFGTFVCGLKIAW**

**BosniaAL KVEFDARWEQGRLATAPYMLITEDISSDKHFGTFVCGLKIAW**

**IraqBL KVEFDARWEQGRLATAPYMLITEDISSDKHFGTFVCGLKIAW**

**GauthierL**  **KVEFDARWEQGRLATAPYMLITEDISSDKHFGTFVCGLKIAW**

**SamoaDL**  **KVEFDARWEQGRLATAPYMLITEDISSDKHFGTFVCGLKIAW**

**CDC2L**  **KVEFDARWEQGRLATAPYMLITEDISSDKHFGTFVCGLKIAW**

**Fribourg-BlancL** **KVEFDARWEQGRLATAPYMLITEDISSDKHFGTFVCGLKIAW**

10 20 30 40 50 60 70 80

....|....|....|....|....|....|....|....|....|....|....|....|....|....|....|....|

**NicholsL**  **ctgcaccgtgcagtctttttttgtgcgggcgccggggcgctgctcccggggtttggcgcactgcccgttttttctgagca**

**Bal3L ctgcaccgtgcagtctttttttgtgcgggcgccggggcgctgctcccggggtttggcgcactgcccgttttttctgagca**

**Sea81-4L**  **ctgcaccgtgcagtctttttttgtgcgggcgccggggcgctgctcccggggtttggcgcactgcccgttttttctgagca**

**MexicoAL**  **ctgcaccgtgcagtctttttttgtgcgggggccggggcgctgctcccggggtttggcgcactgcccgttttttctgagca**

**Street14L ctgcaccgtgcagtctttttttgtgcgggggccggggcgctgctcccggggtttggcgcactgcccgttttttctgagca**

**CuniculiAL**  **ctgcaccgtgcagtctttttttgtgcgggcgccggggcgctgctcccggggtttggcgcactgcccgttttttctgagca**

**BosniaAL ctgcaccgtgcagtctttttttgtgcgggcgccggggcgctgctcccggggtttggcgcactgcccgttttttctgagca**

**IraqBL ctgcaccgtgcagtctttttttgtgcgggcgccggggcgctgctcccggggtttggcgcactgcccgttttttctgagca**

**GauthierL**  **--------------------------------------------------------------------------------**

**SamoaDL**  **--------------------------------------------------------------------------------**

**CDC2L**  **--------------------------------------------------------------------------------**

**Fribourg-BlancL** **--------------------------------------------------------------------------------**

90 100 110 120 130 140 150 160

....|....|....|....|....|....|....|....|....|....|....|....|....|....|....|....|

**NicholsL**  **gctaggcattgccccgcaggtaactggtcacgcccagctgcagtggggcattaagtttaaaaagaacccttcggtgcaac**

**Bal3L gctaggcattgccccgcaggtaactggtcacgcccagctgcagtggggcattaagtttaaaaagaacccttcggtgcaac**

**Sea81-4L**  **gctaggcattgccccgcaggtaactggtcacgcccagctgcagtggggcattaagtttaaaaagaacccttcggtgcaac**

**MexicoAL**  **gctaggcattgccccgcaggtaactggtcacgcccagctgcagtggggcattaagtttaaaaagaacccttcgtcgcagc**

**Street14L ggtaggcattgccccgcaggtaactggtcacgcccagctgcagtggggcattaagtttaaaaagaacccttcgtcgcagc**

**CuniculiAL**  **gctaggcattgccccgcaggtaactggtcacgcccagctgcagtggggcattaagtttaaaaagaacccttcggtgcaac**

**BosniaAL gctaggcattgccccgcaggtaactggtcacgcccagctgcagtggggcattaagtttaaaaagaacccttcggtgcaac**

**IraqBL gctaggcattgccccgcaggtaactggtcacgcccagctgcagtggggcattaagtttaaaaagaacccttcggtgcaac**

**GauthierL**  **----------------------------------------------------------atgcggtgccatgaaaacgggc**

**SamoaDL**  **----------------------------------------------------------atgcggtgccatgaaaacgggc**

**CDC2L**  **----------------------------------------------------------atgcggtgccatgaaaacgggc**

**Fribourg-BlancL** **----------------------------------------------------------atgcggtgccatgaaaacgggc**

170 180 190 200 210 220 230 240

....|....|....|....|....|....|....|....|....|....|....|....|....|....|....|....|

**NicholsL**  **cgaacgagtacacgcacgggtttcgcaccaccaatgatttgaaaatctcgctccccctcgtgcccaaggccacgcacctg**

**Bal3L cgaacgagtacacgcacgggtttcgcaccaccaatgatttgaaaatctcgctccccctcgtgcccaaggccacgcacctg**

**Sea81-4L**  **cgaacgagtacacgcacgggtttcgcaccaccaatgatttgaaaatctcgctccccctcgtgcccaaggccacgcacctg**

**MexicoAL**  **cgaaccagtacacgcacgggtttcgcaccaccaatgatttgaaaatctcgcttcccctcgtgcccaaggccacgcacctg**

**Street14L cgaaccagtacacgcacgggtttcgcaccaccaatgatttgaaaatctcgcttcccctcgtgcccaaggccacgcacctg**

**CuniculiAL**  **cgaacgagtacacgcacgggtttcgcaccaccaatgatttgaaaatctcgctccccctcgtgcccaaggccacgcacctg**

**BosniaAL cgaacgagtacacgcacgggtttcgcaccaccaatgatttgaaaatctcgctccccctcgtgcccaaggccacgcacctg**

**IraqBL cgaacgagtacacgcacgggtttcgcaccaccaatgatttgaaaatctcgctccccctcgtgcccaaggccacgcacctg**

**GauthierL**  **acgtgcgctgcgctgcagcgcgcggcgcacggggcgatacaaaactccctctgcgcactgagaattgcaagtatagcgcg**

**SamoaDL**  **acgtgcgctgcgctgcagcgcgcggcgcacggggcgatacaaaactccctctgcgcactgagaattgcaagtatagcgcg**

**CDC2L**  **acgtgcgctgcgctgcagcgcgcggcgcacggggcgatacaaaactccctctgcgcactgagaattgcaagtatagcgcg**

**Fribourg-BlancL** **acgtgcgctgcgctgcagcgcgcggcgcacggggcgatacaaaactccctctgcgcactgagaattgcaagtatagcgcg**

250 260 270 280 290 300 310 320

....|....|....|....|....|....|....|....|....|....|....|....|....|....|....|....|

**NicholsL**  **cgtcgcggcggggcgcgctcaggcgtgtgggcggagctgcggctcaaggacctgaccgttgattttgaatcccccaggcc**

**Bal3L cgtcgcggcggggcgcgctcaggcgtgtgggcggagctgcggctcaaggacctgaccgttgattttgaatcccccaggcc**

**Sea81-4L**  **cgtcgcggcggggcgcgctcaggcgtgtgggcggagctgcggctcaaggacctgaccgttgattttgaatcccccaggcc**

**MexicoAL**  **cgtcgcggcggggcgcgctcaggcgtgtgggcggagctgcggctcaaggagctgaccgttgattttgaatcccccaagcc**

**Street14L cgtcgcggcggggcgcgctcaggcgtgtgggcggagctgcggctcaaggagctgaccgttgattttgaatcccccaagcc**

**CuniculiAL**  **cgtcgcggcggggcgcgctcaggcgtgtgggcggagctgcggctcaaggacctgaccgttgattttgaatcccccaggcc**

**BosniaAL cgtcgcggcggggcgcgctcaggcgtgtgggcggagctgcggctcaaggacctgaccgttgattttgaatcccccaggcc**

**IraqBL cgtcgcggcggggcgcgctcaggcgtgtgggcggagctgcggctcaaggacctgaccgttgattttgaatcccccaggcc**

**GauthierL**  **cccccacccccctgtctacctgaggagggggcggagctgcggctcaaggacctgaccgttgattttgaatcccccaagcc**

**SamoaDL**  **cccccacccccctgtctacctgaggagggggcggagctgcggctcaaggacctgaccgttgattttgaatcccccaagcc**

**CDC2L**  **cccccacccccctgtctacctgaggagggggcggagctgcggctcaaggacctgaccgttgattttgaatcccccaagcc**

**Fribourg-BlancL** **cccccacccccctgtctacctgaggagggggcggagctgcggctcaaggacctgaccgttgattttgaatcccccaagcc**

330 340 350 360 370 380 390 400

....|....|....|....|....|....|....|....|....|....|....|....|....|....|....|....|

**NicholsL**  **ggggcaggcctttacgctcaaaaaacccaaagcgtcttttgaagcaacgctccactgctacaacgcgtacctgaccattg**

**Bal3L ggggcaggcctttacgctcaaaaaacccaaagcgtcttttgaagcaacgctccactgctacaacgcgtacctgaccattg**

**Sea81-4L**  **ggggcaggcctttacgctcaaaaaacccaaagcgtcttttgaagcaacgctccactgctacaacgcgtacctgaccattg**

**MexicoAL**  **ggggcaggcctttacgctcaaaaaacccaaagcgtcttttgaagcaacgctccactgctacaacgcgtacctgaccattg**

**Street14L ggggcaggcctttacgctcaaaaaacccaaagcgtcttttgaagcaacgctccactgctacaacgcgtacctgaccattg**

**CuniculiAL**  **ggggcaggcctttacgctcaaaaaacccaaagcgtcttttgaagcaacgctccactgctacaacgcgtacctgaccattg**

**BosniaAL ggggcaggcctttacgctcaaaaaacccaaagcgtcttttgaagcaacgctccactgctacaacgcgtacctgaccattg**

**IraqBL ggggcaggcctttacgctcaaaaaacccaaagcgtcttttgaagcaacgctccactgctacaacgcgtacctgaccattg**

**GauthierL**  **ggggcaggcctttacgctcaaaaaacccaaagcgtcttttgaagcaacgctccactgctacaacgcgtacctgaccattg**

**SamoaDL**  **ggggcaggcctttacgctcaaaaaacccaaagcgtcttttgaagcaacgctccactgctacaacgcgtacctgaccattg**

**CDC2L**  **ggggcaggcctttacgctcaaaaaacccaaagcgtcttttgaagcaacgctccactgctacaacgcgtacctgaccattg**

**Fribourg-BlancL** **ggggcaggcctttacgctcaaaaaacccaaagcgtcttttgaagcaacgctccactgctacaacgcgtacctgaccattg**

410 420 430 440 450 460 470 480

....|....|....|....|....|....|....|....|....|....|....|....|....|....|....|....|

**NicholsL**  **gcaaggacccgaactgctttattaactttgcgcagctgtgggacccgttcgtgaccagcgactacaagcaggaggacgtg**

**Bal3L gcaaggacccgaactgctttattaactttgcgcagctgtgggacccgttcgtgaccagcgactacaagcaggaggacgtg**

**Sea81-4L**  **gcaaggacccgaactgctttattaactttgcgcagctgtgggacccgttcgtgaccagcgactacaagcaggaggacgtg**

**MexicoAL**  **gcaaggacccgaactgctttattaactttgcgcagctgtgggacccgttcgtgaccagcgactacaagcaggaggacgtg**

**Street14L gcaaggacccgaactgctttattaactttgcgcagctgtgggacccgttcgtgaccagcgactacaagcaggaggacgtg**

**CuniculiAL**  **gcaaggacccgaactgctttattaactttgcgcagctgtgggacccgttcgtgaccagcgactacaagcaggaggacgtg**

**BosniaAL gcaaggacccgaactgctttattaactttgcgcagctgtgggacccgttcgtgaccagcgactacaagcaggaggacgtg**

**IraqBL gcaaggacccgaactgctttattaactttgcgcagctgtgggacccgttcgtgaccagcgactacaagcaggaggacgtg**

**GauthierL**  **gcaaggacccgaactgctttattaactttgcgcagctgtgggacccgttcgtgaccagcgactacaagcaggaggacgtg**

**SamoaDL**  **gcaaggacccgaactgctttattaactttgcgcagctgtgggacccgttcgtgaccagcgactacaagcaggaggacgtg**

**CDC2L**  **gcaaggacccgaactgctttattaactttgcgcagctgtgggacccgttcgtgaccagcgactacaagcaggaggacgtg**

**Fribourg-BlancL** **gcaaggacccgaactgctttattaactttgcgcagctgtgggacccgttcgtgaccagcgactacaagcaggaggacgtg**

490 500 510 520 530 540 550 560

....|....|....|....|....|....|....|....|....|....|....|....|....|....|....|....|

**NicholsL**  **cgctatgcgcccggctttggcggctatgggggcaagctcggctaccgtgcgcaggatatcggcggcagtggcattgggtt**

**Bal3L cgctatgcgcccggctttggcggctatgggggcaagctcggctaccgtgcgcaggatatcggcggcagtggcattgggtt**

**Sea81-4L**  **cgctatgcgcccggctttggcggctatgggggcaagctcggctaccgtgcgcaggatatcggcggcagtggcattgggtt**

**MexicoAL**  **cgctatgcgcccggctttggcggctatgggggcaagctcggctaccgtgcgcaggatatcggcggcagtggcattgggtt**

**Street14L cgctatgcgcccggctttggcggctatgggggcaagctcggctaccgtgcgcaggatatcggcggcagtggcattgggtt**

**CuniculiAL**  **cgctatgcgcccggctttggcggctatgggggcaagctcggctaccgtgcgcaggatatcggcggcagtggcattgggtt**

**BosniaAL cgctatgcgcccggctttggcggctatgggggcaagctcggctaccgtgcgcaggatatcggcggcagtggcattgggtt**

**IraqBL cgctatgcgcccggctttggcggctatgggggcaagctcggctaccgtgcgcaggatatcggcggcagtggcattgggtt**

**GauthierL**  **cgctatgcgcccggctttggcggctatgggggcaagctcggctaccgtgcgcaggatatcggcggcagtggcattgggtt**

**SamoaDL**  **cgctatgcgcccggctttggcggctacgggggcaagctcggctaccgtgcgcaggatatcggcggcagtggcattgggtt**

**CDC2L**  **cgctatgcgcccggctttggcggctacgggggcaagctcggctaccgtgcgcaggatatcggcggcagtggcattgggtt**

**Fribourg-BlancL** **cgctatgcgcccggctttggcggctatgggggcaagctcggctaccgtgcgcaggatatcggcggcagtggcattgggtt**

570 580 590 600 610 620 630 640

....|....|....|....|....|....|....|....|....|....|....|....|....|....|....|....|

**NicholsL**  **ggacgtgggcttgctctcgtttgcgtcaaacggtatgtgggatagtggtactgcgcacagcaagtacggctttggagcag**

**Bal3L ggacgtgggcttgctctcgtttgcgtcaaacggtatgtgggatagtggtactgcgcacagcaagtacggctttggagcag**

**Sea81-4L**  **ggacgtgggcttgctctcgtttgcgtcaaacggtatgtgggatagtggtactgcgcacagcaagtacggctttggagcag**

**MexicoAL**  **ggacgtgggcttgctctcgtttgcgtcaaacggtatgtgggatagtggtactgcgcacagcaagtacggctttggagcag**

**Street14L ggacgtgggcttgctctcgtttgcgtcaaacggtatgtgggatagtggtactgcgcacagcaagtacggctttggagcag**
[truncated: 19,794 more chars]
