# Supplementary material for: Fine Analysis of Genetic Diversity of the tpr Gene Family among Treponemal Species, Subspecies and Strains
Source: PLoS Negl Trop Dis. 2013 May 16;7(5):e2222. doi: 10.1371/journal.pntd.0002222 (PMC3656149; doi:10.1371/journal.pntd.0002222)
Supplement: Figure S2 — Alignment of amino acid sequences of the predicted protein sequences encoded at the tprD (2.1), tprC (2.2), and tprF and tprI (2.3) loci. The TprD2 truncated proteins encoded by the tprC/D loci in T. paraluiscuniculi, as well as by the tprF locus in T. p. pallidum strains are not included in the alignment for clarity purposes. Also, no T. paraluiscuniculi tprF and tprI ORFs are included because the tprF or tprI coding sequences are absent in the rabbit pathogen. DVR: Discrete variable regions. EL: External loops predicted by 3D models. SP: Predicted signal peptide. The last letter on the left column (strain name) indicates the locus where the predicted protein sequence is encoded. Red font, T. p. pallidum subspecies; blue T. p. pertenue; brilliant green, T. p. endemicum; and yellow, the Simian treponeme. (DOCX) [file pntd.0002222.s002.docx]

**Supplemental Figure 2**

**2.1**

**EL1 EL2**

**SP DVR1**

10 20 30 40 50 60 70 80 90 100

....|....|....|....|....|....|....|....|....|....|....|....|....|....|....|....|....|....|....|....|

**NicholsD**  **MGRQVMQAGVLAGMVCAASGYAGVLTPQVSGTAQLQWGIAFQKNPRTGPGKHTHGFRTTNSLTISLPLVSKHTHTRRGEARSGVWAQLQLKDLAVELASS**

**Street14D MGRQVMQAGVLAGMVCAASGYAGVLTPQVSGTAQLQWGIAFQKNPRTGPGKHTHGFRTTNSLTISLPLVSKHTHTRRGEARSGVWAQLQLKDLAVELASS**

**GauthierD MGKQVMQAGVLAGMVCAASGYAGVLTPQVSGTAQLQWGIAFQKNPHTVPGKHTHGFRTTNSLTISLPLVSKHTHTRRGEARSGVWAQLQLKDLAVELASS**

**CDC2D MGKQVMQAGVLAGMVCAASGYAGVLTPQVSGTAQLQWGIAFQKNPHTVPGEHTHGFRTTNSLTISLPLVSKHTHTRRGEARSGVWAQLQLKDLAVELASS**

**Bal-3D MGRQVMQAGVLAGMVCAASGYAGVLTPQVSGTAQLQWGIAFQKNPRTGPGKHTHGFRTTNSLTISLPLVSKHTHTRRGEARSGVWAQLQLKDLAVELASS**

**MexicoD MGRQVMQAGVLAGMVCAASGYAGVLTPQVSGTAQLQWGIAFQKNPRTGPGKHTHGFRTTNSLTISLPLVSKHTHTRRGEARSGVWAQLQLKDLAVELASS**

**Sea81-4D MGRQVMQAGVLAGMVCAASGYAGVLTPQVSGTAQLQWGIAFQKNPRTGPGKHTHGFRTTNSLTISLPLVSKHTHTRRGEARSGVWAQLQLKDLAVELASS**

**SamoaDD MGRQVMQAGVLAGMVCAASGYAGVLTPQVSGTAQLQWGIAFQKNPRTGPGKHTHGFRTTNSLTISLPLVSKHTHTRRGEARSGVWAQLQLKDLAVELASS**

**IraqBD MGRQVMQAGVLAGMVCAASGYAGVLTPQVSGTAQLQWGIAFQKNPRTGPGKHTHGFRTTNSLTISLPLVSKHTHTRRGEARSGVWAQLQLKDLAVELASS**

**BosniaAD MGKQVMQAGVLAGMVCAASGYAGVLTPQVSGTAQLQWGIAFQKNPHTVPGKHTHGFRTTNSLTISLPLVSKHTHTRRGEARSGVWAQLQLKDLAVELASS**

**Fribourg-BlancD MGRQVMQAGVRAGVVCAASGYAGVLTPQVSGTAQLQWGIAFQKNPHTDPGKHTHGFRTTNSLTISLPLVSKHTHTRRGEARSGVWAQLQLKDLAVELASS**

**EL3 EL4**

110 120 130 140 150 160 170 180 190 200

....|....|....|....|....|....|....|....|....|....|....|....|....|....|....|....|....|....|....|....|

**NicholsD**  **KSSTALSFTKPTASFQATLHCYGAYLTVGTSPSCVVNFAQLWKPFVTRAYSEKDTRYAPGFSGSGAKLGYQAHNVGNSGVDVDIGFLSFLSNGAWDSTDT**

**Street14D**  **KSSTALSFTKPTASFQATLHCYGAYLTVGTSPSCVVNFAQLWKPFVTRAYSEKDTRYAPGFSGSGAKLGYQAHNVGNSGVDVDIGFLSFLSNGAWDSTDT**

**GauthierD**  **KSSTALSFTKPTASFQATLHCYGAYLTVGTSPSCVVNFAQLWKPFVTRAYSEKDTRYAPGFSGSGAKLGYQAHNVGNSGVDVDIGFLSFLSNGAWDSTDT**

**CDC2D**  **KSSTALSFTKPTASFQATLHCYGAYLTVGTSPSCVVNFAQLWKPFVTRAYSEKDTRYAPGFSGSGAKLGYQAHNVGNSGVDVDIGFLSFLSNGAWDSTDT**

**Bal-3D**  **KSSTALSFTKPTASFQATLHCYGAYLTVGTSPSCVVNFAQLWKPFVTRAYSEKDTRYAPGFSGSGAKLGYQAHNVGNSGVDVDIGFLSFLSNGAWDSTDT**

**MexicoD**  **KSSTALSFTKPTASFQATLHCYGAYLTVGTSPSCVVNFAQLWKPFVTRAYSEKDTRYAPGFSGSGAKLGYQAHNVGNSGVDVDIGFLSFLSNGAWDSTDT**

**Sea81-4D**  **KSSTALSFTKPTASFQATLHCYGAYLTVGTSPSCVVNFAQLWKPFVTRAYSEKDTRYAPGFSGSGAKLGYQAHNVGNSGVDVDIGFLSFLSNGAWDSTDT**

**SamoaDD**  **KSSTALSFTKPTASFQATLHCYGAYLTVGTSPSCVVNFAQLWKPFVTRAYSEKDTRYAPGFSGSGAKLGYQAHNVGNSGVDVDIGFLSFLSNGAWDSTDT**

**IraqBD KSSTALSFTKPTASFQATLHCYGAYLTVGTSPSCVVNFAQLWKPFVTRAYSEKDTRYAPGFSGSGAKLGYQAHNVGNSGVDVDIGFLSFLSNGAWDSTDT**

**BosniaAD**  **KSSTALSFTKPTASFQATLHCYGAYLTVGTSPSCVVNFAQLWKPFVTRAYSEKDTRYAPGFSGSGAKLGYQAHNVGNSGVDVDIGFLSFLSNGAWDSTDT**

**Fribourg-BlancD** **KSSTALSFTKPTASFQATLHCYGAYLTVGTSPSCVVNFAQLWEPFVTRAYSEKNTRYAPGFSGSGAKLGYQAHNVGNSGVDVDIGFLSFLSNGAWDSTDT**

**EL5 EL6**

**DVR2 DVR3**

210 220 230 240 250 260 270 280 290 300

....|....|....|....|....|....|....|....|....|....|....|....|....|....|....|....|....|....|....|....|

**NicholsD**  **THSKYGFGADATLSYGVDRQRLLTLELAGNATLDQNYVKGTEDSKNENKTALLWGVGGRLTLEPGAGFRFSFALDAGNQHQSNAHAQTQERAILKAREVF**

**Street14D**  **THSKYGFGADATLSYGVDRQRLLTLELAGNATLDQNYVKGTEDSKNENKTALLWGVGGRLTLEPGAGFRFSFALDAGNQHQSNAHAQTQERAILKAREVF**

**GauthierD**  **THSKYGFGADATLSYGVDRQRLLTLELAGNATLEQHYRKGTEDSTNENKTALLWGVGGRLTLEPGAGFRFSFALDAGNQHQSNADAQTQKERVSLAGEVF**

**CDC2D**  **THSKYGFGADATLSYGVDRQRLLTLELAGNATLEQHYRKGTEDSTNENKTALLWGVGGRLTLEPGAGFRFSFALDAGNQHQSNADAQTQKERVSLAGEVF**

**Bal-3D**  **THSKYGFGADATLSYGVDRQRLLTLELAGNATLDQNYVKGTEDSKNENKTALLWGVGGRLTLEPGAGFRFSFALDAGNQHQSNAQFYARMAPSQRVHEVI**

**MexicoD**  **THSKYGFGADATLSYGVDRQRLLTLELAGNATLDQNYVKGTEDSKNENKTALLWGVGGRLTLEPGAGFRFSFALDAGNQHQSNAQFYARMAPSQRVHEVI**

**Sea81-4D**  **THSKYGFGADATLSYGVDRQRLLTLELAGNATLDQNYVKGTEDSKNENKTALLWGVGGRLTLEPGAGFRFSFALDAGNQHQSNAQFYARMAPSQRVHEVI**

**SamoaDD**  **THSKYGFGADATLSYGVDRQRLLTLELAGNATLDQNYVKGTEDSKNENKTALLWGVGGRLTLEPGAGFRFSFALDAGNQHQSNAQFYARMAPSQRVHEVI**

**IraqBD THSKYGFGADATLSYGVDRQRLLTLELAGNATLEQNYLKGTEDPKNENKTALLWGVGGRLTLEPGAGFRFSFALDAGNQHQSDTKFYFRMAPSQRVHEVI**

**BosniaAD**  **THSKYGFGADATLSYGVDRQRLLTLELAGNATLEQNYLKGTEDPKNENKTALLWGVGGRLTLEPGAGFRFSFALDAGNQHQSDTKFYFRMAPSQRVHEVI**

**Fribourg-BlancD** **THSKYGFGADATLXYGVDRQRLLTLELAGNATLEQNYLKGTEDPKNENKTALLWGVGGRLTLEPGAGFRFSFALDAGNQHQSDAQFYARMAPSQRVHEVI**

**EL7**

**DVR4**

310 320 330 340 350 360 370 380 390 400

....|....|....|....|....|....|....|....|....|....|....|....|....|....|....|....|....|....|....|....|

**NicholsD**  **RRVEGKLVQNLPNIMMPPGITE--QTTLIEMVGLAALIAEGTLGSAIQTVLAAGALAALVSQLVPNIEQGVRDVFRSSDPRVVTAKLLAFLERAPMNALN**

**Street14D**  **RRVEGKLVQNLPNIMMPPGITE--QTTLIEMVGLAALIAEGTLGSAIQTVLAAGALAALVSQLVPNIEQGVRDVFRSSDPRVVTAKLLAFLERAPMNALN**

**GauthierD**  **GQVVGKLVQNLPNIMMPLGITE--QTTLIEMVGLAALIAEGTLGSAIQTVLAAGALAALVSQLVPHIEQGVRDVFRSSDPRVVTAKLLAFLERAPMNALN**

**CDC2D**  **GQVVGKLVQNLPNIMMPLGITE--QTTLIEMVGLAALIAEGTLGSAIQTVLAAGALAALVSQLVPHIEQGVRDVFRSSDPRVVTAKLLAFLERAPMNALN**

**Bal-3D**  **TSLGDTLLTSPQQDVVSFFVQELSKGSLLEKAGLVTLLAQRT----IVGLASSGGYLRHLNGKGLEINMRLIEQQKNPDARMRTALFISWLQFTYTKTLN**

**MexicoD**  **TSLGDTLLTSPQQDVVSFFVQELSKGSLLEKAGLVTLLAQRT----IVGLASSGGYLRHLNGKGLEINMRLIEQQKNPDARMRTALFISWLQFTYTKTLN**

**Sea81-4D**  **TSLGDTLLTSPQQDVVSFFVQELSKGSLLEKAGLVTLLAQRT----IVGLASSGGYLRHLNGKGLEINMRLIEQQKNPDARMRTALFISWLQFTYTKTLN**

**SamoaDD**  **TSLGDTLLTSPQQDVVSFFVQELSKGSLLEKAGLVTLLAQRT----IVGLASSGGYLRHLNGKGLEINMRLIEQQKNPDARMRTALFISWLQFTYTKTLN**

**IraqBD TSLGDTLLTSPQQDVVSFFVQELSKGSLLEKAGLVTLLAQRT----IVGLASSGGYLRHLNGKGLEINMRLIEQQKNPDARMRTALFISWLQFTYTKTLN**

**BosniaAD**  **NSLGDTLLTSPQQDVVSFFVQELSKGSLLEKAGLVTLLAQRT----IVGLASSGGYLRHLNGKGLEINMRLIEQQKNPDARMRTALFISWLQFTYTKTLN**

**Fribourg-BlancD** **NSLGDTLLTSPKQDVVSFFVQELSKGSLLEKAGLVTLLAQRT----IVSLASTGGYLRHLNGKGLEINMRLIEQQKNPDARMRTALFISWLQFTYTKTLN**

**EL8 EL9**

**DVR5**

410 420 430 440 450 460 470 480 490 500

....|....|....|....|....|....|....|....|....|....|....|....|....|....|....|....|....|....|....|....|

**NicholsD**  **IDALLRMQWKWLSSGIYFATAGTNIFGKRVFATTRAHYFDFAGFLKLETKSGDPYTHLLTGLNAGVEARVYIPLTYIRYRNNGGYELNGAVPPGTINMPI**

**Street14D**  **IDALLRMQWKWLSSGIYFATAGTNIFGKRVFATTRAHYFDFAGFLKLETKSGDPYTHLLTGLNAGVEARVYIPLTYVFYRNNGGYELNRVVPPGTINMPI**

**GauthierD**  **IDALLRMQWKWLSSGIYFATAGTNIFGKRVFATTRAHYFDFAGFLKLETKSGDPYTHLLTGLNAGVEARVYIPLTYVFYKNNGGYPLNGVVPSGTINMPI**

**CDC2D**  **IDALLRMQWKWLSSGIYFATAGTNIFGKRVFATTRAHYFDFAGFLKLETKSGDPYTHLLTGLNAGVEARVYIPLTYVFYKNNGGHPLNGVVPSGTINMPI**

**Bal-3D**  **IDALLRMQWRWLSSGIYFATAGTNIFGERVFFKNQADHFDFAGFLKLETKSGDPYTHLLTGLNAGVEARVYIPLTYIFYINNGGAQYKGSNSDGVINTPI**

**MexicoD**  **IDALLRMQWRWLSSGIYFATAGTNIFGERVFFKNQADHFDFAGFLKLETKSGDPYTHLLTGLNAGVEARVYIPLTYIFYINNGGAQYKGSNSDGVINTPI**

**Sea81-4D**  **IDALLRMQWRWLSSGIYFATAGTNIFGERVFFKNQADHFDFAGFLKLETKSGDPYTHLLTGLNAGVEARVYIPLTYIFYINNGGAQYKGSNSDGVINTPI**

**SamoaDD**  **IDALLRMQWRWLSSGIYFATAGTNIFGERVFFKNQADHFDFAGFLKLETKSGDPYTHLLTGLNAGVEARVYIPLTYIFYINNGGAQYKGSNSDGVINTPI**

**IraqBD IDALLRMQWKWLSSGIYFATAGTNIFGERVFFKNQADHFDFAGFLKLETKSGDPYTHLLTGLNAGVEARVYIPLTYIFYINNGGAQYKGSNSDGVINTPI**

**BosniaAD**  **IDALLRMQWKWLSSGIYFATAGTNIFGERVFFKNQADHFDFAGFLKLETKSGDPYTHLLTGLNAGVEARVYIPLTYIFYINNGGAQYKGSNSDGVINTPI**

**Fribourg-BlancD** **IDALLGMQWRWLSSGIYFATAGTNIFGERVFFKNQADHFDFAGFLKLETKSGDPYTHLLTGLNAGVEARVYIPLTYIFYKNNGGAQYKGSNSDGVINTPI**

**EL10 EL11**

**DVR6 DVR7**

510 520 530 540 550 560 570 580 590 **600**

....|....|....|....|....|....|....|....|....|....|....|....|....|....|....|....|....|....|....|....**|**

**NicholsD**  **LGKAWCSYRIPLGSHAWLAPHTSVLGTTNRFNIINPAGNLLNERALQYQVGLTFSPFEKVELSAQWEQGVLADAPYMGIAESIWSERHFGTLVCGMKVTW**

**Street14D**  **LGKAWCSYRIPLGSHAWLAPHTSVLGTTNRFNIINPAGNLLNERALQYQVGLTFSPFEKVELSAQWEQGVLADAPYMGIAESIWSERHFGTLVCGMKVTW**

**GauthierD**  **LGKAWCSYRIPLGSHAWLAPHTSVLGTTNRFNIINAAGNLVNERALQYQVGLTFSPFEKVELSAQWEQGVLSDVPYMGITQSIWSERHFGTFVCGMKVTW**

**CDC2D**  **LGKAWCSYRIPLGSHAWLAPHTSVLGTTNRFNIINAAGNLVNERALQYQVGLTFSPFEKVELSAQWEQGVLSDVPYMGITQSIWSERHFGTLVCGMKVTW**

**Bal-3D**  **LSKAWCSYRIPLGSHAWLAPHTSVLWATNRFNHNQSGDALLREHALQYQVGLTFSPFEKVELSAQWEQGVLADAPYMGIAESIWSERHFGTLVCGMKVTW**

**MexicoD**  **LSKAWCSYRIPLGSHAWLAPHTSVLWATNRFNHNQSGDALLREHALQYQVGLTFSPFEKVELSAQWEQGVLADAPYMGIAESIWSERHFGTLVCGMKVTW**

**Sea81-4D**  **LSKAWCSYRIPLGSHAWLAPHTSVLWATNRFNHNQSGDALLREHALQYQVGLTFSPFEKVELSAQWEQGVLADAPYMGIAESIWSERHFGTLVCGMKVTW**

**SamoaDD**  **LSKAWCSYRIPLGSHAWLAPHTSVLWATNRFNHNQSGDALLREHALQYQVGLTFSPFEKVELSAQWEQGVLADAPYMGIAESIWSERHFGTLVCGMKVTW**

**IraqBD LSKAWCSYRIPLGSHAWLAPHTSVLWATNRFNHNQSGDALLREHALQYQVGLTFSPFEKVELSAQWEQGVLSDVPYMGIAESIWSERHFGTLVCGMKVTW**

**BosniaAD**  **LSKAWCSYRIPLGSHAWLAPHTSVLWATNRFNHNQSGDALLREHALQYQVGLTFSPFEKVELSAQWEQGVLSDVPYMGIAESIWSERHFGTLVCGMKVTW**

**Fribourg-BlancD** **LSKAWCSYRIPLGSHAWLAPHTSVLWATNRFNHNQSGDALLREHALQYQVGLTFSPFEKVELSAQWEQGVLADAPYMGITESIWSERHFGTFVCGMKVTW**

**2.2**

**EL1 EL2**

**SP DVR1**

10 20 30 40 50 60 70 80 90 100

....|....|....|....|....|....|....|....|....|....|....|....|....|....|....|....|....|....|....|....|

**NicholsC**  **MGRQVMQAGVLAGMVCAASGYAGVLTPQVSGTAQLQWGIAFQKNPRTGPGKHTHGFRTTNSLTISLPLVSKHTHTRRGEARSGVWAQLQLKDLAVELASS**

**Street14C**  **MGRQVMQAGVLAGMVCAASGYAGVLTPQVSGTAQLQWGIAFQKNPRTGPGKHTHGFRTTNSLTISLPLVSKHTHTRRGEARSGVWAQLQLKDLAVELASS**

**Bal3C**  **MGRQVMQAGVLAGMVCAASGYAGVLTPQVSGTAQLQWGIAFQKNPRTGPGKHTHGFRTTNSLTISLPLVSKHTHTRRGEARSGVWAQLQLKDLAVELASS**

**Sea81-4C**  **MGRQVMQAGVLAGMVCAASGYAGVLTPQVSGTAQLQWGIAFQKNPRTGPGKHTHGFRTTNSLTISLPLVSKHTHTRRGEARSGVWAQLQLKDLAVELASS**

**MexicoAC**  **MGRQVMQAGVLAGMVCAASGYAGVLTPQVSGTAQLQWGIAFQKNPRTGPGKHTHGFRTTNSLTISLPLVSKHTHTRRGEARSGVWAQLQLKDLAVELASS**

**GauthierC**  **MGKQVMQAGVLAGMVCAASGYAGVLTPQVSGTAQLQWGIAFQKNPHTVPGEHTHGFRTTNSLTISLPLVSKHTHTRRGEARSGVWAQLQLKDLAVELASS**

**CDC2C**  **MGKQVMQAGVLAGMVCAASGYAGVLTPQVSGTAQLQWGIAFQKNPHTVPGEHTHGFRTTNSLTISLPLVSKHTHTRRGEARSGVWAQLQLKDLAVELASS**

**SamoaDC**  **MGKQVMQAGVLAGMVCAASGYAGVLTPQVSGTAQLQWGIAFQKNPHTVPGKHTHGFRTTNSLTISLPLVSKHTHTRRGEARSGVWAQLQLKDLAVELASS**

**IraqBC**  **MGKQVMQAGVLAGMVCAASGYAGVLTPQVSGTAQLQWGIAFQKNPHTVPGKHTHGFRTTNSLTISLPLVSKHTHTRRGEARSGVWAQLQLKDLAVELASS**

**BosniaAC**  **MGKQVMQAGVLAGMVCAASGYAGVLTPQVSGAAQLQWGIAFQKNPHTVPGKHTHGFRTTNSLTISLPLVSKHTHTRRGEARSGVWAQLQLKDLAVELASS**

**Fribourg-BlancC** **MGKQVMQAGVLAGMVCAASGYAGVLTPQVSGTAQLQWGIAFQKNPHTVPGKHTHGFRTTNSLTISLPLVSKHTHTRRGEARSGVWAQLQLKDLAVELASS**

**EL3 EL4**

110 120 130 140 150 160 170 180 190 200

....|....|....|....|....|....|....|....|....|....|....|....|....|....|....|....|....|....|....|....|

**NicholsC**  **KSSTALSFTKPTASFQATLHCYGAYLTVGTSPSCVVNFAQLWKPFVTRAYSEKDTRYAPGFSGSGAKLGYQAHNVGNSGVDVDIGFLSFLSNGAWDSTDT**

**Street14C**  **KSSTALSFTKPTASFQATLHCYGAYLTVGTSPSCVVNFAQLWKPFVTRAYSEKDTRYAPGFSGSGAKLGYQAHNVGNSGVDVDIGFLSFLSNGAWDSTDT**

**Bal3C**  **KSSTALSFTKPTASFQATLHCYGAYLTVGTSPSCVVNFAQLWKPFVTRAYSEKDTRYAPGFSGSGAKLGYQAHNVGNSGVDVDIGFLSFLSNGAWDSTDT**

**Sea81-4C**  **KSSTALSFTKPTASFQATLHCYGAYLTVGTSPSCVVNFAQLWKPFVTRAYSEKDTRYAPGFSGSGAKLGYQAHNVGNSGVDVDIGFLSFLSNGAWDSTDT**

**MexicoAC**  **KSSTALSFTKPTASFQATLHCYGAYLTVGTSPSCVVNFAQLWKPFVTRAYSEKDTRYAPGFSGSGAKLGYQAHNVGNSGVDVDIGFLSFLSNGAWDSTDT**

**GauthierC**  **KSSTALSFTKPTASFQATLHCYGAYLTVGTSPSCVVNFAQLWKPFVTRAYSEKDTRYAPGFSGSGAKLGYQAHNVGNSGVDVDIGFLSFLSNGAWDSTDT**

**CDC2C**  **KSSTALSFTKPTASFQATLHCYGAYLTVGTSPSCVVNFAQLWKPFVTRAYSEKDTRYAPGFSGSGAKLGYQAHNVGNSGVDVDIGFLSFLSNGAWDSTDT**

**SamoaDC**  **KSSTALSFTKPTASFQATLHCYGAYLTVGTSPSCVVNFAQLWKPFVTRAYSEKDTRYAPGFSGSGAKLGYQAHNVGNSGVDVDIGFLSFLSNGAWDSTDT**

**IraqBC**  **KSSTALSFTKPTASFQATLHCYGAYLTVGTSPSCVVNFAQLWKPFVTRAYSEKDTRYAPGFSGSGAKLGYQAHNVGNSGVDVDIGFLSFLSNGAWDSTDT**

**BosniaAC**  **KSSTALSFTKPTASFQATLHCYGAYLTVGTSPSCVVNFAQLWKPFVTRAYSEKDTRYAPGFSGSGAKLGYQAHNVGNSGVDVDIGFLSFLSNGAWDSTDT**

**Fribourg-BlancC** **KSSTALSFTKPTASFQATLHCYGAYLTVGTSPSCVVNFAQLWKPFVTRAYSEKDTRYAPGFSGSGAKLGYQAHNVGNSGVDVDIGFLSFLSNGAWDSTDA**

**EL5 EL6**

**DVR2 DVR3**

210 220 230 240 250 260 270 280 290 300

....|....|....|....|....|....|....|....|....|....|....|....|....|....|....|....|....|....|....|....|

**NicholsC**  **THSKYGFGADATLSYGVDRQRLLTLELAGNATLDQNYVKGTEDSKNENKTALLWGVGGRLTLEPGAGFRFSFALDAGNQHQSNAHAQTQERAILKAREVF**

**Street14C**  **THSKYGFGADATLSYGVDRQRLLTLELAGNATLDQNYVKGTEDSKNENKTALLWGVGSRLTLEPGAGFRFSFALDAGNQHQSNAHAQTQERAILKAREVF**

**Bal3C**  **THSKYGFGADATLSYGVDRQRLLTLELAGNATLDQNYVKGTEDSKNENKTALLWGVGGRLTLEPGAGFRFSFALDAGNQHQSNAHAQTQERAILKAREVF**

**Sea81-4C**  **THSKYGFGADATLSYGVDRQRLLTLELAGNATLDQNYVKGTEDSKNENKTALLWGVGGRLTLEPGAGFRFSFALDAGNQHQSNAHAQTQERAILKAREVF**

**MexicoAC**  **THSKYGFGADATLSYGVDRQRLLTLELAGNATLEQHYRKGTEDSTNENKTALLWGVGGRLTLEPGAGFRFSFALDAGNQHQSNAHAQTQERAILKAREVF**

**GauthierC**  **THSKYGFGADATLSYGVDRQRLLTLELAGNATLEQHYRKGTEDSTNENKTALLWGVGGRLTLEPGAGFRFSFALDAGNQHQSNADAQTQKERVSLAGEVF**

**CDC2C**  **THSKYGFGADATLSYGVDRQRLLTLELAGNATLEQHYRKGTEDSTNENKTALLWGVGGRLTLEPGAGFRFSFALDAGNQHQSNADAQTQKERVSLAGEVF**

**SamoaDC**  **THSKYGFGADATLSYGVDRQRLLTLELAGNATLEQHYRKGTEDSTNENKTALLWGVGGRLTLEPGAGFRFSFALDAGNQHQSNAHAQTQKERVSLAGEVF**

**IraqBC**  **THSKYGFGADATLSYGVDRQRLLTLELAGNATLEQNYLKGTEDPKNENKTALLWGVGGRLTLEPGAGFRFSFALDAGNQHQSNADAQTQEERVSLAGEVF**

**BosniaAC**  **THSKYGFGADATLSYGVDRQRLLTLELAGNATLEQNYLKGTEDPKNENKTALLWGVGGRLTLEPGAGFRFSFALDAGNQHQSNADAQTQKERVSLAGEVF**

**Fribourg-BlancC** **THSKYGFGADATLSYGVDRQRLLTLELAGNATLEQHYRKGTEDSTNENKTALLWGVGGRLTLEPGAGFRFSFALDAGNQHQSDTDAQTQKERVSLAGEVF**

**EL7**

**DVR4**

310 320 330 340 350 360 370 380 390 400

....|....|....|....|....|....|....|....|....|....|....|....|....|....|....|....|....|....|....|....|

**NicholsC**  **RRVEGKLVQNLPNIMMPPGITEQTTLIEMVGLAALIAEGTLGSAIQTVLAAGALAALVSQLVPNIEQGVRDVFRSSDPRVVTAKLLAFLERAPMNALNID**

**Street14C**  **RRVEGKLVQNLPNIMMPPGITEQTTLIEMVGLAALIAEGTLGSAIQTVLAAGALAALVSQLVPNIEQGVRDVFRSSDPRVVTAKLLAFLERAPMNALNID**

**Bal3C**  **RRVEGKLVQNLPNIMMPPGITEQTTLIEMVGLAALIAEGTLGSAIQTVLAAGALAALVSQLVPNIEQGVRDVFRSSDPRVVTAKLLAFLERAPMNALNID**

**Sea81-4C**  **RRVEGKLVQNLPNIMMPPGITEQTTLIEMVGLAALIAEGTLGSAIQTVLAAGALAALVSQLVPNIEQGVRDVFRSSDPRVVTAKLLAFLERAPMNALNID**

**MexicoAC**  **RRVEGKLVQNLPNIMMPPGITEQTTLIEMVGLAALIAEGTLGSAIQTVLAAGALAALVSQLVPNIEQGVRDVFRSSDPRVVTAKLLAFLERAPMNALNID**

**GauthierC**  **GQVVGKLVQNLPNIMMPLGITEQTTLIEMVGLAALIAEGTLGSAIQTVLAAGALAALVSQLVPHIEQGVRDVFRSSDPRVVTAKLLAFLERAPMNALNID**

**CDC2C**  **GQVVGKLVQNLPNIMMPLGITEQTTLIEMVGLAALIAEGTLGSAIQTVLAAGALAALVSQLVPHIEQGVRDVFRSSDPRVVTAKLLAFLERAPMNALNID**

**SamoaDC**  **GQVVGKLVQNLPNIMMPLGITEQTTLIEMVGLAALIAEGTLGSAIQTVLAAGALAALVSQLVPHIEQGVRDVFRSSDPRVVTAKLLAFLERAPMNALNID**

**IraqBC**  **GQVVGKLVQNLPNIMMPLGITEQTTLIEMVGLAALIAEGTLGSAIQTVLAAGALAALVSQLVPHIEQGVRDVFRSSDPRVVTAKLLAFLERAPMNALNID**

**BosniaAC**  **GQVVGKLVQNLPNIMMPLGITEQTTLIEMVGLAALIAEGTLGSAIQTVLAAGALAALVSQLVPHIEQGVRDVFRSSDPRVVTAKLLAFLERAPMNALNID**

**Fribourg-BlancC** **GRVVEKLVQNLLNIMMPLGITEQTTLIEMVGLAALIAEGTLGSAIQTVPAAGVPAALVSQLVPHIEQGVRDVFRSSDPRVVTAKLLAFLERAPMNALNID**

**EL8 EL9**

**DVR5**

410 420 430 440 450 460 470 480 490 500

....|....|....|....|....|....|....|....|....|....|....|....|....|....|....|....|....|....|....|....|

**NicholsC**  **ALLRMQWKWLSSGIYFATAGTNIFGKRVFATTRAHYFDFAGFLKLETKSGDPYTHLLTGLNAGVEARVYIPLTYIRYRNNGGYELNGAVPPGTINMPILG**

**Street14C**  **ALLRMQWKWLSSGIYFATAGTNIFGKRVFATTRAHYFDFAGFLKLETKSGDPYTHLLTGLNAGVEARVYIPLTYVFYRNNGGYELNGAVPPGTINMPILG**

**Bal3C**  **ALLRMQWKWLSSGIYFATAGTNIFGKRVFATTRAHYFDFAGFLKLETKSGDPYTHLLTGLNAGVEARVYIPLTYVFYRNNGGYELNRVVPSGIINMPILG**

**Sea81-4C**  **ALLRMQWKWLSSGIYFATAGTNIFGKRVFATTRAHYFDFAGFLKLETKSGDPYTHLLTGLNAGVEARVYIPLTYVFYRNNGGYELNRVVPSGIINMPILG**

**MexicoAC**  **ALLRMQWKWLSSGIYFATAGTNIFGKRVFATTRAHYFDFAGFLKLETKSGDPYTHLLTGLNAGVEARVYIPLTYVFYRNNGGYELNRVVPSGIINMPILG**

**GauthierC**  **ALLRMQWKWLSSGIYFATAGTNIFGKRVFATTRAHYFDFAGFLKLETKSGDPYTHLLTGLNAGVEARVYIPLTYVFYKNNGGYPLNGVVPSGTINMPILG**

**CDC2C**  **ALLRMQWKWLSSGIYFATAGTNIFGKRVFATTRAHYFDFAGFLKLETKSGDPYTHLLTGLNAGVEARVYIPLTYVFYKNNGGHPLNGVVPSGTINMPILG**

**SamoaDC**  **ALLRMQWKWLSSGIYFATAGTNIFGKRVFATTRAHYFDFAGFLKLETKSGDPYTHLLTGLNAGVEARVYIPLTYVFYKNNGGYPLNGVVPSGTINMPILG**

**IraqBC**  **ALLRMQWKWLSSGIYFATAGTNIFGKRVFATTRAHYFDFAGFLKLETKSGDPYTHLLTGLNAGVEARVYIPLTYVFYKNNGGYELNGVVPPGIINMPILG**

**BosniaAC**  **ALLRMQWKWLSSGIYFATAGTNIFGKRVFATTRAHYFDFAGFLKLETKSGDPYTHLLTGLNAGVEARVYIPLTYVFYKNNGGYELNGVVPPGIINMPILG**

**Fribourg-BlancC** **ALLRMQWKWLSSGIYFATAGTNIFGKRVFATTRAHYFDFAGFLKLETKSGDPYTHLLTGLNAGVEARVYIPLTYVFYKNNGGHLLDGVVPLGTINMPILG**

**EL10 EL11**

**DVR6 DVR7**

510 520 530 540 550 560 570 580 590

....|....|....|....|....|....|....|....|....|....|....|....|....|....|....|....|....|....|....|...

**NicholsC**  **KAWCSYRIPLGSHAWLAPHTSVLGTTNRFNIINPAGNLLNERALQYQVGLTFSPFEKVELSAQWEQGVLADAPYMGIAESIWSERHFGTLVCGMKVTW**

**Street14C**  **KAWCSYRIPLGSHAWLTPHTSVLGTTNRFNVINPAGNLLNERALQYQVGLTFSPFEKVELSAQWEQGVLADAPYMGITQSIGSDRHFGTLVCGMKVTW**

**Bal3C**  **KAWCSYRIPLGSHAWLAPHTSVLGTTNRFNIINAAGNLLNERALQYQVGLTFSPFEKVELSAQWEQGVLSDVPYMGIAESIWSERHFGTLVCGMKVTW**

**Sea81-4C**  **KAWCSYRIPLGSHAWLAPHTSVLGTTNRFNIINAAGNLLNERALQYQVGLTFSPFEKVELSAQWEQGVLSDVPYMGIAESIWSERHFGTLVCGMKVTW**

**MexicoAC**  **KAWCSYRIPLGSHAWLAPHTSVLGTTNRFNIINAAGNLLNERALQYQVGLTFSPFEKVELSAQWEQGVLADAPYMGITQSIGSDRHFGTLVCGMKVTW**

**GauthierC**  **KAWCSYRIPLGSHAWLAPHTSVLGTTNRFNIINAAGNLVNERALQYQVGLTFSPFEKVELSAQWEQGVLSDVPYMGITQSIWSERHFGTFVCGMKVTW**

**CDC2C**  **KAWCSYRIPLGSHAWLAPHTSVLGTTNRFNIINAAGNLVNERALQYQVGLTFSPFEKVELSAQWEQGVLSDVPYMGITQSIWSERHFGTFVCGMKVTW**

**SamoaDC**  **KAWCSYRIPLGSHAWLAPHTSVLGTTNRFNIINAAGNLVNERALQYQVGLTFSPFEKVELSAQWEQGVLSDVPYMGITQSIWSERHFGTFVCGMKVTW**

**IraqBC**  **KAWCSYRIPLGSHAWLAPHTSVLGTTNRFNIINAAGNLVNERALQYQVGLTFSPFEKVELSAQWEQGVLSDVPYMGIAESIWSERHFGTLVCGMKVTW**

**BosniaAC**  **KAWCSYRIPLGSHAWLAPHTSVLGTTNRFNIINAAGNLVNERALQYQVGLTFSPFEKVELSAQWEQGVLSDVPYMGIAESIWSERHFGTLVCGMKVTW**

**Fribourg-BlancC** **KAWCSYRIPLGSHAWLAPHTSVLGTTNRFNIINAAGNLVNERALQYQVGLTFSPFEKVELSAQWEQGVLSDVPYMGIAESIWSERHFGTFVCGMKVTW**

**2.3**

**SP EL1 EL2**

**DVR1**

10 20 30 40 50 60 70 80 90 100

....|....|....|....|....|....|....|....|....|....|....|....|....|....|....|....|....|....|....|....|

**NicholsI**  **MGRQVMQAGVLAGMVCAASGYAGVLTPQVSGTAQLQWGIAFQKNPRTGPGKHTHGFRTTNSLTISLPLVSKHTHTRRGEARSGVWAQLQLKDLAVELASS**

**Bal-3I**  **MGRQVMQAGVLAGMVCAASGYAGVLTPQVSGTAQLQWGIAFQKNPRTGPGKHTHGFRTTNSLTISLPLVSKHTHTRRGEARSGVWAQLQLKDLAVELASS**

**Sea81-4I**  **MGRQVMQAGVLAGMVCAASGYAGVLTPQVSGTAQLQWGIAFQKNPRTGPGKHTHGFRTTNSLTISLPLVSKHTHTRRGEARSGVWAQLQLKDLAVELASS**

**MexicoAI**  **MGRQVMQAGVLAGMVCAASGYAGVLTPQVSGTAQLQWGIAFQKNPRTGPGKHTHGFRTTNSLTISLPLVSKHTHTRRGEARSGVWAQLQLKDLAVELASS**

**Street14I** **MGRQVMQAGVLAGMVCAASGYAGVLTPQVSGTAQLQWGIAFQKNPRTGPGKHTHGFRTTNSLTISLPLVSKHTHTRRGEARSGVWAQLQLKDLAVELASS**

**GauthierI** **MGRQVMQAGVLAGMVCAASGYAGVLTPQVSGTAQLQWGIAFQKNPHTVPGEHTHGFRTTNSLTISLPLVSKHTHTRRGEARLGVWAQLQLKDLAVELASS**

**SamoaDI**  **MGRQVMQAGVLAGMVCAASGYAGVLTPQVSGTAQLQWGIAFQKNPHTVPGEHTHGFRTTNSLTISLPLVSKHTHTRRGEARSGVWAQLQLKDLAVELASS**

**CDC2I**  **MGRQVMQAGVLAGMVCAASGYAGVLTPQVSGTAQLQWGIAFQKNPHTVPGEHTHGFRTTNSLTISLPLVSKHTHTRRGEARSGVWAQLQLKDLAVELASS**

**BosniaAI**  **MGRQVMQAGVLAGMVCAASGYAGVLTPQVSGTAQLQWGIAFQKNPRTVPGEHTHGFRTTNSLTISLPLVSKHTHTRRGEARSGVWAQLQLKDLAVELASS**

**IraqBI**  **MGRQVMQAGVLAGMVCAASGYAGVLTPQVSGTAQLQWGIAFQKNPRTVPGEHTHGFRTTNSLTISLPLVSKHTHTRRGEARSGVWAQLQLKDLAVELASS**

**FribourgBlancI**  **MGRQVMQAGVLAGMVCAASGYAGVLTPQVSGTAQLQWGIAFQKNPHTVPGEHTHGFRTTNSLTISLPLVSKHTHTRRGEARSGVWAQLQLKDLAVELASS**

**SamoaDF**  **MGRQVMQAGVLAGMVCAASGYAGVLTPQVSGTAQLQWGIAFQKNPHTVPGEHTHGFRTTNSLTISLPLVSKHTHTRRGEARSGVWAQLQLKDLAVELASS**

**GauthierF** **MGRQVMQAGVLAGMVCAASGYAGVLTPQVSGTAQLQWGIAFQKNPHTVPGEHTHGFRTTNSLTISLPLVSKHTHTRRGEARLGVWAQLQLKDLAVELASS**

**CDC2F**  **MGRQVMQAGVLAGMVCAASGYAGVLTPQVSGTAQLQWGIAFQKNPHTVPGEHTHGFRTTNSLTISLPLVSKHTHTRRGEARSGVWAQLQLKDLAVELASS**

**Fribourg-BlancF**  **MGRQVMQAGVLAGMVCAASGYAGVLTPQVSGTAQLQWGIAFQKNPHTVPGEHTHGFRTTNSLTISLPLVSKHTHTRRGEARSGVWAQLQLKDLAVELASS**

**EL3 EL4**

110 120 130 140 150 160 170 180 190 200

....|....|....|....|....|....|....|....|....|....|....|....|....|....|....|....|....|....|....|....|

**NicholsI**  **KSSTALSFTKPTASFQATLHCYGAYLTVGTSPSCVVNFAQLWKPFVTRAYSEKDTRYAPGFSGSGAKLGYQAHNVGNSGVDVDIGFLSFLSNGAWDSTDT**

**Bal-3I**  **KSSTALSFTKPTASFQATLHCYGAYLTVGTSPSCVVNFAQLWKPFVTRAYSEKDTRYAPGFSGSGAKLGYQAHNVGNSGVDVDIGFLSFLSNGAWDSTDT**

**Sea81-4I**  **KSSTALSFTKPTASFQATLHCYGAYLTVGTSPSCVVNFAQLWKPFVTRAYSEKDTRYAPGFSGSGAKLGYQAHNVGNSGVDVDIGFLSFLSNGAWDSTDT**

**MexicoAI**  **KSSTALSFTKPTASFQATLHCYGAYLTVGTSPSCVVNFAQLWKPFVTRAYSEKDTRYAPGFSGSGAKLGYQAHNVGNSGVDVDIGFLSFLSNGAWDSTDT**

**Street14I** **KSSTALSFTKPTASFQATLHCYGAYLTVGTSPSCVVNFAQLWKPFVTRAYSEKDTRYAPGFSGSGAKLGYQAHNVGNSGVDVDIGFLSFLSNGAWDSTDT**

**GauthierI** **KSSTALSFTKPTASFQATLHCYGAYLTVGTSPSCVVNFAQLWKPFVTRAYSEKDTRYAPGFSGSGAKLGYQAHNVGNSGVDVDIGFLSFLSNGAWDSTDT**

**SamoaDI**  **KSSTALSFTKPTASFQATLHCYGAYLTVGTSPSCVVNFAQLWKPFVTRAYSEKDTRYAPGFSGSGAKLGYQAHNVGNSGVDVDTGFLSFLSNGAWDSTDP**

**CDC2I**  **KSSTALSFTKPTASFQATLHCYGAYLTVGTSPSCVVNFAQLWKPFVTRAYSEKDTRYAPGFSGSGAKLGYQAHNVGNSGIDVDIGFLSFLSNGAWDSTDT**

**BosniaAI**  **KSSTALSFTKPTASFQATLHCYGAYLTVGTSPSCVVNFAQLWKPFVTRAYSEKDTRYAPGFSGSGAKLGYQAHNVGNSGVDVDIGFLSFLSNGAWDSTDT**

**IraqBI**  **KSSTALSFTKPTASFQATLHCYGAYLTVGTSPSCVVNFAQLWKPFVTRAYSEKDTRYAPGFSGSGAKLGYQAHNVGNSGVDVDIGFLSFLSNGAWDSTDT**

**Fribourg-BlancI**  **KSSTALSFTKPTASFQATLHCYGAYLTVGTSPSCVVNFAQLWKPFVTRAYSEKDTRYAPGFSGSGAKLGYQAHNVGNSGVDVDTGFLSFLSNGAWDSTDP**

**SamoaDF**  **KSSTALSFTKPTASFQATLHCYGAYLTVGTSPSCVVNFAQLWKPFVTRACSEKDTRYAPGFSGSGAKLGYQAHNVGNSGVDVDIGFLSFLSNGAWDSTDP**

**GauthierF** **KSSTALSFTKPTASFQATLHCYGAYLTVGTSPSCVVNFAQLWKPFVTRAYSEKDTRYAPGFSGSGAKLGYQAHNVGNSGVDVDIGFLSFLSNGAWDSTDT**

**CDC2F**  **KSSTALSFTKPTASFQATLHCYGAYLTVGTSPSCVVNFAQLWKPFVTRAYSEKDTRYAPGFSGSGAKLGYQAHNVGNSGVDVDIGFLSFLSNGAWDSTDT**

**Fribourg-BlancF**  **KSSTALSFTKPTASFQATLHCYGAYLTVGTSPSCVVNFAQLWKPFVTRAYSEKDTRYAPGFSGSGAKLGYQAHNVGNSGVDVDIGFLSFLSNGAWDSTDA**

**EL5 EL6**

**DVR3 DVR4**

210 220 230 240 250 260 270 280 290 300

....|....|....|....|....|....|....|....|....|....|....|....|....|....|....|....|....|....|....|....|

**NicholsI**  **THSKYGFGADATLSYGVDRQRLLTLELAGNATLEQHYRKGTEDSTNENKTALLWGVGGRLTLEPGAGFRFSFALDAGNQHQDPADAGNRLLATGSSREKF**

**Bal-3I**  **THSKYGFGADATLSYGVDRQRLLTLELAGNATLEQHYRKGTEDSTNENKTALLWGVGGRLTLEPGAGFRFSFALDAGNQHQDPADAGNRLLATGSSREKF**

**Sea81-4I**  **THSKYGFGADATLSYGVDRQRLLTLELAGNATLEQHYRKGTEDSTNENKTALLWGVGGRLTLEPGAGFRFSFALDAGNQHQDPADAGNRLLATGSSREKF**

**MexicoAI**  **THSKYGFGADATLSYGVDRQRLLTLELAGNATLEQHYRKGTEDSTNENKTALLWGVGGRLTLEPGAGFRFSFALDAGNQHQDPADAGNRLLATGSSREKF**

**Street14I** **THSKYGFGADATLSYGVDRQRLLTLELAGNATLDQNYVKGTEDSTNENKTALLWGVGGRLTLEPGAGFRFSFALDAGNQHQDPADAGNRLLATGSSREKF**

**GauthierI** **THSKYGFGADATLSYGVDRQRLLTLELAGNATLEQHYRKGTEDSPNENKTALPWGVGGRLTLEPGAGFRFSFALDAGNQHQSNADADCRLPATGNSREKF**

**SamoaDI**  **THSKYGFGADATLSYGVDRQRLLTLELAGNATLEQHYRKGTEDSTNENKTALLWGVGGRLTLEPGAGFRFSFALDAGNQHQSNADADCRLPATGSSREKF**

**CDC2I**  **THSKYGFGADATLSYGVDRQRLLTLELAGNATLEQHYRKGTEDSTNENKTALLWGVGGRLTLEPGAGFRFSFALDAGNQHQSNADADCRLPATGSSREKF**

**BosniaAI**  **MHSKYGFGADATLSYGVDRQRLLTLELAGNATLEQHYRKGTEDSTNENKTALLWGVGGRLTLEPGAGFRFSFALDAGNQHQSNADADCRLPATGSSREKF**

**IraqBI**  **MHSKYGFGADATLSYGVDRQRLLTLELAGNATLEQHYRKGTEDSTNENKTALLWGVGGRLTLEPGAGFRFSFALDAGNQHQSNADADCRLPATGSSREKF**

**Fribourg-BlancI**  **THSKYGFGADATLSYGVDRQRLLTLELAGNATLEQHYRKGTEDSTNENKTALLWGVGGRLTLEPGAGFRFSFALDAGNQHQSDADADCRLPATGSSREKF**

**SamoaDF**  **THSKYGFGADATLSYGVDRQRLLTLELAGNATLEQHYRKGTEDSTNENKTALLWGVGGRLTLEPGAGFRFSFALDAGNQHQSNADADCRLPATGSSREKF**

**GauthierF** **THSKYGFGADATLSYGVDRQRLLTLELAGNATLEQHYRKGTEDSPNENKTALPWGVGGRLTLEPGAGFRFSFALDAGNQHQSNADADCRLPATGNSREKF**

**CDC2F**  **THSKYGFGADATLSYGVDRQRLLTLELAGNATLEQHYRKGTEDSTNENKTALLWGVGGRLTLEPGAGFRFSFALDAGNQHQSNADADCRLPATGSSREKF**

**Fribourg-BlancF**  **THSKYGFGADATLSYGVDRQRLLTLELAGNATLEQHYRKGTEDSTNENKTALLWGVGGRLTLEPGAGFRFSFALDAGNQHQSDADADCRLPATGSSREKF**

**EL7**

**DVR5**

310 320 330 340 350 360 370 380 390 400

....|....|....|....|....|....|....|....|....|....|....|....|....|....|....|....|....|....|....|....|

**NicholsI**  **DSAFDALRVEQYRVKDKYLEFLLGQMAESSILERVGLALTLQDGTLVSTLTKVATDSGDRFIQMALVKLLPQRAQAEQRLQEIVAPSQSDIVLIMLLTWL**

**Bal-3I**  **DSAFDALRVEQYRVKDKYLEFLLGQMAESSILERVGLALTLQDGTLVSTLTKVATDSGDRFIQMALVKLLPQRAQAEQRLQEIVAPSQSDIVLIMLLTWL**

**Sea81-4I**  **DSAFDALRVEQYRVKDKYLEFLLGQMAESSILERVGLALTLQDGTLVSTLTKVATDSGDRFIQMALVKLLPQRAQAEQRLQEIVAPSQSDIVLIMLLTWL**

**MexicoAI**  **DSAFDALRVEQYRVKDKYLEFLLGQMAESSILERVGLALTLQDGTLVSTLTKVATDSGDRFIQMALVKLLPQRAQAEQRLQEIVAPSQSDIVLIMLLTWL**

**Street14I** **DSAFDALRVEQYRVKDKYLEFLLGQMAESSILERVGLALTLQDGTLVSTLTKVATDSGDRFIQMALVKLLPQRAQAEQRLQEIVAPSQSDIVLIMLLTWL**

**GauthierI** **DRAFDALRVEQYRVKDKYLEFLLGQTAESSILERVGLALTLQDGTLVSTLTKVATDSGDRFIQMALVKLLPQRAQAEQGLREIVAPSQSDIVLIMLLTWL**

**SamoaDI**  **DRAFDALRVEQYRVKDKYLEFLLGQMAESSILERVGLALTLQDGTLVSTLTKVATDSGDRFIQMALVKLLPQRAQAEQGLREIVAPSQSDIVLIMLLTWL**

**CDC2I**  **DRAFDALRVEQYRVKDKYLEFLLGQMAESSILERVGLALTLQDGTLVSTLTKVATDSGDWFIQMALVKLLPQRAQAEQGLREIVAPSQSDIVLIMLLTWL**

**BosniaAI**  **DRAFDALRVEQYRVKDKYLEFLLGQMAESSILERVGLALTLQDGTLVSTLTKVATDSGDRFIQMALVKLLPQRAQAEQGLREIVAPSQSDIVLIMLLTWL**

**IraqBI**  **DRAFDALRVEQYRVKDKYLEFLLGQMAESSILERVGLALTLQDGTLVSTLTKVATDSGDRFIQMALVKLLPQRAQAEQGLREIVAPSQSDIVLIMLLTWL**

**Fribourg-BlancI**  **DRAFDALRVEQYYVKDKYLEFLLGQMAESSILERVGLALTLQDGTLVSTLTKVATDSGDQFIQMALVKLLPQRAQAEQGLREIVAPSQSDIVLIMLLTWL**

**SamoaDF**  **DRAFDALRVEQYRVKDKYLEFLLGQMAESSILERVGLALTLQDGTLVSTLTKVATDSGDQFIQMALVKLLPQRAQAEQGLREIVAPSQSDIVLIMLLTWL**

**GauthierF** **DRAFDALRVEQYRVKDKYLEFLLGQTAESSILERVGLALTLQDGTLVSTLTKVATDSGDRFIQMALVKLLPQRAQAEQGLREIVAPSQSDIVLIMLLTWL**

**CDC2F**  **DRAFDALRVEQYRVKDKYLEFLLGQMAESSILERVGLALTLQDGTLVSTLTKVATDSGDWFIQMALVKLLPQRAQAEQGLREIVAPSQSDIVLIMLLTWL**

**Fribourg-BlancF**  **DRAFDALRVEQYYVKDKYLEFLLGQMAESSILERVGLALTLQDGTLVSTLTKVATDSGDRFIQMALVKLLPQRAQAEQGLREIVAPSQSDIVLIMLLTWL**

**EL8 EL9**

**DVR6 DVR7**

410 420 430 440 450 460 470 480 490 500

....|....|....|....|....|....|....|....|....|....|....|....|....|....|....|....|....|....|....|....|

**NicholsI**  **ERARLDRFNADALLTAQWTYVSAGLYGATAGTNVFGKRVLPALRSWHFDFAGFLKLETKSGDPYTHLLTGLNAGVEARVYIPLTYIRYRNNGGYELNGAV**

**Bal-3I**  **ERARLDRFNADALLTAQWTYVSAGLYGATAGTNVFGKRVLPALRSWHFDFAGFLKLETKSGDPYTHLLTGLNAGVEARVYIPLTYIRYRNNGGYELNGAV**

**Sea81-4I**  **ERARLDRFNADALLTAQWTYVSAGLYGATAGTNVFGKRVLPALRSWHFDFAGFLKLETKSGDPYTHLLTGLNAGVEARVYIPLTYIRYRNNGGYELNGAV**

**MexicoAI**  **ERARLDRFNADALLTAQWTYVSAGLYGATAGTNVFGKRVLPALRSWHFDFAGFLKLETKSGDPYTHLLTGLNAGVEARVYIPLTYIRYRNNGGYELNGAV**

**Street14I** **ERARLDRFNADALLTAQWTYVSAGLYGATAGTNVFGKRVLPALRSWHFDFAGFLKLETKSGDPYTHLLTGLNAGVEARVYIPLTYIRYRNNGGYELNGAV**

**GauthierI** **ERARLDRFNADALLTAQWTYVSAGLYGATAGTNVFGKRVLPALQSWHFDFAGFLKLETKSGDPYTHLLTGLNAGVEARVYIPLTYIRYRNNGGYPLNGVV**

**SamoaDI**  **ERARLDRFNADALLTAQWTYVSAGLYGATAGTNVFGKRVLPALQSWHFDFAGFLKLETKSGDPYTHLLTGLNAGVEARVYIPLTYIRYRNNGGYPLNGVV**

**CDC2I**  **ERARLDRFNADALLTAQWTYVSAGLYGATAGTNVFGKRVLPALQSWHFDFAGFLKLETKSGDPYTHLLTGLNAGVEARVYIPLTYIRYRNNGGYPLNGVV**

**BosniaAI**  **ERARLDRFNADALLTAQWTYVSAGLYGATAGTNVFGKRVLPALQSWHFDFAGFLKLETKSGDPYTHLLTGLNAGVEARVYIPLTYIRYRNNGGYPLNGVV**

**IraqBI**  **ERARLDRFNADALLTAQWTYVSAGLYGATAGTNVFGKRVLPALQSWHFDFAGFLKLETKSGDPYTHLLTGLNAGVEARVYIPLTYIRYRNNGGYPLNGVV**

**Fribourg-BlancI**  **ERARLDRFNADALLTAQWTYVSAGLYGATAGTNVFGKRVLPALQSWHFDFAGFLKLETKSGDPYTHLLTGLNAGVEARVYIPLTYIRYRNNGGYLLDGVV**

**SamoaDF**  **ERARLDRFNADALLTAQWTYVSAGLYGATAGTNVFGKRVLPALQSWHFDFAGFLKLKTKSGDPYTHLLTGLNAGVEARVYIPLTYVRYRNNGGYPLNGVV**

**GauthierF** **ERARLDRFNADALLTAQWTYVSAGLYGATAGTNVFGKRVLPALQSWHFDFAGFLKLETKSGDPYTHLLTGLNAGVEARVYIPLTYIRYRNNGGYPLNGVV**

**CDC2F**  **ERARLDRFNADALLTAQWTYVSAGLYGATAGTNVFGKRVLPALQSWHFDFAGFLKLETKSGDPYTHLLTGLNAGVEARVYIPLTYIRYRNNGGYPLNGVV**

**Fribourg-BlancF**  **ERARLDRFNADALLTAQWTYVSAGLYGATAGTNVFGKRVLPALQSWHFDFAGFLKLETKSGDPYTHLLTGLNAGVEARVYIPLTYIRYRNNGGYLLDGVV**

**EL1O EL11**

**DVR8 DVR9**

510 520 530 540 550 560 570 580 590 600

....|....|....|....|....|....|....|....|....|....|....|....|....|....|....|....|....|....|....|....|

**NicholsI**  **PPGTINMPILGKAWCSYRIPLGSHAWLTPHTSVLGTTNRFNVINPAYTLLNERALQYQVGLTFSPFEKVELSAQWEQGVLADAPYMGIAESMWSERYFGT**

**Bal-3I**  **PPGTINMPILGKAWCSYRIPLGSHAWLTPHTSVLGTTNRFNVINPAYTLLNERALQYQVGLTFSPFEKVELSAQWEQGVLADAPYMGIAESMWSERYFGT**

**Sea81-4I**  **PPGTINMPILGKAWCSYRIPLGSHAWLTPHTSVLGTTNRFNVINPAYTLLNERALQYQVGLTFSPFEKVELSAQWEQGVLADAPYMGIAESMWSERYFGT**

**MexicoAI**  **PPGTINMPILGKAWCSYRIPLGSHAWLTPHTSVLGTTNRFNVINPAYTLLNERALQYQVGLTFSPFEKVELSAQWEQGVLADAPYMGIAESMWSERYFGT**

**Street14I** **PPGTINMPILGKAWCSYRIPLGSHAWLTPHTSVLGTTNRFNVINPAYTLLNERALQYQVGLTFSPFEKVELSAQWEQGVLADAPYMGIAESMWSERYFGT**

**GauthierI** **PPGTINMPILGKAWCSYRIPLGSHAWLTPHTSVLGTTNRFNVINPAHTLLNERALQYQVGLTFSPFEKVELSAQWEQGVLADAPYMGIAEGMWSERYFGT**

**SamoaDI**  **PPGTINMPILGKAWCSYRIPLGSHAWLTPHTSVLGTTNRFNVINPAYTLLNERALQYQVGLTFSPFEKVELSAQWEQGVLADAPYMGIAESMWSERYFGT**

**CDC2I**  **PPGTINMPILGKAWCSYRIPLGSHAWLTPHTSVLGTTNRFNVINPAYTLLNERALQYQVGLTFSPFEKVELSAQWEQGVLADAPYMGIAESMWSERYFGT**

**BosniaAI**  **PPGTINMPILGKAWCSYRIPLGSHAWLTPHTSVLGTTNRFNVINPAYTLLNERALQYQVGLTFSPFEKVELSAQWEQGVLSDVPYMGIAESMWSERYFGT**

**IraqBI**  **PPGTINMPILGKAWCSYRIPLGSHAWLTPHTSVLGTTNRFNVINPAYTLLNERALQYQVGLTFSPFEKVELSAQWEQGVLSDVPYMGIAESMWSERYFGT**

**Fribourg-BlancI**  **PPGTINMPILGKAWCSYRIPLGSHAWLTPHTSVLGTTNRFNVINPAYTLLNERALQYQVGLTFSPFEKVELSAQWEQGVLADAPYMGIAESMWSERYFGT**

**SamoaDF**  **PPGTINMPILGKAWCSYRIPLGSHAWLTPHTSVLGTTNRFNVINPAYTLLNERALQYQVGLTFSPFEKVELSAQWEQGVLADAPYMGIAESMWSERYFGT**

**GauthierF** **PPGTINMPILGKAWCSYRIPLGSHAWLTPHTSVLGTTNRFNVINPAHTLLNERALQYQVGLTFSPFEKVELSAQWEQGVLADAPYMGIAEGMWSERYFGT**

**CDC2F**  **PPGTINMPILGKAWCSYRIPLGSHAWLTPHTSVLGTTNRFNVINPAYTLLNERALQYQVGLTFSPFEKVELSAQWEQGVLADAPYMGIAESMWSERYFGT**

**Fribourg-BlancF**  **PPGTINMPILGKAWCSYRIPLGSHAWLTPHTSVLGTTNRFNVINPAYTLLNERALQYQVGLTFSPFEKVELSAQWEQGVLADAPYMGIAESMWSERYFGT**

610

....|....|

**NicholsI**  **FICGVKVVW**

**Bal-3I**  **FICGVKVVW**

**Sea81-4I**  **FICGVKVVW**

**MexicoAI**  **FICGVKVVW**

**Street14I** **FICGVKVVW**

**GauthierI** **FICGVKVVW**

**SamoaDI**  **FICGVKVVW**

**CDC2I**  **FICGVKVVW**

**BosniaAI**  **FICGVKVVW**

**IraqBI**  **FICGVKVVW**

**Fribourg-BlancI**  **FICGVKVVW**

**SamoaDF**  **FICGVKVVW**

**GauthierF** **FICGVKVVW**

**CDC2F**  **FICGVKVVW**

**Fribourg-BlancF**  **FICGVKVVW**
