# Supplementary material for: Fine Analysis of Genetic Diversity of the tpr Gene Family among Treponemal Species, Subspecies and Strains
Source: PLoS Negl Trop Dis. 2013 May 16;7(5):e2222. doi: 10.1371/journal.pntd.0002222 (PMC3656149; doi:10.1371/journal.pntd.0002222)
Supplement: Table S1 — Primers used for amplification (1) or sequencing (2). (DOCX) [file pntd.0002222.s003.docx]

| **Locus** | **Primer** | **Sequence** | **Use** |
| --- | --- | --- | --- |
| *tprA* | TPRA- L | CGTATGCTTTTACCCGCTGT | 1 |
|  | TPRA- R | CGTAATCGAAGATGGTTGCA | 1 |
|  | NICHOLS A2SENSE | TAAGGAACCTAGAGTGTGCG | 2 |
|  | ARTPCR-S | TACCTACCGGGATACGAACAGT | 2 |
|  | ARTPCR-AS | TGCAAGGCATGGGTGTAATCAT | 2 |
|  | CUNIA1S | TTAGGCGCGAAGGCGAACTTC | 2 |
|  | CUNIA1AS | TAGGGTGCAAGATCACTTGG*T* | 2 |
|  | M13 FWD | GTAAAACGACGGCCAGTG | 2 |
|  | M13 REV | GGTCATAGCTGTTTCCTG | 2 |
| *tprB* | TPRBFLK2-S | CTTCGCGCCTAAGTTAATGC | 1 |
|  | TPRBFLK2-AS | GTAGGGGATCCGCATGAGTA | 1 |
|  | CUNI-B-1S | GTGAGGATCCCATATGGGT | 2 |
|  | CUNI-B2-S | TTTCGCTGGGAGCATACGTA | 2 |
|  | BRTPCRS | AGTCACCACCAGGTGTGTGG | 2 |
|  | BRTPCRAS | ACGATTCTCTTTCTAAGCTTGTGTC | 2 |
|  | TPRB5-SENSE | TATTCGTGGGTGGGAAGAAG | 2 |
|  | M13 FWD | GTAAAACGACGGCCAGTG | 2 |
|  | M13 REV | GGTCATAGCTGTTTCCTG | 2 |
| *tprC* | C2L | GGGGGTGAGGTAGAAGTGAGA | 1 |
|  | CR | TAATACCTCACATTCCCCCG | 1 |
|  | CUNITPRC739S | CACCCGTGCCTATTCAGAAA | 2 |
|  | CUNITPRC/D712AS | CCACACTGGACCAGAACTACG | 2 |
|  | TPRD1469AS | GAACTGAATGGAGCTGTGCC | 2 |
|  | TPRC2-R | ATTTTGTCTCGGATGAGGG | 2 |
|  | CUNI-C-1S | TGGGATAGTACTGACACCAC | 2 |
|  | M13 FWD | GTAAAACGACGGCCAGTG | 2 |
|  | M13 REV | GGTCATAGCTGTTTCCTG | 2 |
|  |  |  |  |
| *tprD* | DCRP | TATAGGTGCGCCGGGTTC | 1 |
|  | DF I-19 AS | TCTTTCTCACCAATGCCATG | 1 |
|  | DL | AAGAGGTTCAGGAAGCAACG | 1 |
|  | DR | ACTTCGTAGGAGCAGCAGGA | 1 |
|  | CUNI-D2AS | GGGATAGTACTGACACCACG | 2 |
|  | D131 I-1 AS | ACCACACACATCCGTGCTC | 2 |
|  | RTPCR C/D AS | CCAAGAGTTGTCACTGCTAAA | 2 |
|  | DSENSELC | ACATATTCCCTAGACGGGT | 2 |
|  | M13 FWD | GTAAAACGACGGCCAGTG | 2 |
|  | M13 REV | GGTCATAGCTGTTTCCTG | 2 |
| *tprE* | EL | CAGGATTTTCCGGTTCATTGG | 1 |
|  | E314R | GAGAGCATACCCTGTTCCCA | 1 |
|  | ECRP | CATGGTTTCGGTTGTACCC | 1 |
|  | ER | GCAGAACATTAAACGCGTGA | 1 |
|  | EFLANKING SENSE | CGTTGAATGCCGTTATTGTG | 1 |
|  | EFLANKING AS | TCAAACTTCTCCCGTGAG | 1 |
|  | CUNIE1S | GAGGTAGCATGGGGTGCA | 2 |
|  | CUNIE2S | AAGGACGCAAACAATCAGAAC | 2 |
|  | CUNIE1AS | GAGCAGTAGGAGCTACCTAT | 2 |
|  | TPRE-314AS | AATGCCAAACGAAACGGTAG | 2 |
|  | ECRPS | CATGGTTTCGGTTGTACCC | 2 |
|  | M13 FWD | GTAAAACGACGGCCAGTG | 2 |
|  | M13 REV | GGTCATAGCTGTTTCCTG | 2 |
| *tprF* | FL | TGTAGGGGTCACCGCTCTTA | 1 |
|  | FR | AGACCGGGGGAAGGTAGTAC | 1 |
|  | M13 FWD | GTAAAACGACGGCCAGTG | 2 |
|  | M13 REV | GGTCATAGCTGTTTCCTG | 2 |
| *tprG* | GL | GCATCGATGACCAGGAACTT | 1 |
|  | RTFAS | GAACAGGGGGTGCTTGCTGA | 1 |
|  | FR | AGACCGGGGGAAGGTAGTAC | 1 |
|  | GEND1 | TCACCCTCTCCTGGTAGTC | 1 |
|  | GPRO1 | CCCTGCGTTTCCCATCTG | 2 |
|  | GPRO3 | AAACATGCGCAAAATAAGGG | 1,2 |
|  | GFWD4 | TGCGCACCACCCACACGCGCCG | 2 |
|  | CUNI GF2S | GGTCAGCCTTGATGTGGGG | 2 |
|  | FL | TAAGAGCGGTGACCCCTACA | 2 |
|  | RTPCR I-AS | AGTCAGTCGGACATCGTGCTTA | 2 |
|  | M13 FWD | GTAAAACGACGGCCAGTG | 2 |
|  | M13 REV | GGTCATAGCTGTTTCCTG | 2 |
|  | RTPCR G-S | GAAGGTGTTCATTACCGACCCT | 2 |
|  | RTPCR G-AS | TTGTAGCCTCAGCCGTAAGCTT | 2 |
| *tprH* | CUNI-H-1S | TCGAGCGCTTGCTGCAGTA | 1 |
|  | TPRHFLANKINGAS | CGTTCCAAACTCCTGAGGAA | 1 |
|  | TPRH2S | AAGCGACGCTACACTGCTTT | 2 |
|  | CUNI-H-1AS | ACAGAAAATCTAGTTCAT | 2 |
|  | HRTPCRS | GCAGAAGCTCGATAGTGTCAAG | 2 |
|  | HRTPCR-AS | TTTCCTACGTATGGGAGCA | 2 |
|  | M13 FWD | GTAAAACGACGGCCAGTG | 2 |
|  | M13 REV | GGTCATAGCTGTTTCCTG | 2 |
| *tprI* | I2L | CGTCACCCTCTCCTGGTAGT | 1 |
|  | I2R | TCAGTTTACAGGCGAGGGAT | 1 |
|  | RTPCR I-AS | AGTCAGTCGGACATCGTGCTTA | 2 |
|  | M13 FWD | GTAAAACGACGGCCAGTG | 2 |
|  | M13 REV | GGTCATAGCTGTTTCCTG | 2 |
| *tprJ* | JL | CGAGTGAGGCTCATCAAGAA | 1 |
|  | JPROM1 | AAGTTTGCTTTCAGAT | 1 |
|  | JPROM2 | AAAGAAAAAGGATTTCCGCA | 1 |
|  | JR | TGTGTGGTTAACTTTGCCCA | 1 |
|  | J2R | GGTAGTCACACGCGGCTAGA | 1 |
|  | CUNIGF1S/JIAS | ACCTGCAGGCGGACCTGA | 2 |
|  | IBEG1 | ACAGCTGCGTGCTGGTATTT | 2 |
|  | FL | TAAGAGCGGTGACCCCTACA | 2 |
|  | GFSEQ1 | CGGCGTCACCCTCTCCTGG | 2 |
|  | CDCPERTGFSAS | CGCTCGAGAATCGAGGACT | 2 |
|  | JIGF4 | TGAGAGGAGGGGGAGTGAGTG | 2 |
|  | RTJS | TCTTCACACCCCGCAGGGAA | 2 |
|  | RTJAS | CGTTATTTCCGTTCGCATCATC | 2 |
|  | RTI | GACCCTGCCGATGCAGGTAAT | 2 |
|  | JI2S | GAAGCCGTGGGTGA | 2 |
|  | M13 FWD | GTAAAACGACGGCCAGTG | 2 |
|  | M13 REV | GGTCATAGCTGTTTCCTG | 2 |
| *tprL* | L2 | TTTTAAACGTTTCGTGCGCT | 1 |
|  | R5 | AAGACGCCGTCACGTACAGA | 1 |
|  | RTPCRL-S | GGTGGTTTCCCATTTGGAAGG | 2 |
|  | RTPCRL-AS | CAAGTAGTCTGTAAGCTGCCTG | 2 |
|  | M13 FWD | GTAAAACGACGGCCAGTG | 2 |
|  | M13 REV | GGTCATAGCTGTTTCCTG | 2 |
