# Supplementary material for: Fine Analysis of Genetic Diversity of the tpr Gene Family among Treponemal Species, Subspecies and Strains
Source: PLoS Negl Trop Dis. 2013 May 16;7(5):e2222. doi: 10.1371/journal.pntd.0002222 (PMC3656149; doi:10.1371/journal.pntd.0002222)
Supplement: Table S2 — GenBank accession numbers per locus and strain. (DOCX) [file pntd.0002222.s004.docx]

| **Strain/*tpr* locus** | **GenBank accession number** |
| --- | --- |
| Bal3A | JX079747 |
| MexicoAA | JX079748 |
| Sea81-4A | JX079749 |
| GauthierA | JX079750 |
| SamoaDA | JX079751 |
| CDC2A | JX079752 |
| BosniaAA | JX079753 |
| IraqBA | JX079754 |
| Fribourg-BlancA | JX079755 |
| Bal3B | JX079756 |
| Sea81-4B | JX079757 |
| MexicoAB | JX079758 |
| GauthierB | JX079759 |
| SamoaDB | JX079760 |
| CDC2B | JX079761 |
| BosniaAB | JX079762 |
| IraqBB | JX079763 |
| Fribourg-BlancB | JX079764 |
| Bal3C | JX079765 |
| Sea81-4C | JX079766 |
| MexicoAC | JX07976 |
| GauthierC | JX079768 |
| CDC2C | JX079769 |
| SamoaDC | DQ886671 |
| Fribourg-BlancC | JX079770 |
| IraqBC | JX079771 |
| BosniaAC | DQ886673 |
| IraqBD | JX079772 |
| Bal-3D | JX079773 |
| MexicoAD | JX079774 |
| Sea81-4D | JX079775 |
| SamoaDD | JX079776 |
| GauthierD | JX079777 |
| CDC2D | JX079778 |
| BosniaAD | JX079779 |
| Fribourg-BlancD | JX079780 |
| Bal3E | JX079781 |
| Sea81-4E | JX079782 |
| MexicoAE | JX079783 |
| GauthierE | JX079784 |
| SamoaDE | JX079785 |
| CDC2E | JX079786 |
| BosniaE | JX079787 |
| IraqE | JX079788 |
| Fribourg-BlancE | JX079789 |
| Bal3F | JX079790 |
| Sea81-4F | JX079791 |
| MexicoAF | JX079792 |
| GauthierF | JX079793 |
| SamoaDF | JX079794 |
| CDC2F | JX079795 |
| Fribourg-BlancF | JX079796 |
| Bal-3I | JX079797 |
| Sea81-4I | JX079798 |
| MexicoAI | DQ886682 |
| GauthierI | DQ886680 |
| SamoaI | JX079799 |
| CDC2I | JX079800 |
| BosniaI | DQ886678 |
| IraqI | DQ886679 |
| Fribourg-BlancI | JX079801 |
| Bal3G | JX079802 |
| MexicoAG | JX079803 |
| GauthierG | DQ886676 |
| CDC2G | JX079804 |
| Sea81-4G | JX079805 |
| BosniaAG | JX079806 |
| IraqAG | JX079807 |
| Fribourg-BlancG | JX079808 |
| Bal-3H | JX079809 |
| Sea81-4H | JX079810 |
| MexicoAH | JX079811 |
| GauthierH | JX079812 |
| SamoaDH | JX079813 |
| CDC2H | JX079814 |
| BosniaAH | JX079815 |
| IraqBH | JX079816 |
| Fribourg-BlancH | JX079817 |
| Bal3J | JX079818 |
| MexicoAJ | JX079819 |
| Sea81-4J | DQ886674 |
| GauthierJ | JX079820 |
| CDC2J | JX079821 |
| Fribourg-BlancJ | JX079823 |
| IraqJ | JX079822 |
| BosniaAJ | JX079824 |
| Bal-3L | JX079825 |
| Sea81-4L | JX079826 |
| MexicoAL | JX079827 |
| BosniaAtprL | JX079828 |
| IraqBtprL | JX079829 |
| GauthierL | JX079830 |
| SamoaDL | JX079831 |
| CDC2L | JX079832 |
| Fribourg-BlancL | JX079833 |
